# Switchable Cycloadditions of Mesoionic Dipoles: Refreshing up a Regioselective Approach to Two Distinctive Heterocycles

**DOI:** 10.1021/acs.joc.2c01444

**Published:** 2022-09-14

**Authors:** M. Pilar Romero-Fernández, Pedro Cintas, Sergio Rojas-Buzo

**Affiliations:** Department of Organic and Inorganic Chemistry, Faculty of Sciences, and IACYS-Green Chemistry and Sustainable Development Unit, University of Extremadura, 06006 Badajoz, Spain

## Abstract

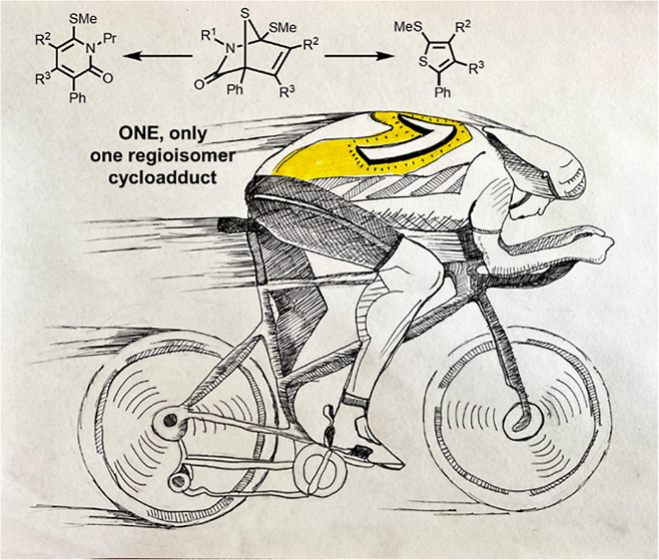

Mesoionic rings are among the most versatile 1,3-dipoles, as witnessed recently by their incorporation into bio-orthogonal strategies, and capable of affording unconventional heterocycles beyond the expected scope of Huisgen cycloadditions. Herein, we revisit in detail the reactivity of thiazol-3-ium-4-olates with alkynes, leading to thiophene and/or pyrid-2-one derivatives. A structural variation at the parent mesoionic dipole alters sufficiently the steric outcome, thereby favoring the regioselective formation of a single transient cycloadduct, which undergoes chemoselective fragmentation to either five- or six-membered heterocycles. The synthetic protocol benefits largely from microwave (MW) activation, which enhances reaction rates. The mechanism has been interrogated with the aid of density functional theory (DFT) calculations, which sheds light into the origin of the regioselectivity and points to a predictive formulation of reactivity involving competing pathways of mesoionic cycloadditions.

## Introduction

The synthetic elaboration of heterocyclic units represents a common and indispensable strategy toward pharmaceuticals and agrochemicals, among other high-value added compounds.^1^ In general, there exist multiple and complementary protocols leading to a given heterocyclic scaffold, while routes capable of producing two or more different structures by merely changing experimental conditions or substitution patterns are unusual. The latter may however be achieved through bifurcated mechanisms involving a common intermediate. In context, cycloadditions with mesoionic dipoles have enough tactic versatility to produce either stable or labile cycloadducts, which lead to new rings through selective bond cleavage.^2^ By definition, mesoionics are five-membered heterocycles that cannot be represented by any Lewis structure not involving charge separation and comprising a sextet of electrons. The electronic stabilization render them stable enough to be isolated in most cases, yet lacking a conclusive aromaticity.^3^ The use of these non-conventional dipoles in synthetic pursuits is well established, albeit recent advances embracing transition metal catalysis, domino reactions, and asymmetric variations have been disclosed during the past two decades.^2^ Of particular interest is the application of some mesoionic rings, notably sydnones, to bio-orthogonal chemistry compatible with *in vivo* environments.^4^ Computational screening also unveils the origin and steric control of fast copper-free strain-promoted cycloadditions of some mesoionics and mesomeric betaines, with cyclooctynes.^5^

**Figure 1:**
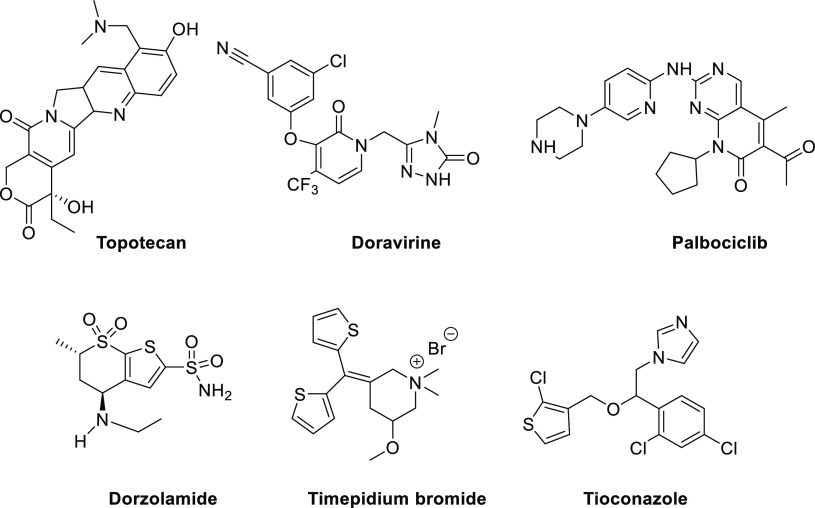
Some FDA-approved small molecule drugs containing 2-pyridone or thiophene units.

The simultaneous incorporation of O, N, and S heteroatoms into mesoionic dipoles enhances the diversity toward highly functionalized heterocyclic targets, which is well portrayed by thiazol-3-ium-4-olates (widely dubbed thioisomünchnones). This class of masked thiocarbonyl ylide dipoles are willing partners against a broad range of dipolarophiles (double and triple bonds and heterocumulenes).^2b,6^ Reactions of thioisomünchones with alkynes provide straightforward access to either pyrid-2-one or thiophene nuclei, two privileged scaffolds in drug design, as witnessed by a series of blockbuster drugs (Figure 1),^7^ with thiophenes being common bioisosteres of the phenyl ring.^8^

Despite their apparent simplicity with pioneering studies dating back to the mid-1970s, such cycloadditions often lack an appropriate rationale. Some transformations exhibit high levels of regiocontrol and diastereocontrol, but selectivity is largely substrate-dependent and product mixtures are also encountered. In general, sulfur extrusion appears to be a dominant pathway starting from aryl-substituted thioisomünchnones at C-2 and C-3, leading to 2-pyridones. The aromatic group at C-2 does not alter this outcome in reactions with acetylenedicarboxylates. A third phenyl group at C-5 slows down the reaction rate, and thiophenes are obtained as major products through isocyanate elimination. Variable amounts of thiophene and pyrid-2-ones are observed with alkynes bearing electron-withdrawing groups.^9^ These and subsequent observations indicate that steric, rather than electronic effects, are controlling elements during cycloadduct fragmentation.^2b,2e,6a^ A nitrile modification at C-2 leads preferentially to pyrid-2-ones, while a thioalkyl group at the same position switches the dipolar cycloaddition to thiophenes.^10^ An *N*,*N*-dialkylamino substituent at C-2 enhances the reactivity further, and mild room-temperature cycloadditions afford pyrid-2-one derivatives, although thiophene formation occurs as well, depending on the aryl substituent at C-3.^11^ Unlike monocyclic thioisomünchnones, bicyclic and ring-fused thioisomünchnones usually favor the formation of six-membered rings, presumably by alleviating the steric hindrance in cycloadducts by sulfur extrusion,^2b,12^ whereas intramolecular reactions with propargylic groups afford thiophene and thiazole derivatives.^13^ Also, pioneering work on the reactions of bridged bis- and tris(thiazolium-4-olates) evidenced the formation of pyridone derivatives after sulfur elimination.^14^ When collectively considered, such facts still point to a prevalent steric control, although the substitution pattern at the parent mesoionic ring modulates the fragmentation to some extent. Alternative studies from our group, involving both mono- and bicyclic thioisomünchnones with double and triple bonds unveil actually stereoelectronic effects, with cycloadduct fragmentation being determined by donor–acceptor interactions at the saddle-point structures.^15−17^ The most salient aspects of such computational insights are the interplay between concerted and stepwise routes. The latter appears to be dominant for isocyanate elimination. The concerted fragmentation would be disfavored owing to intermolecular steric repulsions during the dipole–dipolarophile interaction. Furthermore, cycloadditions with acetylenes resulting in sulfur elimination may also proceed via an initial sigmatropic shift, at least for conformationally restricted thioisomünchnones.^17^

**Figure 2:**
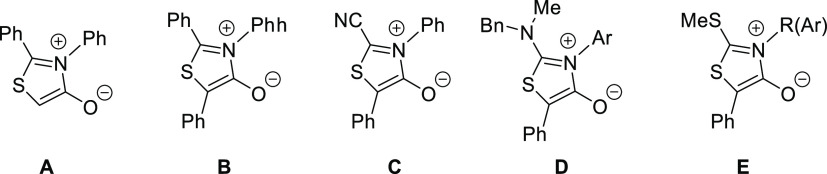
Representative structural variations in monocyclic thioisomünchnones employed in cycloaddition reactions with alkynes.

Given the somewhat capricious behavior of monocyclic thioisomünchnones (Figure 2), otherwise readily accessible in a few steps, we reasoned that regioselection might be fine-tuned by merging a small thiomethyl group at C-2 (Figure 2, structure E) as an exocyclic sulfur atom appears to have a stabilizing effect toward sulfur removal, with N-alkyl/aryl substitution at C-3 which would also modify the stereoelectronic interactions in the transient cycloadduct. Experimental and theoretical analyses detailed herein justify sufficiently this surmise.

## Results and Discussion

### Dipole Synthesis and Microwave-Assisted Cycloadditions

**Scheme 1:**
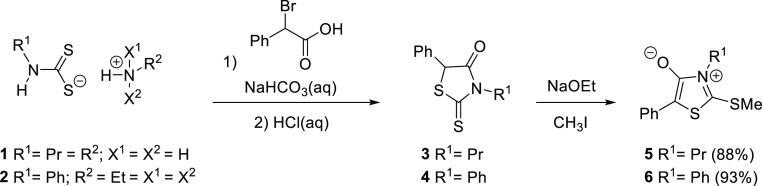
Sequential Cyclization and Mesoionization Leading to 2-Methylthiothiazol-3-ium-4-olate Derivatives

The preparation of 2-methylthio-5-phenyl-3-propylthiazol-3-ium-4-olate (**5**) and 2-methylthio-3,5-diphenylthiazol-3-ium-4-olate (**6**) was performed according to the procedure developed by Sandström et al. involving the reaction of 2-bromophenyl acetic acid (or its ethyl ester) with an alkylammonium *N*-alkyl(aryl)dithiocarbamate,^18^ which can easily be generated from the corresponding alkyl(aryl) amine and CS_2_ (Scheme 1). Salt formation along with subsequent heterocyclization and mesoionization can be conducted in aqueous and alcoholic conditions, more convenient than alternative protocols using volatile organics (C_6_H_6_/Et_3_N and CH_2_Cl_2_).^10^ The thiomethylated mesoionics were easily obtained by methylation (MeI) in basic medium of the corresponding thiazolidine-2-thione precursors **3** and **4**. Compounds **5** and **6** were isolated in high yields as air-stable, water-insoluble yellowish or orange solids. They were essentially pure after spontaneous crystallization, although **5** is often contaminated with NaI, which rendered it more stable than the naked mesoionic. This could be ascribed to a potential halogen interaction,^19^ although at this stage, we have no evidence supporting the classical Kosower-type π-interaction^20^ or charge transfer complexes, characteristic of mesoionic carbenes.^21^

After recrystallization, a low-melting heterocycle resulted for which no satisfactory microanalysis could be obtained, nevertheless. The molecular structures of such mesoionics were characterized by Fourier transform infrared (FTIR) and nuclear magnetic resonance (NMR) spectroscopy, and elemental analyses (see Experimental Section). In general, mesoionic rings are temperature-sensitive substances, and **5** and **6** are not exception to this rule. Cycloaddition reactions with acetylenic dipolarophiles, however, were sluggish at ambient temperature, and enhanced reactions were performed in refluxing toluene (110 °C) until the disappearance of the starting mesoionics [thin-layer chromatography (TLC) analysis]. As expected, this caused a gradual decomposition that forced chromatographic separation and resulted in the modest yields of pure products. While such cycloadditions required *ca*. 2 h for completion under conventional heating (with the exception of **6** with ethyl phenylpropiolate, taking longer), the same transformations conducted under dielectric heating using a professional microwave (MW) oven were essentially completed in less than 20 min at 100 °C.^22^ Optimized conditions involved an initial 1 min ramping heating at 500 W until reaching 100 °C and then at constant temperature for a given time.

### Regioselective Cycloaddition of Thioisomünchnone and Activated Acetylene Compounds

#### Structural Features

**Scheme 2:**
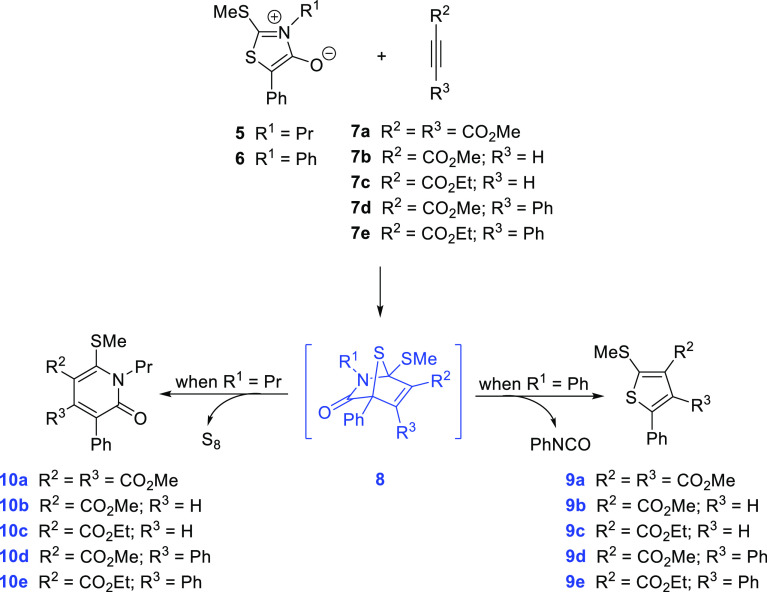
Divergent Fragmentation of Thioisomünchnone-alkyne Cycloadducts Producing Pyrid-2-ones or Thiophenes

From a mechanistic standpoint, the first step is the [3 + 2] cycloaddition of both partners, yielding an initial 1:1 cycloadduct (**8a–e**, not isolated). A subsequent retrocycloaddition removing phenyl isocyanate (PhNCO) gives rise to thiophene derivatives **9a–e**, whereas sulfur extrusion that also alleviates steric congestion leads to the corresponding 2-pyridones **10a–e** (Scheme 2). Inspection of crude mixtures indicates that only one heterocycle stems from each mesoionic ring. The presence of proton resonances at upper field, attributable to the propyl group, evidences the formation of pyridones and rules out a competing channel furnishing thiophenes. In stark contrast, the integration of downfield signals between 7.0 and 8.0 ppm for compounds **9a–e** is consistent with the liberation of PhNCO.

**Scheme 3:**
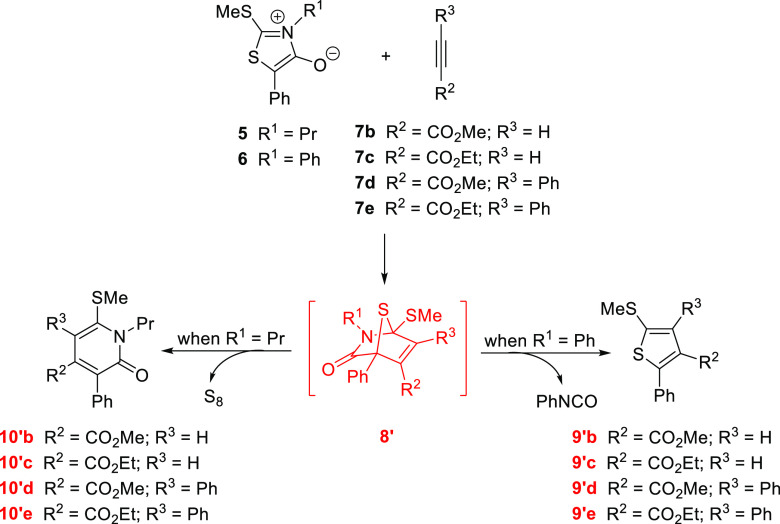
Alternative Regiochemistry for the Tandem Cycloaddition-Fragmentation of Thioisomünchnones 5 and 6 and Activated Acetylenes

However, with the exception of dimethyl acetylenedicarboxylate (**7a**), the remaining non-symmetrically substituted alkynes would approach to the mesoionic ring through two different orientations. It is obvious that similar fragmentation routes mirroring those in Scheme 2 could be postulated to produce either pyridones or thiophenes with the alternative regiochemistry, arising from the other cycloadduct intermediates (Scheme 3). Thus, as taken for such diagrams, the reaction of, for instance, **6** with methyl phenylpropiolate (**7d**) could generate the corresponding transient cycloadducts **8** and **8′**. The removal of PhNCO from such species gives rise to regioisomeric thiophenes **9d** and **9′d**, respectively.

The set of all-carbon quaternary atoms impedes an immediate discrimination between such substitution patterns. By comparing the phenyl protons in thiophene **9b** with those of **9a** (Figures S11 and S9, respectively), the former exhibits a broader chemical shift distribution (7.54–7.26 ppm) than the latter (7.47–7.36 ppm), which suggests that the methoxycarbonyl group of **9b** is not vicinal to the aromatic ring. This hypothesis can also be corroborated by recording heteronuclear multiple bond connectivity (HMBC) and heteronuclear multiple quantum coherence NMR spectra for the reaction product arising from **6** and **7e** (Figures S43 and S44, respectively). In the HMBC spectrum, the upfield signal at 2.61 ppm (attributable to the SCH_3_ protons) correlates well with a peak resonating at 147.8 ppm. This quaternary carbon could be attached to either an ester group (like in regioisomer **9e**) or an aromatic ring, like its counterpart **9′e**.

**Figure 3:**
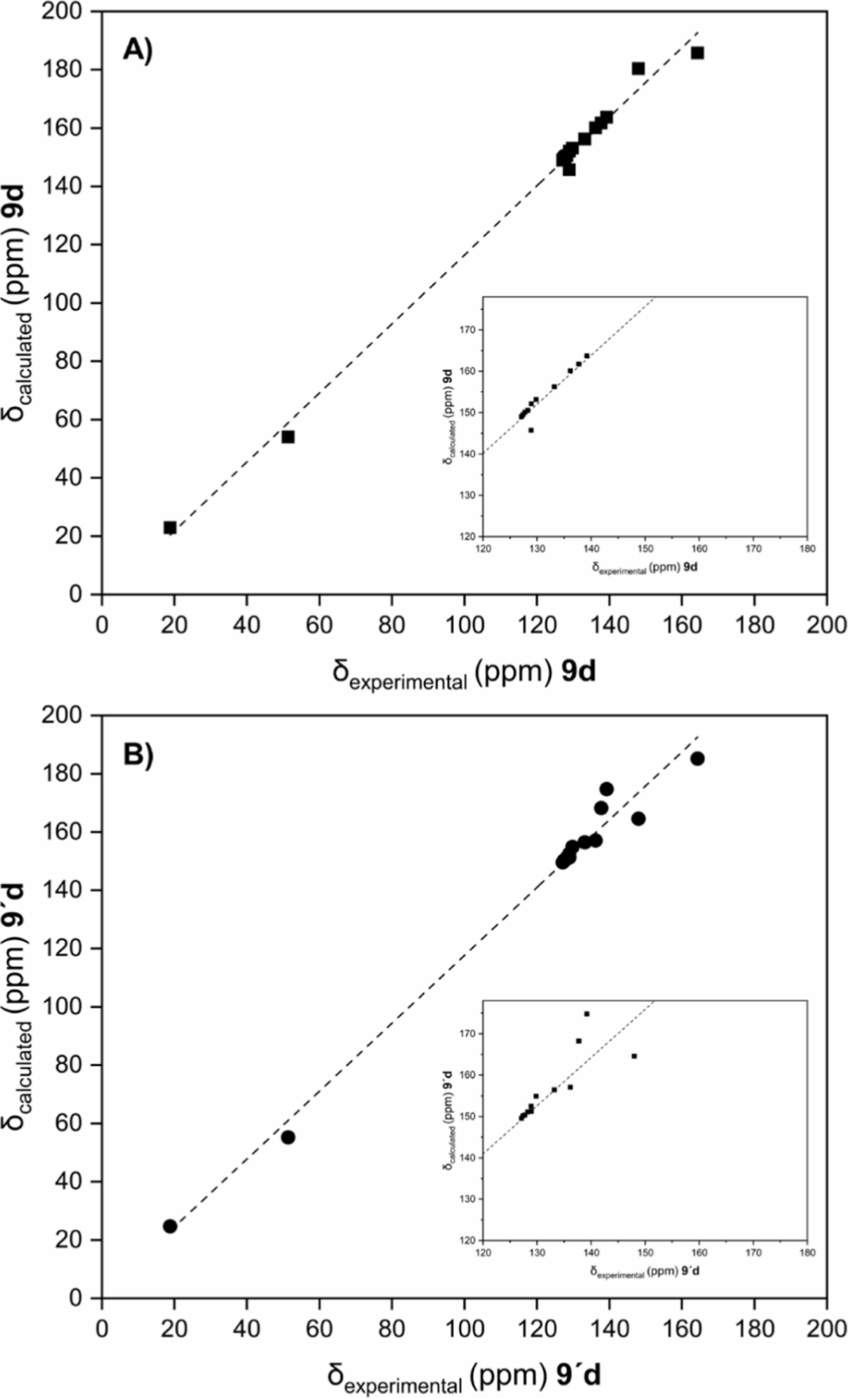
Plots obtained for correlations between GIAO-computed ^13^C-shifts of either (A) **9d** (black squares) or (B) **9′d** (black circles) structures and experimental carbon chemical shifts of the reaction product (from **6** and **7d**).

For similar thiophenes (**9d** and **9′d**), ^13^C chemical shifts were simulated by computing the magnetic shielding tensors with the GIAO-SCF method^23^ and then plotted against the experimental signals of the reaction product (Figure 3). A better linear relationship was obtained for **9d** than for the alternative regioisomer **9′d**, thereby pointing to the structural arrangement shown by compounds **9b–e**, where the COOMe or COOEt groups are spatially contiguous to the thiomethyl function.

**Figure 4:**
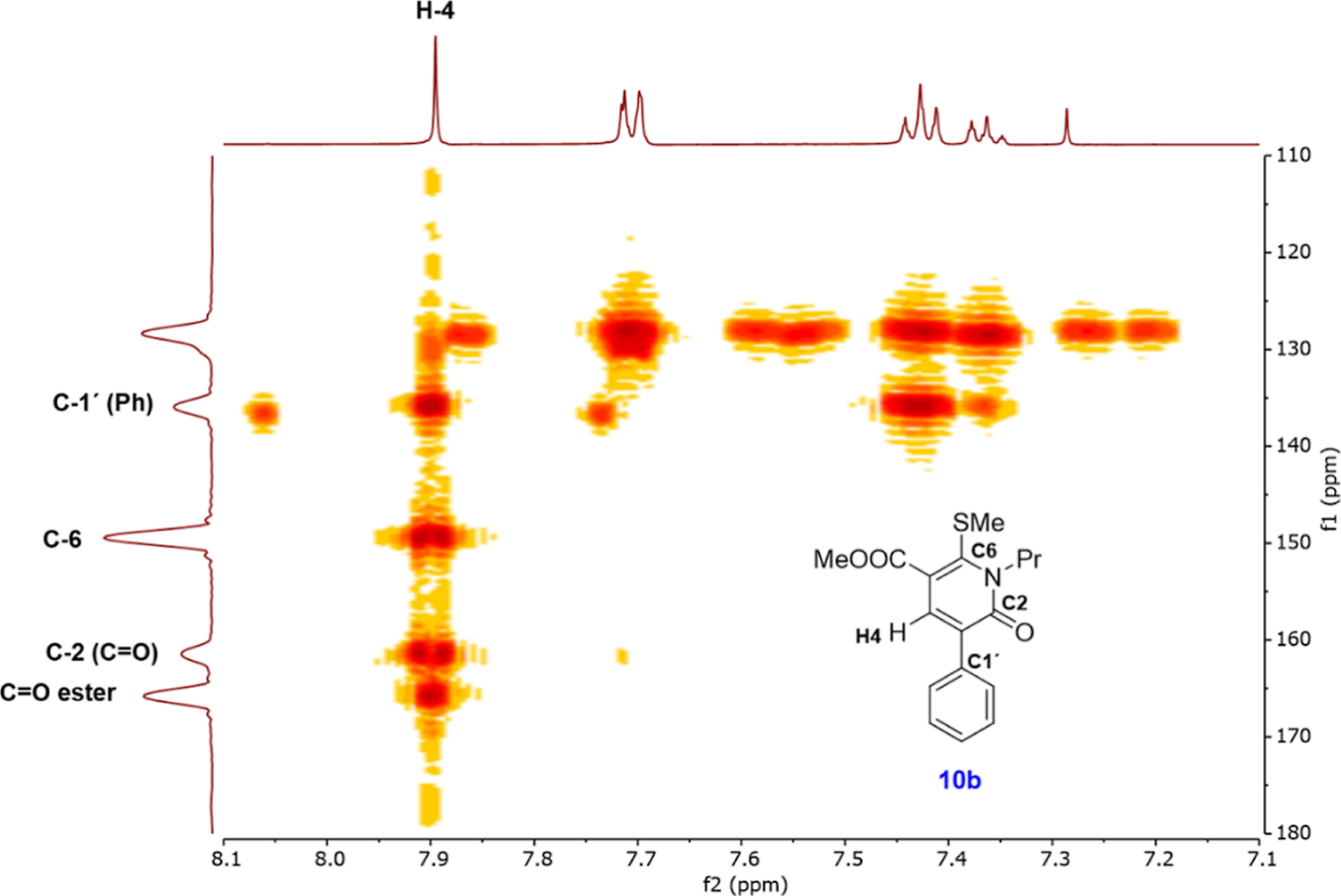
Heteronuclear HMBC spectrum for regioisomeric pyridone **10b** highlighting the diagnostic correlations with the H-4 proton.

**Figure 5:**
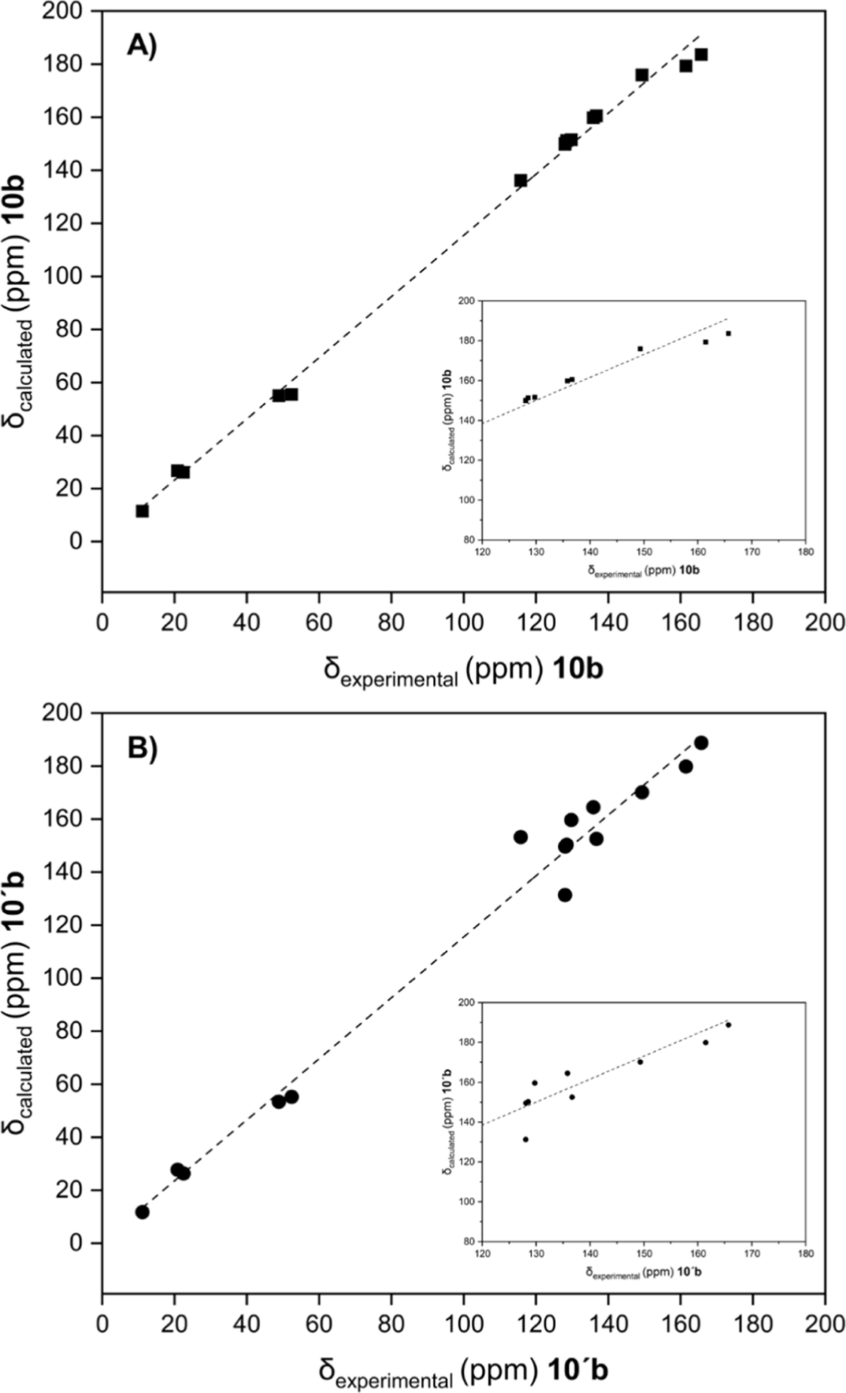
Linear correlations between GIAO-computed ^13^C chemical shifts of (A) **10b** (black squares) or (B) **10′b** (black circles) structures and experimental resonances for the reaction product derived from **5** and **7b**.

Likewise, the reaction channel involving the [3 + 2] cycloaddition of **5** with dipolarophiles **7a–e** proceeds through the selective sulfur removal from the corresponding cycloadducts, for which two regioisomeric approaches can be envisaged. In line with thiophenes, the HMBC spectrum recorded for the reaction product derived from **5** and methyl propiolate (**7b**) evidences that the singlet signal at 7.88 ppm shows four three-bond correlations with both carbonyl carbon atoms (C-2 and COOMe), C-6, and C-1′ of the phenyl group (Figure 4). Accordingly, the resulting pyrid-2-one moiety should be consistent with the 5-carboxylate regioisomer (**10b**). The hypothetical regioisomer (**10′b**), for which the ring proton signal would be located at C-5, should show only two three-bond correlations to C-3 and the carbonyl carbon of the COOMe group. To verify this assumption, the GIAO simulation of carbon shifts for **10b** and **10′b** plotted against the experimental ^13^C resonances leads to linear relationships, the former exhibiting the best fit (Figure 5).

### Reactivity and Regioselectivity

#### DFT Analyses

The tunable regioselection observed for these thioalkyl-substituted mesoionics can be assessed and largely understood through a rationale within the classical FMO framework. To this end, a DFT analysis was undertaken using the well-established and robust hybrid functional M06-2X at the 6-311G++(d,p) level, with inclusion of bulk solvation in toluene to simulate thermal reactions conducted in this solvent (see experimental details and Supporting Information). A preliminary inspection of both coefficients and OM energies reveals that in all cases, the regiochemical fate is dictated by a dominating highest occupied molecular orbital (HOMO)(dipole)–lowest unoccupied molecular orbital (LUMO)(dipolarophile) interaction, with a lower energy than the opposite orbitals (Figure S45), which is typical of type-I 1,3-dipolar cycloadditions.^24^

**Figure 6:**
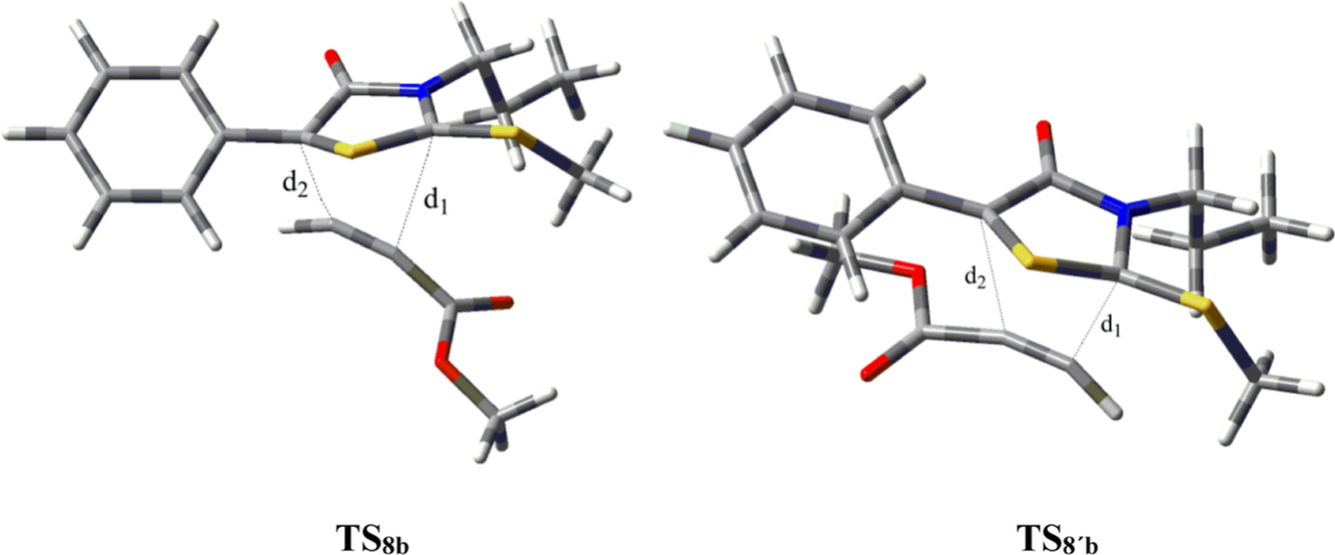
Optimized geometries of **TS**_**8b**_ (higher energy) and **TS**_**8’b**_ at the M06-2X/6-311++G(d,p) level in toluene.

**Scheme 4:**
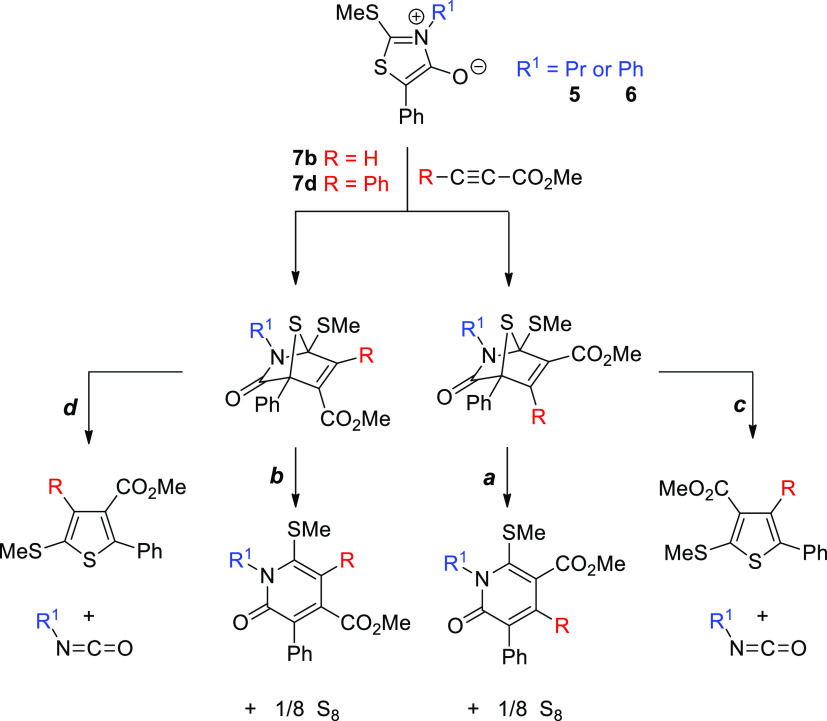
Potentially Competitive Routes for the Dipolar Cycloaddition of Mesoionics **5** and **6** with Methyl Propiolates **7b** and **7d**

Scheme 4 shows the regiodivergent pathways (**a–b**), from which the combination of **5** with methyl propiolate (or methyl phenyl propiolate) would evolve into a full set of pyrid-2-one derivatives through the intermediacy of two transient cycloadducts. Table 1 summarizes the energy differences (electronic, enthalpies, and free energies) among cycloadducts and transition structures, together with the corresponding imaginary frequencies and bond lengths, for the cycloadditive routes involving methyl propiolate (**7b**). Figure 6 also displays the optimized saddle points leading to cycloadducts **8b** and **8′b**. Formation of such N-propyl-based cycloadducts takes place through concerted mechanisms, although **TS**_**8’b**_ exhibits marked asynchronicity, as witnessed by the bond-forming distances (*d*_1_ = 2.14 Å, *d*_2_ = 2.44 Å). Moreover, Δ*G* values indicate that pathway **a** becomes the most favorable approach, thus accounting for the formation of cycloadduct **8b** and its conversion into pyridone **10b** as experimentally isolated.

**Table 1:** Energy Data and Geometric Parameters for the Cycloaddition of **5** and **7b**

structure	Δ*E* (kcal/mol)	Δ*H* (kcal/mol)	Δ*G* (kcal/mol)	frequency (cm^–1^)	*d*_1_ (Å)	*d*_2_ (Å)
5 + 7b	0	0	0			
**TS**_**8b**_	12.10	12.28	26.66	–443.26	2.373	2.123
**8b**	–38.65	–36.39	–20.95		1.554	1.526
**TS**8’_**b**_	14.63	14.35	29.03	–453.78	2.146	2.441
8′**b**	–39.77	–37.46	–22.61		1.530	1.545

An NBO (natural bond order)^25^ analysis is often useful to estimate the stabilization energy associated with electron delocalization between donor and acceptor orbitals, not only in the ground-state structure but also in transition structures. Table 2 lists the main donor–acceptor orbital interactions and their second-order perturbation stabilization energy which have been calculated for the mesoionic fragment (dipole end points, C2 and C3) and the triple bond (numbered C4 and C5 for clarity), leading to the productive cycloaddition. The stabilization is primarily caused by LP_C2_/π*(3)_C4–C5_, LP_C3_*/π*(3)_C4–C5_, π(3)_C4–C5_/LP_C2_, and π(3)_C4–C5_/LP_C3_* interactions. Although the charge transfers from dipole to dipolarophile are greater in the saddle points of stepwise processes than those of concerted pathways, as reported for structurally rigid mesoionics,^17b^ the prevalent interaction herein occurs in **TS**_**8b**_ and involves the LP_C2_/π*(3)_C4–C5_ interaction (59.75 kcal/mol), coincidental with the approach of the acetylenic hydrogen to the dipole C2 atom.

**Table 2:** NBO Analysis for Transition Structures Involving the Cycloaddition of **5** and **7b** (Stabilizing Energies Given in kcal/mol)

**TS8’**_**b**_		acceptor orbitals		
		π* (3)_C4–_C5	LP_C2_	LP_C3_*
donor orbitals	LP_C2_	19.26		
		38.08		
	LP_C3_*			
	π (3)_C4–C5_		22.43	
	π (3)_C4–C5_			86.09

**TS**_**8b**_		acceptor orbitals		
		π* (3)_C4–C5_	LP_C2_	LP_C3_*
donor orbital	LP_C2_	59.75		
		15.79		
	LP_C3_*			
	π (3)_C4–C5_		47.82	
	π (3)_C4–C5_			53.73

**Figure 7:**
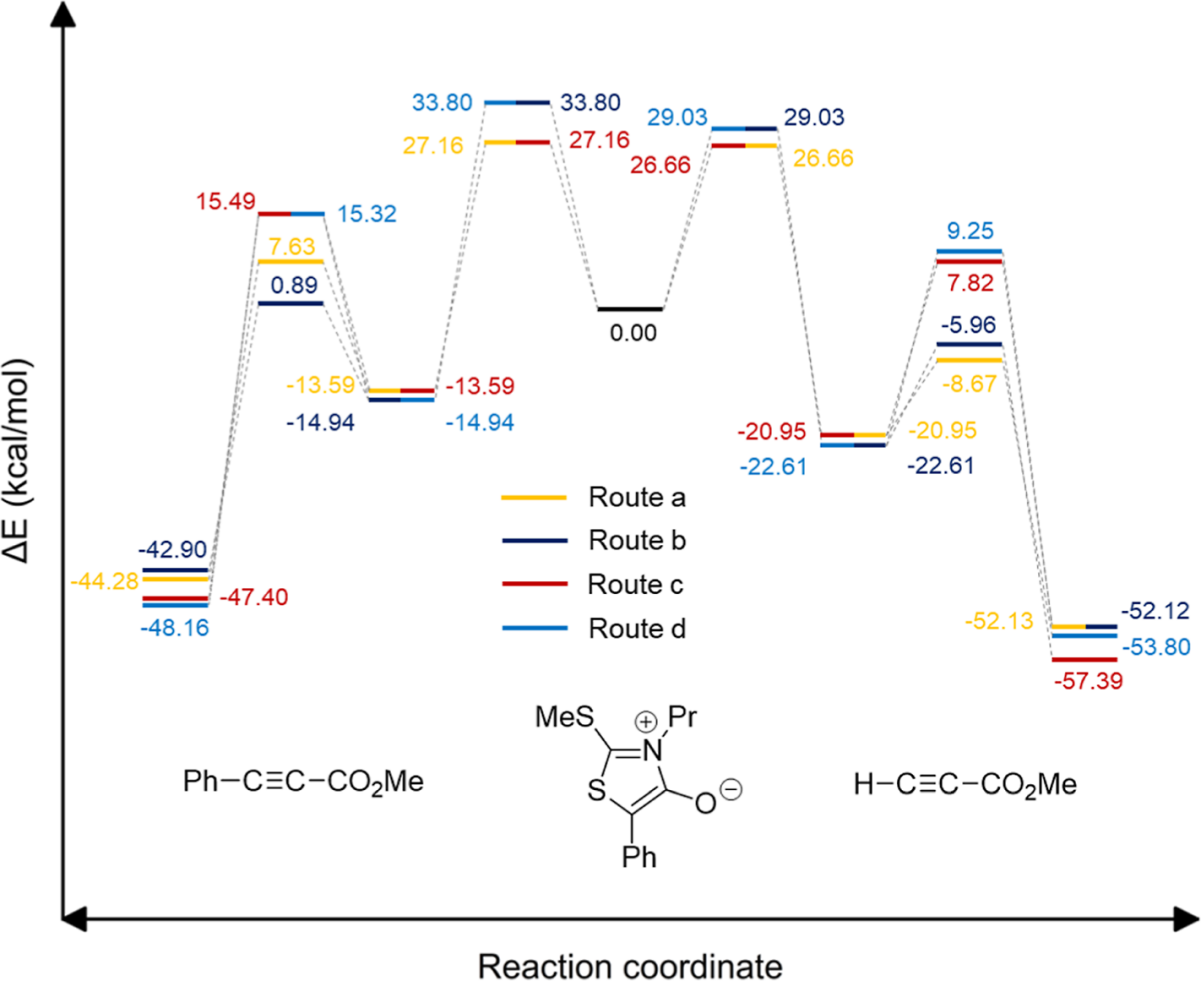
Energy profiles computed for the cycloadditions of heterocycle **5** with methyl propiolate (**7b**) and methyl phenyl propiolate (**7d**), producing either pyrid-2-ones (routes **a/b**) or thiophenes (routes **c/d**) with identification of all stationary points relative to the two reacting partners.

Even though thiophenes could not be detected from mesoionic **5**, we computed the four reaction routes stemming from ring-opening of the two regioisomeric cycloadducts (Scheme 4). For comparative purposes between the pathways leading to pyridones and thiophenes, Figure 7 depicts the entire energy landscapes for the reaction of **5** with both methyl propiolate and methyl phenyl propiolate. Close profiles are obtained regardless of the dipolarophile with routes **a** and **c**, showing the lowest energy gaps. However, sulfur extrusion and its conversion into pyridones is *ca*. 16.5 and 7.9 kcal/mol more favorable than isocyanate elimination, producing the corresponding thiophene in the case of the cycloaddition of heterocycle **5** with methyl propiolate (**7b**) and methyl phenyl propiolate (**7d**), respectively. This is clearly consistent with the experimental observation of the six-membered ring as the most stable reaction channel.

A similar analysis can be elaborated for the competitive formation of cycloadducts **8** and **8′** bearing an endocyclic N-phenyl bond (Schemes 2 or 3), generated from mesoionic 6, and now ending up in the chemoselective formation of thiophenes by loss of phenyl isocyanate. Such regiochemical pathways are illustrated in Scheme 4 for the two asymmetrically-substituted dipolarophiles methyl propiolate and methyl phenyl propiolate, leading to the corresponding thiophenes or their regioisomers. The energy data and imaginary frequencies capturing the identification of the corresponding saddle points are collected in Table 3. However, unlike the aforementioned calculation for pyridone formation, it is noteworthy that formation of cycloadduct 8b is a stepwise process occurring through the intermediacy of a dipolar species (**Int**_**8b**_), where both the positive charge developed at the C-2 position of the heterocycle and the negative charge from the dipolarophile are delocalized. On the contrary, the alternative path yielding cycloadduct **8′b** appears to be a concerted, yet asynchronous, reaction consistent with two disparate bond-forming distances (*d*_1_ = 2.13 Å and *d*_2_ = 2.49 Å) in **TS**_**8’b**_.

**Table 3:** Energy Data and Geometric Parameters for Species Involved in the Cycloaddition of **6** and **7b**

structure	Δ*E* (kcal/mol)	Δ*H* (kcal/mol)	Δ*G* (kcal/mol)	frequency (cm^–1^)	*d*_1_ (Å)	*d*_2_ (Å)
6 + 7b	0	0	0			
**TS1**_**8b**_	10.57	11.00	25.65	–397.82	3.143	1.919
**Int**_**8b**_	6.70	8.14	22.94		3.367	1.590
**TS2**_**8b**_	8.29	8.94	25.00	–175.95	2.792	1.608
**8b**	–43.70	–40.46	–23.85		1.542	1.542
**TS**8’_**b**_	12.37	12.58	27.72	–466.36	2.126	2.493
8′**b**	–44.60	–41.58	–25.33		1.532	1.540

**Figure 8:**
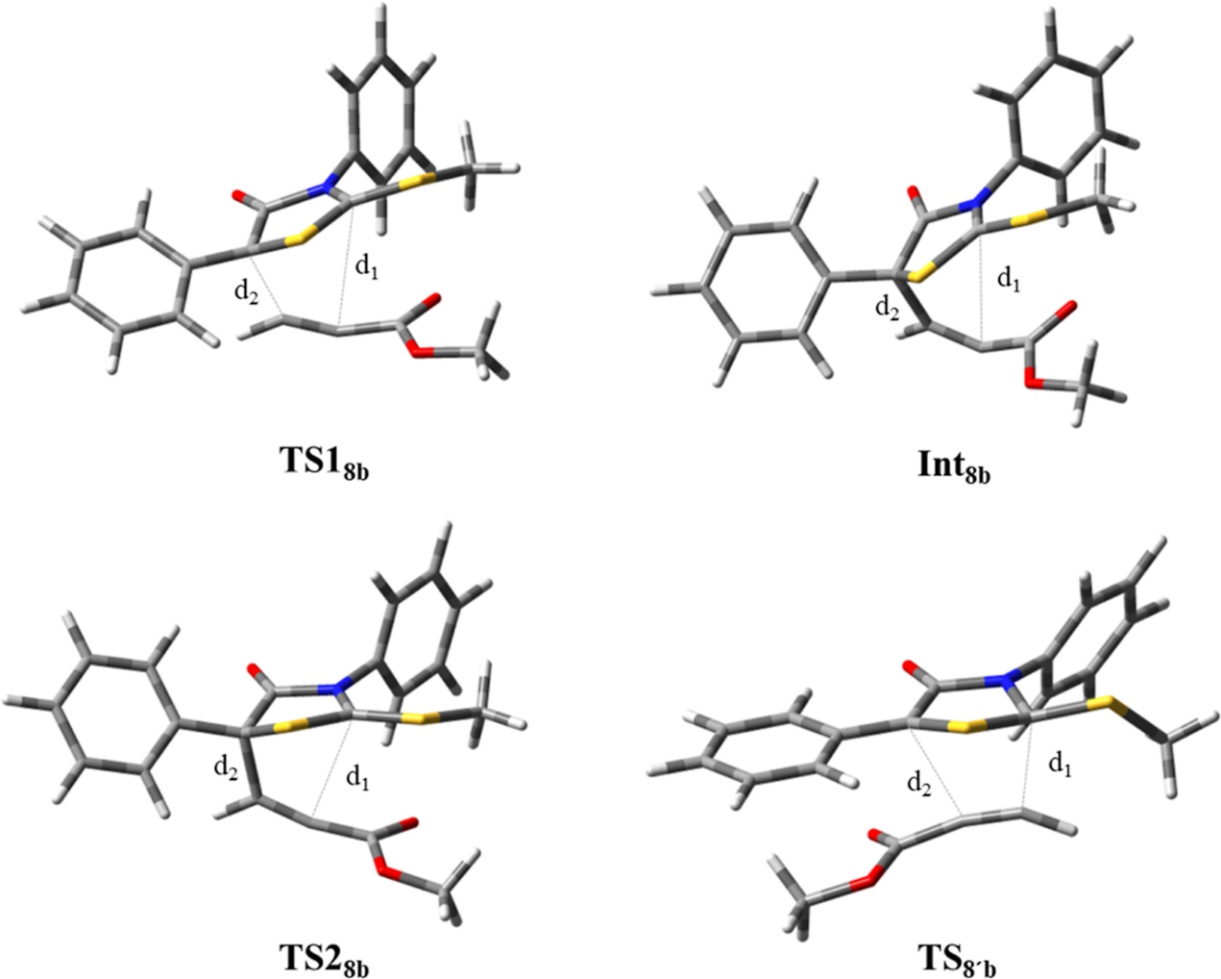
Optimized geometries of **TS1**_**8b**_, **Int**_**8b**_, **TS2**_**8b**_, and **TS**_**8’b**_ at the M06-2X/6-311++G(d,p) level in toluene.

**Figure 9:**
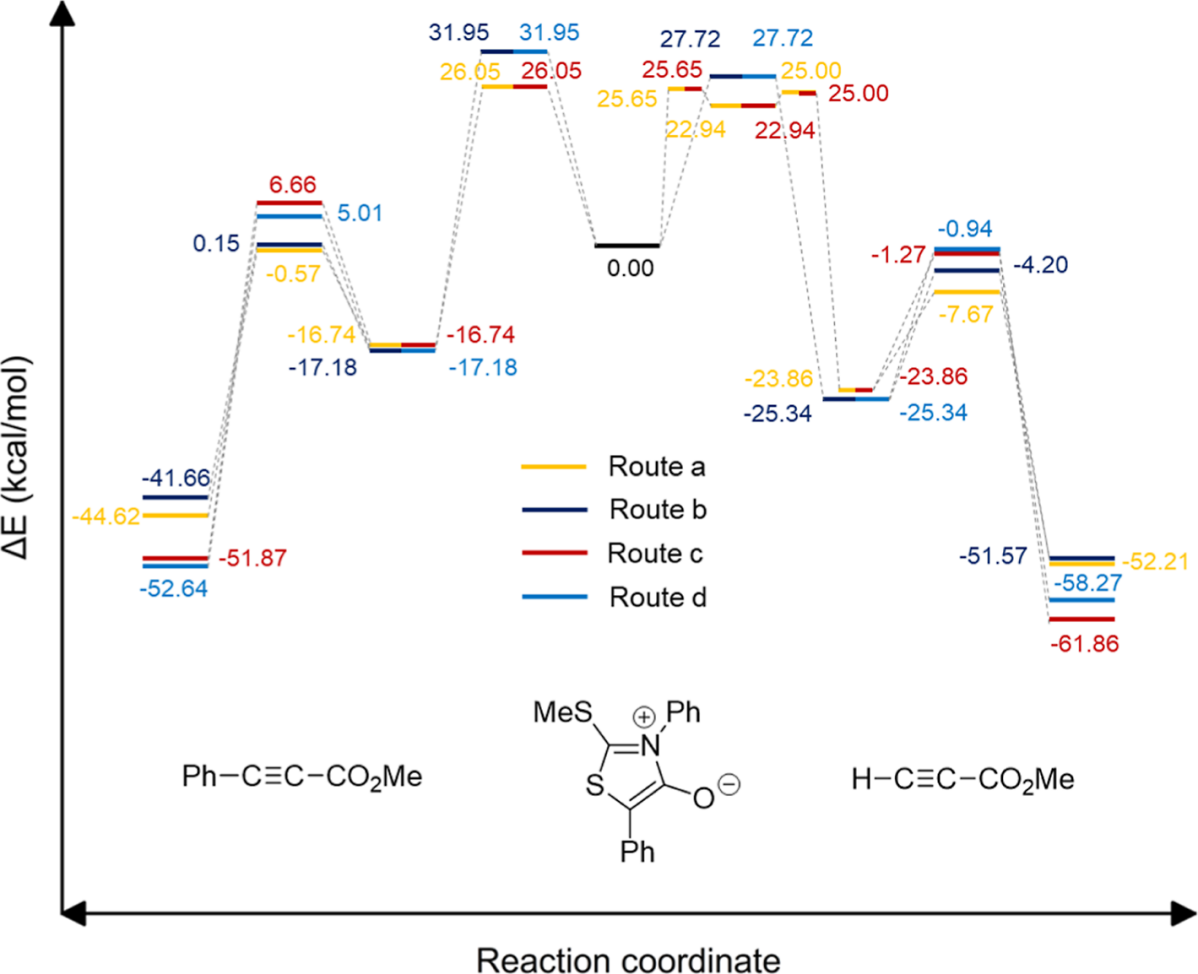
Energy profiles computed for the cycloadditions of heterocycle **6** with methyl propiolate (**7b**) and methyl phenyl propiolate (**7d**) with identification of all stationary points relative to the two reacting partners. Pyrid-2-one derivatives (not observed in experiments) arise from routes **a/b,** whereas thiophenes emerge from routes **c/d**; only **9b** and **9d** through path **c** were isolated.

Δ*G* values evidence again the favorable approach of pathway **c** producing selectively the formation of cycloadduct **8b** and its evolution into thiophene **9b**, thereby agreeing with experiments. Ball-and-stick models of such stationary points are shown in Figure 8. Like in the case of mesoionic **5**, all the energy profiles for the cycloaddition of **6** with methyl propiolate (**7b**) and methyl phenyl propiolate (**7d**) were computed at the same level of theory, as shown in Figure 9. Such graphs reflect the energy gaps among the four routes, where fragmentation of the regioisomeric cycloadducts would afford two sets of pyrid-2-one and thiophene derivatives, the former not experimentally observed. In line with Figure 7 above, pathways **a** and **c** are globally favored, thus explaining the selective formation of cycloadduct **8**. However, in this case, the energy difference between sulfur extrusion and isocyanate elimination is lower than that of mesoinic **5**.

These computational results adhere, in general, to the mechanistic trends observed for the spontaneous loss of sulfur or isocyanate in cycloadducts derived from thiazolium-4-olate systems. The liberation of isocyanate most likely involves a stepwise route,^11^ while sulfur extrusion has long been held to proceed through a concerted retro-cheletropic mechanism in thioisomünchnones and other sulfur-containing mesoionics. A recent analysis points to an unexpected detour involving a sequential 1,3-sigmatropic rearrangement of sulfur, followed by thiirane fragmentation, globally ending up in pyridones.^17a^ Likewise, the above analysis clearly indicates that the rate-limiting step is invariably the incipient formation of cycloadducts, irrespective of either pyridone or thiophene formation. The six-membered ring is kinetically favored with respect to the intramolecular retro-cycloaddition of isocyanate, furnishing thiophenes. The extreme regioselection observed for cycloadditions of **5** or **6** toward acetylenes should reasonably be ascribed to a higher inertness of such thiomethylated dipoles at C-2, evolving more slowly to a given cycloadduct than other reactive thioisomünchnones. The point has been checked by computing the four reaction channels of 2-dimethylamino-5-phenyl-3-propylthiazol-3-ium-4-olate (**11**, see Supporting Information) against methyl propiolate and methyl phenyl propiolate (Figure S46). As mentioned in the introductory remarks, such dialkylamino-substituted thioisomünchnones (Figure 2, structure **D**) are reactive enough and give rise to multiple cycloadduct fragmentations.^2b^Table S1 highlights the comparative assessment of the three mesoionic dipoles for the limiting step, leading to the kinetically-favored 2-pyridone ring (route **a**), with a lower energy barrier for cycloadduct formation starting from mesoionic **11**.

## Conclusions

Summing up, the present study combining the theory and experiment discloses structural and mechanistic aspects that unveil the controlled selection of 2-thiomethyl-substituted thiazolium-4-olate dipoles toward acetylenic dipolarophiles. While such cycloadditions have long been known in mesoionic chemistry, this reinvestigation provides new vistas in both chemoselection and regioselection. Spectroscopic correlations and linear analysis were employed to achieve unambiguous structural determination, given the existence of multiple quaternary centers in reaction products. As inferred from the theoretical analysis, the mild “nucleophilicity” of 2-thioalkyl-derived dipoles favors the selective formation of a given cycloadduct through slower thermal reactions, relative to other high-lying HOMO-controlled cycloadditions. Finally, this work enables new prospects and opportunities to invigorate the cycloaddition chemistry of mesoionic heterocycles.

## Experimental Section

### General Information

Unless otherwise stated, all solvents and chemicals (including compounds **7a–7e**) were purchased from commercial suppliers and used without further purification. Solvents were evaporated below 50 °C at estimated pressures between 15 and 30 mm Hg. Melting points were measured on the Electrothermal 9100 apparatus in capillary tubes and are uncorrected. Elemental analyses for C, H, N, and S were performed using the Leco CHNS-932 analyzer. FTIR spectra were recorded on the Thermo IR-300 spectrophotometer (4000–600 cm^–1^ range) using KBr pellets. All reactions were monitored by TLC on Aldrich Polygram Sil G/UV254 plates (7 × 3 cm) using benzene/acetonitrile (5:1, v/v) as the eluent. Flash chromatography purification was carried out on Merck silica gel 60 (400–230 mesh). The corresponding eluents are specified in every case. ^1^H NMR (500, 400 MHz) and ^13^C NMR (125, 100 MHz) spectra were obtained using Bruker AVANCE spectrometers, which were recorded at rt in CDCl_3_ and DMSO-*d*_6_. Chemical shifts are reported in parts per million (δ), downfield from tetramethylsilane (Me_4_Si, TMS) as the internal reference. Coupling constants (*J* values) are given in Hz, and standard abbreviations were used to indicate spin multiplicities, namely s = singlet, d = doublet, t = triplet, q = quartet, dd = doublet of doublet, m = multiplet, and bs = broad singlet. Carbon chemical shifts in the ^13^C{^1^H} NMR spectra are reported relative to CHCl_3_ (δ_C_ 77.00 ppm, central line of triplet). The nature of the carbons (C, CH, CH_2_, and CH_3_) was determined by recording the DEPT spectra. The latter, together with 2D NMR spectra, enabled the assignment of signals. For MW-irradiated reactions, the reaction mixture was heated in an open vessel (maximum power: 500 W) with a multimode apparatus from Milestone (Advance Microwave LabStation, Ethos touch control model), with the temperature ramping from room temperature to 100 °C within 1 min (using a fiber-optic probe to determine the temperature profile). The reaction was maintained at that temperature for 10 min and then cooled to room temperature. Computational data were obtained using the Gaussian09 program package.^26^ All structures were optimized by means of DFT^27^ by combining the M06-2X method^28^ and the 6-311++G(d,p) basis set.^29^ The nature of the stationary points was confirmed by frequency analysis at the above-mentioned level of theory at 298.15 K. Solvent effects were estimated using the solvation model density (SMD).^30^ In order to reproduce thermal reactions in toluene, the SMD calculations were performed in toluene at 373.15 K. Intrinsic reaction coordinate analysis validated that all the transition structures belonged to the reaction path. Simulation of NMR resonances was achieved by computing NMR shielding tensors with the gauge-independent atomic orbital-SCF method, at the M06-2X/6-311++G(2d,p), in chloroform. NBO analysis has been carried out with the NBO 3.1 package.^31^

#### Propylammonium Propylcarbamodithioate (**1**)

To a solution of CS_2_ (6 mL, 0.1 mol) in dry diethyl ether (25 mL), was added dropwise a solution (cooled at 0 °C) of propylamine (16.4 mL, 0.2 mol) in dry diethyl ether (50 mL). The mixture was stirred for 90 min, furnishing a white crystalline solid that was collected by filtration, washed with diethyl ether, dried under vacuum, and used in next steps without further purification.

#### Triethylammonium phenylcarbamodithioate (**2**)

To CS_2_ (6 mL, 99 mmol) and aniline (9 mL, 99 mmol) was added dropwise triethylamine (14 mL, 99 mmol), and the resulting mixture was stirred at 0 °C for 30 min. A yellowish crystalline solid was obtained, which was filtered, washed with diethyl ether, dried under vacuum, and used in next steps without further purification.

#### 5-Phenyl-3-propyl-2-thioxothiazolidin-4-one (**3**)

To a solution of α-bromophenyl acetic acid (4.06 g, 20 mmol) and NaHCO_3_ (1.93 g, 22 mmol) in water (20 mL), was added propylammonium *N*-propyldithiocarbamate (3.24 g, 20 mmol), and the mixture was stirred vigorously for 5 h. The resulting oily product was separated by decantation and treated with 5 M HCl solution until pH ≈ 1 and then refluxed for 15 min. The aqueous phase was separated, and the organic oil was washed with water (3 × 100 mL). The residue was treated with ethanol (30 mL) and heated at 50 °C, giving rise to a yellowish solid that was filtered and washed with cold ethanol (2.25 g, 57%) and had mp 60–61 °C (recrystallized from EtOH). IR (KBr): ν*®*_max_ 3022, 2961, 2934, 2874, 1734, 1352, 1285, 1211, 768, 717, 557, 527 cm^–1^. ^1^H NMR (500 MHz, CDCl_3_) δ: 7.42–7.32 (m, 5H), 5.24 (s, 1H), 4.00 (t, *J* = 7.5 Hz, 2H), 1.75–1.67 (m, 2H), 0.94 (t, *J* = 7.5 Hz, 3H) ppm. ^13^C{^1^H} NMR (125 MHz, CDCl_3_) δ: 200.1, 175.0, 133.9, 129.3, 129.1, 128.2, 54.3, 46.4, 20.3, 11.1 ppm.

#### 3,5-Diphenyl-2-thioxothiazolidin-4-one (**4**)

To a solution of α-bromophenyl acetic acid (3,9 g, 18 mmol) in water (20 mL), was added NaHCO_3_ (1,7 g, 20 mmol) and triethylammonium *N*-phenyldithiocarbamate (4,87 g, 18 mmol), and the resulting mixture was stirred vigorously for 24 h. An oily product was separated by decantation and treated with 5 M HCl solution until pH ∼1. The aqueous layer was decanted, and the remaining oil was washed with water (3 × 100 mL). The residue was treated with ethanol (30 mL) and warmed at 50 °C, giving rise to a solid material that was filtered and washed with cold ethanol. That crude product was further purified by column chromatography using benzene as the eluent. The yellowish solid obtained by evaporation and dried under vacuum (1.78 g, 46%) had mp 225–226 °C. IR (KBr): ν*®*_max_ 1729, 1255, 1226, 749 cm^–1^. ^1^H NMR (500 MHz, CDCl_3_) δ: 7.57–7.38 (m, 10H), 6.01 (s, 1H) ppm. ^13^C{^1^H} NMR (125 MHz, CDCl_3_) δ: 201.4, 174.9, 135.5, 134.8, 129.4, 129.3, 129.1, 128.8, 128.7, 54.8 ppm. Anal. Calcd for C_15_H_11_NOS_2_: C, 63.13; H, 3.89; N, 4.91; S, 22.47. Found: C, 63.69; H, 3.84; N, 4.67; S, 22.75.

#### 2-(Methylthio)-5-phenyl-3-propylthiazol-3-ium-4-olate (**5**)

To a solution of **3** (0.65 g, 2.59 mmol) in 1 M sodium ethoxide (3 mL) cooled at 0 °C, was added methy iodide (0.20 mL, 3.24 mmol), and the mixture was stirred for 30 min. After 24 h at 5–6 °C, the solution was evaporated under reduced pressure, giving rise to a yellow solid that was filtered and washed with cold ethanol. An essentially pure compound was obtained by crystallization from EtOH–H_2_O, followed by recrystallization from toluene, although no satisfactory microanalysis could be obtained (0.61 g, 88%); mp: 59–60 °C. IR (KBr): ν*®*_max_ 3407, 1600, 1578, 1495, 1120, 752 cm^–1^. ^1^H NMR (500 MHz, CDCl_3_) δ: 7,82 (d, *J* = 7.5 Hz, 2H), 7.31 (t, *J* = 8.0 Hz, 2H), 7.07 (t, *J* = 7.5 Hz, 1H), 4.07 (t, *J* = 7.5 Hz, 2H), 2,72 (s, 3H), 1.90–1.82 (m, 2H), 1.02 (t, *J* = 7.5 Hz, 3H) ppm. ^13^C{^1^H} NMR (125 MHz, CDCl_3_) δ: 159.4, 151.6, 133.7, 128.6, 124.3, 123.2, 95.8, 48.3, 21.3, 16.5, 11.3 ppm.

#### 2-(Methylthio)-3,5-diphenylthiazol-3-ium-4-olate (**6**)

To a solution of **4** (0.48 g, 1.6 mmol) in 1 M sodium ethoxide (1.8 mL) cooled at 0 °C, was added methyl iodide (0.12 mL, 1.9 mmol), and the mixture was stirred for 30 min, which gave rise to the spontaneous formation of an orange solid that was collected by filtration and washed with distilled water (0,47 g, 93%); mp: 161–162 °C. IR (KBr): ν*®*_max_ 3043, 1613, 1584, 1495, 1115 cm^–1^. ^1^H NMR (500 MHz, CDCl_3_) δ: 7.85–7.07 (m, 10H), 2.62 (s, 3H) ppm. ^13^C{^1^H} NMR (125 MHz, CDCl_3_) δ: 160.2 154.1, 135.8, 135.6, 130.2, 129.7, 128.6, 127.1, 124.3, 123.1, 94.9, 15.9 ppm. Anal. Calcd for C_16_H_13_NOS_2_: C, 64.18; H, 4.38; N, 4.68; S, 21.42. Found: C, 63.72; H, 4.32; N, 4.70; S, 20.84.

### Reactions of Thiazol-3-ium-4-olates with Acetylenic Compounds

#### General Procedure

A mixture containing the mesoionic dipole (**5** or **6**) (1.37 mmol, 1.00 equiv, 0.27 M) and the corresponding acetylenic dipolarophile **7a**–**e** (1.65 mmol, 1.20 equiv) in toluene (5 mL) was placed in a 10 mL pyrex flask and heated under MW irradiation (vide supra: general information) until the disappearance of the starting heterocycle (TLC analyses were conducted in benzene/acetonitrile, 5:1). The reaction mixture was then cooled to room temperature, the solvent was evaporated to dryness, and the resulting residue was purified by column chromatography with petroleum ether/diethyl ether (PE/DE = 1:1).

#### Dimethyl 2-(methylthio)-5-phenylthiophene-3,4-dicarboxylate (**9a**)

The title compound was isolated by silica gel flash column chromatography (PE/DE = 1:1), as a white crystalline solid (130 mg, 30% yield). Mp: 129–130 °C [lit.^10^ 130–131 °C]. IR (KBr): ν*®*_max_ 2957, 1727, 1698, 1447, 1226 cm^–1^. ^1^H NMR (500 MHz, CDCl_3_) δ: 7.47–7.36 (m, 5H), 3.87 (s, 3H), 3.80 (s, 3H), 2.61 (s, 3H) ppm. ^13^C{^1^H} NMR (125 MHz, CDCl_3_) δ: 165.9, 162.8, 150.6, 140.1, 131.9, 131.3, 128.8, 128.7, 128.1, 125.8, 52.6, 52.0, 18.6 ppm. Anal. Calcd for C_15_H_14_O_4_S_2_: C, 55.88; H, 4.38; S, 19.88. Found: C, 55.74; H, 4.31; S, 20.57.

#### Methyl 2-(methylthio)-5-phenylthiophene-3-carboxylate (**9b**)

The title compound was isolated by silica gel flash column chromatography (PE/DE = 1:1), as a white crystalline solid (90 mg, 25% yield). mp: 102–103 °C. IR (KBr): ν*®*_max_ 1698, 1452, 1243, 1054, 753 cm^–1^. ^1^H NMR (500 MHz, CDCl_3_) δ: 7.61 (s, 1H), 7.54–7.26 (m, 5H), 3.88 (s, 3H), 2.64 (s, 3H) ppm. ^13^C{^1^H} NMR (125 MHz, CDCl_3_) δ: 163.6, 151.1, 139.9, 133.3, 129.0, 127.7, 126.6, 125.2, 124.7, 51.6, 18.3 ppm. Anal. Calcd for C_13_H_12_O_2_S_2_: C, 59.06; H, 4.58; S, 24.26. Found: C, 58.97; H, 4.77; S, 25.41.

#### Ethyl 2-(methylthio)-5-phenylthiophene-3-carboxylate (**9c**)

The title compound was isolated by silica gel flash column chromatography (PE/DE = 1:1), as a white crystalline solid (110 mg, 34% yield). mp: 86–87 °C. IR (KBr): ν*®*_max_ 3104, 2992, 2979, 2949, 1692, 1448, 1434, 1375, 1241, 1054, 758, 688, 582, 464 cm^–1^. ^1^H NMR (500 MHz, CDCl_3_) δ: 7.61 (s, 1H), 7.54–7.53 (m, 2H), 7.39–7.36 (m, 2H), 7.29–7.26 (m, 1H), 4.35 (q, *J* = 7 Hz, *J* = 14.5 Hz, 2H), 2.63 (s, 3H), 1.39 (t, *J* = 7.5 Hz, 3H) ppm. ^13^C{^1^H} NMR (125 MHz, CDCl_3_) δ: 163.2, 150.8, 139.8, 133.3, 128.9, 127.6, 126.9, 125.2, 124.8, 60.6, 18.3, 14.4 ppm. Anal. Calcd for C_14_H_14_O_2_S_2_: C, 60.40; H, 5.07; S, 23.04. Found: C, 60.40; H, 5.19; S, 23.05.

#### Methyl 2-(methylthio)-4,5-diphenylthiophene-3-carboxylate (**9d**)

The title compound was isolated by silica gel flash column chromatography (PE/DE = 1:1), as a white crystalline solid (180 mg, 38% yield). mp: 127–128 °C. IR (KBr): ν*®*_max_ 2947, 1717, 1437, 1215, 696 cm^–1^. ^1^H NMR (500 MHz, CDCl_3_) δ: 7.61 (s, 1H), 7.54–7.26 (m, 5H), 3.88 (s, 3H), 2.64 (s, 3H) ppm. ^13^C{^1^H} NMR (125 MHz, CDCl_3_) δ: 163.6, 151.1, 139.9, 133.3, 129.0, 127.7, 126.6, 125.2, 124.7, 51.6, 18.3 ppm. Anal. Calcd for C_19_H_16_O_2_S_2_: C, 67.03; H, 4.74; S, 18.84. Found: C, 67.43; H, 4.74; S, 19.49.

#### Ethyl 2-(methylthio)-4,5-diphenylthiophene-3-carboxylate (**9e**)

The title compound was isolated by silica gel flash column chromatography (PE/DE = 1:1), as a white crystalline solid (110 mg, 27% yield). mp: 103–104 °C. IR (KBr): ν*®*_max_ 3050, 2973, 1705, 1208, 1138, 694 cm^–1^. ^1^H NMR (500 MHz, CDCl_3_) δ: 7.27–7.25 (m, 3H), 7.18–7.15 (m, 5H), 7.12–7.10 (m, 2H), 4.06 (q, *J* = 7.2 Hz, *J* = 14.4 Hz, 2H), 2.64 (s, 3H), 0.94 (t, *J* = 7.2 Hz, 3H) ppm. ^13^C{^1^H} NMR (125 MHz, CDCl_3_) δ: 163.9, 148.0, 139.2, 137.6, 136.5, 133.2, 129.2, 128.8, 128.3, 127.7, 127.4, 127.0, 60.4, 18.8, 13.6 ppm. Anal. Calcd for C_20_H_18_O_2_S_2_: C, 67.76; H, 5.12; S, 18.09. Found: C, 67.53; H, 5.04; S, 17.92.

#### Dimethyl 2-(methylthio)-6-oxo-5-phenyl-1-propyl-1,6-dihydropyridine-3,4-dicarboxylate (**10a**)

The title compound was isolated by silica gel flash column chromatography (PE/DE = 1:1), as a white crystalline solid (81 mg, 23% yield). mp: 103–104 °C. IR (KBr): ν*®*_max_ 3436, 2952, 2872, 1723, 1643, 1437, 1225, 742, 700 cm^–1^. ^1^H NMR (500 MHz, CDCl_3_) δ: 7.39–7.30 (m, 5H), 4.36 (t, *J* = 7.5 Hz, 1H), 4.36 (dd, *J* = 5.0 Hz, *J* = 10.0 Hz, 1H), 3.86 (s, 3H), 3.51 (s, 3H), 2.55 (s, 3H), 1.77 (m, *J* = 7.5 Hz, 2H), 0.99 (t, *J* = 7.5 Hz, 3H) ppm. ^13^C{^1^H} NMR (125 MHz, CDCl_3_) δ: 166.6, 166.2, 161.5, 144.2, 139.2, 134.1, 131.0, 129.1, 128.5, 128.0, 118.6, 52.8, 52.5, 49.2, 22.4, 21.0, 11.2 ppm. Anal. Calcd for C_19_H_21_NO_5_S: C, 60.78; H, 5.64; N, 3.73; S, 8.54. Found: C, 60.79; H, 5.61; N, 3.77; S, 8.12.

#### Methyl 2-(methylthio)-6-oxo-5-phenyl-1-propyl-1,6-dihydropyridine-3-carboxylate (**10b**)

The title compound was isolated by silica gel flash column chromatography (PE/DE = 1:1), as a white crystalline solid (55 mg, 18% yield). mp: 84–85 °C. IR (KBr): ν*®*_max_ 2955, 2921, 1723, 1649, 1229, 1075, 793, 700 cm^–1^. ^1^H NMR (500 MHz, CDCl_3_) δ: 7.88 (s, 1H), 7.69–7.68 (m, 2H), 7.42–7.39 (m, 2H), 7.35–7.33 (m, 1H), 4.47 (t, *J* = 8.0 Hz, 1H), 4.47 (dd, *J* = 5.5 Hz, *J* = 10.5 Hz, 1H), 3.90 (s, 3H), 2.57 (s, 3H), 1.78 (m, *J* = 7.5 Hz, 2H), 1.02 (t, *J* = 7.5 Hz, 3H) ppm. ^13^C{^1^H} NMR (125 MHz, CDCl_3_) δ: 175.7, 161.5, 149.3, 136.7, 135.8, 129.1, 129.8, 128.5, 128.1, 128.1, 115.8, 52.4, 48.9, 22.5, 20.8, 11.1 ppm. Anal. Calcd for C_17_H_19_NO_3_S: C, 64.33; H, 6.03; N, 4.41; S, 10.10. Found: C, 64.06; H, 5.73; N, 4.46; S, 9.78.

#### Ethyl 2-(Methylthio)-6-oxo-5-phenyl-1-propyl-1,6-dihydropyridine-3-carboxylate (**10c**)

The title compound was isolated by silica gel flash column chromatography (PE/DE = 1:1), as a white crystalline solid (89 mg, 28% yield). mp: 50–51 °C. IR (KBr): ν*®*_max_ 2976, 2958, 2928, 2877, 1726, 1642, 1230, 1168, 1079, 793, 697 cm^–1^. ^1^H NMR (500 MHz, CDCl_3_) δ: 7.85 (s, 1H), 7.69–7.67 (m, 1H), 7.42–7.39 (m, 2H), 7.35–7.33 (m, 1H), 4.47 (t, *J* = 8.0 Hz, 1H), 4.47 (dd, *J* = 5.5 Hz, *J* = 10.5 Hz, 1H), 4.37 (q, *J* = 7 Hz, 2H), 2.57 (s, 3H), 1.78 (m, *J* = 7.5 Hz, 2H), 1.39 (t, *J* = 7.5 Hz, 3H), 1.02 (t, *J* = 7.5 Hz, 3H) ppm. ^13^C{^1^H} NMR (125 MHz, CDCl_3_) δ: 165.5, 161.6, 148.8, 136.7, 135.9, 129.9, 128.6, 128.1, 128.1, 116.5, 61.5, 48.9, 22.5, 20.9, 14.2, 11.2 ppm. Anal. Calcd for C_18_H_21_NO_3_S: C, 65.23; H, 6.39; N, 4.23; S, 9.67. Found: C, 64.88; H, 6.36; N, 4.36; S, 9.36.

#### Methyl 2-(methylthio)-6-oxo-4,5-diphenyl-1-propyl-1,6-dihydropyridine-3-carboxylate (**10d**)

The title compound was isolated by silica gel flash column chromatography (PE/DE = 1:1), as a white crystalline solid (80 mg, 22% yield). mp: 98–99 °C. IR (KBr): ν*®*_max_ 3060, 3031, 2962, 2925, 2876, 1736, 1639, 1509, 1426, 1306, 1212, 1083, 697 cm^–1^. ^1^H NMR (500 MHz, CDCl_3_) δ: 7.16–7.02 (m, 10H), 4.38 (t, *J* = 7.6 Hz, 1H), 4.38 (dd, *J* = 5.2 Hz, *J* = 10.4 Hz, 1H), 3.48 (s, 3H), 2.55 (s, 3H), 1.88–1.80 (m, 2H), 1.03 (t, *J* = 7.6 Hz, 3H) ppm. ^13^C{^1^H} NMR (125 MHz, CDCl_3_) δ: 166.9, 161.9, 146.3, 139.6, 136.3, 134.6, 132.0, 130.5, 128.6, 127.6, 127.3, 126.9, 124.5, 52.1, 48.8, 22.4, 21.0, 11.2 ppm. Anal. Calcd for C_23_H_23_NO_3_S: C, 70.20; H, 5.89; N, 3.56; S, 8.15. Found: C, 69.76; H, 5.78; N, 3.53; S, 7.98.

#### Ethyl 2-(methylthio)-6-oxo-4,5-diphenyl-1-propyl-1,6-dihydropyridine-3-carboxylate (**10e**)

The title compound was isolated by silica gel flash column chromatography (PE/DE = 1:1), as a white crystalline solid (89 mg, 28% yield). mp: 128–129 °C. IR (KBr): ν*®*_max_ 3056, 2965, 2927, 1719, 1641, 1293, 1209, 695 cm^–1^. ^1^H NMR (500 MHz, CDCl_3_) δ: 7.16–7.14 (m, 4H), 7.13–7.10 (m, 2H), 7.07–7.05 (m, 4H), 4.38 (t, *J* = 8.0 Hz, 1H), 4.38 (dd, *J* = 5.5 Hz, *J* = 10.5 Hz, 1H), 3.94 (q, *J* = 7.5 Hz, *J* = 14.5 Hz, 2H), 2.56 (s, 3H), 1.88–1.81 (m, 2H), 1.03 (t, *J* = 7.5 Hz, 3H), 0.93 (t, *J* = 7.5 Hz, 3H) ppm. ^13^C{^1^H} NMR (125 MHz, CDCl_3_) δ: 166.4, 162.0, 146.5, 139.6, 136.5, 134.8, 132.1, 130.6, 128.9, 127.7, 127.7, 127.5, 127.1, 124.9, 61.5, 48.9, 22.6, 21.2, 13.5, 11.3 ppm. Anal. Calcd for C_24_H_25_NO_3_S: C, 70.73; H, 6.18; N, 3.44; S, 7.87. Found: C, 70.83; H, 6.12; N, 3.39; S, 7.92.

## Supporting Information Available

Copies of IR, 1H, 13C, and 2D NMR spectra of products and Cartesian coordinates of all computed structures (PDF)

## Dedication

This article is dedicated with warm appreciation to Prof. Joan Bosch on his 75th birthday.
