# Supplementary material for: Switchable Cycloadditions of Mesoionic Dipoles: Refreshing up a Regioselective Approach to Two Distinctive Heterocycles
Source: J Org Chem. 2022 Sep 14;87(19):12854–66. doi: 10.1021/acs.joc.2c01444 (PMC9552231; doi:10.1021/acs.joc.2c01444)
Supplement: Supplementary file 1 — jo2c01444_si_001.pdf [file jo2c01444_si_001.pdf]

Supporting Information

Switchable Cycloadditions of Mesoionic Dipoles: Refreshing Up a Regioselective Approach to Two Distinctive Heterocycles

M. Pilar Romero-Fernández,* Pedro Cintas, and Sergio Rojas-Buzo*

Department of Organic and Inorganic Chemistry, Faculty of Sciences, and IACYS-Green Chemistry and Sustainable Development Unit, University of Extremadura, 06006 Badajoz, Spain

Table of Contents

I. NMR Spectra. 3

II. FTIR spectra. 17

III. Two-dimensional NMR spectra. 24

IV. Computational Data and Cartesian Coordinates for Optimized Structures. 25

I. NMR Spectra.

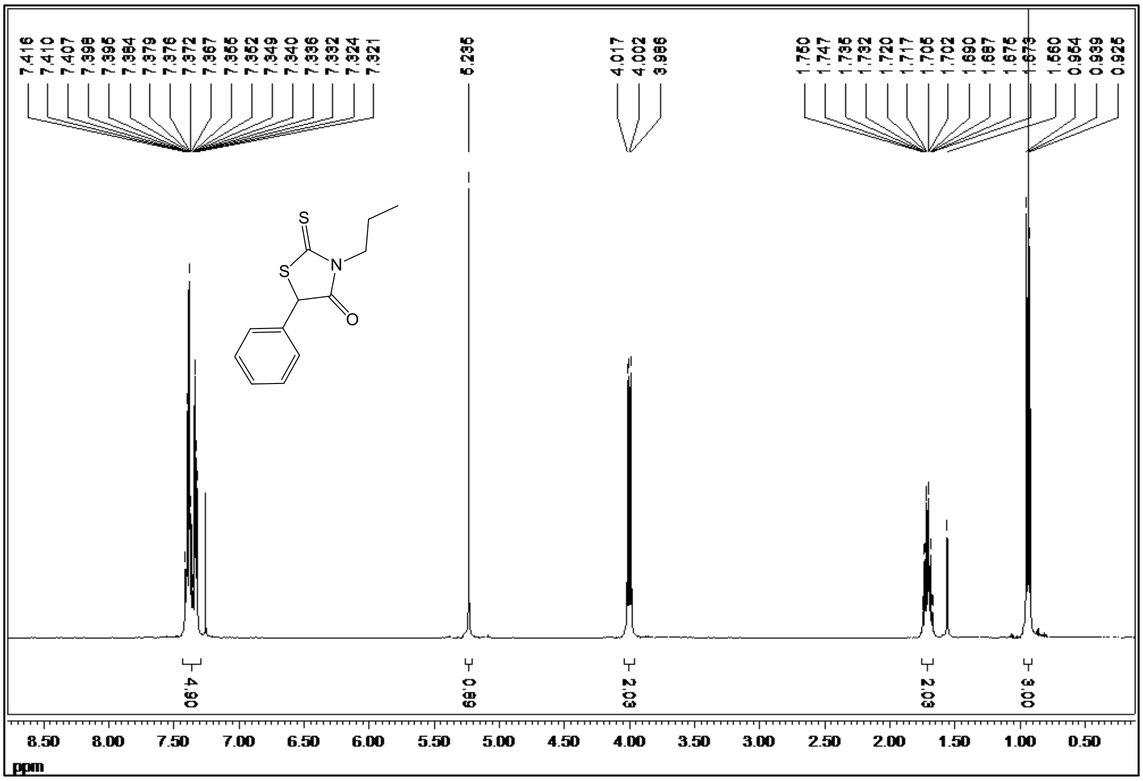

Figure S1: ^{1}H NMR (500 MHz, $CDCl_3$) spectrum of 5-Phenyl-3-propyl-2-thioxothiazolidin-4-one (**3**).

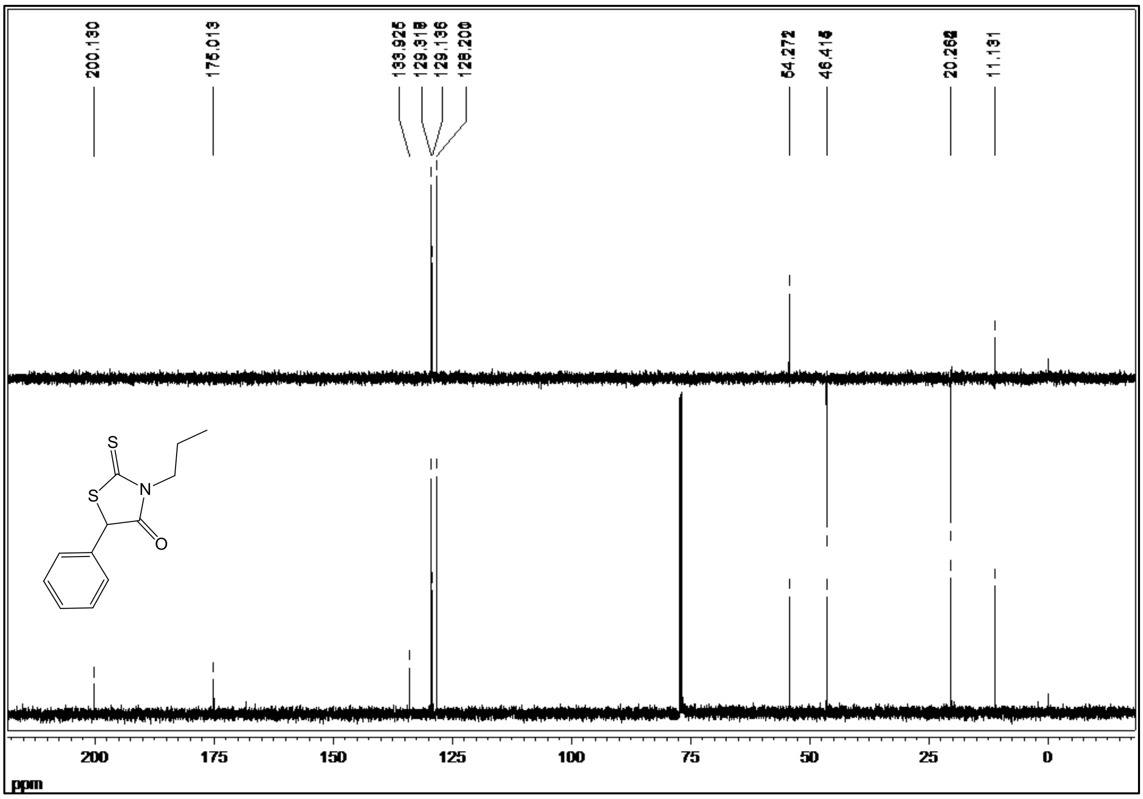

Figure S2: $^{13}C\{^1H\}$ NMR (125 MHz, $CDCl_3$) spectrum of 5-Phenyl-3-propyl-2-thioxothiazolidin-4-one (**3**).

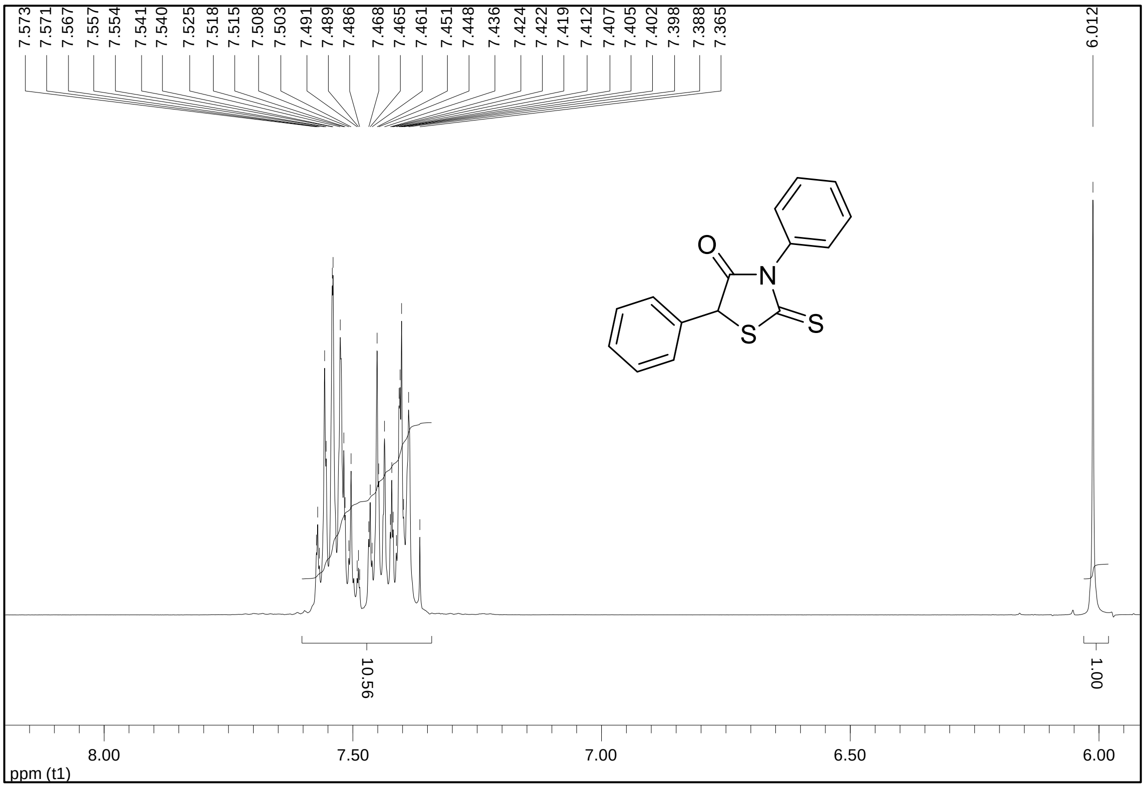

Figure S3: ^{1}H NMR (500 MHz, $CDCl_3$) spectrum of 3,5-Diphenyl-2-thioxothiazolidin-4-one (**4**).

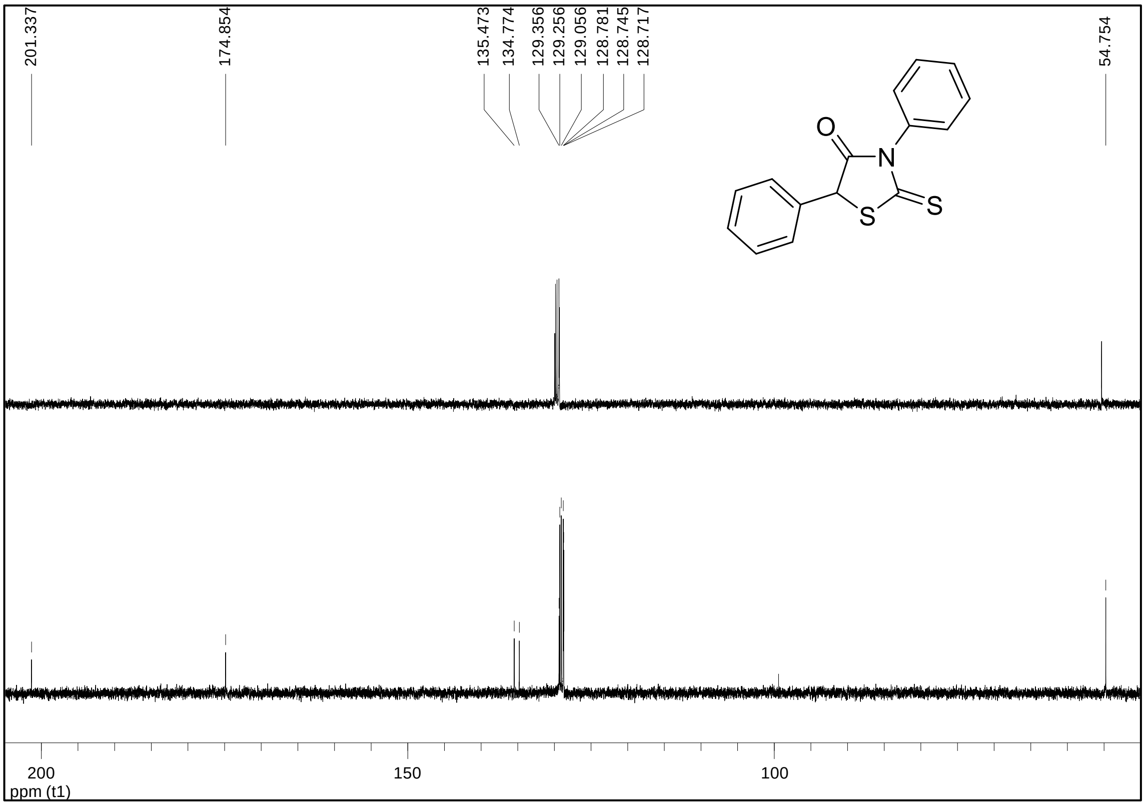

Figure S4: ^{13}C{^{1}H} NMR (125 MHz, $CDCl_3$) spectrum of 3,5-Diphenyl-2-thioxothiazolidin-4-one (**4**).

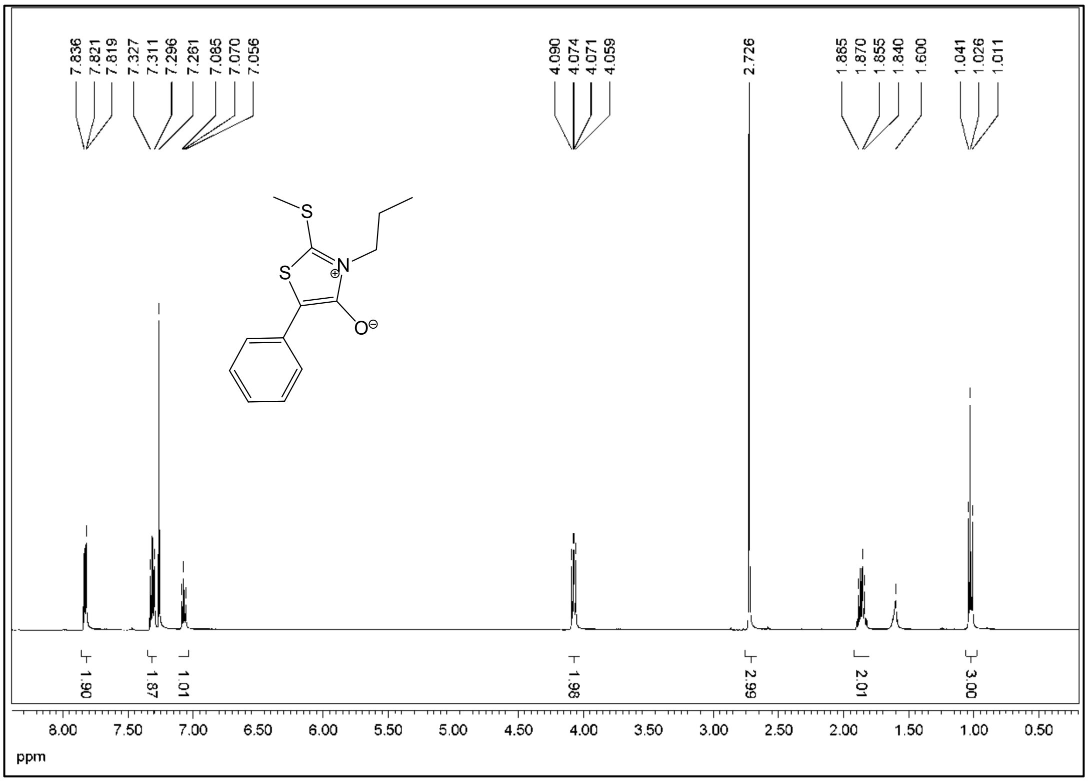

Figure S5: ^{1}H NMR (500 MHz, $CDCl_3$) spectrum of 2-(Methylthio)-5-phenyl-3-propylthiazol-3-ium-4-olate (**5**).

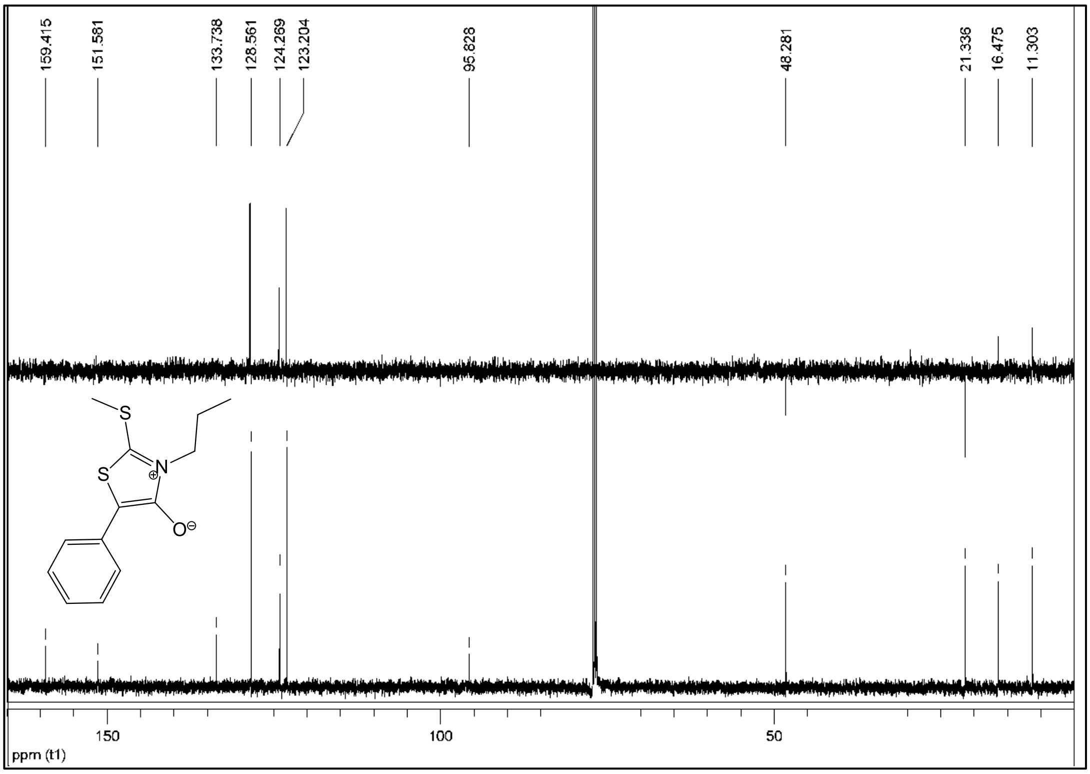

Figure S6: $^{13}C\{^{1}H\}$ NMR (125 MHz, $CDCl_3$) spectrum of 2-(Methylthio)-5-phenyl-3-propylthiazol-3-ium-4-olate (**5**).

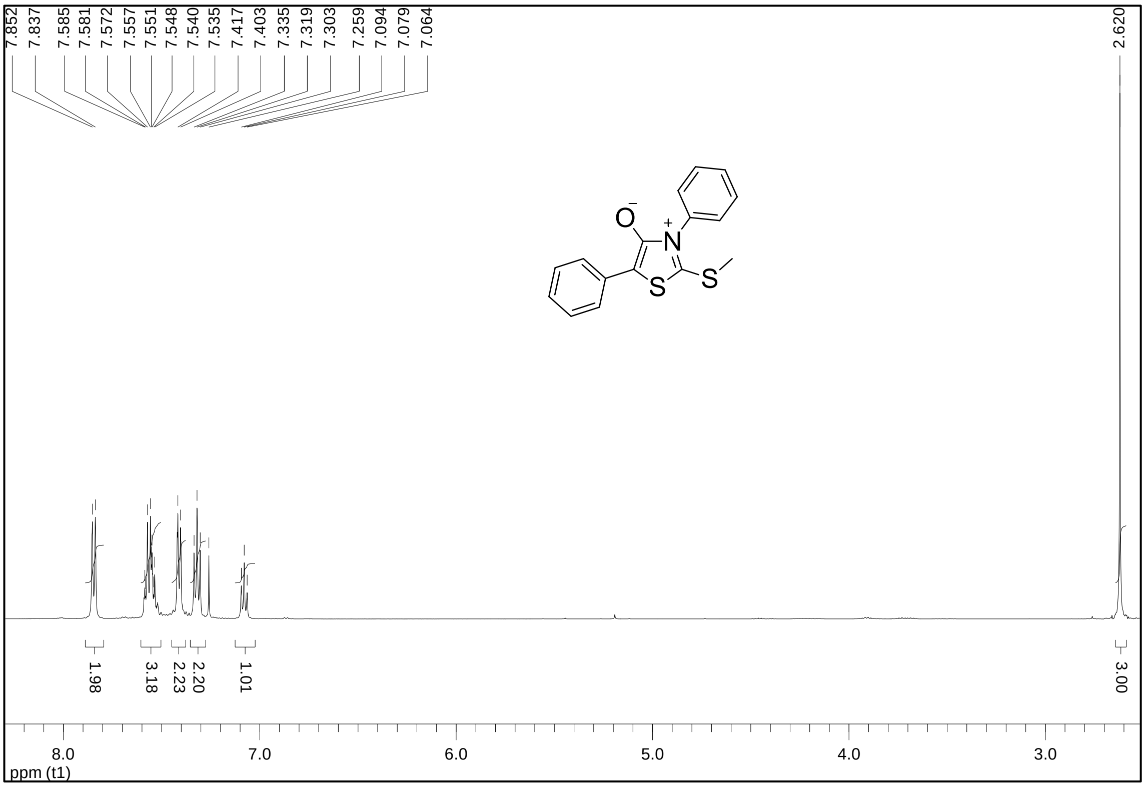

Figure S7: ^{1}H NMR (500 MHz, $CDCl_3$) spectrum of 2-(Methylthio)-3,5-diphenylthiazol-3-ium-4-olate (**6**).

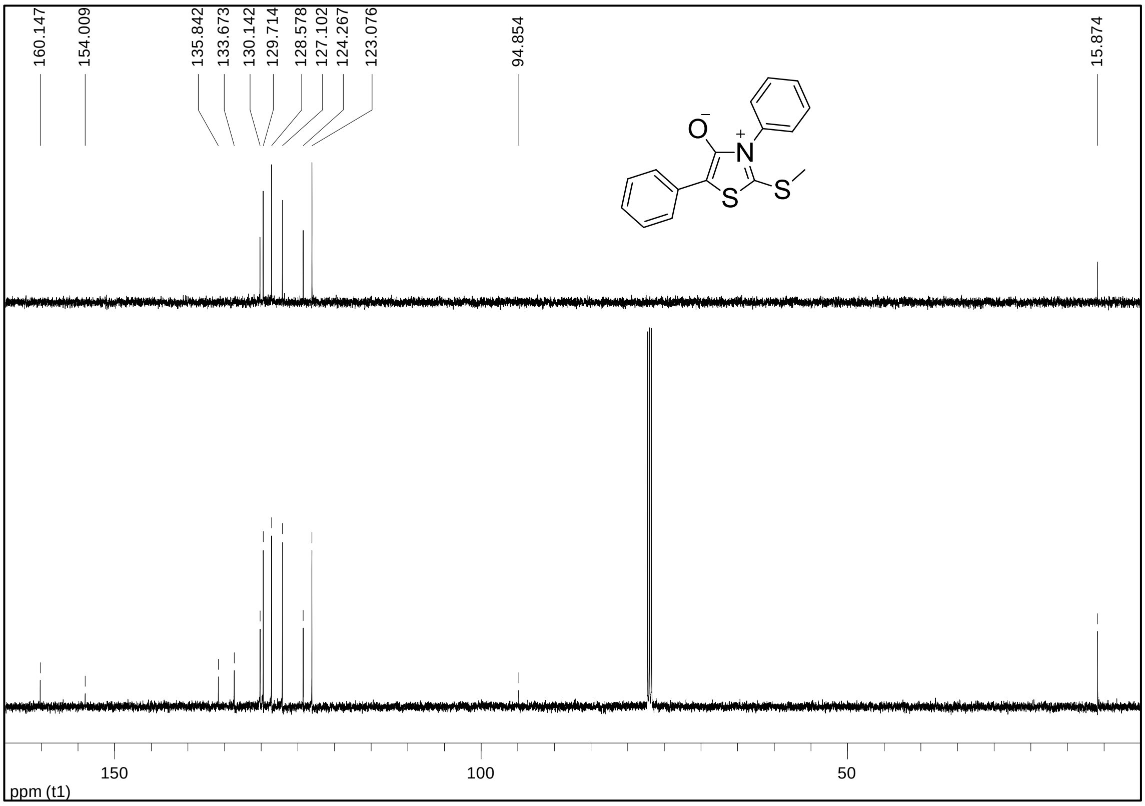

Figure S8: $^{13}C\{^{1}H\}$ NMR (125 MHz, $CDCl_3$) spectrum of 2-(Methylthio)-3,5-diphenylthiazol-3-ium-4-olate (**6**).

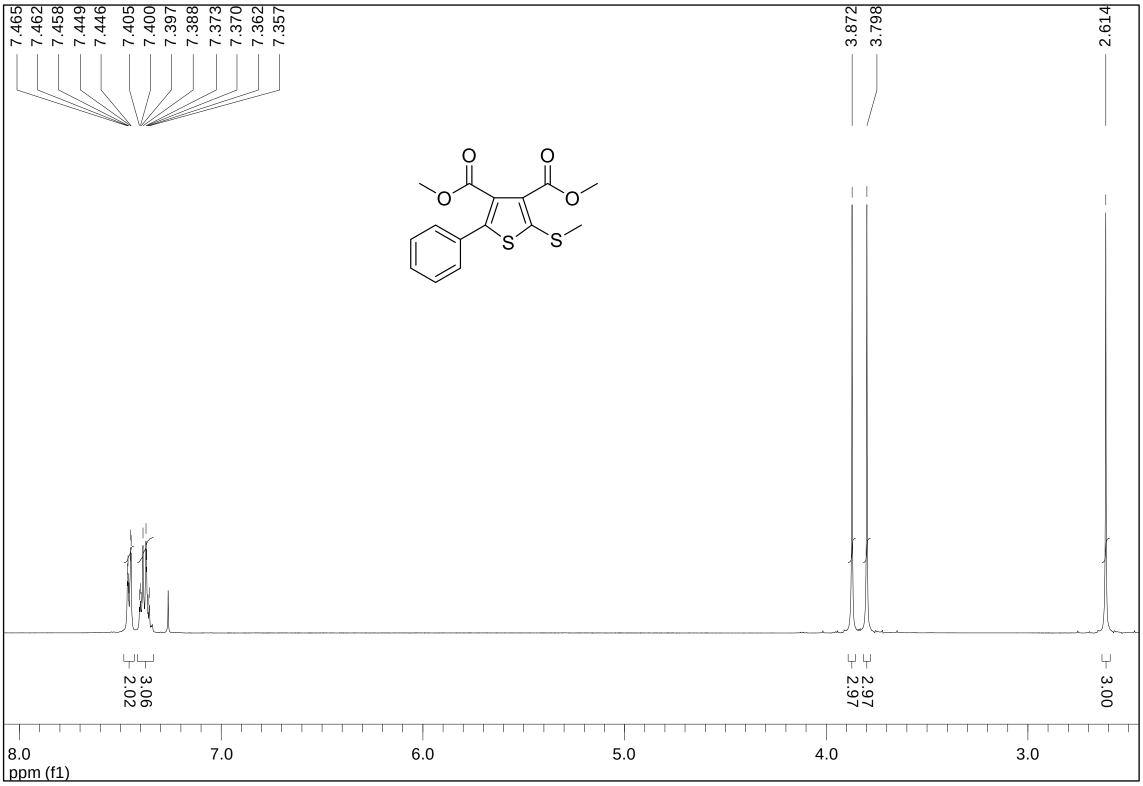

Figure S9: ^{1}H NMR (500 MHz, $CDCl_3$) spectrum of Dimethyl 2-(methylthio)-5-phenylthiophene-3,4-dicarboxylate (**9a**).

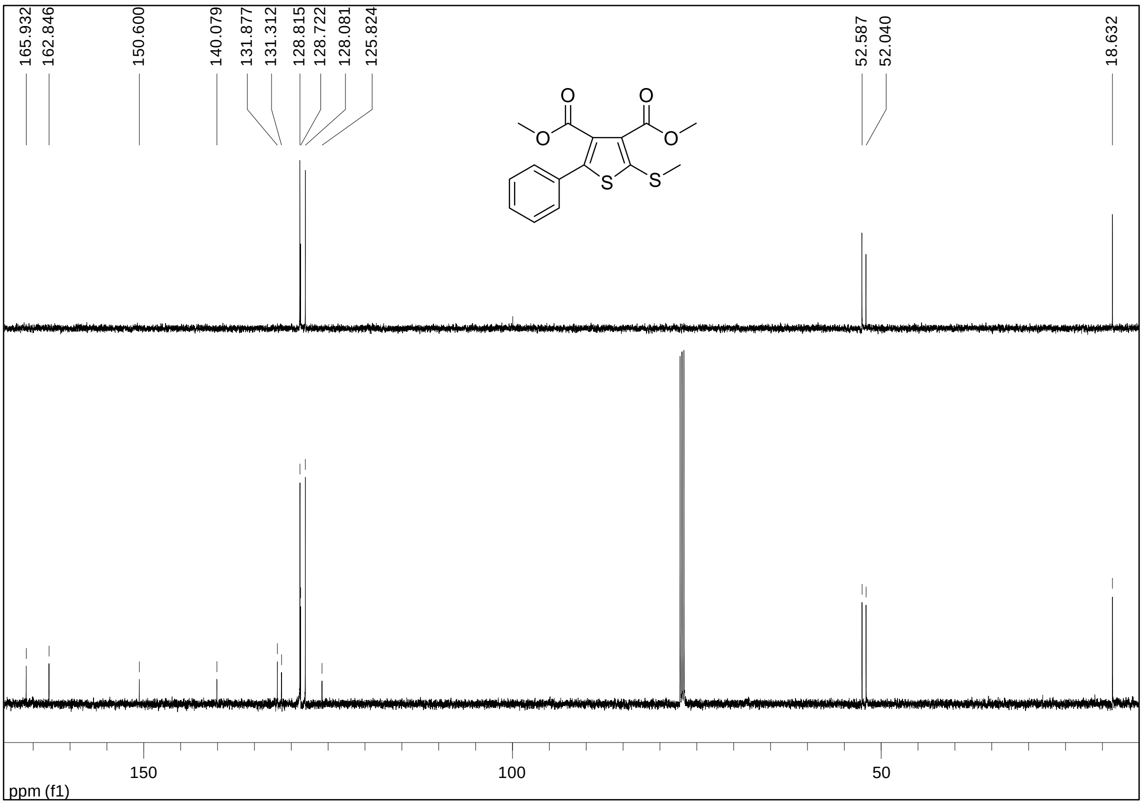

Figure S10: $^{13}C\{^{1}H\}$ NMR (125 MHz, $CDCl_3$) spectrum of Dimethyl 2-(methylthio)-5-phenylthiophene-3,4-dicarboxylate (**9a**).

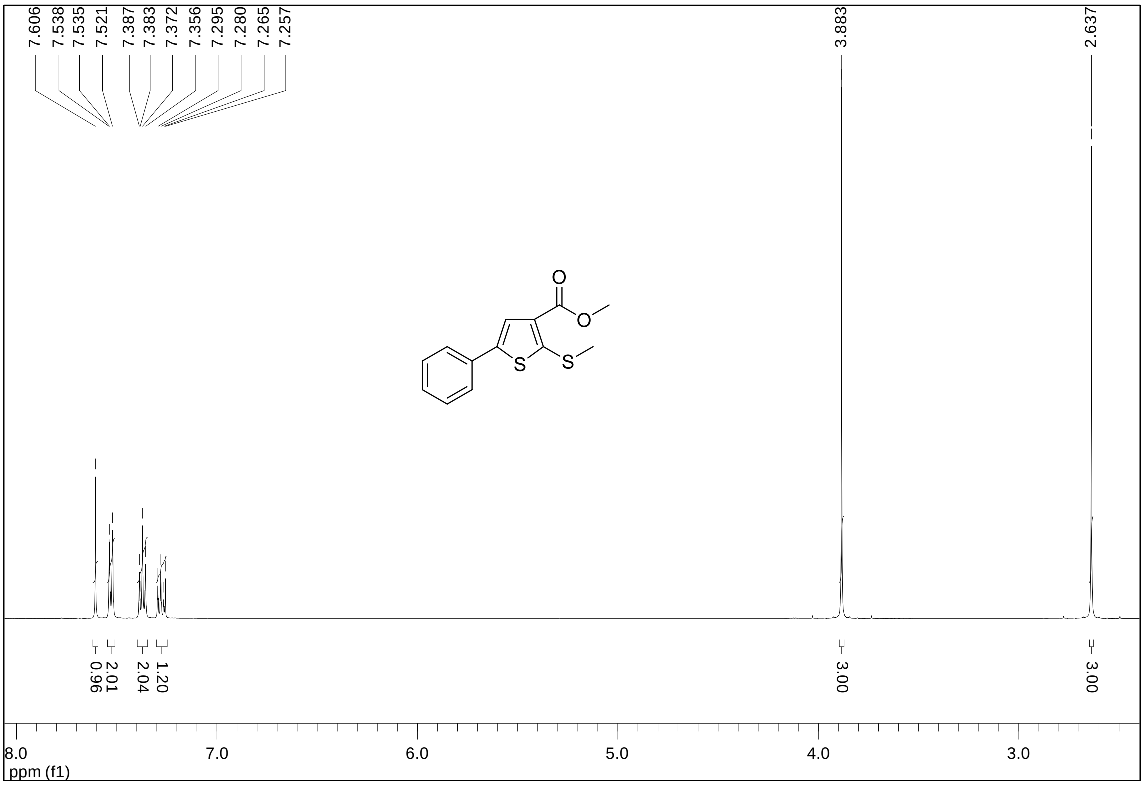

Figure S11: ^{1}H NMR (500 MHz, $CDCl_3$) spectrum of Methyl 2-(methylthio)-5-phenylthiophene-3-carboxylate (**9b**).

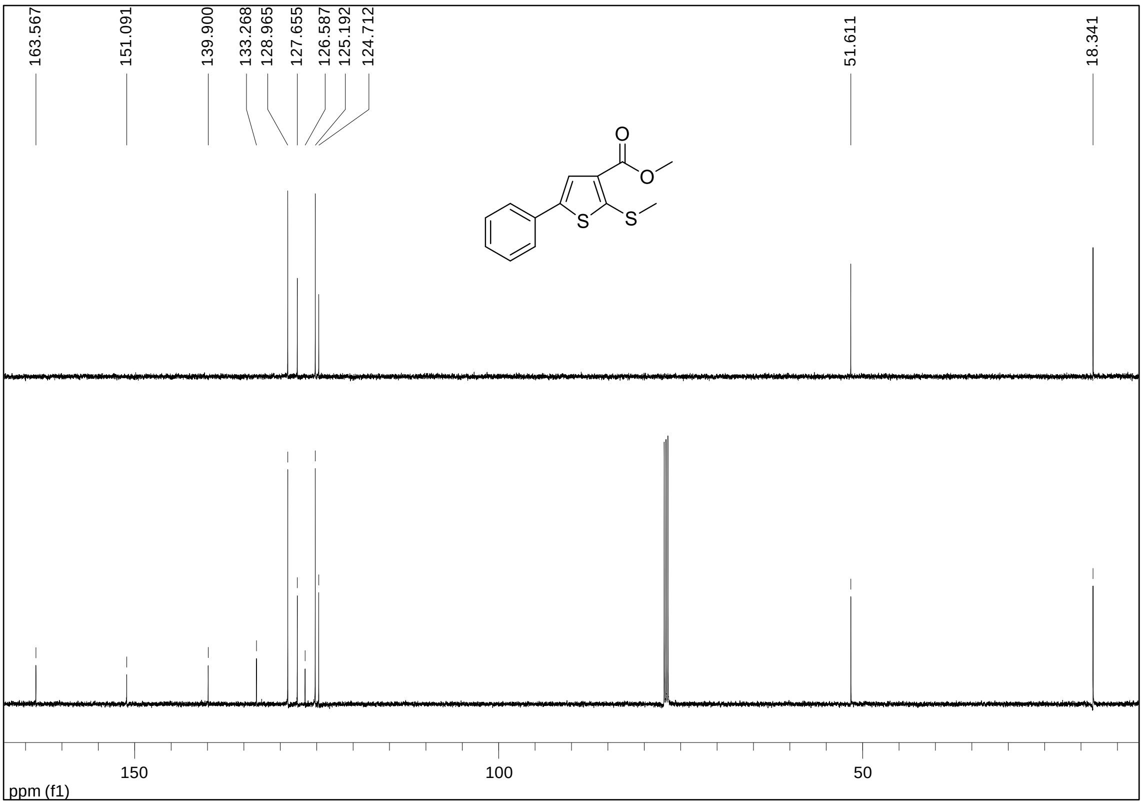

Figure S12: ^{13}C{^{1}H} NMR (125 MHz, $CDCl_3$) spectrum of Methyl 2-(methylthio)-5-phenylthiophene-3-carboxylate (**9b**).

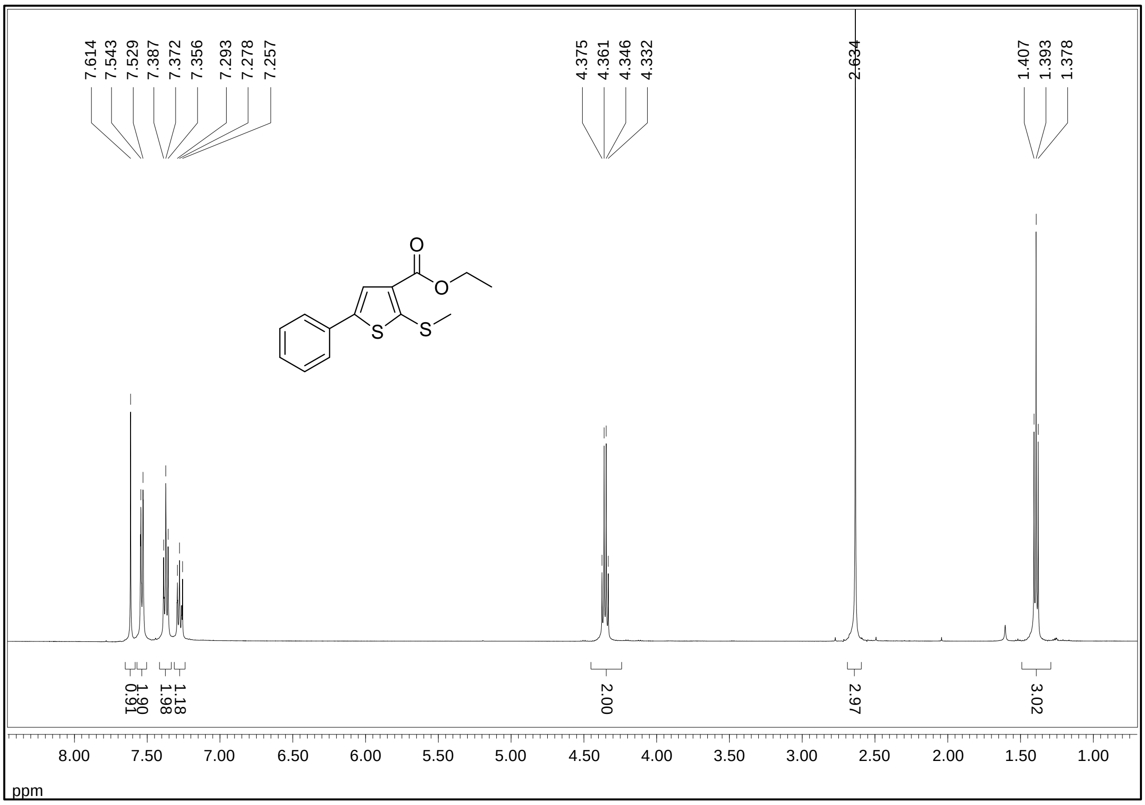

Figure S13: ^{1}H NMR (500 MHz, $CDCl_3$) spectrum of Ethyl 2-(methylthio)-5-phenylthiophene-3-carboxylate (**9c**).

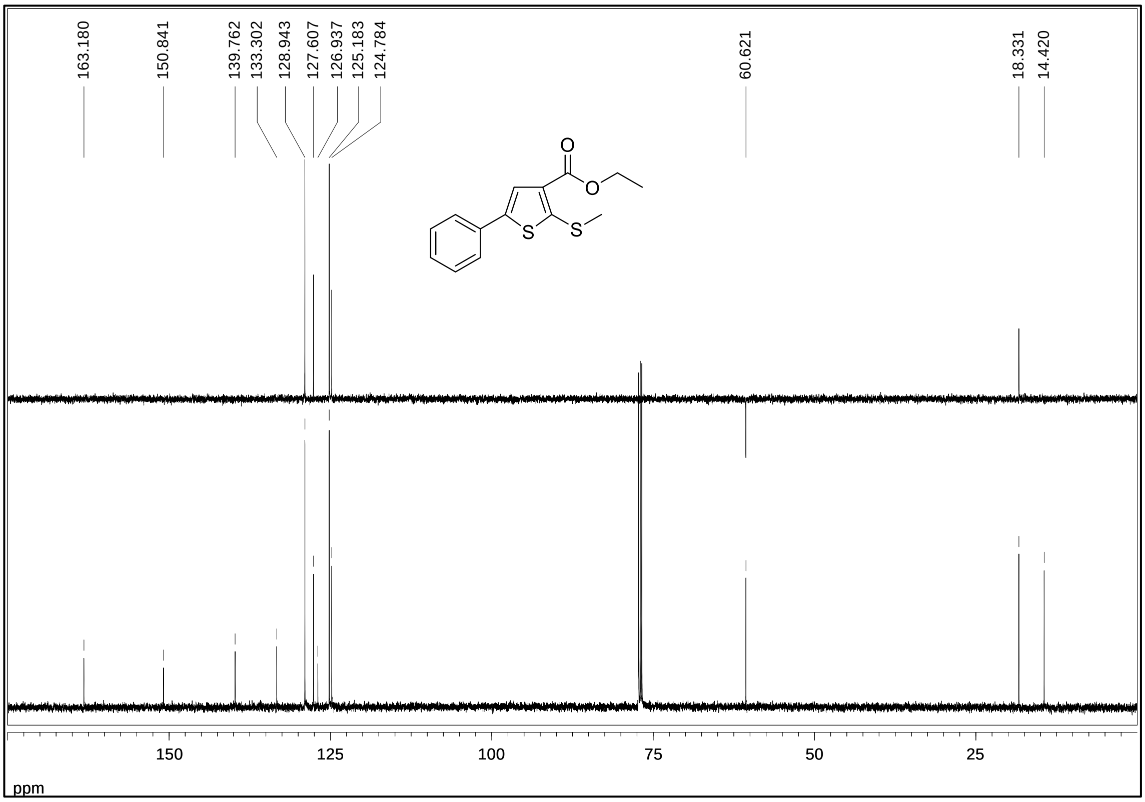

Figure S14: $^{13}C\{^{1}H\}$ NMR (125 MHz, $CDCl_3$) spectrum of Ethyl 2-(methylthio)-5-phenylthiophene-3-carboxylate (**9c**).

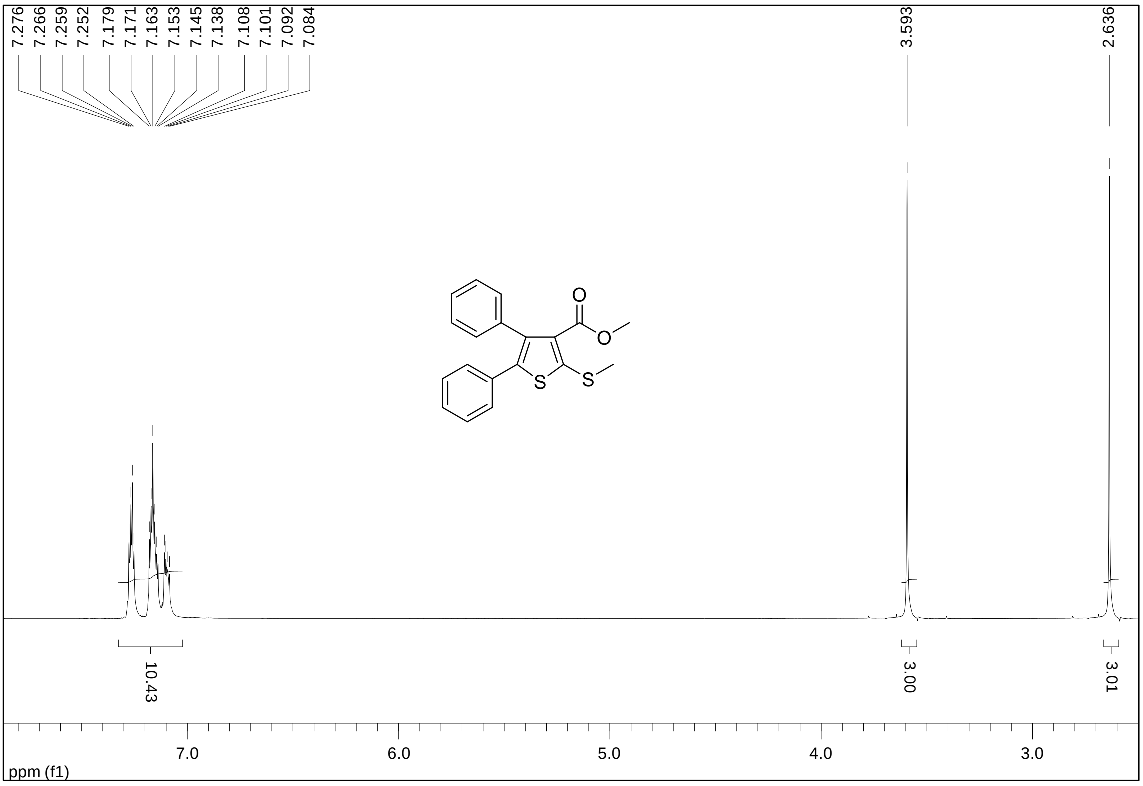

Figure S15: ^{1}H NMR (500 MHz, $CDCl_3$) spectrum of Methyl 2-(methylthio)-4,5-diphenylthiophene-3-carboxylate (**9d**).

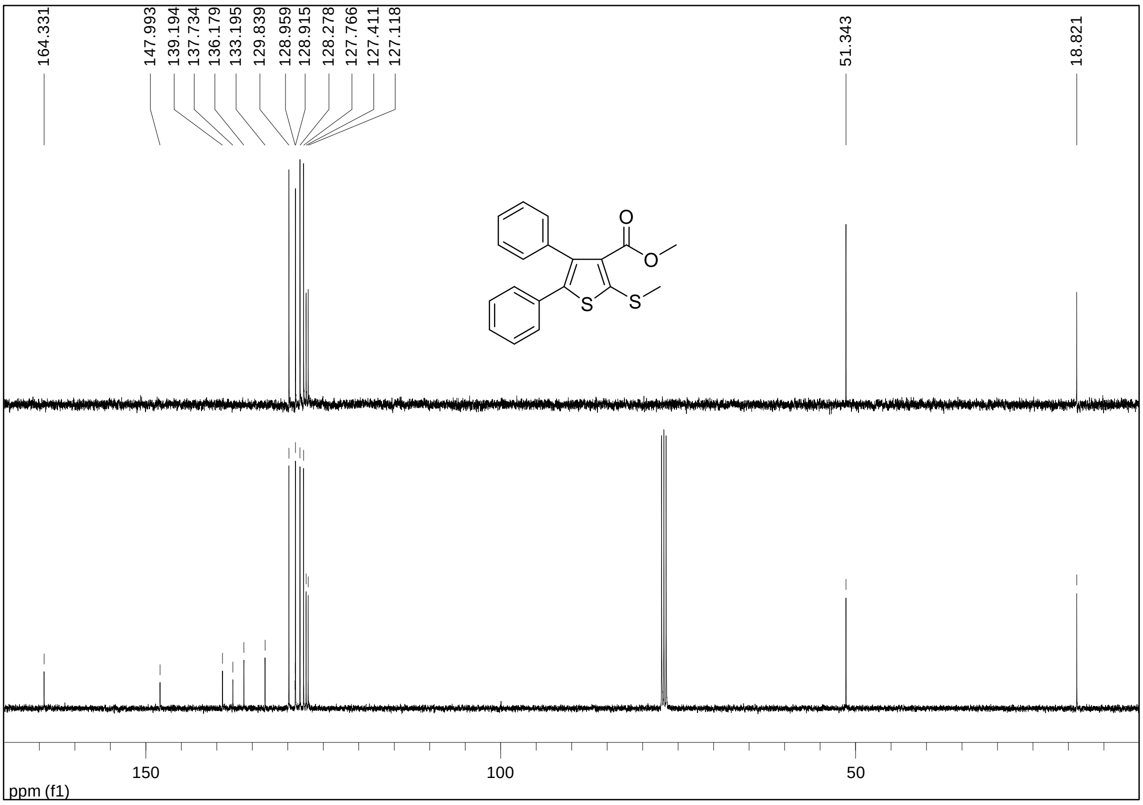

Figure S16: $^{13}C\{^{1}H\}$ NMR (125 MHz, $CDCl_3$) spectrum of Methyl 2-(methylthio)-4,5-diphenylthiophene-3-carboxylate (**9d**).

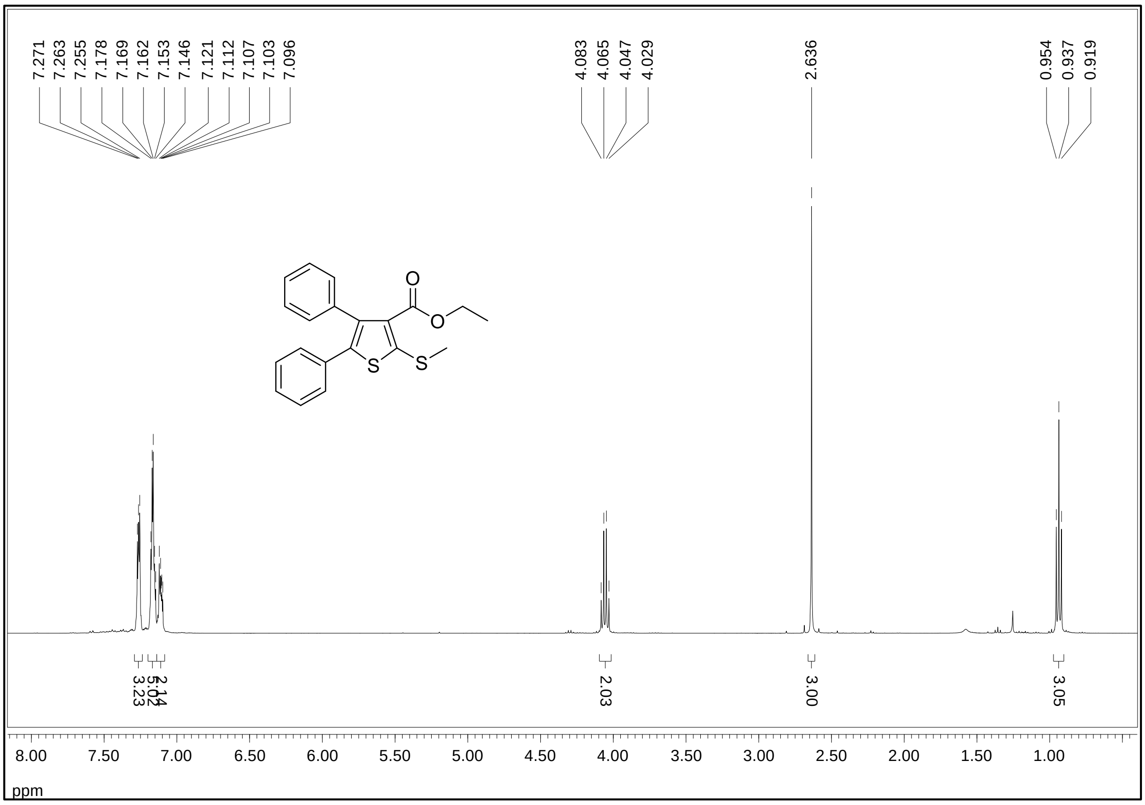

Figure S17: ^{1}H NMR (500 MHz, $CDCl_3$) spectrum of Ethyl 2-(methylthio)-4,5-diphenylthiophene-3-carboxylate (**9e**).

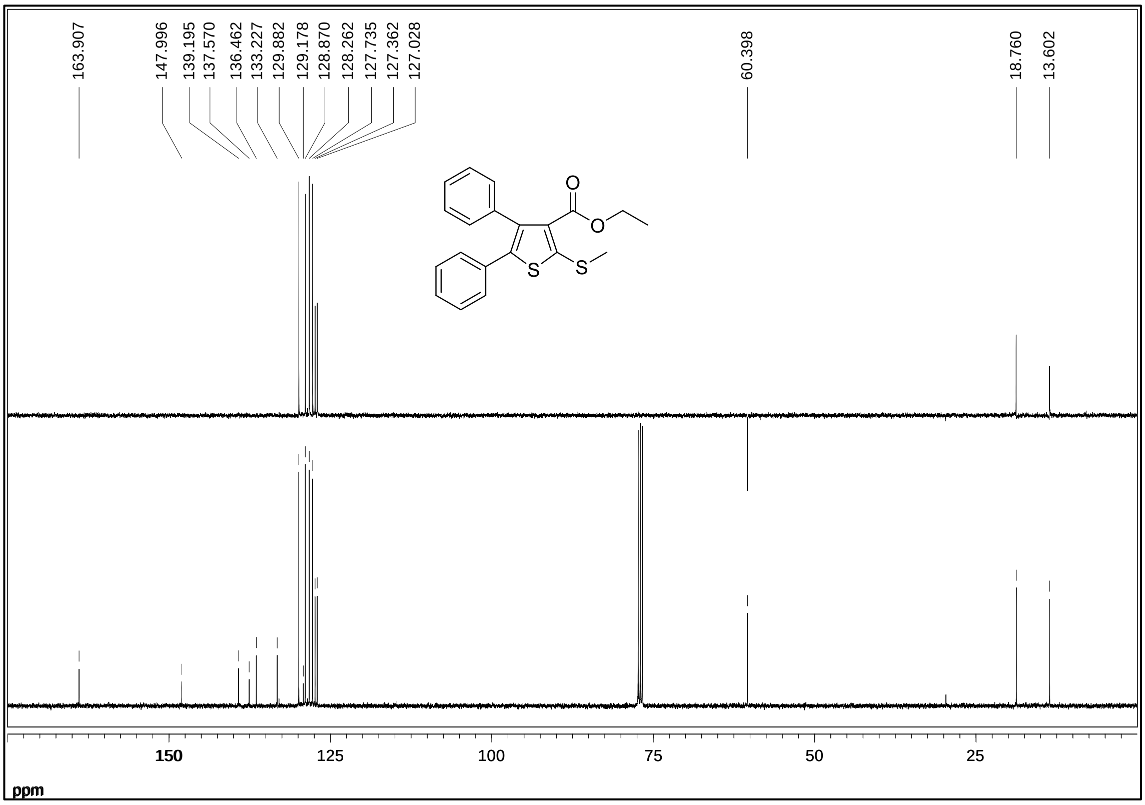

Figure S18: ^{13}C{^{1}H} NMR (125 MHz, $CDCl_3$) spectrum of Ethyl 2-(methylthio)-4,5-diphenylthiophene-3-carboxylate (**9e**).

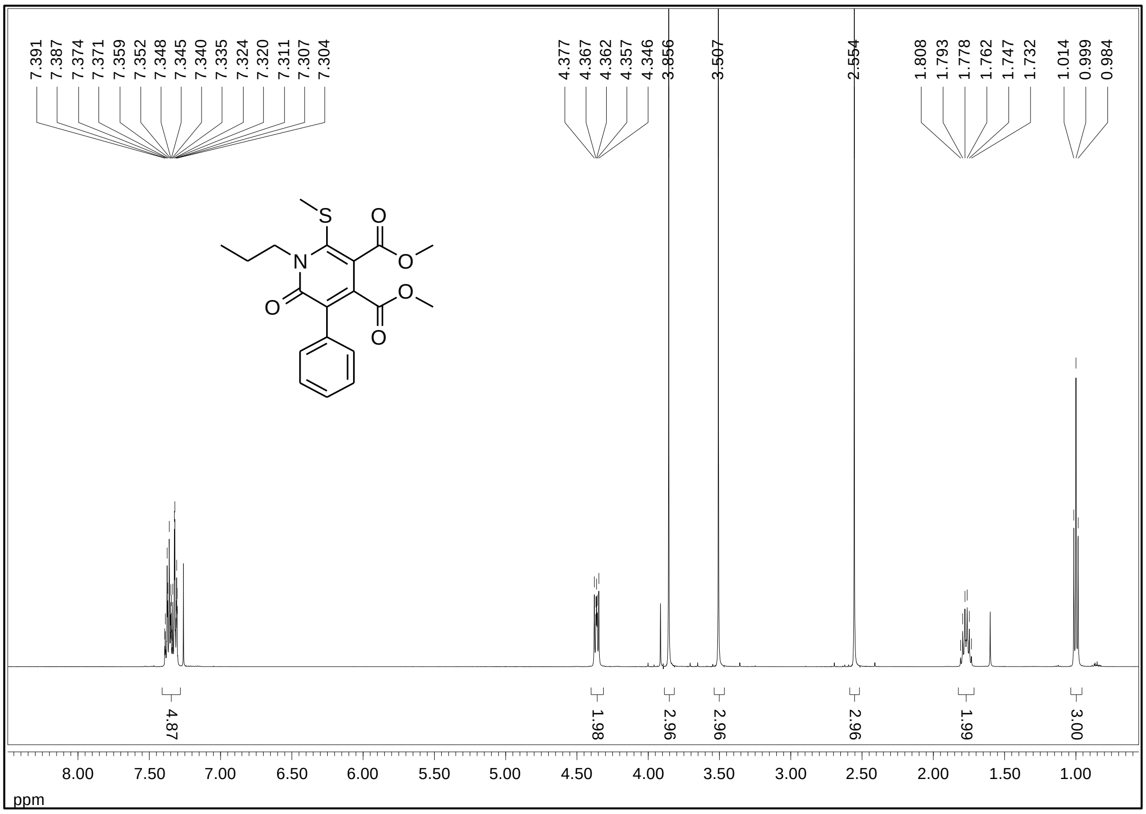

Figure S19: ^{1}H NMR (500 MHz, $CDCl_3$) spectrum of Dimethyl 2-(methylthio)-6-oxo-5-phenyl-1-propyl-1,6-dihydropyridine-3,4-dicarboxylate (**10a**).

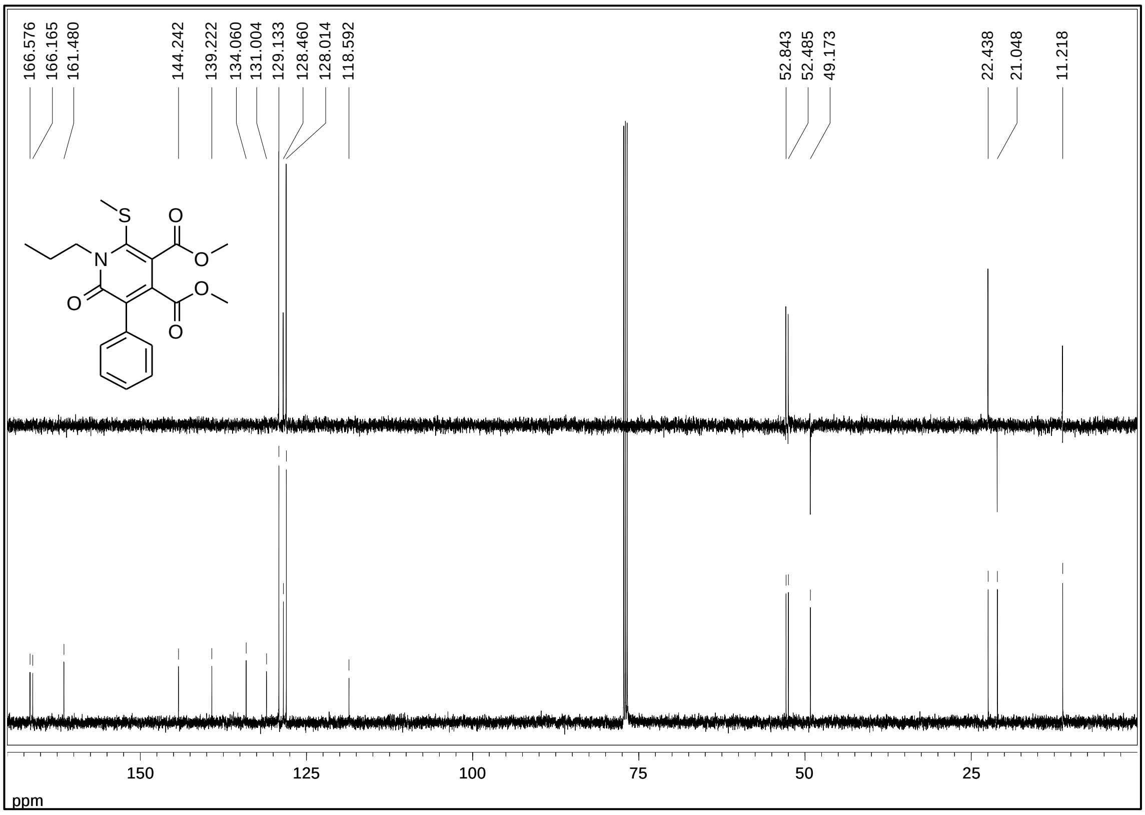

Figure S20: $^{13}C\{^{1}H\}$ NMR (125 MHz, $CDCl_3$) spectrum of Dimethyl 2-(methylthio)-6-oxo-5-phenyl-1-propyl-1,6-dihydropyridine-3,4-dicarboxylate (**10a**).

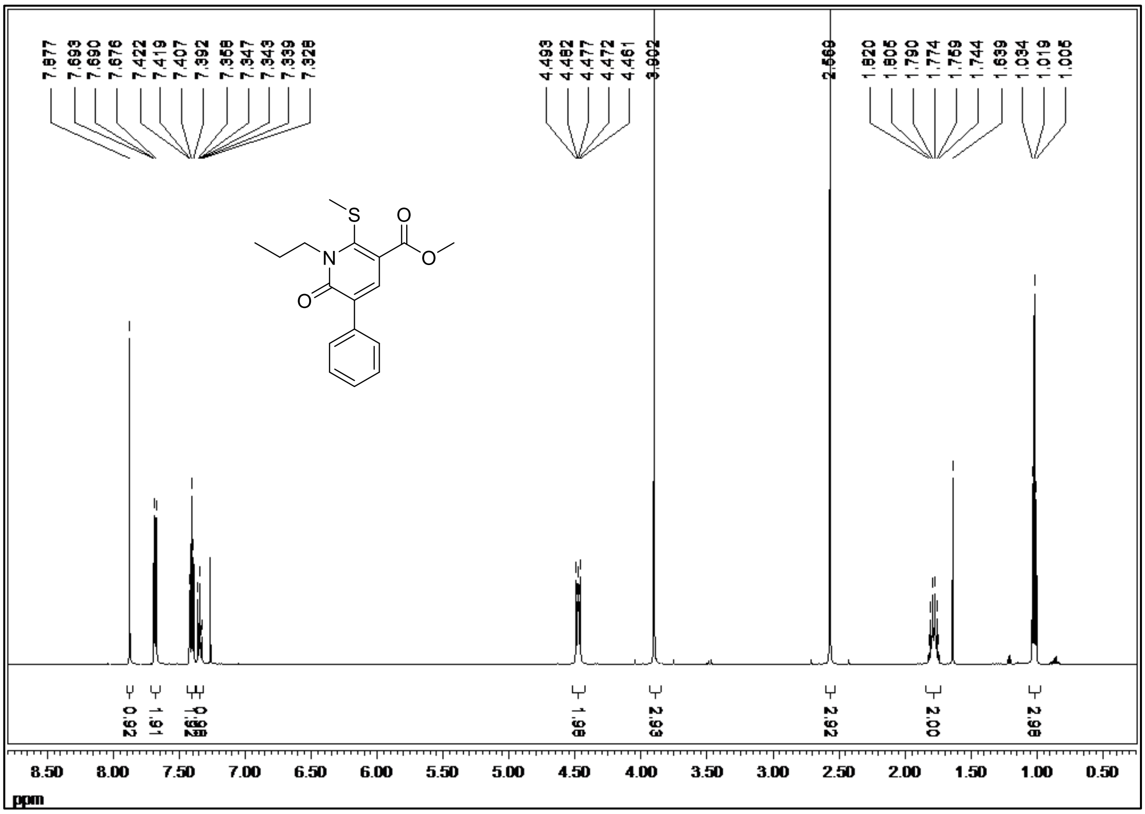

Figure S21: ^{1}H NMR (500 MHz, $CDCl_3$) spectrum of Methyl 2-(methylthio)-6-oxo-5-phenyl-1-propyl-1,6-dihydropyridine-3-carboxylate (**10b**).

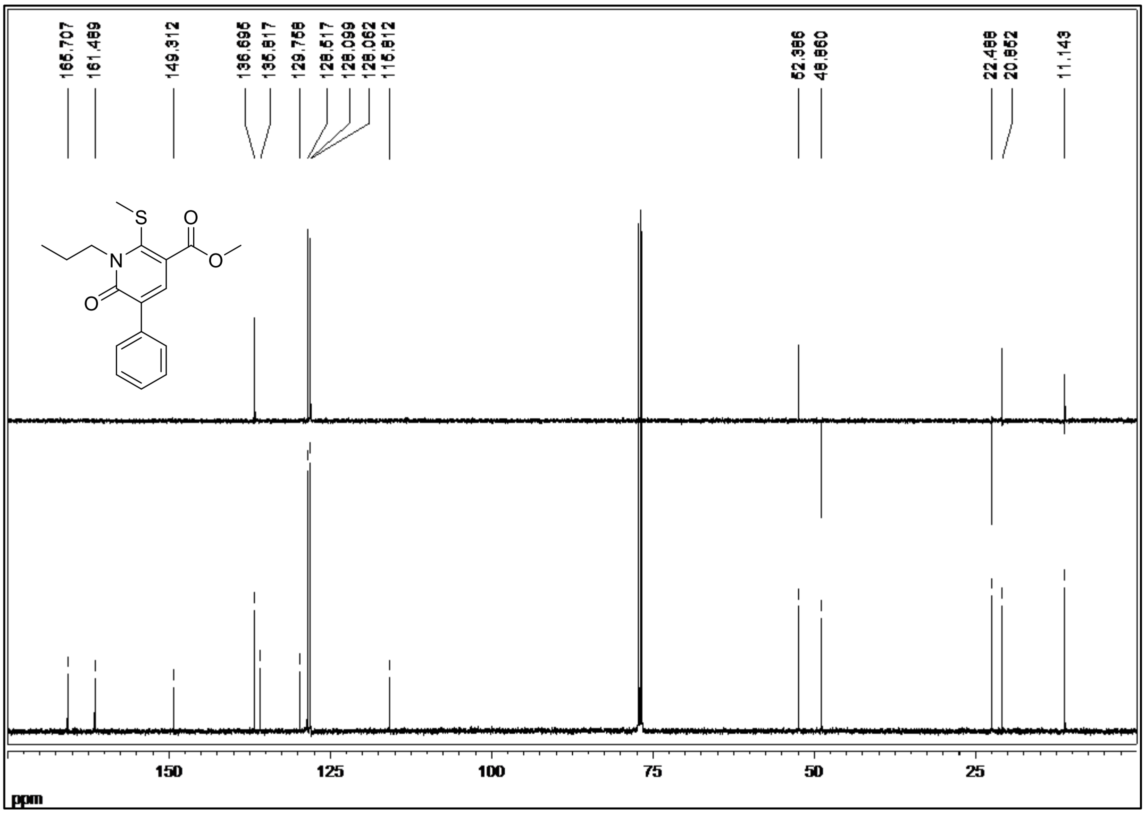

Figure S22: $^{13}C\{^{1}H\}$ NMR (125 MHz, $CDCl_3$) spectrum of Methyl 2-(methylthio)-6-oxo-5-phenyl-1-propyl-1,6-dihydropyridine-3-carboxylate (**10b**).

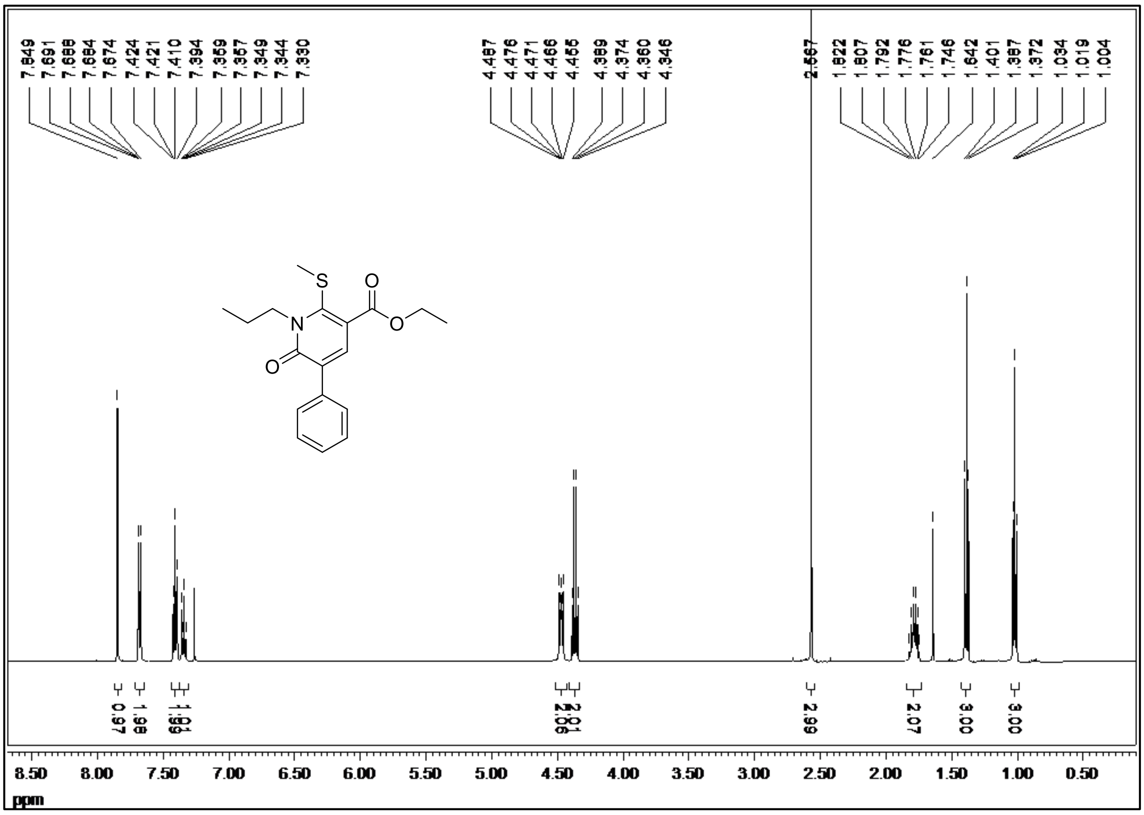

Figure S23: ^{1}H NMR (500 MHz, $CDCl_3$) spectrum of Ethyl 2-(Methylthio)-6-oxo-5-phenyl-1-propyl-1,6-dihydropyridine-3-carboxylate (**10c**).

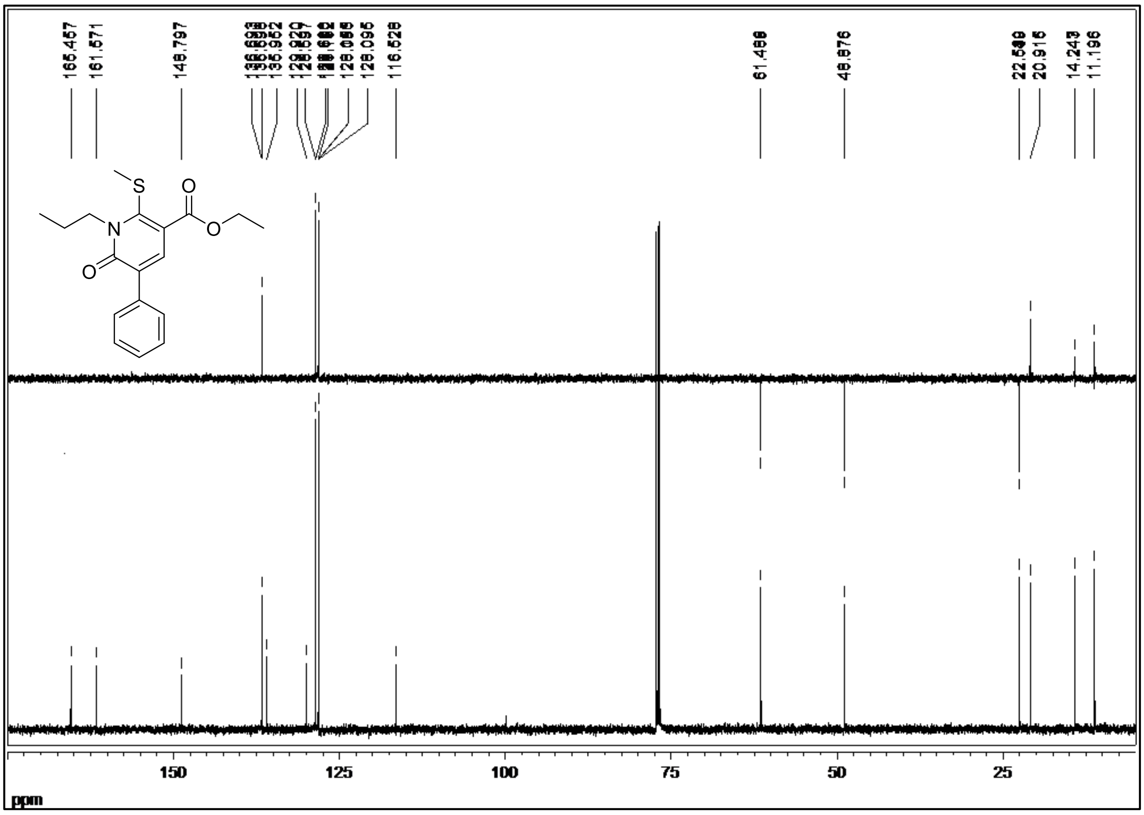

Figure S24: $^{13}C\{^{1}H\}$ NMR (125 MHz, $CDCl_3$) spectrum of Ethyl 2-(Methylthio)-6-oxo-5-phenyl-1-propyl-1,6-dihydropyridine-3-carboxylate (**10c**).

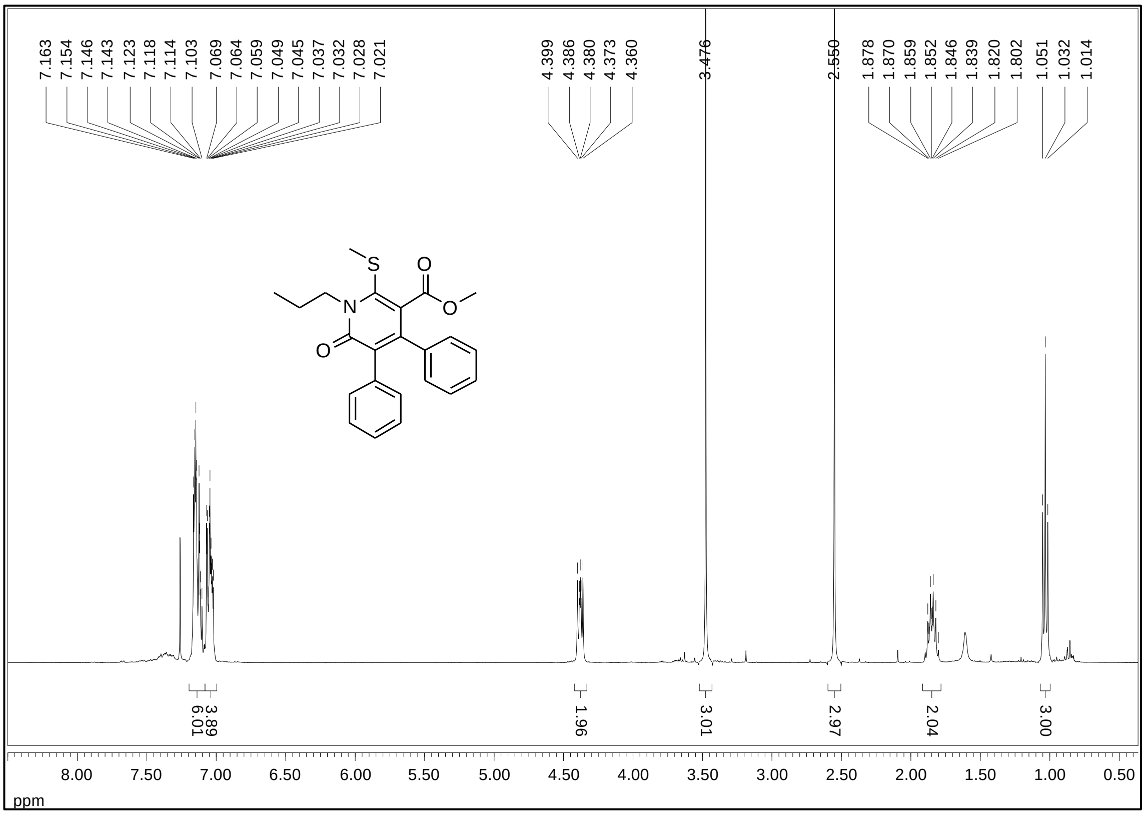

Figure S25: ^{1}H NMR (500 MHz, $CDCl_3$) spectrum of Methyl 2-(methylthio)-6-oxo-4,5-diphenyl-1-propyl-1,6-dihydropyridine-3-carboxylate (**10d**).

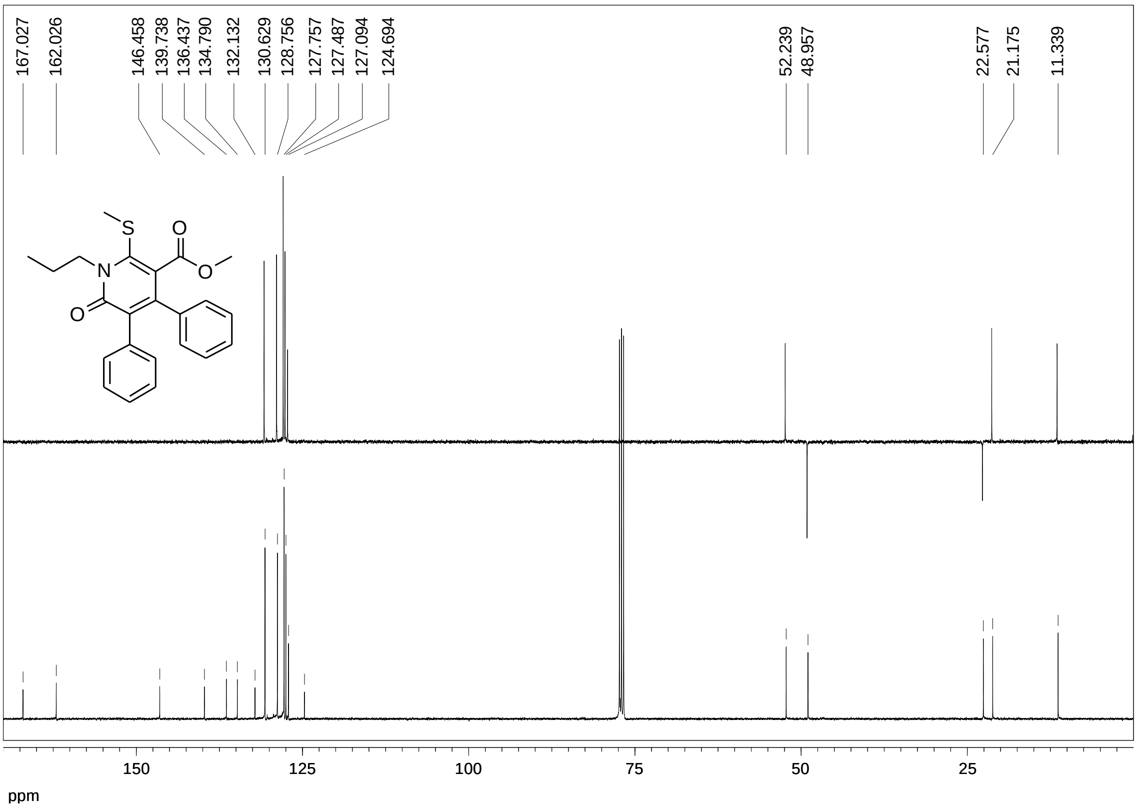

Figure S26: $^{13}C\{^{1}H\}$ NMR (125 MHz, $CDCl_3$) spectrum of Methyl 2-(methylthio)-6-oxo-4,5-diphenyl-1-propyl-1,6-dihydropyridine-3-carboxylate (**10d**).

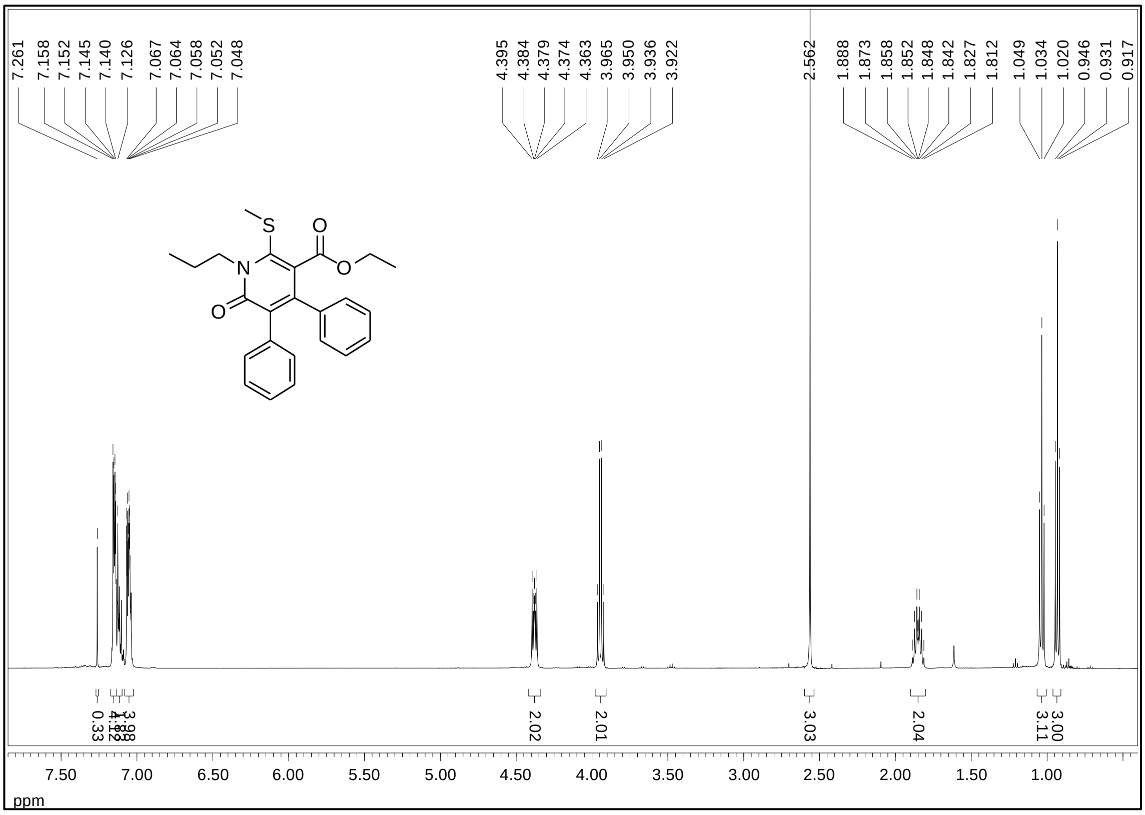

Figure S27: ^{1}H NMR (500 MHz, $CDCl_3$) spectrum of Ethyl 2-(methylthio)-6-oxo-4,5-diphenyl-1-propyl-1,6-dihydropyridine-3-carboxylate (**10e**).

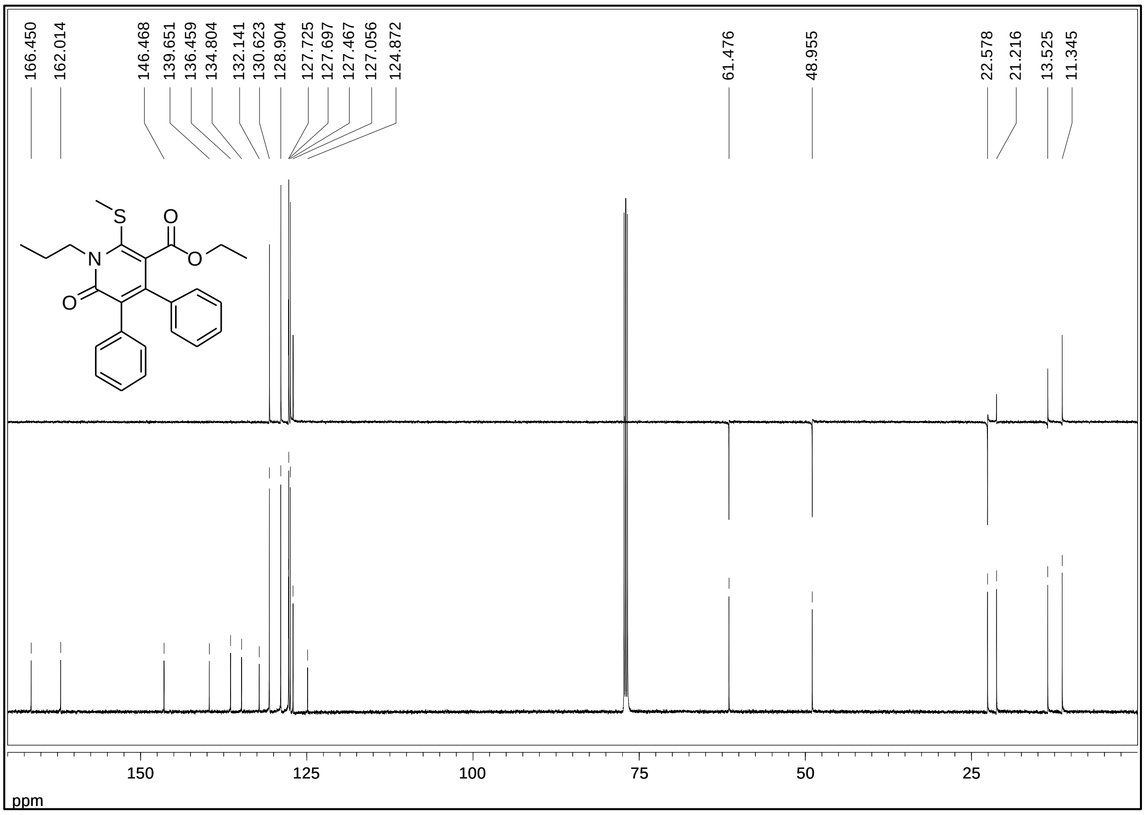

Figure S28: $^{13}C\{^{1}H\}$ NMR (125 MHz, $CDCl_3$) spectrum of Ethyl 2-(methylthio)-6-oxo-4,5-diphenyl-1-propyl-1,6-dihydropyridine-3-carboxylate (**10e**).

II. FTIR spectra.

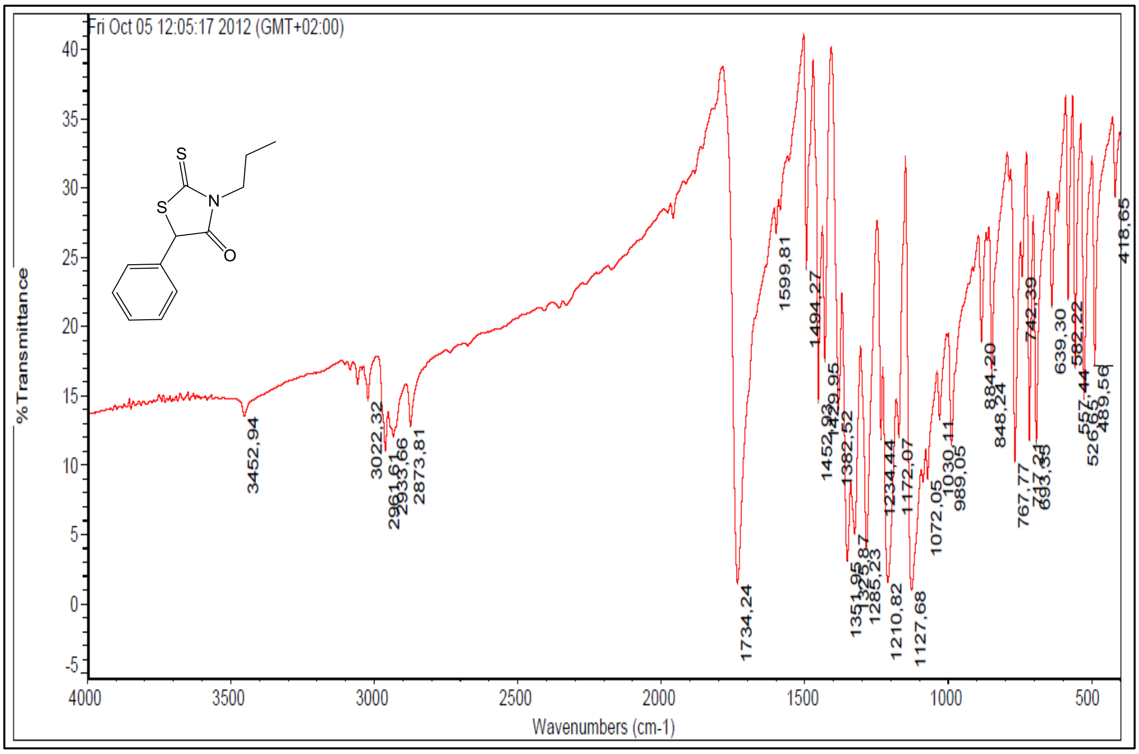

Figure S29: FTIR spectrum of 5-Phenyl-3-propyl-2-thioxothiazolidin-4-one (**3**).

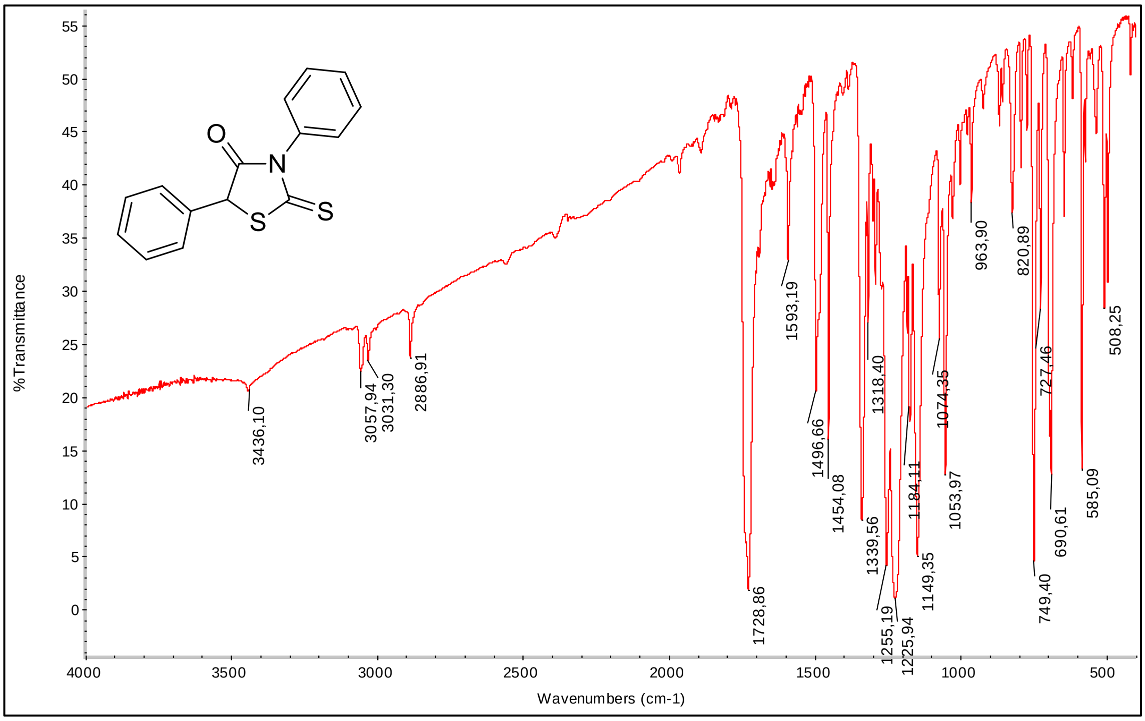

Figure S30: FTIR spectrum of 3,5-Diphenyl-2-thioxothiazolidin-4-one (**4**).

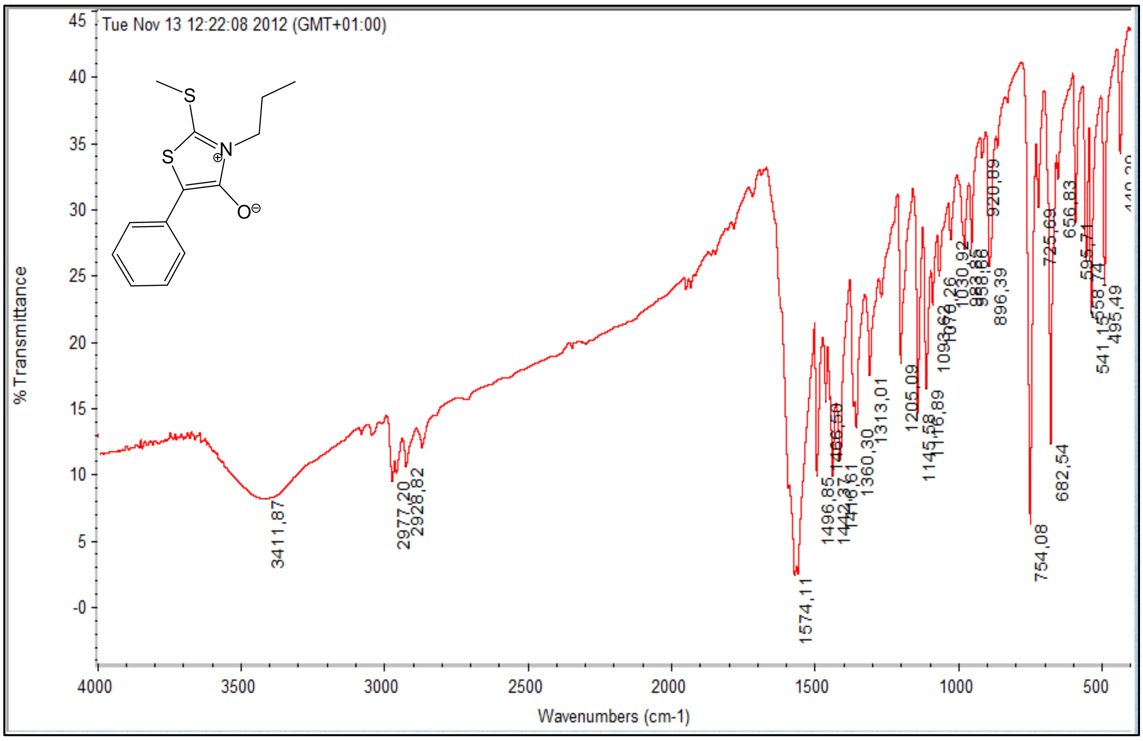

Figure S31: FTIR spectrum of 2-(Methylthio)-5-phenyl-3-propylthiazol-3-ium-4-olate (**5**).

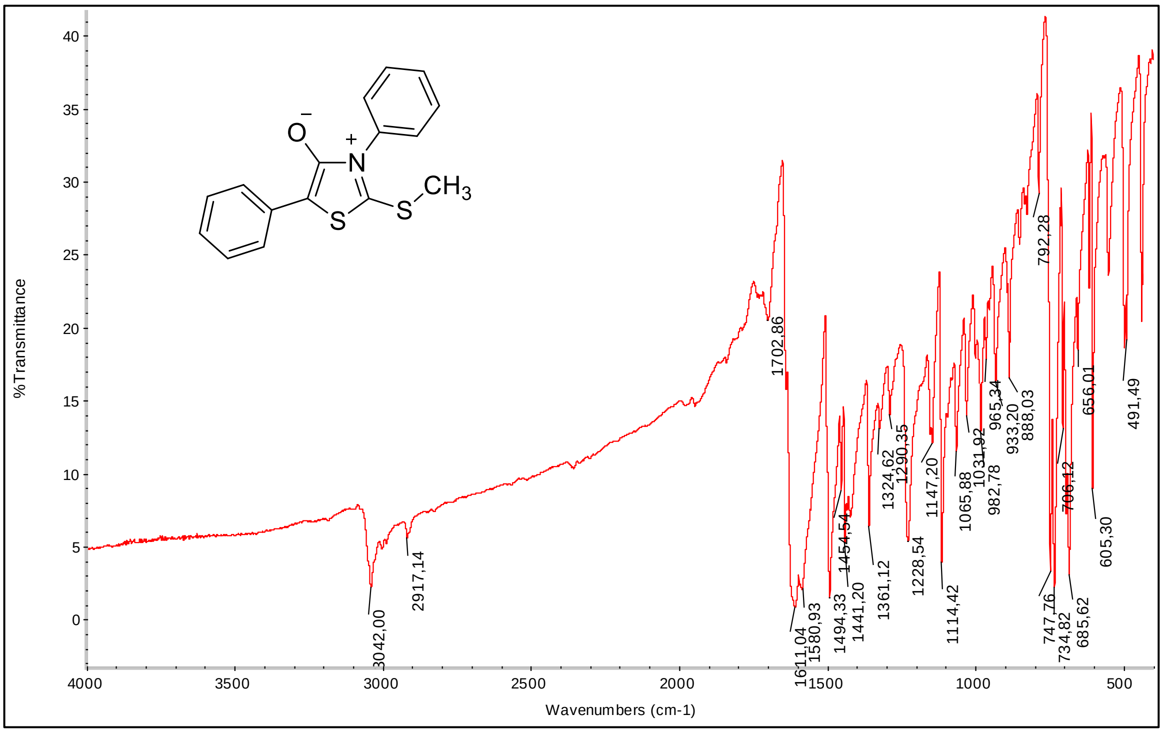

Figure S32: FTIR spectrum of 2-(Methylthio)-3,5-diphenylthiazol-3-ium-4-olate (**6**).

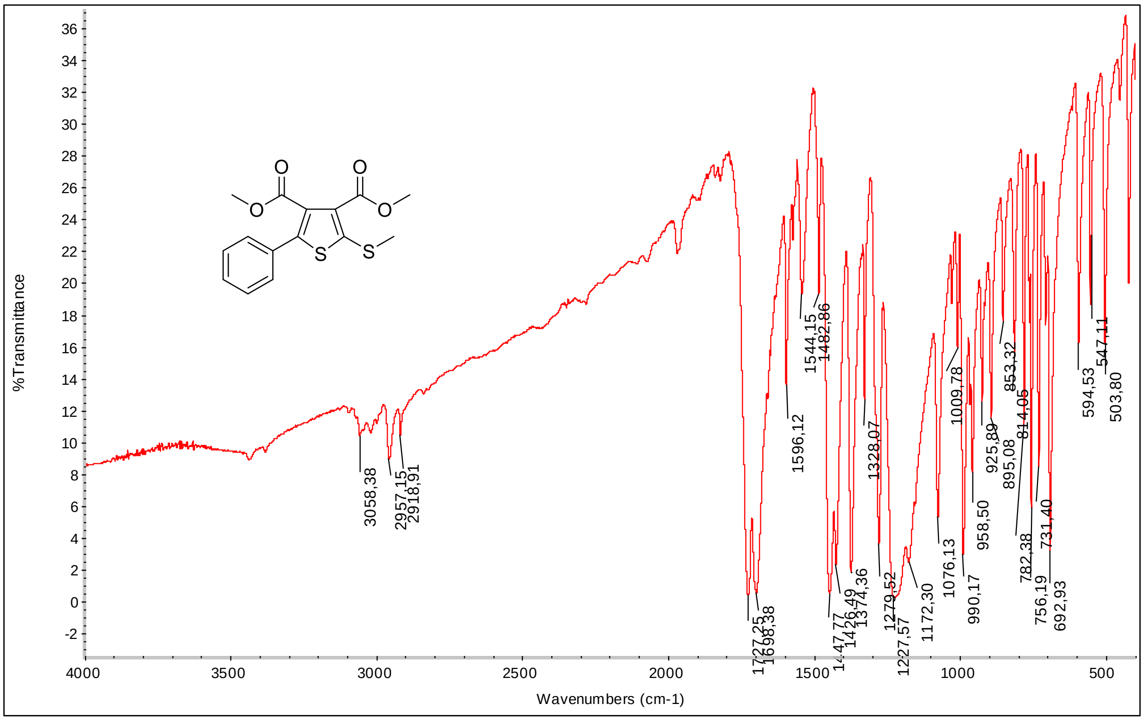

Figure S33: FTIR spectrum of Dimethyl 2-(methylthio)-5-phenylthiophene-3,4-dicarboxylate (**9a**).

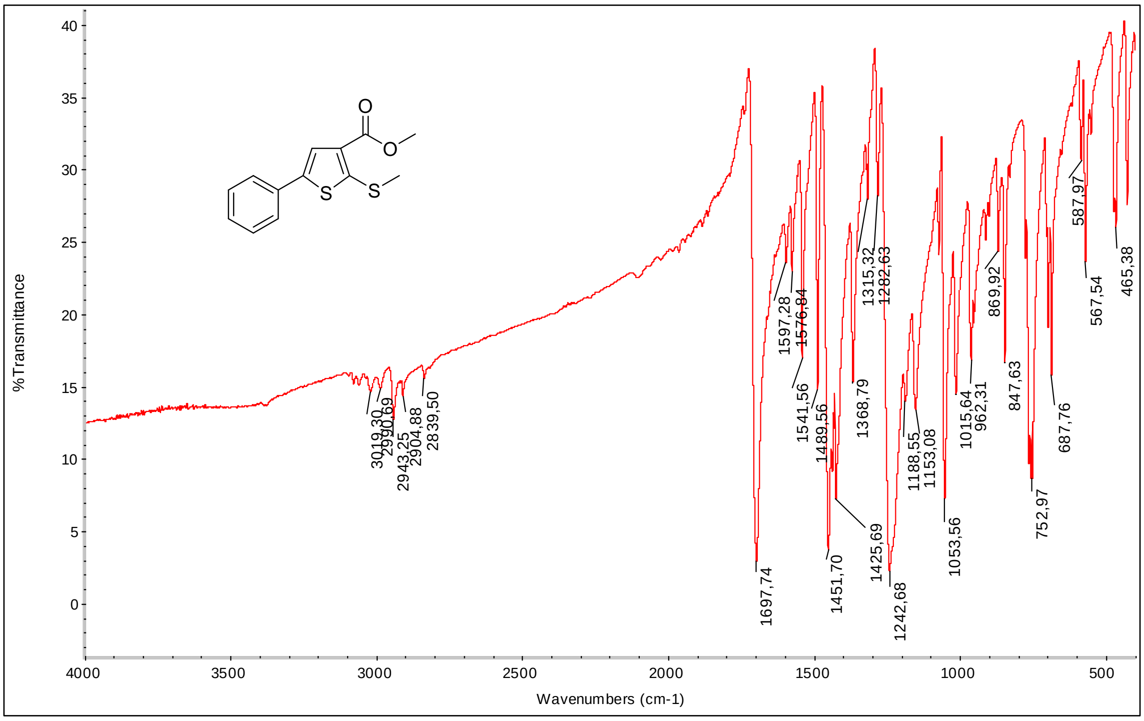

Figure S34: FTIR spectrum of Methyl 2-(methylthio)-5-phenylthiophene-3-carboxylate (**9b**).

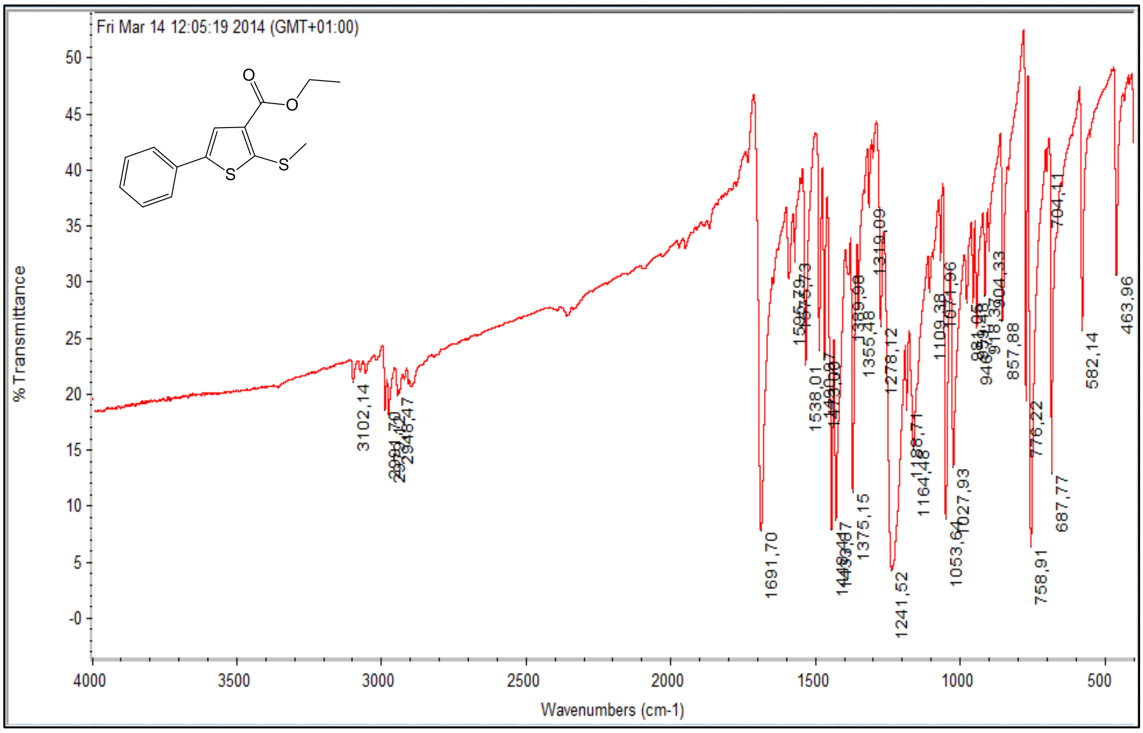

Figure S35: FTIR spectrum of Ethyl 2-(methylthio)-5-phenylthiophene-3-carboxylate (**9c**).

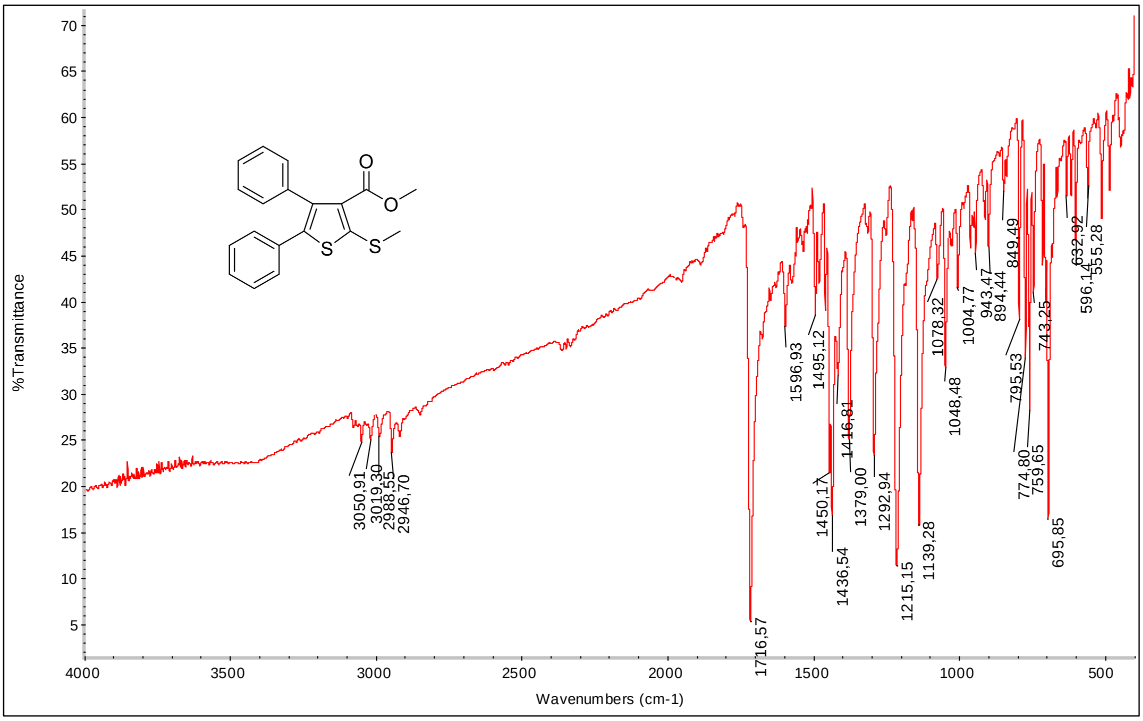

Figure S36: FTIR spectrum of Methyl 2-(methylthio)-4,5-diphenylthiophene-3-carboxylate (**9d**).

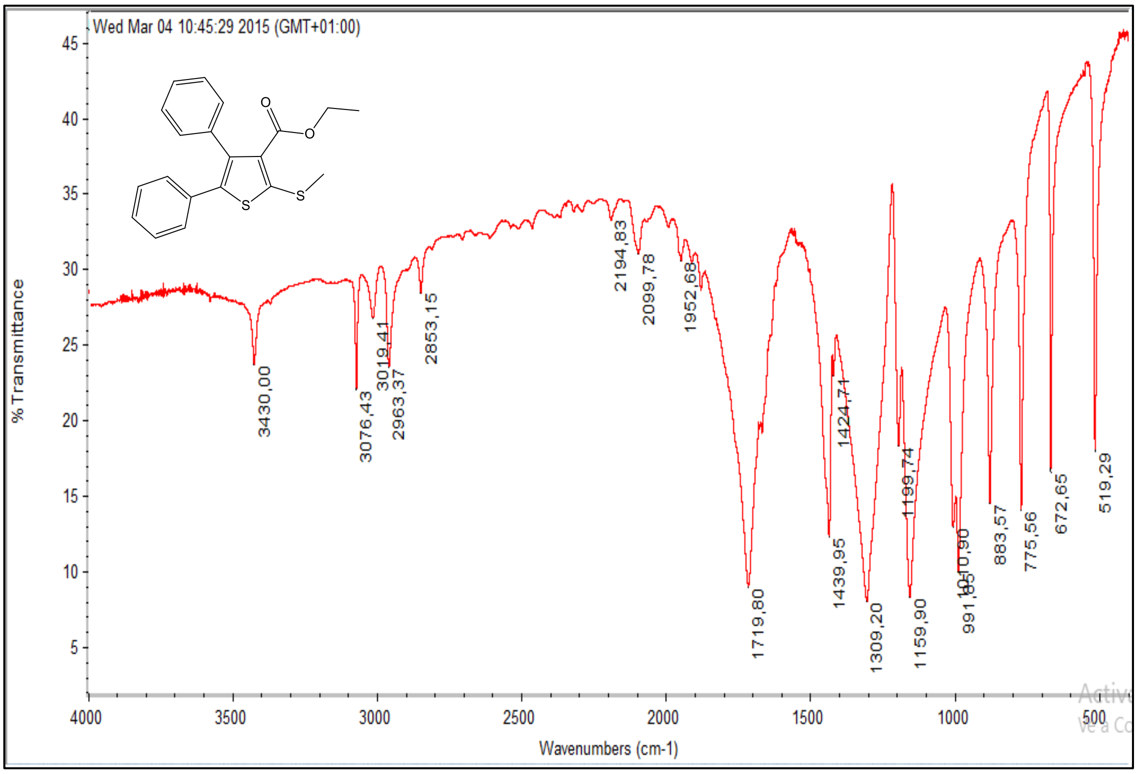

Figure S37: FTIR spectrum of Ethyl 2-(methylthio)-4,5-diphenylthiophene-3-carboxylate (**9e**).

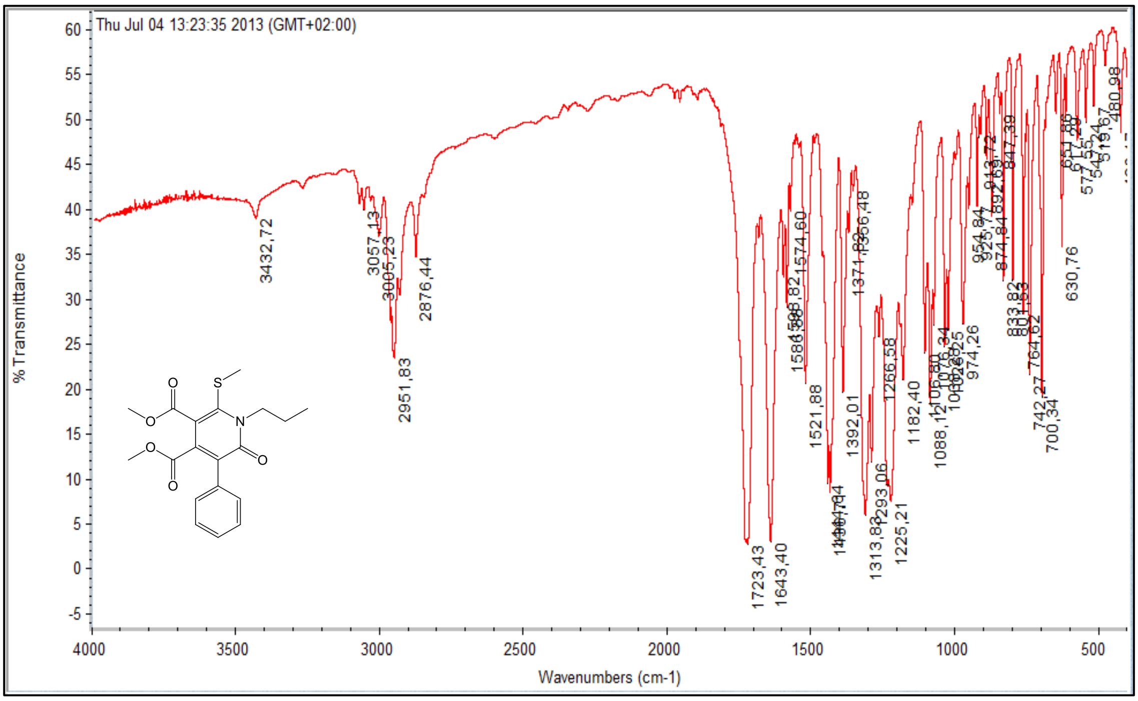

Figure S38: FTIR spectrum of Dimethyl 2-(methylthio)-6-oxo-5-phenyl-1-propyl-1,6-dihydropyridine-3,4-dicarboxylate (**10a**).

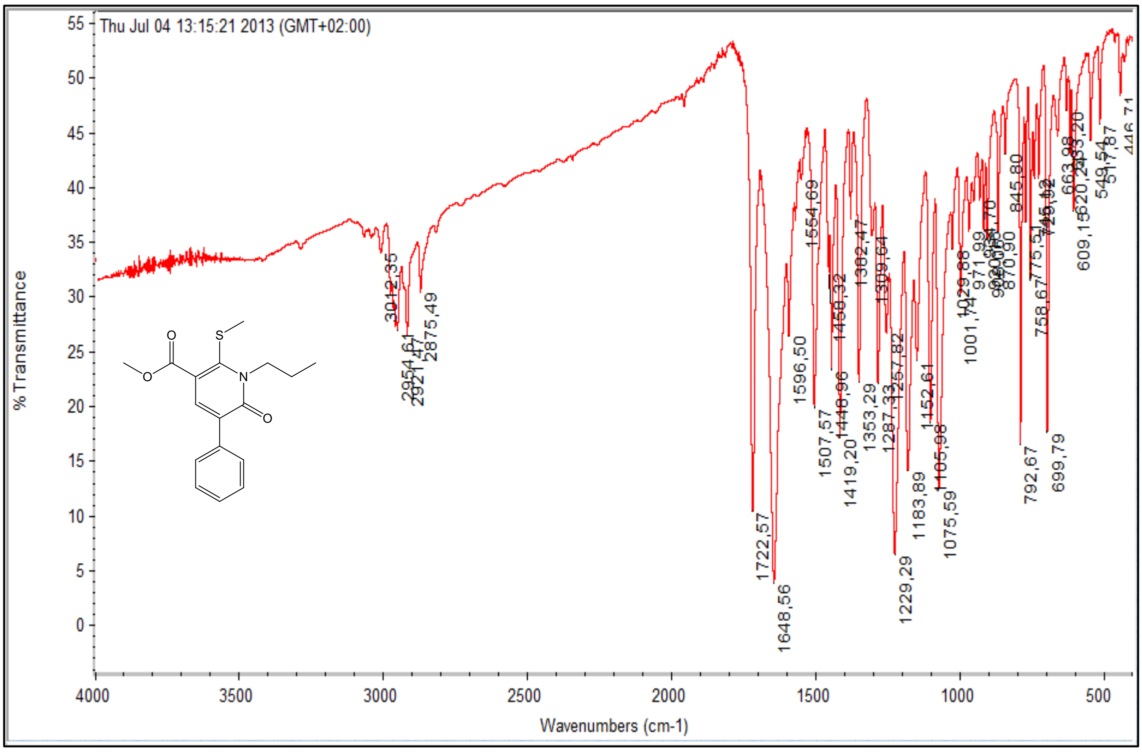

Figure S39: FTIR spectrum of Methyl 2-(methylthio)-6-oxo-5-phenyl-1-propyl-1,6-dihydropyridine-3-carboxylate (**10b**).

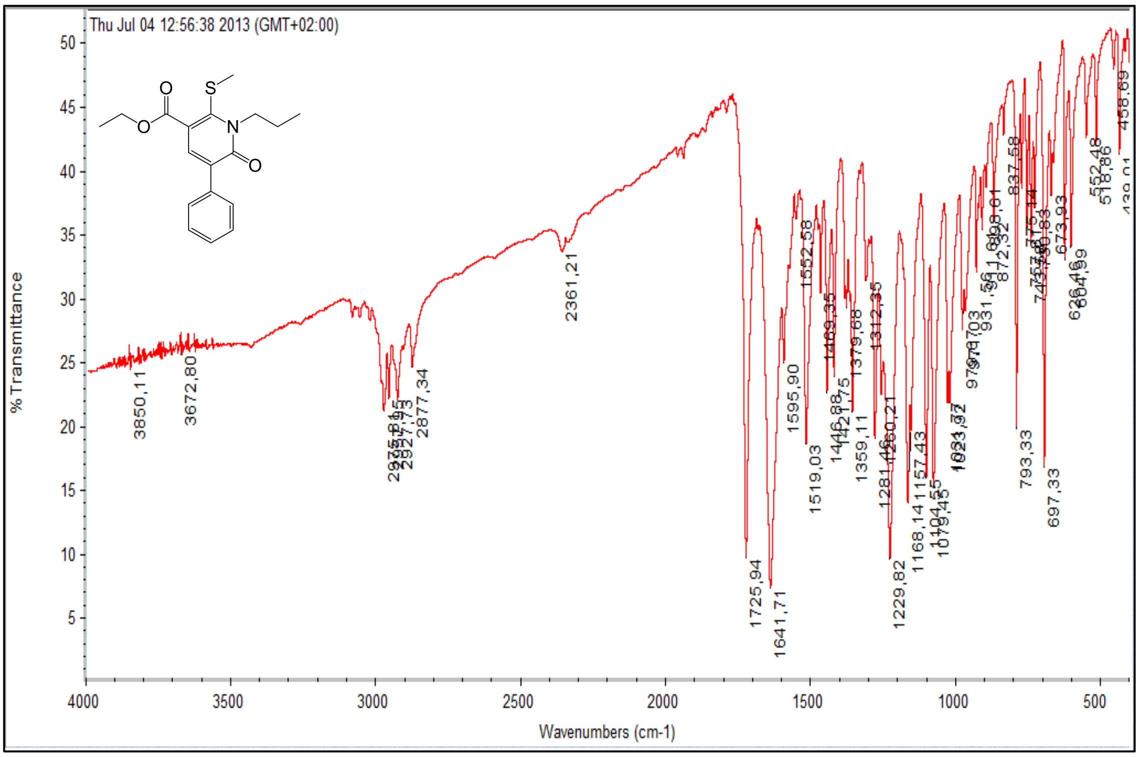

Figure S40: FTIR spectrum of Ethyl 2-(Methylthio)-6-oxo-5-phenyl-1-propyl-1,6-dihydropyridine-3-carboxylate (**10c**).

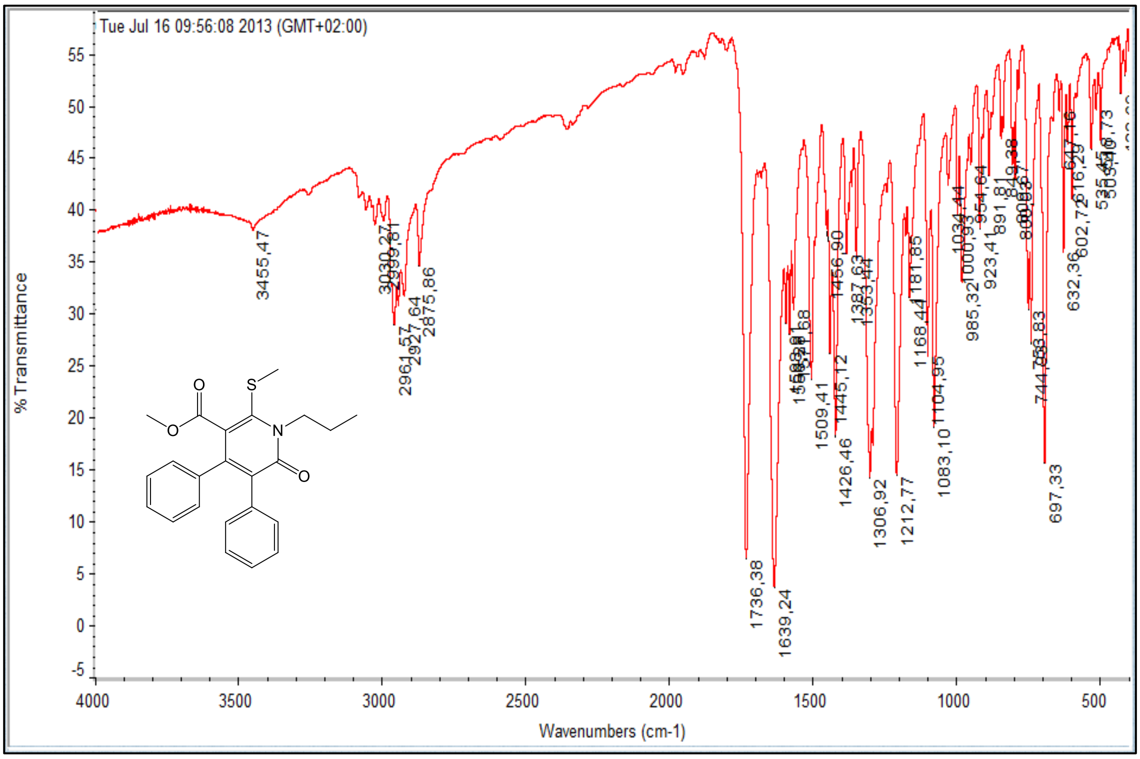

Figure S41: FTIR spectrum of Methyl 2-(methylthio)-6-oxo-4,5-diphenyl-1-propyl-1,6-dihydropyridine-3-carboxylate (**10d**).

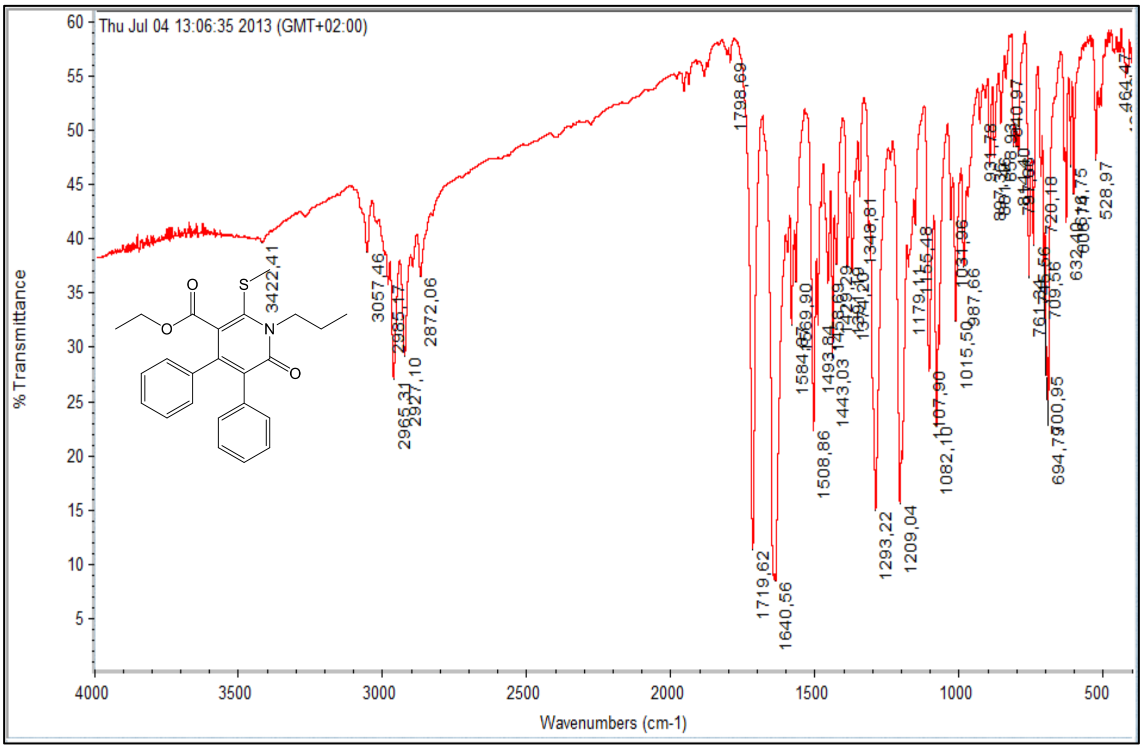

Figure S42: FTIR spectrum of Ethyl 2-(methylthio)-6-oxo-4,5-diphenyl-1-propyl-1,6-dihydropyridine-3-carboxylate (**10e**).

III. Two-dimensional NMR spectra.

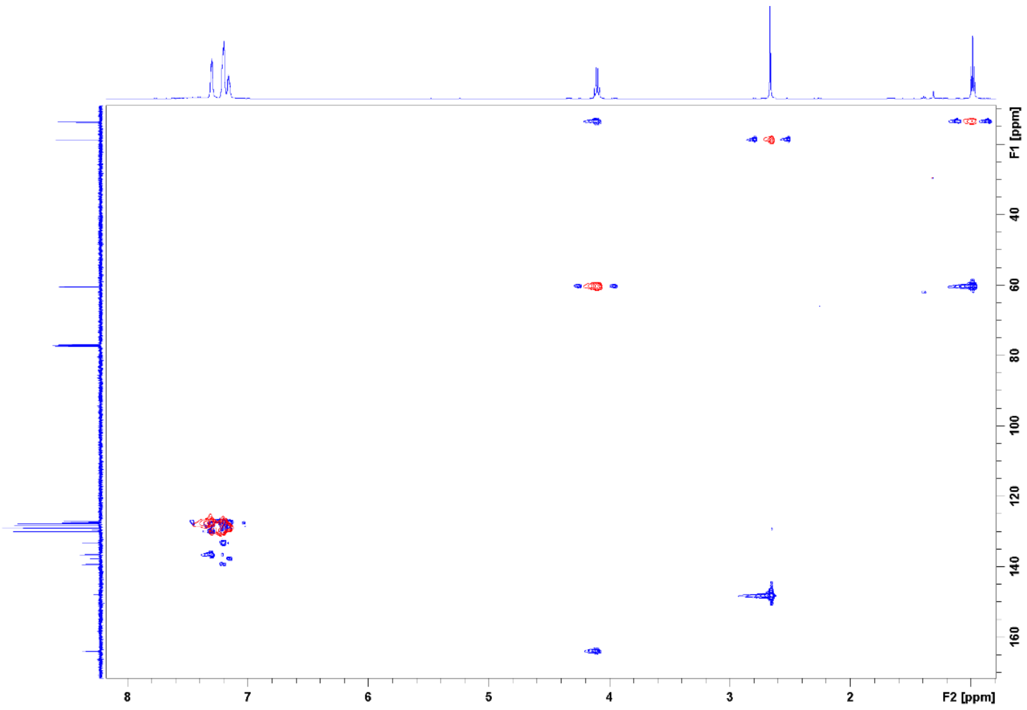

Figure S43: Heteronuclear HMBC spectrum of Ethyl 2-(methylthio)-4,5-diphenylthiophene-3-carboxylate (**9e**).

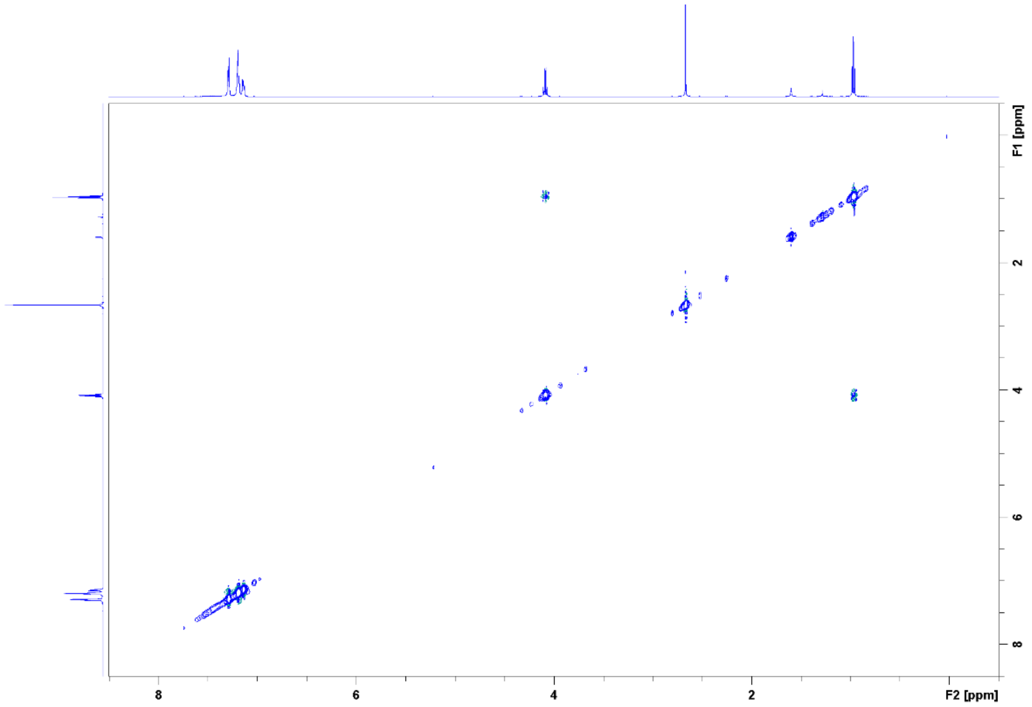

Figure S44: Heteronuclear HMQC spectrum of Ethyl 2-(methylthio)-4,5-diphenylthiophene-3-carboxylate (**9e**).

IV. Computational Data and Cartesian Coordinates for Optimized Structures.

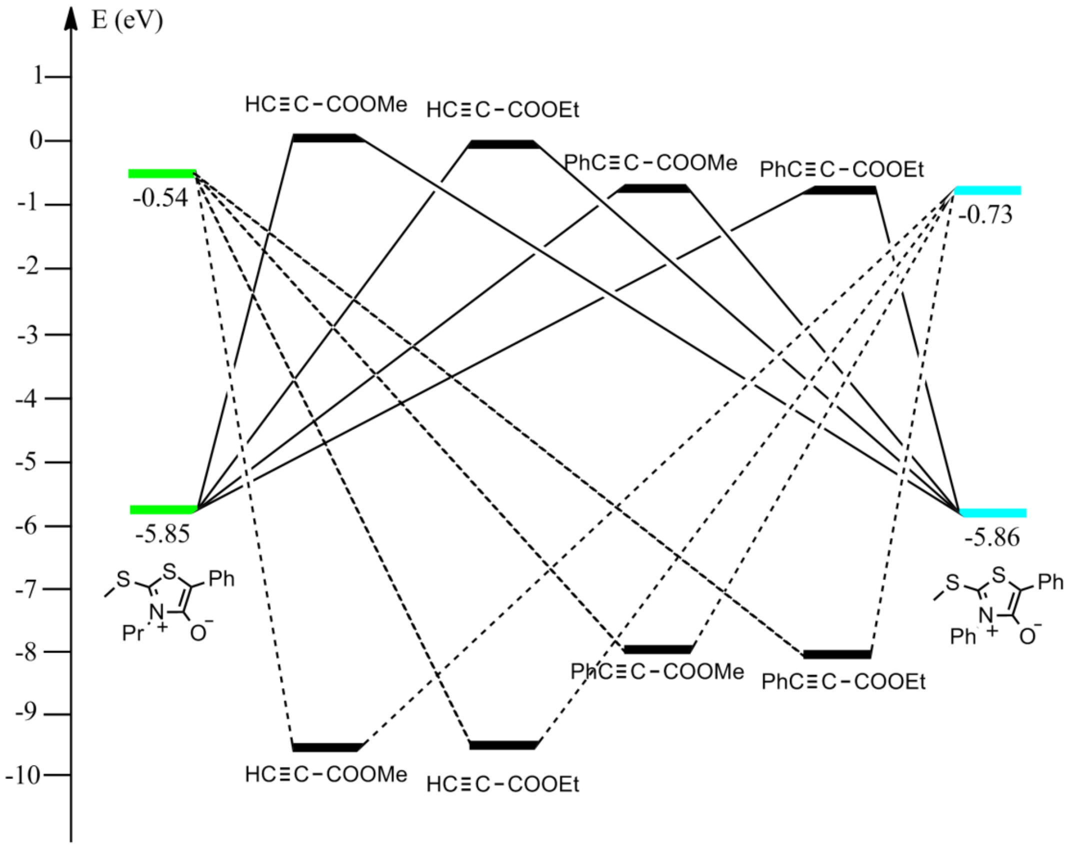

Figure S45: Comparative energy correlations for FMO interactions involving mesoionic dipoles **5** and **6** with substituted acetylenes (at the M06-2X/6-311++G(d,p) level).

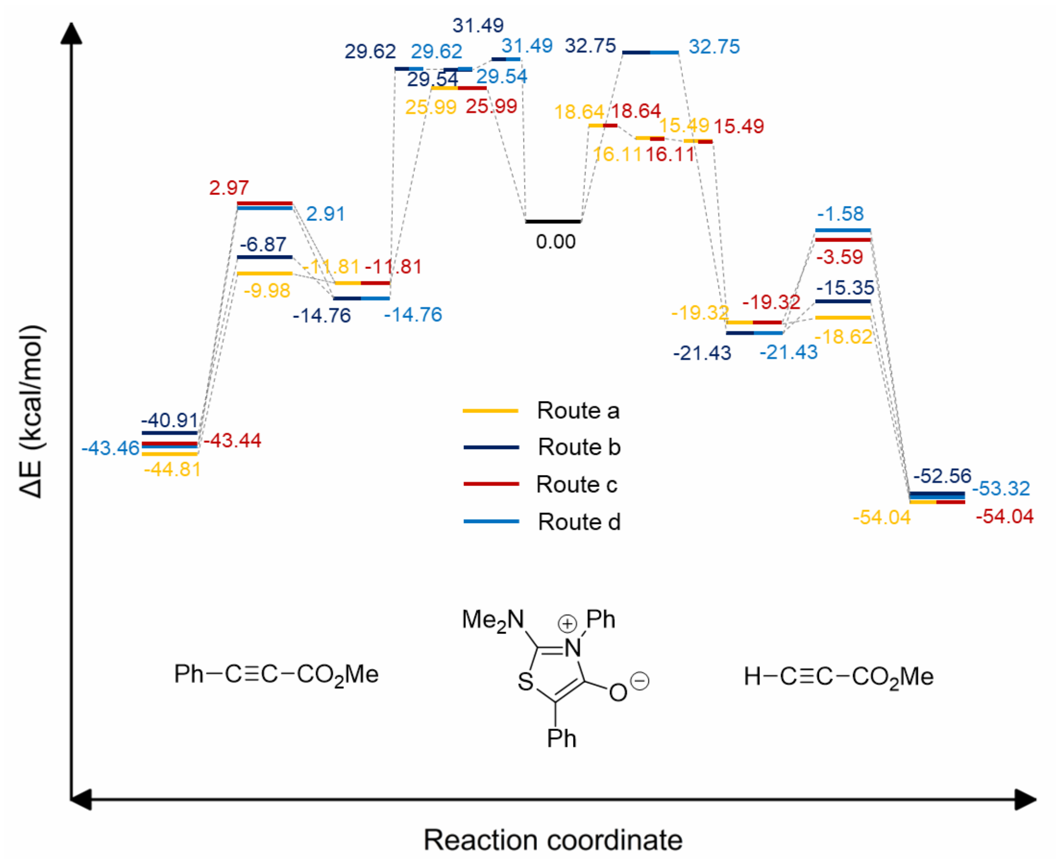

Figure S46: Energy profiles calculated for the cycloadditions of 2-dimethylamino-5-phenyl-3-propylthiazol-3-ium-4-olate (**11**) against methyl propiolate and methyl phenyl propiolate, at the M06-2X/6-311++G(d,p) level.

Table S1. Gibbs Energy (kcal/mol) for Transition Structures in Cycloadduct Formation from Dipoles 5, 6, and 11

Mesoionic Ring	Route *a* HC≡C−COOMe	Route *a* Ph−C≡C−COOMe
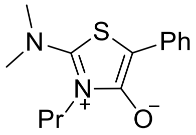 **11**	18.64	25.99
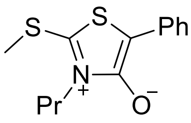 **5**	26.66	27.16
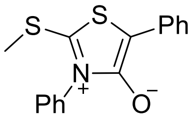 **6**	25.65	26.05

Lists of Cartesian Coordinates

Routes for the Dipolar Cycloaddition of Mesoionic **5** and Methyl Propiolate **7b**

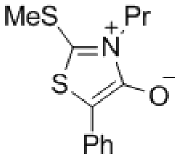

2-(Methylthio)-5-phenyl-3-propylthiazol-3-ium-4-olate (**5**)

Electronic Energy (EE): -1430.66701805

Standard orientation:

Center Number	Atomic Number	Atomic Type	X	Y	Z
1	6	0	1.410885	0.808400	-0.187367
2	7	0	1.309560	-0.493101	-0.380800
3	6	0	-0.042680	-1.039750	-0.344876
4	6	0	-0.939248	0.015051	-0.118152
5	16	0	-0.107347	1.540078	0.039746
6	6	0	-2.386987	-0.068069	-0.004724
7	6	0	2.443457	-1.401106	-0.546768
8	16	0	2.942213	1.636998	-0.179986
9	8	0	-0.201614	-2.249501	-0.505184
10	6	0	2.393112	3.342167	0.085642
11	6	0	-3.167015	1.054692	0.323089
12	6	0	-4.547885	0.963862	0.427453
13	6	0	-5.194046	-0.249661	0.210165
14	6	0	-4.433214	-1.369528	-0.113712
15	6	0	-3.050810	-1.289194	-0.223001
16	1	0	3.223431	-0.884626	-1.112216
17	1	0	2.072795	-2.235515	-1.142728
18	1	0	3.305237	3.938105	0.096059
19	1	0	1.889329	3.442995	1.046700
20	1	0	1.756482	3.676980	-0.732981
21	1	0	-2.695074	2.015455	0.504667
22	1	0	-5.121453	1.848227	0.682111
23	1	0	-6.272154	-0.320409	0.293218
24	1	0	-4.920779	-2.322929	-0.285467
25	1	0	-2.468881	-2.165668	-0.474077
26	6	0	2.959150	-1.893709	0.802810
27	1	0	2.142015	-2.409523	1.313466
28	1	0	3.240827	-1.033786	1.418355
29	6	0	4.149995	-2.827779	0.623824
30	1	0	4.510735	-3.187067	1.589198
31	1	0	3.877491	-3.699956	0.023854
32	1	0	4.979857	-2.320043	0.124449

H-C≡C-COOMe

Methyl propiolate (**7b**)

Electronic Energy (EE): -305.175591563

Standard orientation:

Center Number	Atomic Number	Atomic Type	X	Y	Z
1	6	0	2.624695	-0.408518	-0.000140
2	6	0	1.464072	-0.110078	-0.000079
3	1	0	3.657998	-0.676014	-0.000040
4	6	0	0.069689	0.305900	0.000072
5	8	0	-0.285732	1.453056	0.000089
6	8	0	-0.746116	-0.744038	-0.000001
7	6	0	-2.146827	-0.433715	0.000023
8	1	0	-2.407703	0.137892	-0.891083
9	1	0	-2.657543	-1.393033	-0.000212
10	1	0	-2.407739	0.137477	0.891376

<u>Route a</u>

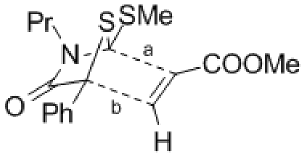

a: 2.373 Angstroms
b: 2.123 Angstroms

Frequency: 443.26i

Electronic Energy (EE): -1735.82333469

Standard orientation:

Center Number	Atomic Number	Atomic Type	Coordinates (Angstroms) X	Y	Z
1	6	0	-0.685483	1.229527	-0.336118
2	6	0	-1.445602	0.102523	0.192807
3	6	0	0.887583	-0.027382	0.818838
4	6	0	-0.618977	-1.107286	-1.342840
5	6	0	0.558674	-1.398193	-1.090653
6	16	0	-0.574595	-0.601721	1.524617
7	16	0	2.423315	-0.445467	1.566138
8	6	0	2.886713	1.129321	2.359390
9	1	0	3.623345	0.867761	3.119823
10	1	0	3.337928	1.817844	1.647766
11	1	0	2.017439	1.574152	2.842247
12	7	0	0.654240	1.095394	0.083478
13	8	0	-1.036326	2.085147	-1.131085
14	6	0	-2.911130	-0.035809	0.107800
15	6	0	-3.727765	1.044216	-0.243670
16	6	0	-3.499042	-1.282161	0.360540
17	6	0	-5.106557	0.877800	-0.319337
18	1	0	-3.278841	2.004610	-0.458732
19	6	0	-4.876375	-1.439361	0.289114
20	1	0	-2.868963	-2.132954	0.602156
21	6	0	-5.685438	-0.357934	-0.051168
22	1	0	-5.730044	1.722180	-0.589975
23	1	0	-5.318425	-2.408710	0.488230
24	1	0	-6.760408	-0.481712	-0.112475
25	6	0	1.680564	1.817845	-0.675817
26	6	0	1.763125	3.289813	-0.283888
27	1	0	1.413858	1.724001	-1.732865
28	1	0	2.630577	1.302487	-0.524586
29	6	0	2.863181	3.992212	-1.072924
30	1	0	0.797358	3.758654	-0.481129
31	1	0	1.951649	3.373117	0.790167
32	1	0	2.925592	5.047601	-0.801279
33	1	0	3.840840	3.538075	-0.886572
34	1	0	2.669456	3.935875	-2.147205
35	1	0	-1.490060	-1.138258	-1.972117
36	6	0	1.899532	-1.896428	-1.248909
37	6	0	3.407729	-3.587248	-0.656871
38	1	0	4.128437	-2.880318	-0.244457
39	1	0	3.385168	-4.497023	-0.061559
40	1	0	3.674227	-3.818077	-1.689082
41	8	0	2.771428	-1.327203	-1.861671
42	8	0	2.083306	-3.049292	-0.590063

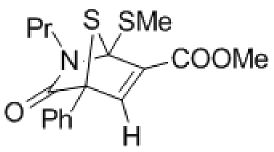

Electronic Energy (EE): -1735.90420771

Standard orientation:

Center Number	Atomic Number	Atomic Type	Coordinates (Angstroms) X	Y	Z
1	6	0	-0.843904	1.364553	0.083774
2	6	0	-1.403750	-0.091577	0.132625
3	6	0	0.835451	-0.058854	0.873912
4	6	0	-0.519503	-0.822700	-0.873525
5	6	0	0.744452	-0.857154	-0.456610
6	16	0	-0.688251	-0.668422	1.747266
7	16	0	2.223942	-0.227997	2.015688
8	6	0	3.705797	0.200787	1.056047
9	1	0	4.536791	-0.157035	1.666635
10	1	0	3.739728	-0.320084	0.102834
11	1	0	3.813536	1.274241	0.914596
12	7	0	0.432294	1.327435	0.566493
13	8	0	-1.421295	2.323023	-0.367348
14	6	0	-2.886510	-0.207730	-0.041171
15	6	0	-3.441350	0.097871	-1.285774
16	6	0	-3.725588	-0.580225	1.005857
17	6	0	-4.815004	0.024660	-1.477731
18	1	0	-2.798005	0.407634	-2.102224
19	6	0	-5.101656	-0.654781	0.812354
20	1	0	-3.314309	-0.812986	1.982768
21	6	0	-5.648759	-0.353337	-0.428888
22	1	0	-5.235318	0.268108	-2.446502
23	1	0	-5.742813	-0.948331	1.635131
24	1	0	-6.720377	-0.409743	-0.580067
25	6	0	1.357587	2.416296	0.293665
26	6	0	2.048846	2.298580	-1.063981
27	1	0	2.076466	2.468986	1.113967
28	1	0	0.765001	3.332920	0.327433
29	6	0	3.062885	3.419964	-1.263378
30	1	0	2.544626	1.326460	-1.152785
31	1	0	1.286252	2.330886	-1.848234
32	1	0	3.544988	3.347387	-2.239951
33	1	0	2.580202	4.398806	-1.198696
34	1	0	3.846317	3.385868	-0.500186
35	1	0	-0.905316	-1.246590	-1.792378
36	6	0	1.790401	-1.602015	-1.204080
37	6	0	3.596623	-3.086429	-1.031751
38	1	0	4.254269	-2.445789	-1.621464
39	1	0	4.148263	-3.564672	-0.226119
40	1	0	3.147225	-3.837370	-1.682262
41	8	0	1.880218	-1.614353	-2.404480
42	8	0	2.578484	-2.308107	-0.392333

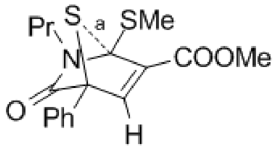

a: 2.714 Angstroms

Frequency: 235.70i

Electronic Energy (EE): -1735.88359978

Standard orientation:

Center Number	Atomic Number	Atomic Type	Coordinates (Angstroms) X	Y	Z
1	6	0	1.118040	-0.049281	0.262748
2	6	0	-1.455161	0.269300	-0.065264
3	16	0	0.440436	0.681294	1.833127
4	16	0	-3.012885	0.742523	0.482146
5	6	0	-3.355654	-0.533905	1.720992
6	1	0	-2.432647	-0.759551	2.257736
7	1	0	-4.067428	-0.078981	2.409747
8	1	0	-3.795975	-1.417040	1.265845
9	6	0	-1.250841	2.444224	-1.240660
10	1	0	-0.728534	2.703663	-2.162172
11	1	0	-2.312739	2.352660	-1.478739
12	7	0	-0.763087	1.107955	-0.862638
13	6	0	0.670708	0.952800	-0.808497
14	8	0	1.371917	1.651460	-1.477345
15	6	0	2.616613	-0.266257	0.185891
16	6	0	3.139516	-0.892861	-0.948899
17	6	0	3.484019	0.141058	1.193452
18	6	0	4.504866	-1.114882	-1.070748
19	1	0	2.473341	-1.200671	-1.750040
20	6	0	4.854110	-0.080628	1.071987
21	1	0	3.080716	0.626938	2.073176
22	6	0	5.367753	-0.708375	-0.055729
23	1	0	4.896329	-1.598862	-1.958084
24	1	0	5.519226	0.240872	1.865212
25	1	0	6.434143	-0.879729	-0.147526
26	6	0	0.330309	-1.309946	0.065309
27	6	0	-0.999275	3.504140	-0.170374
28	1	0	-1.433789	3.185625	0.779881
29	1	0	0.077438	3.596356	-0.008223
30	6	0	-1.588591	4.841186	-0.606980
31	1	0	-1.400067	5.611082	0.143247
32	1	0	-1.147514	5.178338	-1.549134
33	1	0	-2.671011	4.770930	-0.747884
34	6	0	-0.987136	-1.153639	-0.082052
35	1	0	0.817801	-2.278127	0.048150
36	6	0	-1.911592	-2.255447	-0.436796
37	8	0	-3.008808	-2.106237	-0.915091
38	8	0	-1.367430	-3.449539	-0.194641
39	6	0	-2.161086	-4.580237	-0.574898
40	1	0	-2.365312	-4.559094	-1.645842
41	1	0	-1.566548	-5.453381	-0.318145
42	1	0	-3.101658	-4.586771	-0.023361

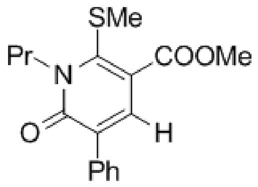

Electronic Energy (EE): -1337.76456980

Standard orientation:

Center Number	Atomic Number	Atomic Type	Coordinates (Angstroms) X	Y	Z
1	6	0	1.321044	-0.050326	0.107338
2	6	0	0.625066	1.109712	-0.030411
3	6	0	-0.795845	1.158232	-0.028565
4	6	0	-1.497785	-0.019413	0.096522
5	6	0	2.802475	-0.059447	0.122170
6	6	0	3.493895	0.957848	0.789608
7	6	0	3.538020	-1.041367	-0.551036
8	6	0	4.882726	1.005102	0.772017
9	1	0	2.938705	1.707638	1.342722
10	6	0	4.926444	-0.988837	-0.571698
11	1	0	3.019161	-1.843573	-1.058594
12	6	0	5.604405	0.032375	0.087957
13	1	0	5.400404	1.797545	1.300404
14	1	0	5.481160	-1.752695	-1.104503
15	1	0	6.687661	0.065280	0.074642
16	16	0	-3.276959	-0.088196	-0.071774
17	6	0	-3.755181	0.262585	1.648455
18	1	0	-3.419952	1.259865	1.923201
19	1	0	-4.844787	0.223175	1.671063
20	1	0	-3.354663	-0.491589	2.326113
21	8	0	1.113975	-2.404829	0.337183
22	6	0	0.594043	-1.305616	0.235314
23	6	0	-1.519627	-2.490409	0.405181
24	6	0	-1.732373	-3.182433	-0.935780
25	1	0	-2.469569	-2.312044	0.903104
26	1	0	-0.892735	-3.105197	1.048474
27	6	0	-2.424217	-4.527817	-0.745903
28	1	0	-2.335736	-2.535430	-1.579483
29	1	0	-0.761199	-3.319617	-1.417998
30	1	0	-2.575352	-5.028733	-1.703878
31	1	0	-1.826082	-5.189215	-0.113382
32	1	0	-3.404014	-4.403767	-0.275991
33	1	0	1.164220	2.039716	-0.166268
34	6	0	-1.461281	2.482033	-0.165122
35	8	0	-0.708838	3.324427	-0.887203
36	8	0	-2.513212	2.805009	0.323958
37	6	0	-1.226393	4.650742	-1.029602
38	1	0	-1.352519	5.118586	-0.052325
39	1	0	-0.488703	5.192471	-1.616696
40	1	0	-2.184500	4.630516	-1.549671
41	7	0	-0.819720	-1.199490	0.252466

S_8

Electronic Energy (EE): -3185.48856569

Standard orientation:

Center Number	Atomic Number	Atomic Type	Coordinates (Angstroms) X	Y	Z
1	16	0	1.011041	2.026185	0.586692
2	16	0	1.772248	0.024772	1.111822
3	16	0	2.026455	-1.011021	-0.586623
4	16	0	0.024644	-1.771971	-1.111836
5	16	0	-1.010893	-2.026357	0.586623
6	16	0	-1.772462	-0.024685	1.111803
7	16	0	-2.026438	1.010937	-0.586679
8	16	0	-0.024594	1.772141	-1.111802

Route c

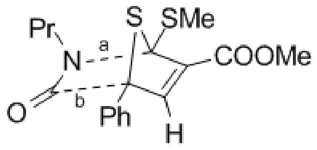

a: 2.155 Angstroms
b: 2.909 Angstroms

Frequency: 578.91i

Electronic Energy (EE): -1735.85296131

Standard orientation:

Center Number	Atomic Number	Atomic Type	Coordinates (Angstroms) X	Y	Z
1	6	0	-0.688536	1.350951	-0.450687
2	6	0	-1.371841	-0.327389	0.148326
3	6	0	0.906334	-0.456926	0.958007
4	6	0	-0.545812	-1.166564	-0.670704
5	6	0	0.735187	-1.282360	-0.214854
6	16	0	-0.628647	-0.293544	1.792877
7	16	0	2.336097	-0.146890	1.902375
8	6	0	3.585749	0.153360	0.613611
9	1	0	4.134957	1.047801	0.908548
10	1	0	4.261913	-0.693239	0.532865
11	1	0	3.082663	0.335746	-0.336229
12	7	0	0.550777	1.346191	-0.166385
13	8	0	-1.557115	2.045294	-0.907961
14	6	0	-2.850711	-0.337388	0.034325
15	6	0	-3.431099	-0.273334	-1.235174
16	6	0	-3.678994	-0.368584	1.156386
17	6	0	-4.812320	-0.264685	-1.376768
18	1	0	-2.798867	-0.208107	-2.113612
19	6	0	-5.061898	-0.356147	1.013189
20	1	0	-3.253440	-0.412445	2.153509
21	6	0	-5.633170	-0.307672	-0.253285
22	1	0	-5.248391	-0.211762	-2.367450
23	1	0	-5.691167	-0.385138	1.895081
24	1	0	-6.710923	-0.296673	-0.365360
25	6	0	1.238331	2.600037	0.104880
26	6	0	2.005860	3.107034	-1.114158
27	1	0	1.928684	2.440115	0.942196
28	1	0	0.513892	3.359314	0.425111
29	6	0	2.759838	4.394629	-0.798358
30	1	0	2.701785	2.333114	-1.451954
31	1	0	1.295865	3.272361	-1.929339
32	1	0	3.296721	4.763079	-1.674872
33	1	0	2.074101	5.181555	-0.472987
34	1	0	3.491167	4.238651	0.000331
35	1	0	-0.889931	-1.589849	-1.607523
36	6	0	1.739020	-2.065040	-0.968125
37	6	0	3.768834	-3.232298	-0.866820
38	1	0	4.217283	-2.653575	-1.675974
39	1	0	4.511090	-3.463023	-0.106052
40	1	0	3.345039	-4.152117	-1.270761
41	8	0	1.619862	-2.366485	-2.128724
42	8	0	2.759040	-2.464804	-0.203765

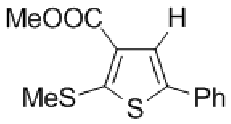

Electronic Energy (EE): -1449.35350206

Standard orientation:

Center Number	Atomic Number	Atomic Type	Coordinates (Angstroms) X	Y	Z
1	6	0	-1.094283	-0.115070	0.012303
2	6	0	-0.180012	-1.119798	0.073693
3	6	0	1.174566	-0.669929	0.040580
4	6	0	1.282839	0.704119	-0.043904
5	16	0	-0.289082	1.429429	-0.093645
6	1	0	-0.436353	-2.167231	0.164035
7	6	0	2.279788	-1.641716	0.107985
8	8	0	2.127562	-2.836375	0.184148
9	8	0	3.481575	-1.054113	0.073921
10	6	0	4.609833	-1.930433	0.132104
11	1	0	4.602505	-2.498281	1.063075
12	1	0	4.601788	-2.618490	-0.713701
13	1	0	5.485089	-1.286650	0.089026
14	6	0	-2.561490	-0.198900	0.013312
15	6	0	-3.348790	0.821876	0.556483
16	6	0	-3.194547	-1.321096	-0.533491
17	6	0	-4.734828	0.722103	0.554021
18	1	0	-2.875461	1.690712	1.001531
19	6	0	-4.579466	-1.422670	-0.523766
20	1	0	-2.597152	-2.105282	-0.984426
21	6	0	-5.355419	-0.401237	0.017120
22	1	0	-5.330241	1.521078	0.980491
23	1	0	-5.054443	-2.297352	-0.952781
24	1	0	-6.436234	-0.479227	0.017111
25	16	0	2.742323	1.673974	-0.108819
26	6	0	2.048873	3.344296	-0.207334
27	1	0	1.459357	3.588195	0.676968
28	1	0	2.911594	4.010173	-0.245173
29	1	0	1.462218	3.482540	-1.115920

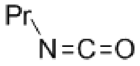

Electronic Energy (EE): -286.578726553

Standard orientation:

Center Number	Atomic Number	Atomic Type	Coordinates (Angstroms) X	Y	Z
1	6	0	-2.716505	-0.374060	0.000117
2	6	0	-1.196734	-0.499271	-0.000222
3	1	0	-3.189289	-1.357697	-0.000878
4	1	0	-3.067130	0.163867	0.884882
5	1	0	-3.067355	0.165739	-0.883422
6	6	0	-0.531553	0.871752	0.000118
7	1	0	-0.862360	-1.055348	0.880835
8	1	0	-0.862689	-1.054747	-0.881778
9	1	0	-0.833285	1.444885	-0.879939
10	1	0	-0.832908	1.444255	0.880714
11	7	0	0.914291	0.794486	-0.000199
12	6	0	1.810835	0.009918	-0.000027
13	8	0	2.764840	-0.670300	0.000133

Route b

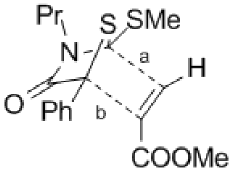

a: 2.146 Angstroms
b: 2.440 Angstroms

Frequency: 453.78i

Electronic Energy (EE): -1735.81930205

Standard orientation:

Center Number	Atomic Number	Atomic Type	Coordinates (Angstroms) X	Y	Z
1	6	0	-0.300182	0.413803	-1.168896
2	6	0	0.580650	-0.601216	-0.590937
3	6	0	-1.758747	-1.081227	-0.128161
4	6	0	-0.084100	0.284180	1.583098
5	6	0	-1.234932	-0.140452	1.727989
6	16	0	-0.309394	-2.033123	-0.239681
7	16	0	-3.269008	-2.031070	-0.120089
8	6	0	-4.305961	-1.188906	1.114678
9	1	0	-5.303771	-1.604875	0.968959
10	1	0	-3.984476	-1.411448	2.130511
11	1	0	-4.349466	-0.115021	0.939311
12	7	0	-1.621741	0.022838	-0.944004
13	8	0	-0.007504	1.460165	-1.726797
14	6	0	2.041783	-0.605224	-0.694420
15	6	0	2.710323	0.265574	-1.568594
16	6	0	2.798649	-1.463240	0.118393
17	6	0	4.097752	0.248954	-1.641429
18	1	0	2.134363	0.947440	-2.178506
19	6	0	4.184021	-1.471999	0.039686
20	1	0	2.299769	-2.107597	0.834480
21	6	0	4.839171	-0.617900	-0.843107
22	1	0	4.602115	0.920569	-2.326873
23	1	0	4.752652	-2.137220	0.678791
24	1	0	5.921442	-0.622896	-0.902417
25	6	0	-2.690476	0.974829	-1.229226
26	6	0	-2.807065	2.078088	-0.178588
27	1	0	-3.622158	0.414436	-1.332337
28	1	0	-2.450492	1.416776	-2.197889
29	6	0	-3.889453	3.076782	-0.573385
30	1	0	-3.033023	1.642978	0.799072
31	1	0	-1.838313	2.575757	-0.085301
32	1	0	-3.987745	3.866019	0.174107
33	1	0	-3.653956	3.550790	-1.529856
34	1	0	-4.863613	2.588355	-0.673856
35	1	0	-2.113653	-0.258540	2.327861
36	6	0	1.246614	0.780257	1.866329
37	8	0	2.000481	0.240789	2.638474
38	8	0	1.550339	1.858555	1.145982
39	6	0	2.910414	2.295862	1.248397
40	1	0	3.589541	1.467454	1.039753
41	1	0	3.022543	3.077161	0.500363
42	1	0	3.107556	2.692039	2.245921

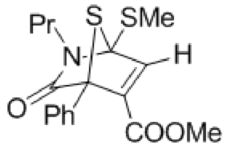

Electronic Energy (EE): -1735.90599351

Standard orientation:

Center Number	Atomic Number	Atomic Type	Coordinates (Angstroms) X	Y	Z
1	6	0	0.127166	-0.929583	1.067373
2	6	0	-0.791817	-0.518010	-0.134428
3	6	0	1.458050	-0.699181	-0.830824
4	6	0	-0.242017	0.874762	-0.514822
5	6	0	1.021073	0.764100	-0.929993
6	16	0	-0.041986	-1.567899	-1.477275
7	16	0	2.913006	-1.247834	-1.743140
8	6	0	4.158175	0.013095	-1.344454
9	1	0	5.006992	-0.219413	-1.989315
10	1	0	3.812595	1.016155	-1.591553
11	1	0	4.485629	-0.040221	-0.307625
12	7	0	1.403133	-1.073760	0.593824
13	8	0	-0.215518	-1.032338	2.219567
14	6	0	-2.264949	-0.715706	0.090232
15	6	0	-2.864730	-0.208637	1.245564
16	6	0	-3.050127	-1.397280	-0.836199
17	6	0	-4.223097	-0.386928	1.464873
18	1	0	-2.269125	0.331873	1.968841
19	6	0	-4.413016	-1.576221	-0.615492
20	1	0	-2.610303	-1.790984	-1.746639
21	6	0	-5.002270	-1.072238	0.536429
22	1	0	-4.675439	0.014137	2.364342
23	1	0	-5.008190	-2.110189	-1.346879
24	1	0	-6.062918	-1.210826	0.711822
25	6	0	2.535962	-0.975006	1.503570
26	6	0	2.864081	0.461613	1.907430
27	1	0	3.391198	-1.467547	1.034770
28	1	0	2.271840	-1.558184	2.388328
29	6	0	4.071168	0.508817	2.837794
30	1	0	3.056747	1.065636	1.014419
31	1	0	1.988129	0.895840	2.398774
32	1	0	4.310247	1.534556	3.124825
33	1	0	3.880685	-0.059103	3.752079
34	1	0	4.956882	0.080225	2.358818
35	1	0	1.662928	1.575252	-1.244933
36	6	0	-0.990976	2.153628	-0.448074
37	8	0	-2.173237	2.263429	-0.263243
38	8	0	-0.176247	3.199933	-0.648521
39	6	0	-0.817543	4.479649	-0.662236
40	1	0	-1.299622	4.673158	0.296522
41	1	0	-0.026136	5.203633	-0.840957
42	1	0	-1.561168	4.524611	-1.458648

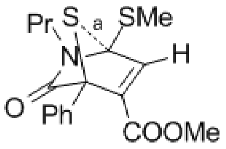

a: 2.721 Angstroms

Frequency: 228.43i

Electronic Energy (EE): -1735.87988700

Standard orientation:

Center Number	Atomic Number	Atomic Type	Coordinates (Angstroms) X	Y	Z
1	6	0	-0.451147	-0.260282	0.160196
2	6	0	1.859256	0.860948	-0.323017
3	16	0	0.468171	-0.164283	1.778502
4	16	0	3.393933	1.536017	0.075159
5	6	0	2.879927	3.083949	0.864934
6	1	0	3.767021	3.451541	1.379923
7	1	0	2.557235	3.822118	0.133242
8	1	0	2.097008	2.870698	1.593245
9	6	0	2.983530	-1.233900	-1.026527
10	1	0	2.733282	-1.939325	-1.819501
11	1	0	3.806027	-0.611441	-1.386869
12	7	0	1.804932	-0.369354	-0.864463
13	6	0	0.534452	-1.037209	-0.722413
14	8	0	0.388134	-2.132171	-1.178339
15	6	0	-1.791655	-0.972924	0.161273
16	6	0	-2.495029	-1.069501	-1.042973
17	6	0	-2.347685	-1.511585	1.314430
18	6	0	-3.740136	-1.681860	-1.088341
19	1	0	-2.066374	-0.664969	-1.956425
20	6	0	-3.594361	-2.132244	1.269287
21	1	0	-1.805326	-1.434210	2.248601
22	6	0	-4.295197	-2.216131	0.072659
23	1	0	-4.273809	-1.748801	-2.029598
24	1	0	-4.016391	-2.548991	2.176801
25	1	0	-5.265251	-2.698862	0.039579
26	6	0	-0.506347	1.162370	-0.362072
27	6	0	3.358208	-1.979341	0.252191
28	1	0	3.514827	-1.266986	1.065712
29	1	0	2.520691	-2.615357	0.548530
30	6	0	4.612139	-2.816816	0.025834
31	1	0	4.874285	-3.372945	0.927406
32	1	0	4.463386	-3.540385	-0.780323
33	1	0	5.467216	-2.188195	-0.238886
34	6	0	0.679279	1.728538	-0.558391
35	1	0	0.822441	2.751094	-0.881890
36	6	0	-1.754348	1.966070	-0.552152
37	8	0	-1.906964	2.748858	-1.454819
38	8	0	-2.634617	1.741757	0.411124
39	6	0	-3.894175	2.411267	0.282282
40	1	0	-4.473597	2.111944	1.151869
41	1	0	-3.752106	3.492250	0.269385
42	1	0	-4.391450	2.092908	-0.635050

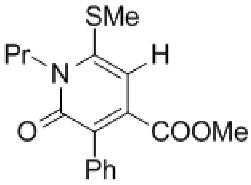

Electronic Energy (EE): -1337.76315074

Standard orientation:

Center Number	Atomic Number	Atomic Type	Coordinates (Angstroms) X	Y	Z
1	6	0	-0.646175	0.244069	0.109823
2	6	0	-0.499770	-1.092246	-0.130838
3	6	0	0.781145	-1.702762	-0.185964
4	6	0	1.892473	-0.936474	-0.019677
5	1	0	0.877120	-2.765027	-0.358253
6	6	0	-1.967942	0.889496	0.289273
7	6	0	-2.897186	0.330344	1.171523
8	6	0	-2.313035	2.043518	-0.418406
9	6	0	-4.156022	0.899912	1.328369
10	1	0	-2.626252	-0.546201	1.752372
11	6	0	-3.573757	2.607029	-0.265773
12	1	0	-1.593349	2.493263	-1.090524
13	6	0	-4.498409	2.038528	0.606340
14	1	0	-4.863329	0.460626	2.022562
15	1	0	-3.833493	3.497264	-0.827000
16	1	0	-5.477739	2.486987	0.728991
17	16	0	3.500095	-1.706052	-0.097559
18	6	0	3.782131	-1.937160	1.688523
19	1	0	3.033288	-2.610946	2.102445
20	1	0	4.770676	-2.386338	1.790108
21	1	0	3.766388	-0.984076	2.217400
22	8	0	0.511425	2.298184	0.373876
23	6	0	-1.675873	-2.005527	-0.332415
24	8	0	-1.821776	-3.037098	0.270247
25	8	0	-2.499045	-1.564746	-1.277153
26	6	0	-3.693522	-2.330953	-1.469093
27	1	0	-4.234221	-1.835298	-2.271270
28	1	0	-3.449373	-3.356377	-1.747965
29	1	0	-4.288182	-2.331173	-0.554286
30	6	0	0.546350	1.084680	0.236178
31	6	0	2.967396	1.286753	0.308790
32	6	0	3.402927	1.810733	-1.055262
33	1	0	3.772242	0.723741	0.775900
34	1	0	2.689543	2.112932	0.961107
35	6	0	4.621600	2.717246	-0.926190
36	1	0	3.632034	0.959483	-1.703730
37	1	0	2.569059	2.355219	-1.505830
38	1	0	4.941075	3.086095	-1.902575
39	1	0	4.400563	3.584002	-0.297483
40	1	0	5.464702	2.182152	-0.479917
41	7	0	1.784132	0.416669	0.200275

Route d

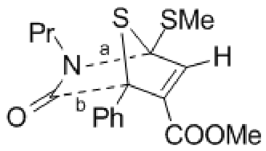

a: 2.089 Angstroms
b: 1.992 Angstroms

Frequency: 627.93i

Electronic Energy (EE): -1735.85055388

Standard orientation:

Center Number	Atomic Number	Atomic Type	Coordinates (Angstroms) X	Y	Z
1	6	0	0.351447	-0.158687	1.530705
2	6	0	-0.723455	-0.585878	-0.091517
3	6	0	1.537114	-1.062877	-0.809126
4	6	0	-0.174337	0.458736	-0.928317
5	6	0	1.105493	0.209025	-1.320798
6	16	0	0.155317	-2.094588	-0.524298
7	16	0	3.076318	-1.829507	-1.096399
8	6	0	4.031328	-0.465616	-1.814280
9	1	0	5.034375	-0.864016	-1.968923
10	1	0	3.618892	-0.157704	-2.774059
11	1	0	4.088494	0.374716	-1.122585
12	7	0	1.525237	-0.452928	1.189088
13	8	0	-0.343669	0.242251	2.424193
14	6	0	-2.154519	-0.687766	0.282454
15	6	0	-2.785145	0.397887	0.900013
16	6	0	-2.889541	-1.853535	0.059816
17	6	0	-4.121904	0.320588	1.266854
18	1	0	-2.221708	1.298321	1.114177
19	6	0	-4.226991	-1.931604	0.432608
20	1	0	-2.427525	-2.707966	-0.423460
21	6	0	-4.848399	-0.844059	1.035212
22	1	0	-4.594325	1.169328	1.747968
23	1	0	-4.781261	-2.843996	0.245759
24	1	0	-5.890390	-0.904085	1.327616
25	6	0	2.724183	0.086445	1.802456
26	6	0	2.942562	1.561533	1.474155
27	1	0	3.577027	-0.510735	1.463154
28	1	0	2.646790	-0.047735	2.888773
29	6	0	4.237458	2.082762	2.087533
30	1	0	2.959592	1.691741	0.387347
31	1	0	2.087179	2.136684	1.841726
32	1	0	4.385638	3.140070	1.859660
33	1	0	4.226962	1.974839	3.175495
34	1	0	5.104777	1.534220	1.707667
35	1	0	1.758887	0.924709	-1.798354
36	6	0	-0.823045	1.775460	-1.214278
37	8	0	-0.285008	2.838790	-1.044618
38	8	0	-2.045116	1.622445	-1.712152
39	6	0	-2.786716	2.827248	-1.932824
40	1	0	-2.290705	3.449125	-2.678640
41	1	0	-3.762840	2.508711	-2.289520
42	1	0	-2.885530	3.383923	-0.999650

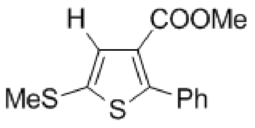

Electronic Energy (EE): -1449.34901908

Standard orientation:

Center Number	Atomic Number	Atomic Type	Coordinates (Angstroms) X	Y	Z
1	6	0	-2.326906	-0.549206	0.034632
2	6	0	-1.866484	0.732425	0.038862
3	6	0	-0.436819	0.808009	0.016356
4	6	0	0.179086	-0.418382	0.009487
5	16	0	-1.010771	-1.682355	0.029100
6	1	0	-2.489896	1.614896	0.039944
7	16	0	-3.974048	-1.160440	0.034247
8	6	0	-4.876568	0.407023	0.043947
9	1	0	-5.934393	0.144220	0.045928
10	1	0	-4.648289	0.979885	0.942751
11	1	0	-4.653668	0.987404	-0.851434
12	6	0	0.211165	2.140441	-0.048379
13	8	0	-0.376614	3.171870	0.168167
14	8	0	1.498003	2.087495	-0.396032
15	6	0	2.177308	3.343649	-0.469721
16	1	0	1.735007	3.970131	-1.245357
17	1	0	2.126575	3.863124	0.487929
18	1	0	3.208687	3.104104	-0.718211
19	6	0	1.602819	-0.806848	0.047559
20	6	0	2.116473	-1.696879	-0.898527
21	6	0	2.441210	-0.322792	1.056118
22	6	0	3.451148	-2.085349	-0.845328
23	1	0	1.473051	-2.069592	-1.688027
24	6	0	3.772061	-0.713012	1.107319
25	1	0	2.040294	0.355926	1.800251
26	6	0	4.281229	-1.594762	0.156000
27	1	0	3.840756	-2.770815	-1.588949
28	1	0	4.413116	-0.335575	1.895592
29	1	0	5.320418	-1.899821	0.199007

Routes for the Dipolar Cycloaddition of Mesoionic **5** and Methyl Phenyl Propiolate **7d**

Ph−C≡C−COOMe

Electronic Energy (EE): -536.207451494

Standard orientation:

Center Number	Atomic Number	Atomic Type	Coordinates (Angstroms) X	Y	Z
1	6	0	-0.001921	0.113069	-0.000006
2	6	0	1.199704	0.194955	0.000123
3	6	0	2.636395	0.373174	0.000305
4	8	0	3.180887	1.445867	0.000372
5	8	0	3.275078	-0.797320	-0.000116
6	6	0	4.706501	-0.715875	-0.000085
7	1	0	5.056009	-0.193431	0.890934
8	1	0	5.056357	-1.744811	-0.000257
9	1	0	5.056021	-0.193140	-0.890931
10	6	0	-1.428571	0.020539	-0.000050
11	6	0	-2.203734	1.187437	-0.000154
12	6	0	-2.052749	-1.232815	0.000019
13	6	0	-3.588433	1.095833	-0.000200
14	1	0	-1.711493	2.152519	-0.000204
15	6	0	-3.438258	-1.313675	-0.000027
16	1	0	-1.445317	-2.129975	0.000110
17	6	0	-4.206382	-0.151911	-0.000136
18	1	0	-4.186486	1.999406	-0.000291
19	1	0	-3.920033	-2.284139	0.000023
20	1	0	-5.288092	-0.219191	-0.000171

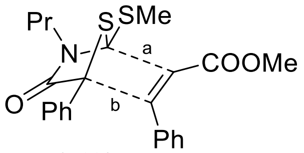

a: 2.118 Angstroms
b: 2.450 Angstroms

Frequency: 426.52i

Electronic Energy (EE): -1966.85528158

Standard orientation:

Center Number	Atomic Number	Atomic Type	Coordinates (Angstroms) X	Y	Z
1	6	0	0.071632	-1.504331	0.152975
2	6	0	-0.654369	-0.823556	-0.907065
3	6	0	1.689289	-0.188916	-0.859249
4	6	0	-0.320693	1.102755	0.569621
5	6	0	0.871630	1.364708	0.325092
6	16	0	0.450686	-0.195574	-2.079710
7	16	0	3.331785	0.262623	-1.358737
8	6	0	4.035240	-1.381081	-1.703765
9	1	0	4.927272	-1.204309	-2.305818
10	1	0	4.319548	-1.890228	-0.784255
11	1	0	3.326880	-1.979001	-2.276262
12	7	0	1.442829	-1.205611	0.033800
13	8	0	-0.366947	-2.169957	1.081758
14	6	0	-2.089469	-0.907633	-1.150324
15	6	0	-2.937691	-1.567039	-0.246356
16	6	0	-2.665028	-0.252970	-2.252264
17	6	0	-4.312858	-1.566795	-0.449578
18	1	0	-2.511063	-2.065426	0.612181
19	6	0	-4.036477	-0.267507	-2.452955
20	1	0	-2.037330	0.285053	-2.955363
21	6	0	-4.869783	-0.922035	-1.548248
22	1	0	-4.952761	-2.073512	0.263918
23	1	0	-4.457888	0.239881	-3.313142
24	1	0	-5.942830	-0.926612	-1.700607
25	6	0	2.300636	-1.481879	1.189749
26	6	0	2.633456	-2.965325	1.322884
27	1	0	1.772464	-1.130273	2.082731
28	1	0	3.207443	-0.883938	1.080195
29	6	0	3.626060	-3.192327	2.458655
30	1	0	1.710719	-3.517302	1.506959
31	1	0	3.045637	-3.330002	0.377422
32	1	0	3.846511	-4.254559	2.579456
33	1	0	4.570477	-2.672344	2.272816
34	1	0	3.225835	-2.825997	3.407752
35	6	0	1.981607	2.260911	0.633032
36	6	0	3.341873	3.973396	-0.201721
37	1	0	4.254175	3.400783	-0.029975
38	1	0	3.418624	4.544367	-1.123880
39	1	0	3.164366	4.639352	0.643429
40	8	0	2.620008	2.235273	1.653374
41	8	0	2.226890	3.093126	-0.384355
42	6	0	-1.643797	1.156282	1.103519
43	6	0	-1.949765	0.484219	2.295748
44	6	0	-2.660782	1.824495	0.403503
45	6	0	-3.249374	0.502957	2.786240
46	1	0	-1.166858	-0.051950	2.818523
47	6	0	-3.954885	1.834035	0.902438
48	1	0	-2.422649	2.330561	-0.524794
49	6	0	-4.252551	1.175366	2.093989
50	1	0	-3.479027	-0.016209	3.709460
51	1	0	-4.736246	2.350864	0.357145
52	1	0	-5.265654	1.183403	2.478904

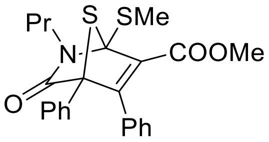

Electronic Energy (EE): -1966.92842371

Standard orientation:

Center Number	Atomic Number	Atomic Type	Coordinates (Angstroms) X	Y	Z
1	6	0	-0.104184	-1.720277	0.891145
2	6	0	-0.880453	-1.019390	-0.267628
3	6	0	1.432437	-0.983968	-0.697846
4	6	0	-0.364403	0.446303	-0.170685
5	6	0	0.941607	0.464888	-0.453623
6	16	0	0.052766	-1.656570	-1.735817
7	16	0	2.989550	-1.322775	-1.548110
8	6	0	4.241376	-0.365925	-0.644461
9	1	0	5.156873	-0.511531	-1.220725
10	1	0	4.021848	0.699466	-0.627775
11	1	0	4.404965	-0.740223	0.363793
12	7	0	1.219672	-1.727905	0.555960
13	8	0	-0.583905	-2.120613	1.924409
14	6	0	-2.362228	-1.249197	-0.272768
15	6	0	-3.108686	-0.932247	0.865920
16	6	0	-3.017872	-1.748229	-1.396402
17	6	0	-4.486056	-1.111268	0.872035
18	1	0	-2.611409	-0.542222	1.745112
19	6	0	-4.398016	-1.928069	-1.388687
20	1	0	-2.460546	-1.996242	-2.293916
21	6	0	-5.134933	-1.609227	-0.254485
22	1	0	-5.053243	-0.861218	1.761068
23	1	0	-4.891777	-2.320027	-2.270120
24	1	0	-6.209606	-1.749878	-0.246639
25	6	0	2.235569	-1.921638	1.579938
26	6	0	2.531192	-0.658632	2.388103
27	1	0	3.133564	-2.315688	1.098675
28	1	0	1.856297	-2.703519	2.241250
29	6	0	3.652020	-0.902271	3.392634
30	1	0	2.797565	0.166612	1.719927
31	1	0	1.617058	-0.356339	2.908518
32	1	0	3.853100	-0.008008	3.985315
33	1	0	3.390295	-1.710165	4.081213
34	1	0	4.581638	-1.184462	2.888643
35	6	0	1.777473	1.693330	-0.556824
36	6	0	2.961927	3.066832	-2.041603
37	1	0	3.882491	3.019278	-1.456848
38	1	0	3.187674	3.065981	-3.104942
39	1	0	2.406632	3.964834	-1.769205
40	8	0	2.071419	2.415997	0.360202
41	8	0	2.159292	1.901346	-1.817044
42	6	0	-1.223355	1.586628	0.204801
43	6	0	-0.900794	2.367099	1.318318
44	6	0	-2.369516	1.891060	-0.539097
45	6	0	-1.712183	3.436701	1.682253
46	1	0	-0.014200	2.130630	1.894081
47	6	0	-3.169898	2.966976	-0.178232
48	1	0	-2.628445	1.287349	-1.401912
49	6	0	-2.845515	3.738680	0.935440
50	1	0	-1.455987	4.032583	2.550594
51	1	0	-4.051211	3.201117	-0.764020
52	1	0	-3.475838	4.573755	1.219017

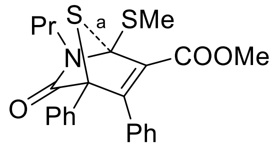

a: 2.777 Angstroms

Frequency: 254.39i

Electronic Energy (EE): -1966.88905631

Standard orientation:

Center Number	Atomic Number	Atomic Type	Coordinates (Angstroms) X	Y	Z
1	6	0	-0.705034	-1.629368	-0.338664
2	6	0	0.436898	-1.035993	0.483309
3	6	0	-1.838130	0.286160	0.419961
4	6	0	0.551789	0.442185	0.125874
5	6	0	-0.627766	1.083860	0.128472
6	16	0	-0.240436	-1.093986	2.223836
7	16	0	-3.163420	1.105439	1.264946
8	6	0	-4.387970	1.288938	-0.080295
9	1	0	-5.057523	2.078571	0.262370
10	1	0	-3.890025	1.619634	-0.989657
11	1	0	-4.959185	0.376205	-0.237263
12	7	0	-1.961277	-0.900023	-0.157417
13	8	0	-0.712152	-2.645188	-0.961644
14	6	0	1.704854	-1.833950	0.223010
15	6	0	2.217596	-1.866714	-1.077397
16	6	0	2.385817	-2.505115	1.232204
17	6	0	3.395436	-2.543391	-1.359411
18	1	0	1.695015	-1.349965	-1.876875
19	6	0	3.568214	-3.187806	0.950262
20	1	0	1.992125	-2.484417	2.240727
21	6	0	4.078689	-3.205085	-0.340788
22	1	0	3.780882	-2.554335	-2.372289
23	1	0	4.088607	-3.704783	1.748573
24	1	0	4.999853	-3.733902	-0.557407
25	6	0	-3.163549	-1.746757	-0.098247
26	6	0	-3.817497	-1.891945	-1.470127
27	1	0	-3.844640	-1.310813	0.632473
28	1	0	-2.849909	-2.721357	0.280639
29	6	0	-5.128036	-2.663107	-1.357916
30	1	0	-3.992643	-0.899849	-1.895926
31	1	0	-3.123825	-2.410094	-2.135632
32	1	0	-5.844596	-2.137755	-0.719585
33	1	0	-5.587528	-2.794296	-2.339095
34	1	0	-4.965626	-3.655544	-0.929686
35	6	0	-0.830861	2.527932	-0.184694
36	6	0	-0.039851	4.705119	0.123966
37	1	0	-1.022897	5.126018	0.336705
38	1	0	0.721009	5.184814	0.734222
39	1	0	0.189505	4.826353	-0.935780
40	8	0	-1.658845	2.918448	-0.972286
41	8	0	-0.002116	3.318913	0.481634
42	6	0	1.846122	1.087102	-0.163934
43	6	0	2.017572	1.812263	-1.345073
44	6	0	2.907248	0.969272	0.737809
45	6	0	3.239777	2.412636	-1.624615
46	1	0	1.198718	1.887786	-2.054075
47	6	0	4.119318	1.588334	0.463326
48	1	0	2.769677	0.410858	1.656698
49	6	0	4.290828	2.303318	-0.719618
50	1	0	3.371632	2.960334	-2.550509
51	1	0	4.934767	1.505273	1.172134
52	1	0	5.243373	2.773461	-0.935475

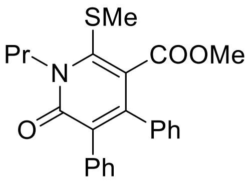

Electronic Energy (EE): -1568.78351729

Standard orientation:

Center Number	Atomic Number	Atomic Type	Coordinates (Angstroms) X	Y	Z
1	6	0	-0.454460	1.111710	0.135622
2	6	0	-0.502005	-0.254959	0.069902
3	6	0	0.718377	-1.007390	0.067157
4	6	0	1.922253	-0.370606	0.154107
5	6	0	-1.669998	1.966195	0.173788
6	6	0	-2.659730	1.760483	1.137755
7	6	0	-1.839870	2.987577	-0.764050
8	6	0	-3.802709	2.552279	1.156119
9	1	0	-2.533002	0.976731	1.876454
10	6	0	-2.987997	3.770433	-0.753102
11	1	0	-1.067297	3.166574	-1.502170
12	6	0	-3.972719	3.555648	0.207138
13	1	0	-4.560576	2.384976	1.912824
14	1	0	-3.110416	4.554002	-1.492034
15	1	0	-4.865057	4.171036	0.219096
16	16	0	3.441870	-1.305538	0.047214
17	6	0	3.657290	-1.706888	1.812777
18	1	0	2.824376	-2.320491	2.151153
19	1	0	4.586464	-2.273099	1.884602
20	1	0	3.742828	-0.798334	2.409092
21	8	0	0.950077	3.023351	0.197459
22	6	0	0.830040	1.807600	0.185309
23	6	0	3.252612	1.723627	0.375743
24	6	0	3.801144	2.145603	-0.982088
25	1	0	3.963869	1.088112	0.899224
26	1	0	3.047097	2.602112	0.985722
27	6	0	5.084253	2.952809	-0.819342
28	1	0	3.989986	1.251992	-1.584736
29	1	0	3.041221	2.739743	-1.495251
30	1	0	5.478791	3.262138	-1.788925
31	1	0	4.904818	3.855354	-0.229029
32	1	0	5.858181	2.367150	-0.315167
33	6	0	0.676315	-2.498407	-0.087145
34	8	0	0.265918	-2.841753	-1.305823
35	8	0	0.953217	-3.290147	0.776495
36	6	0	0.094001	-4.245698	-1.531686
37	1	0	-0.633112	-4.653663	-0.827982
38	1	0	-0.273149	-4.336282	-2.550978
39	1	0	1.046083	-4.765715	-1.420379
40	7	0	1.977988	0.994793	0.254578
41	6	0	-1.789205	-1.005325	0.001376
42	6	0	-2.078058	-1.967764	0.970624
43	6	0	-2.704971	-0.770452	-1.025828
44	6	0	-3.273683	-2.677786	0.920974
45	1	0	-1.372722	-2.150957	1.774258
46	6	0	-3.893570	-1.487572	-1.078775
47	1	0	-2.484235	-0.022615	-1.779409
48	6	0	-4.182303	-2.440502	-0.104546
49	1	0	-3.492183	-3.413979	1.685909
50	1	0	-4.598330	-1.300853	-1.880697
51	1	0	-5.113209	-2.994175	-0.145062

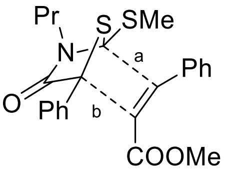

a: 2.315 Angstroms
b: 2.239 Angstroms

Frequency: 399.76i

Electronic Energy (EE): -1966.84745232

Standard orientation:

Center Number	Atomic Number	Atomic Type	Coordinates (Angstroms) X	Y	Z
1	6	0	0.867780	1.664165	0.116460
2	6	0	1.453112	0.588213	-0.683448
3	6	0	-0.905405	0.895979	-1.192115
4	6	0	0.350093	-0.913509	0.557316
5	6	0	-0.847203	-0.937039	0.221355
6	16	0	0.462040	0.285360	-2.076603
7	16	0	-2.361315	1.112025	-2.182115
8	6	0	-3.778550	1.112604	-1.038148
9	1	0	-4.634728	0.889832	-1.676764
10	1	0	-3.693793	0.330077	-0.290392
11	1	0	-3.923013	2.087074	-0.575925
12	7	0	-0.468721	1.814002	-0.274295
13	8	0	1.365661	2.319979	1.016366
14	6	0	2.884113	0.246711	-0.704620
15	6	0	3.834629	1.046562	-0.054923
16	6	0	3.308244	-0.922878	-1.350162
17	6	0	5.177876	0.690603	-0.081292
18	1	0	3.511321	1.935006	0.469539
19	6	0	4.652266	-1.270382	-1.373507
20	1	0	2.576821	-1.576301	-1.814802
21	6	0	5.592944	-0.462768	-0.740771
22	1	0	5.904615	1.320546	0.418986
23	1	0	4.961203	-2.179474	-1.875869
24	1	0	6.642133	-0.735142	-0.756040
25	6	0	-1.323824	2.724506	0.476821
26	6	0	-1.928532	2.075395	1.718038
27	1	0	-2.087540	3.109720	-0.200792
28	1	0	-0.682945	3.557376	0.770223
29	6	0	-2.787384	3.073642	2.486311
30	1	0	-2.526460	1.208690	1.424040
31	1	0	-1.112471	1.706472	2.346213
32	1	0	-3.209286	2.619972	3.385013
33	1	0	-2.198083	3.941072	2.795279
34	1	0	-3.618458	3.435446	1.873449
35	6	0	1.492278	-1.542511	1.210404
36	8	0	1.870532	-2.659095	0.961259
37	8	0	2.086505	-0.723176	2.076461
38	6	0	3.329300	-1.192494	2.609681
39	1	0	4.009472	-1.459905	1.798529
40	1	0	3.732723	-0.362827	3.185353
41	1	0	3.165402	-2.058991	3.252096
42	6	0	-2.162426	-1.527575	0.241486
43	6	0	-2.699060	-2.084643	-0.926612
44	6	0	-2.925300	-1.528628	1.417853
45	6	0	-3.984432	-2.610114	-0.920583
46	1	0	-2.105298	-2.091616	-1.833351
47	6	0	-4.212969	-2.050557	1.412256
48	1	0	-2.501428	-1.114296	2.325525
49	6	0	-4.750480	-2.583623	0.242995
50	1	0	-4.391707	-3.038217	-1.829385
51	1	0	-4.796832	-2.045294	2.325611
52	1	0	-5.756197	-2.987011	0.241548

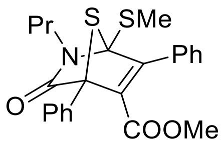

Electronic Energy (EE): -1966.92788845

Standard orientation:

Center Number	Atomic Number	Atomic Type	Coordinates (Angstroms) X	Y	Z
1	6	0	-0.947065	-1.590244	0.778951
2	6	0	-1.469181	-0.598706	-0.309463
3	6	0	0.742890	-1.241186	-0.788866
4	6	0	-0.529581	0.608452	-0.142876
5	6	0	0.728033	0.283956	-0.444449
6	16	0	-0.779709	-1.389288	-1.841607
7	16	0	2.114629	-1.973183	-1.709203
8	6	0	3.618259	-1.656114	-0.733796
9	1	0	4.434583	-1.857847	-1.429580
10	1	0	3.689576	-0.618874	-0.415194
11	1	0	3.707876	-2.331309	0.115032
12	7	0	0.314284	-1.965712	0.418636
13	8	0	-1.533252	-1.908374	1.785325
14	6	0	-2.945865	-0.342422	-0.278318
15	6	0	-3.514667	0.174350	0.888910
16	6	0	-3.761449	-0.597060	-1.376926
17	6	0	-4.877932	0.430777	0.949913
18	1	0	-2.891422	0.362755	1.756319
19	6	0	-5.128239	-0.343413	-1.313393
20	1	0	-3.339456	-0.989880	-2.296133
21	6	0	-5.689126	0.171502	-0.151418
22	1	0	-5.308013	0.829097	1.861523
23	1	0	-5.750624	-0.547973	-2.176662
24	1	0	-6.753318	0.370759	-0.102820
25	6	0	1.209450	-2.589542	1.379835
26	6	0	1.967921	-1.581552	2.240426
27	1	0	1.887645	-3.253291	0.838120
28	1	0	0.580917	-3.219323	2.013083
29	6	0	2.915329	-2.285338	3.205807
30	1	0	2.529468	-0.894172	1.600232
31	1	0	1.239498	-0.978533	2.792383
32	1	0	3.449781	-1.568077	3.831415
33	1	0	2.367140	-2.962891	3.865890
34	1	0	3.661110	-2.877388	2.666723
35	6	0	-1.036341	1.973827	0.179378
36	8	0	-1.738438	2.611245	-0.556823
37	8	0	-0.617856	2.400636	1.373176
38	6	0	-1.044434	3.721249	1.736528
39	1	0	-2.133552	3.773033	1.759045
40	1	0	-0.633537	3.900490	2.727068
41	1	0	-0.656913	4.450905	1.024855
42	6	0	1.902606	1.179323	-0.487019
43	6	0	2.532312	1.436147	-1.710099
44	6	0	2.398970	1.768343	0.678673
45	6	0	3.650071	2.259953	-1.759710
46	1	0	2.138172	0.992455	-2.617154
47	6	0	3.521657	2.588936	0.623975
48	1	0	1.902844	1.576811	1.622662
49	6	0	4.152189	2.830587	-0.592317
50	1	0	4.128987	2.458082	-2.711591
51	1	0	3.904531	3.036992	1.533696
52	1	0	5.027510	3.468691	-0.632309

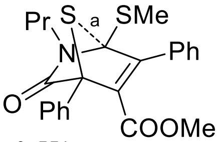

a: 2.771 Angstroms

Frequency: 211.87i

Electronic Energy (EE): -1966.90003499

Standard orientation:

Center Number	Atomic Number	Atomic Type	Coordinates (Angstroms) X	Y	Z
1	6	0	0.604173	-1.778925	-0.483754
2	6	0	1.245231	-0.753839	0.453003
3	6	0	-1.378023	-0.842411	0.391543
4	6	0	0.534477	0.564524	0.204819
5	6	0	-0.798509	0.530048	0.204455
6	16	0	0.719091	-1.360930	2.126375
7	16	0	-2.911183	-1.099648	1.138325
8	6	0	-2.970427	0.271910	2.324333
9	1	0	-3.610754	-0.086578	3.130416
10	1	0	-1.966119	0.446743	2.711928
11	1	0	-3.397898	1.166721	1.878445
12	7	0	-0.846020	-1.822447	-0.350917
13	8	0	1.152781	-2.574644	-1.183118
14	6	0	2.740140	-0.659408	0.189238
15	6	0	3.174226	-0.343749	-1.102383
16	6	0	3.683138	-0.818748	1.198358
17	6	0	4.526272	-0.184185	-1.376783
18	1	0	2.449692	-0.222110	-1.901602
19	6	0	5.039674	-0.662582	0.922737
20	1	0	3.347741	-1.057540	2.199478
21	6	0	5.465345	-0.343490	-0.360573
22	1	0	4.846335	0.058720	-2.383692
23	1	0	5.764225	-0.788629	1.719103
24	1	0	6.521708	-0.221246	-0.571229
25	6	0	-1.483347	-3.139543	-0.489131
26	6	0	-2.551240	-3.151660	-1.578209
27	1	0	-1.889641	-3.423616	0.488490
28	1	0	-0.690177	-3.842897	-0.736907
29	6	0	-3.227158	-4.516342	-1.653346
30	1	0	-3.299445	-2.376894	-1.385342
31	1	0	-2.072863	-2.908615	-2.530949
32	1	0	-3.972037	-4.538376	-2.450625
33	1	0	-2.497846	-5.305384	-1.854148
34	1	0	-3.733140	-4.756765	-0.714637
35	6	0	1.330218	1.821112	0.013704
36	8	0	1.952001	2.351713	0.892199
37	8	0	1.268040	2.256644	-1.242459
38	6	0	2.044384	3.430095	-1.522407
39	1	0	3.097896	3.238866	-1.314151
40	1	0	1.893024	3.636099	-2.578946
41	1	0	1.695092	4.267724	-0.917389
42	6	0	-1.685413	1.685054	-0.064477
43	6	0	-1.477434	2.902977	0.589546
44	6	0	-2.726658	1.573534	-0.989907
45	6	0	-2.293651	3.993873	0.311800
46	1	0	-0.692184	2.986281	1.333620
47	6	0	-3.542669	2.664834	-1.261714
48	1	0	-2.887718	0.634710	-1.509491
49	6	0	-3.328801	3.876962	-0.610979
50	1	0	-2.127755	4.932488	0.827651
51	1	0	-4.342081	2.569833	-1.987329
52	1	0	-3.966723	4.726878	-0.823685

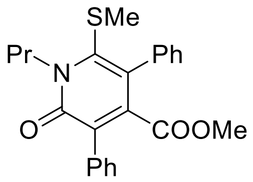

Electronic Energy (EE): -1568.77914969

Standard orientation:

Center Number	Atomic Number	Atomic Type	Coordinates (Angstroms) X	Y	Z
1	6	0	1.326487	0.557519	-0.199879
2	6	0	0.486107	-0.515005	-0.139833
3	6	0	-0.935993	-0.375827	-0.088346
4	6	0	-1.455104	0.889625	-0.143075
5	6	0	2.803125	0.413419	-0.229937
6	6	0	3.413206	-0.422884	-1.168271
7	6	0	3.601460	1.091621	0.696197
8	6	0	4.794133	-0.596982	-1.166537
9	1	0	2.807399	-0.927286	-1.912335
10	6	0	4.977973	0.910075	0.700673
11	1	0	3.138379	1.759415	1.411974
12	6	0	5.578711	0.064219	-0.228967
13	1	0	5.253425	-1.245024	-1.904146
14	1	0	5.585015	1.434777	1.429363
15	1	0	6.654166	-0.071189	-0.226121
16	16	0	-3.222146	1.140445	-0.047749
17	6	0	-3.619674	0.961155	-1.816657
18	1	0	-3.401519	-0.053185	-2.148900
19	1	0	-4.689061	1.151278	-1.913220
20	1	0	-3.069220	1.687141	-2.415255
21	8	0	1.445498	2.925448	-0.220038
22	6	0	1.044428	-1.913177	-0.085221
23	8	0	1.042073	-2.676940	-1.013184
24	8	0	1.518159	-2.194802	1.123560
25	6	0	2.090975	-3.500672	1.274479
26	1	0	2.447346	-3.547607	2.300284
27	1	0	1.334969	-4.266337	1.096010
28	1	0	2.919453	-3.629727	0.577011
29	6	0	0.769303	1.906885	-0.216522
30	6	0	-1.168641	3.359888	-0.336209
31	6	0	-1.328439	3.995100	1.040248
32	1	0	-2.123154	3.324741	-0.856613
33	1	0	-0.460871	3.932724	-0.933571
34	6	0	-1.890947	5.407163	0.922528
35	1	0	-1.995087	3.372882	1.645203
36	1	0	-0.352862	4.011881	1.532382
37	1	0	-1.999565	5.870299	1.905036
38	1	0	-1.232978	6.042674	0.323687
39	1	0	-2.875811	5.397906	0.446769
40	7	0	-0.632558	1.989531	-0.251172
41	6	0	-1.793573	-1.587312	0.075705
42	6	0	-2.140761	-2.367824	-1.027851
43	6	0	-2.222884	-1.973021	1.346696
44	6	0	-2.926238	-3.504337	-0.864812
45	1	0	-1.781850	-2.089255	-2.012625
46	6	0	-3.004717	-3.110703	1.509983
47	1	0	-1.946747	-1.371945	2.206649
48	6	0	-3.361393	-3.876111	0.403247
49	1	0	-3.191935	-4.102904	-1.728665
50	1	0	-3.336721	-3.398633	2.500769
51	1	0	-3.971630	-4.762987	0.529972

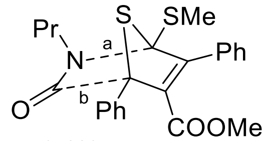

a: 2.330 Angstroms
b: 1.808 Angstroms

Frequency: 463.49i

Electronic Energy (EE): -1966.87420914

Standard orientation:

Center Number	Atomic Number	Atomic Type	Coordinates (Angstroms) X	Y	Z
1	6	0	0.744037	1.700053	0.530064
2	6	0	1.369866	0.375167	-0.529958
3	6	0	-0.850841	0.496629	-1.519681
4	6	0	0.510643	-0.748020	-0.172862
5	6	0	-0.744324	-0.679860	-0.692800
6	16	0	0.700315	1.016000	-2.093314
7	16	0	-2.258823	1.166843	-2.275168
8	6	0	-3.552742	0.907119	-1.021876
9	1	0	-4.273087	1.707949	-1.191276
10	1	0	-4.037831	-0.060459	-1.127685
11	1	0	-3.107800	1.017400	-0.034005
12	7	0	-0.531254	1.734517	0.427991
13	8	0	1.630793	2.325263	1.074297
14	6	0	2.859028	0.180063	-0.483347
15	6	0	3.541810	0.220213	0.736731
16	6	0	3.576474	-0.112312	-1.646836
17	6	0	4.908920	-0.028751	0.783442
18	1	0	3.005679	0.462060	1.642563
19	6	0	4.945395	-0.351193	-1.596442
20	1	0	3.065526	-0.171802	-2.601289
21	6	0	5.616329	-0.312233	-0.380107
22	1	0	5.424198	0.011415	1.736330
23	1	0	5.482876	-0.575379	-2.510442
24	1	0	6.682861	-0.500718	-0.339303
25	6	0	-1.213781	2.982756	0.717226
26	6	0	-2.007296	2.901628	2.020186
27	1	0	-1.895967	3.208676	-0.115268
28	1	0	-0.491181	3.807308	0.778753
29	6	0	-2.797985	4.178966	2.283254
30	1	0	-2.682696	2.040541	1.977294
31	1	0	-1.307313	2.711652	2.839268
32	1	0	-3.350504	4.117205	3.223315
33	1	0	-2.134654	5.046050	2.343848
34	1	0	-3.519964	4.367619	1.483153
35	6	0	0.947045	-1.796157	0.797117
36	8	0	1.074374	-2.955722	0.516077
37	8	0	1.186597	-1.271963	1.997319
38	6	0	1.735742	-2.171742	2.970557
39	1	0	2.674257	-2.590371	2.604729
40	1	0	1.906026	-1.571716	3.861070
41	1	0	1.030823	-2.976703	3.180779
42	6	0	-1.873837	-1.550288	-0.303407
43	6	0	-2.553565	-2.301283	-1.263839
44	6	0	-2.262725	-1.619431	1.035503
45	6	0	-3.617086	-3.112451	-0.885617
46	1	0	-2.242159	-2.252275	-2.302029
47	6	0	-3.330986	-2.427747	1.409560
48	1	0	-1.739898	-1.015814	1.771271
49	6	0	-4.009133	-3.173283	0.449654
50	1	0	-4.137964	-3.700526	-1.632198
51	1	0	-3.635149	-2.473251	2.448908
52	1	0	-4.841202	-3.803646	0.741298

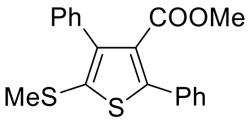

Electronic Energy (EE): -1680.36970739

Standard orientation:

Center Number	Atomic Number	Atomic Type	Coordinates (Angstroms) X	Y	Z
1	6	0	-1.011115	-1.659292	-0.168678
2	6	0	-1.028183	-0.289106	-0.063997
3	6	0	0.300850	0.244672	0.028190
4	6	0	1.288758	-0.707738	-0.007849
5	16	0	0.600887	-2.286056	-0.114379
6	16	0	-2.362253	-2.783106	-0.307534
7	6	0	-3.192165	-2.477662	1.283729
8	1	0	-4.008553	-3.197697	1.349652
9	1	0	-2.496003	-2.640645	2.105561
10	1	0	-3.597246	-1.466475	1.315876
11	6	0	0.600119	1.705872	0.082333
12	8	0	0.195328	2.460654	0.928839
13	8	0	1.383459	2.081228	-0.929920
14	6	0	1.802481	3.449372	-0.914044
15	1	0	0.938845	4.113632	-0.959192
16	1	0	2.376591	3.658551	-0.009858
17	1	0	2.426284	3.575482	-1.795488
18	6	0	2.746712	-0.518909	0.080806
19	6	0	3.617870	-1.256579	-0.725665
20	6	0	3.276895	0.408890	0.984875
21	6	0	4.991316	-1.062946	-0.636046
22	1	0	3.217061	-1.967809	-1.439845
23	6	0	4.649241	0.605188	1.065568
24	1	0	2.612583	0.961474	1.641398
25	6	0	5.510750	-0.129436	0.255463
26	1	0	5.655689	-1.637960	-1.270489
27	1	0	5.047253	1.324700	1.771860
28	1	0	6.581692	0.022220	0.321922
29	6	0	-2.273869	0.518108	-0.086875
30	6	0	-2.618616	1.325403	1.000526
31	6	0	-3.138360	0.442589	-1.182166
32	6	0	-3.811069	2.040482	0.992961
33	1	0	-1.948029	1.390796	1.848239
34	6	0	-4.329276	1.160039	-1.187960
35	1	0	-2.872932	-0.181912	-2.027435
36	6	0	-4.669466	1.958075	-0.099201
37	1	0	-4.069253	2.661646	1.842875
38	1	0	-4.991144	1.097063	-2.043909
39	1	0	-5.598518	2.516339	-0.103731

Routes for the Dipolar Cycloaddition of Mesoionic **6** and Methyl Propiolate **7b**

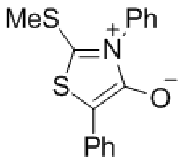

2-(Methylthio)-3,5-diphenylthiazol-3-ium-4-olate (**6**)

Electronic Energy (EE): -1543.76350736

Standard orientation:

Center Number	Atomic Number	Atomic Type	Coordinates (Angstroms) X	Y	Z
1	6	0	-1.382325	0.038349	-0.052481
2	6	0	-0.311686	-0.868984	-0.069079
3	6	0	0.801343	1.225849	-0.004642
4	7	0	0.936619	-0.092057	-0.041670
5	6	0	2.204983	-0.761310	-0.024659
6	6	0	2.543777	-1.515586	1.091836
7	6	0	3.049312	-0.665497	-1.123376
8	6	0	3.766155	-2.174089	1.111664
9	1	0	1.849855	-1.586900	1.920391
10	6	0	4.273888	-1.323930	-1.089942
11	1	0	2.744388	-0.091262	-1.991158
12	6	0	4.631059	-2.075698	0.024951
13	1	0	4.040671	-2.768349	1.974949
14	1	0	4.942375	-1.257816	-1.939773
15	1	0	5.582797	-2.593409	0.044484
16	6	0	-2.801046	-0.283586	-0.045034
17	6	0	-3.784996	0.715013	0.060032
18	6	0	-3.228847	-1.620896	-0.135896
19	6	0	-5.135358	0.394022	0.073328
20	1	0	-3.499447	1.759884	0.136019
21	6	0	-4.582685	-1.930941	-0.121052
22	1	0	-2.489296	-2.405893	-0.215376
23	6	0	-5.546717	-0.932563	-0.016085
24	1	0	-5.870017	1.187191	0.157127
25	1	0	-4.885256	-2.969983	-0.192102
26	1	0	-6.601014	-1.182568	-0.004277
27	8	0	-0.254480	-2.092459	-0.097333
28	16	0	2.164051	2.304660	0.075362
29	6	0	1.307268	3.893735	0.220959
30	1	0	0.699132	4.093675	-0.661136
31	1	0	0.710261	3.934484	1.131747
32	1	0	2.099994	4.639455	0.279488
33	16	0	-0.830907	1.691730	-0.011185

Route a

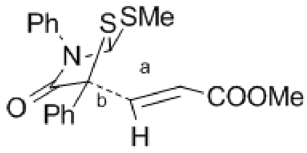

a: 3.143 Angstroms
b: 1.919 Angstroms

Frequency: 397.82i

Electronic Energy (EE): -1848.92225523

Standard orientation:

Center Number	Atomic Number	Atomic Type	Coordinates (Angstroms) X	Y	Z
1	6	0	0.652292	-1.199170	-0.276685
2	6	0	1.632251	-0.166418	0.013584
3	6	0	-0.584383	0.287937	1.061060
4	6	0	1.246716	0.828912	-1.580912
5	6	0	0.293476	1.643598	-1.635671
6	16	0	1.009867	0.853241	1.326712
7	7	0	-0.628362	-0.770383	0.255549
8	1	0	2.015538	0.416811	-2.221189
9	16	0	-1.850425	1.024626	1.987820
10	6	0	-3.427281	0.728838	1.120608
11	1	0	-4.098120	1.446694	1.596208
12	1	0	-3.318098	0.975581	0.067767
13	1	0	-3.793464	-0.281408	1.285111
14	6	0	-1.807540	-1.529622	-0.065104
15	6	0	-2.451457	-1.284254	-1.269023
16	6	0	-2.248157	-2.495108	0.828896
17	6	0	-3.597175	-2.012665	-1.567126
18	1	0	-2.075954	-0.510028	-1.929228
19	6	0	-3.395901	-3.217129	0.518601
20	1	0	-1.704117	-2.668824	1.750552
21	6	0	-4.070156	-2.971472	-0.674399
22	1	0	-4.121512	-1.827881	-2.496887
23	1	0	-3.759518	-3.971415	1.206035
24	1	0	-4.964510	-3.535245	-0.913027
25	6	0	3.093413	-0.435174	0.031193
26	6	0	3.971825	0.654238	0.015145
27	6	0	3.612578	-1.731478	0.038695
28	6	0	5.345562	0.452005	0.037907
29	1	0	3.571321	1.662955	-0.028283
30	6	0	4.989885	-1.928395	0.048678
31	1	0	2.941764	-2.580939	0.028787
32	6	0	5.858526	-0.842023	0.053888
33	1	0	6.014761	1.304348	0.030534
34	1	0	5.383808	-2.938015	0.054128
35	1	0	6.930191	-1.002567	0.062720
36	6	0	-0.855359	2.340762	-1.218080
37	8	0	-2.013951	1.973366	-1.386125
38	8	0	-0.566640	3.534721	-0.644445
39	6	0	-1.694034	4.338287	-0.306173
40	1	0	-2.347617	3.821082	0.398347
41	1	0	-1.287485	5.236618	0.154657
42	1	0	-2.264470	4.602199	-1.198564
43	8	0	0.747397	-2.204732	-0.934369

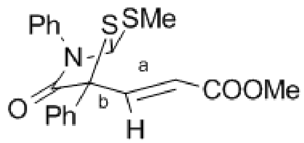

a: 3.367 Angstroms
b: 1.590 Angstroms

Electronic Energy (EE): -1848.92842850

Standard orientation:

Center Number	Atomic Number	Atomic Type	Coordinates (Angstroms) X	Y	Z
1	6	0	0.569930	-1.068543	-0.490383
2	6	0	1.596551	0.008901	-0.276160
3	6	0	-0.571065	0.292948	1.033116
4	6	0	1.378455	0.888080	-1.582663
5	6	0	0.446526	1.779827	-1.811313
6	16	0	1.021347	0.913676	1.208886
7	7	0	-0.679257	-0.683065	0.140544
8	1	0	2.092184	0.541305	-2.331430
9	16	0	-1.773098	0.941363	2.081513
10	6	0	-3.382570	0.751754	1.249990
11	1	0	-4.018606	1.442684	1.806380
12	1	0	-3.279127	1.090365	0.220874
13	1	0	-3.772299	-0.259008	1.336500
14	6	0	-1.868219	-1.445298	-0.142578
15	6	0	-2.556898	-1.173940	-1.315384
16	6	0	-2.265550	-2.434728	0.745973
17	6	0	-3.707420	-1.904632	-1.587159
18	1	0	-2.208192	-0.376803	-1.962484
19	6	0	-3.420908	-3.155678	0.461776
20	1	0	-1.686643	-2.628821	1.642361
21	6	0	-4.140332	-2.886717	-0.699686
22	1	0	-4.266884	-1.702191	-2.492419
23	1	0	-3.754309	-3.928887	1.143346
24	1	0	-5.039811	-3.450588	-0.917914
25	6	0	3.024246	-0.447486	-0.097241
26	6	0	4.044883	0.474811	-0.334385
27	6	0	3.345472	-1.730144	0.346079
28	6	0	5.372389	0.119051	-0.126806
29	1	0	3.794993	1.471444	-0.683610
30	6	0	4.675228	-2.085837	0.543105
31	1	0	2.564813	-2.459675	0.526265
32	6	0	5.689873	-1.162862	0.309981
33	1	0	6.157325	0.843323	-0.310211
34	1	0	4.917987	-3.087391	0.878235
35	1	0	6.725185	-1.443255	0.465395
36	6	0	-0.657068	2.339317	-1.147799
37	8	0	-1.818197	1.906031	-1.169587
38	8	0	-0.386482	3.537082	-0.546608
39	6	0	-1.519781	4.273885	-0.107552
40	1	0	-2.088677	3.724554	0.646413
41	1	0	-1.126607	5.191931	0.327354
42	1	0	-2.179605	4.514610	-0.943936
43	8	0	0.658259	-2.065489	-1.134651

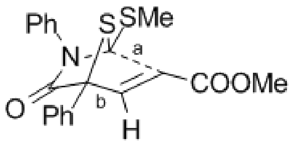

a: 2.792 Angstroms
b: 1.608 Angstroms

Frequency: 175.95i

Electronic Energy (EE): -1848.92588408

Standard orientation:

Center Number	Atomic Number	Atomic Type	Coordinates (Angstroms) X	Y	Z
1	6	0	0.708319	-1.098291	-0.392730
2	6	0	1.662257	0.042120	-0.151786
3	6	0	-0.534347	0.332074	0.969698
4	6	0	1.249314	1.022365	-1.357403
5	6	0	0.163132	1.716410	-1.352858
6	16	0	1.080654	0.807448	1.395911
7	7	0	-0.557510	-0.756659	0.177310
8	1	0	1.969112	0.921446	-2.169673
9	16	0	-1.805047	1.061258	1.869084
10	6	0	-3.409626	0.546538	1.174693
11	1	0	-4.106225	1.221406	1.675547
12	1	0	-3.417464	0.740508	0.105761
13	1	0	-3.650851	-0.482638	1.428001
14	6	0	-1.704138	-1.581251	-0.101152
15	6	0	-2.409437	-1.389919	-1.280019
16	6	0	-2.047690	-2.564173	0.817347
17	6	0	-3.516769	-2.193869	-1.525964
18	1	0	-2.115203	-0.593728	-1.954462
19	6	0	-3.157877	-3.361430	0.560009
20	1	0	-1.457585	-2.690871	1.718416
21	6	0	-3.892133	-3.171641	-0.607765
22	1	0	-4.088591	-2.051702	-2.434968
23	1	0	-3.445882	-4.129749	1.267303
24	1	0	-4.757979	-3.792402	-0.806483
25	6	0	3.131763	-0.276336	-0.100086
26	6	0	4.043068	0.761193	-0.308662
27	6	0	3.603006	-1.554687	0.199749
28	6	0	5.409698	0.523091	-0.218837
29	1	0	3.678811	1.756990	-0.539850
30	6	0	4.970877	-1.790592	0.281504
31	1	0	2.907580	-2.368627	0.360919
32	6	0	5.875936	-0.754834	0.072673
33	1	0	6.108583	1.335182	-0.380337
34	1	0	5.328512	-2.787891	0.509052
35	1	0	6.941243	-0.943444	0.136628
36	6	0	-1.066427	2.291916	-1.095541
37	8	0	-2.171216	1.815355	-1.388346
38	8	0	-0.970451	3.508907	-0.477473
39	6	0	-2.202133	4.197288	-0.301747
40	1	0	-2.907323	3.611311	0.290562
41	1	0	-1.952946	5.116841	0.225605
42	1	0	-2.659855	4.434975	-1.264239
43	8	0	0.873934	-2.083776	-1.051133

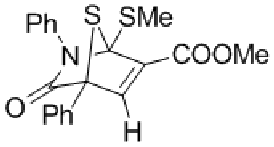

Electronic Energy (EE): -1849.00874607

Standard orientation:

Center Number	Atomic Number	Atomic Type	Coordinates (Angstroms) X	Y	Z
1	6	0	0.542943	-1.066807	-0.611635
2	6	0	1.590602	-0.095584	0.035011
3	6	0	-0.580490	0.383173	0.858046
4	6	0	1.153938	1.277449	-0.445526
5	6	0	-0.056522	1.579191	0.023159
6	16	0	0.964700	-0.077235	1.792371
7	7	0	-0.682134	-0.751492	-0.086663
8	1	0	1.729625	1.871553	-1.142934
9	16	0	-1.896224	0.591488	2.074734
10	6	0	-3.465515	0.678475	1.149962
11	1	0	-4.116754	1.299630	1.766900
12	1	0	-3.326570	1.173057	0.193997
13	1	0	-3.900855	-0.312122	1.032774
14	6	0	-1.874976	-1.460460	-0.414000
15	6	0	-2.600582	-1.114593	-1.549068
16	6	0	-2.302898	-2.488901	0.419718
17	6	0	-3.779950	-1.791528	-1.839700
18	1	0	-2.241234	-0.316013	-2.188862
19	6	0	-3.485548	-3.159018	0.127593
20	1	0	-1.711701	-2.744286	1.291792
21	6	0	-4.225529	-2.807615	-0.998745
22	1	0	-4.350819	-1.525998	-2.721767
23	1	0	-3.826909	-3.956929	0.776432
24	1	0	-5.146205	-3.332665	-1.225497
25	6	0	3.033142	-0.481943	-0.090888
26	6	0	4.019381	0.497562	-0.215472
27	6	0	3.412975	-1.823691	-0.006611
28	6	0	5.362369	0.141388	-0.281286
29	1	0	3.747973	1.546926	-0.245234
30	6	0	4.754918	-2.176780	-0.070561
31	1	0	2.658963	-2.593177	0.103807
32	6	0	5.732860	-1.196631	-0.213800
33	1	0	6.117808	0.912230	-0.380029
34	1	0	5.036684	-3.221525	-0.010092
35	1	0	6.779113	-1.475046	-0.265852
36	6	0	-0.816826	2.790802	-0.350246
37	8	0	-2.015884	2.900698	-0.303362
38	8	0	-0.004762	3.761971	-0.776649
39	6	0	-0.653311	4.962431	-1.212727
40	1	0	-1.239176	5.393482	-0.400518
41	1	0	0.146859	5.638748	-1.502743
42	1	0	-1.303874	4.756013	-2.063413
43	8	0	0.778211	-1.908043	-1.440630

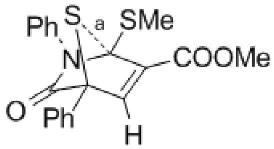

a: 2.741 Angstroms

Frequency: 264.35i

Electronic Energy (EE): -1848.98176144

Standard orientation:

Center Number	Atomic Number	Atomic Type	Coordinates (Angstroms) X	Y	Z
1	6	0	0.397092	-1.026149	-0.412288
2	6	0	1.450257	-0.222392	0.347099
3	6	0	-0.980738	0.761279	0.338421
4	6	0	1.318288	1.218280	-0.052269
5	6	0	0.082193	1.725925	-0.059307
6	16	0	0.834872	-0.286241	2.104853
7	7	0	-0.921828	-0.457954	-0.266697
8	1	0	2.197663	1.814056	-0.269805
9	16	0	-2.351590	1.371459	1.170837
10	6	0	-2.973142	-0.094410	2.036247
11	1	0	-3.485666	0.305591	2.911955
12	1	0	-3.670698	-0.669666	1.430492
13	1	0	-2.120124	-0.698224	2.351483
14	6	0	-2.064118	-1.280682	-0.536239
15	6	0	-3.075425	-0.768606	-1.341614
16	6	0	-2.148571	-2.560250	0.003520
17	6	0	-4.207107	-1.538666	-1.581429
18	1	0	-2.974692	0.223138	-1.768080
19	6	0	-3.277823	-3.327140	-0.255463
20	1	0	-1.347234	-2.939539	0.626944
21	6	0	-4.309395	-2.816826	-1.039259
22	1	0	-5.001114	-1.143953	-2.203851
23	1	0	-3.353289	-4.324191	0.161689
24	1	0	-5.189640	-3.417678	-1.235482
25	6	0	2.834252	-0.782875	0.077657
26	6	0	3.367839	-0.646480	-1.207519
27	6	0	3.581192	-1.432097	1.054369
28	6	0	4.629102	-1.143330	-1.507653
29	1	0	2.788180	-0.156016	-1.984536
30	6	0	4.846599	-1.931381	0.753974
31	1	0	3.169825	-1.539462	2.050396
32	6	0	5.374008	-1.788369	-0.523437
33	1	0	5.029099	-1.031398	-2.508853
34	1	0	5.419137	-2.433448	1.525421
35	1	0	6.359248	-2.176709	-0.754551
36	6	0	-0.254468	3.128045	-0.390190
37	8	0	-1.380677	3.545367	-0.503422
38	8	0	0.835051	3.872918	-0.568915
39	6	0	0.599134	5.244504	-0.911180
40	1	0	0.055127	5.744733	-0.109447
41	1	0	1.583992	5.687959	-1.034549
42	1	0	0.030537	5.312814	-1.839017
43	8	0	0.557489	-2.046546	-1.009540

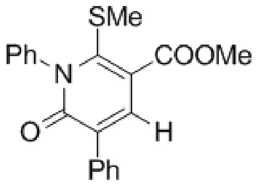

Electronic Energy (EE): -1450.86125235

Standard orientation:

Center Number	Atomic Number	Atomic Type	Coordinates (Angstroms) X	Y	Z
1	6	0	1.550673	-0.317274	-0.008937
2	6	0	1.366104	1.028780	-0.047970
3	6	0	0.076309	1.633910	-0.076630
4	6	0	-1.033668	0.825660	-0.106501
5	6	0	2.907106	-0.907687	0.071211
6	6	0	3.880388	-0.299208	0.871253
7	6	0	3.253769	-2.050697	-0.657955
8	6	0	5.172636	-0.807350	0.929748
9	1	0	3.616235	0.566934	1.468582
10	6	0	4.548018	-2.553413	-0.602131
11	1	0	2.507635	-2.543298	-1.267102
12	6	0	5.511215	-1.935977	0.190336
13	1	0	5.911280	-0.328130	1.561878
14	1	0	4.803615	-3.435238	-1.178419
15	1	0	6.517214	-2.336518	0.237489
16	16	0	-2.692728	1.451816	-0.304317
17	6	0	-3.103805	1.774047	1.437518
18	1	0	-2.399680	2.489771	1.855870
19	1	0	-4.104548	2.207437	1.429948
20	1	0	-3.122103	0.844131	2.004918
21	8	0	0.424988	-2.414365	-0.000907
22	6	0	0.388867	-1.199873	-0.031066
23	1	0	2.231032	1.681703	-0.065264
24	6	0	-0.015896	3.119286	-0.068174
25	8	0	1.028184	3.666542	-0.705466
26	8	0	-0.875847	3.774353	0.461445
27	6	0	1.074401	5.096788	-0.705736
28	1	0	1.134793	5.476407	0.314982
29	1	0	1.969632	5.362445	-1.262755
30	1	0	0.188882	5.505051	-1.193299
31	7	0	-0.868371	-0.538493	-0.084251
32	6	0	-2.013148	-1.425696	-0.090364
33	6	0	-2.541107	-1.849797	1.119487
34	6	0	-2.525932	-1.872039	-1.299365
35	6	0	-3.628651	-2.717243	1.117135
36	1	0	-2.095154	-1.510970	2.047975
37	6	0	-3.611221	-2.739149	-1.294568
38	1	0	-2.080661	-1.533879	-2.228216
39	6	0	-4.164720	-3.159163	-0.087964
40	1	0	-4.049180	-3.054762	2.057083
41	1	0	-4.022905	-3.089647	-2.233624
42	1	0	-5.010455	-3.836707	-0.087748

Route c

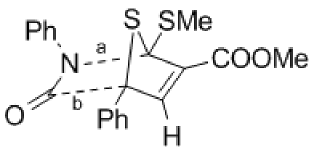

a: 2.335 Angstroms
b: 1.757 Angstroms

Frequency: 382.29i

Electronic Energy (EE): -1848.96700198

Standard orientation:

Center Number	Atomic Number	Atomic Type	Coordinates (Angstroms) X	Y	Z
1	6	0	0.676917	-1.281079	-0.567101
2	6	0	1.645931	-0.006972	0.156537
3	6	0	-0.393780	0.542044	1.393095
4	6	0	0.905088	1.095778	-0.442472
5	6	0	-0.255195	1.372691	0.207217
6	16	0	1.164171	-0.026577	1.910914
7	7	0	-0.408671	-1.415303	0.119383
8	1	0	1.192878	1.534335	-1.390716
9	16	0	-1.718299	0.347674	2.466491
10	6	0	-3.206879	0.709693	1.483871
11	1	0	-3.963628	0.025881	1.867863
12	1	0	-3.511804	1.742643	1.618240
13	1	0	-3.030903	0.480990	0.433428
14	6	0	-1.643261	-1.751782	-0.415260
15	6	0	-2.053021	-1.318600	-1.686930
16	6	0	-2.555030	-2.442994	0.396012
17	6	0	-3.350777	-1.560218	-2.120022
18	1	0	-1.348596	-0.792772	-2.322651
19	6	0	-3.848657	-2.681676	-0.048553
20	1	0	-2.222659	-2.781036	1.370687
21	6	0	-4.257073	-2.234979	-1.303916
22	1	0	-3.657376	-1.221351	-3.103484
23	1	0	-4.543258	-3.219001	0.587791
24	1	0	-5.267639	-2.420914	-1.648046
25	6	0	3.112533	-0.166063	-0.079575
26	6	0	3.901434	0.922540	-0.451776
27	6	0	3.713643	-1.410178	0.122361
28	6	0	5.271865	0.768135	-0.631047
29	1	0	3.448541	1.898745	-0.588016
30	6	0	5.083385	-1.561234	-0.048795
31	1	0	3.103472	-2.262663	0.400803
32	6	0	5.864913	-0.473312	-0.428876
33	1	0	5.875888	1.620207	-0.920339
34	1	0	5.540851	-2.531439	0.105778
35	1	0	6.933145	-0.594374	-0.566908
36	6	0	-1.184160	2.450774	-0.214167
37	8	0	-1.904297	3.061241	0.532715
38	8	0	-1.066573	2.716465	-1.515990
39	6	0	-1.837155	3.828549	-1.990374
40	1	0	-1.544830	4.740721	-1.469163
41	1	0	-1.615141	3.907554	-3.051443
42	1	0	-2.900410	3.642949	-1.836489
43	8	0	1.182608	-1.753656	-1.559584

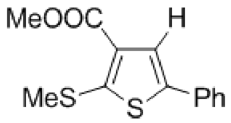

Electronic Energy (EE): -1449.35350206

Standard orientation:

Center Number	Atomic Number	Atomic Type	Coordinates (Angstroms) X	Y	Z
1	6	0	-1.094283	-0.115070	0.012303
2	6	0	-0.180012	-1.119798	0.073693
3	6	0	1.174566	-0.669929	0.040580
4	6	0	1.282839	0.704119	-0.043904
5	16	0	-0.289082	1.429429	-0.093645
6	1	0	-0.436353	-2.167231	0.164035
7	6	0	2.279788	-1.641716	0.107985
8	8	0	2.127562	-2.836375	0.184148
9	8	0	3.481575	-1.054113	0.073921
10	6	0	4.609833	-1.930433	0.132104
11	1	0	4.602505	-2.498281	1.063075
12	1	0	4.601788	-2.618490	-0.713701
13	1	0	5.485089	-1.286650	0.089026
14	6	0	-2.561490	-0.198900	0.013312
15	6	0	-3.348790	0.821876	0.556483
16	6	0	-3.194547	-1.321096	-0.533491
17	6	0	-4.734828	0.722103	0.554021
18	1	0	-2.875461	1.690712	1.001531
19	6	0	-4.579466	-1.422670	-0.523766
20	1	0	-2.597152	-2.105282	-0.984426
21	6	0	-5.355419	-0.401237	0.017120
22	1	0	-5.330241	1.521078	0.980491
23	1	0	-5.054443	-2.297352	-0.952781
24	1	0	-6.436234	-0.479227	0.017111
25	16	0	2.742323	1.673974	-0.108819
26	6	0	2.048873	3.344296	-0.207334
27	1	0	1.459357	3.588195	0.676968
28	1	0	2.911594	4.010173	-0.245173
29	1	0	1.462218	3.482540	-1.115920

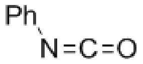

Electronic Energy (EE): -399.684337260

Standard orientation:

Center Number	Atomic Number	Atomic Type	Coordinates (Angstroms) X	Y	Z
1	7	0	1.446721	-0.613707	-0.000039
2	6	0	2.522787	-0.086062	0.000012
3	8	0	3.615971	0.316662	0.000040
4	6	0	0.088244	-0.267649	-0.000023
5	6	0	-0.854225	-1.292429	-0.000012
6	6	0	-0.316660	1.067688	-0.000034
7	6	0	-2.207744	-0.976664	0.000020
8	1	0	-0.514802	-2.320965	-0.000017
9	6	0	-1.672065	1.370304	-0.000002
10	1	0	0.428535	1.855185	-0.000068
11	6	0	-2.621056	0.351818	0.000031
12	1	0	-2.941151	-1.774468	0.000031
13	1	0	-1.985826	2.407719	0.000000
14	1	0	-3.677252	0.593134	0.000057

Route b

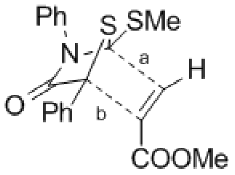

a: 2.126 Angstroms
b: 2.493 Angstroms

Frequency: 466.36i

Electronic Energy (EE): -1848.91938460

Standard orientation:

Center Number	Atomic Number	Atomic Type	Coordinates (Angstroms) X	Y	Z
1	6	0	-0.118938	-0.604228	0.888772
2	6	0	0.957071	0.371247	0.707019
3	6	0	-1.210318	1.321813	0.113951
4	6	0	0.439953	0.103613	-1.717507
5	6	0	-0.598513	0.758775	-1.842709
6	16	0	0.318853	1.967292	0.612258
7	7	0	-1.341905	0.047646	0.644787
8	16	0	-2.553753	2.492009	0.126545
9	6	0	-3.597668	1.940591	-1.261001
10	1	0	-4.588145	2.345974	-1.052097
11	1	0	-3.243252	2.345440	-2.207478
12	1	0	-3.666937	0.854212	-1.296062
13	6	0	-2.548434	-0.713974	0.507368
14	6	0	-2.621863	-1.705489	-0.465984
15	6	0	-3.628819	-0.433730	1.335619
16	6	0	-3.812266	-2.405131	-0.628324
17	1	0	-1.753355	-1.926402	-1.075307
18	6	0	-4.817126	-1.134778	1.160499
19	1	0	-3.531554	0.325907	2.101273
20	6	0	-4.911497	-2.113609	0.175576
21	1	0	-3.879547	-3.181503	-1.381266
22	1	0	-5.665452	-0.920159	1.799563
23	1	0	-5.838388	-2.659487	0.043369
24	6	0	2.373997	0.118829	0.964268
25	6	0	3.319205	1.149464	0.816648
26	6	0	2.827736	-1.162773	1.314711
27	6	0	4.665302	0.916669	1.044843
28	1	0	3.001035	2.139275	0.506578
29	6	0	4.182682	-1.387036	1.540108
30	1	0	2.115582	-1.969308	1.410630
31	6	0	5.104584	-0.355178	1.412478
32	1	0	5.376361	1.726584	0.930208
33	1	0	4.514110	-2.381150	1.816866
34	1	0	6.157902	-0.537003	1.592235
35	8	0	-0.045504	-1.785634	1.169989
36	1	0	-1.356651	1.183754	-2.469542
37	6	0	1.649328	-0.666898	-1.901659
38	8	0	1.719896	-1.868171	-1.844443
39	8	0	2.690399	0.144490	-2.120638
40	6	0	3.967538	-0.501183	-2.181082
41	1	0	4.694075	0.298448	-2.305502
42	1	0	4.158149	-1.050093	-1.257932
43	1	0	4.007755	-1.184287	-3.030708

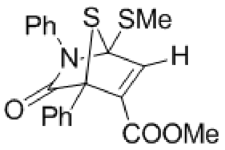

Electronic Energy (EE): -1849.01017128

Standard orientation:

Center Number	Atomic Number	Atomic Type	Coordinates (Angstroms) X	Y	Z
1	6	0	-0.034201	-1.024962	-0.692483
2	6	0	1.111230	-0.494451	0.218499
3	6	0	-0.971385	-0.145516	1.279071
4	6	0	0.789917	1.010743	0.275835
5	6	0	-0.373566	1.211839	0.894818
6	16	0	0.508246	-1.048342	1.889119
7	7	0	-1.226078	-0.865694	-0.004399
8	16	0	-2.306039	-0.191053	2.485372
9	6	0	-3.337747	1.208328	1.955191
10	1	0	-4.252467	1.131976	2.544213
11	1	0	-2.859012	2.160624	2.179092
12	1	0	-3.597693	1.137753	0.899162
13	6	0	-2.453991	-0.677772	-0.710596
14	6	0	-2.554108	0.302616	-1.696356
15	6	0	-3.557627	-1.454866	-0.371115
16	6	0	-3.773016	0.518279	-2.329600
17	1	0	-1.677917	0.881381	-1.969569
18	6	0	-4.776840	-1.226371	-1.000322
19	1	0	-3.447632	-2.223852	0.383148
20	6	0	-4.887030	-0.238540	-1.974929
21	1	0	-3.852933	1.275678	-3.100601
22	1	0	-5.638945	-1.827088	-0.734728
23	1	0	-5.837085	-0.067417	-2.467682
24	6	0	2.530093	-0.909576	-0.041355
25	6	0	3.546036	-0.223202	0.630030
26	6	0	2.859341	-1.987051	-0.861179
27	6	0	4.872980	-0.595421	0.471189
28	1	0	3.293234	0.604487	1.286989
29	6	0	4.192321	-2.357809	-1.018344
30	1	0	2.083367	-2.525662	-1.387340
31	6	0	5.199890	-1.665228	-0.358409
32	1	0	5.651968	-0.053415	0.994660
33	1	0	4.440038	-3.193464	-1.662522
34	1	0	6.235782	-1.957214	-0.486235
35	8	0	0.077469	-1.420639	-1.822687
36	1	0	-0.889582	2.155844	1.008760
37	6	0	1.548761	2.032453	-0.483795
38	8	0	2.211614	1.796367	-1.458259
39	8	0	1.377928	3.254883	0.030903
40	6	0	2.033922	4.315219	-0.675123
41	1	0	1.792662	5.223964	-0.129314
42	1	0	3.112146	4.153022	-0.685397
43	1	0	1.665338	4.376410	-1.699528

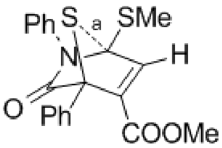

a: 2.768 Angstroms

Frequency: 261.39i

Electronic Energy (EE): -1848.97511944

Standard orientation:

Center Number	Atomic Number	Atomic Type	Coordinates (Angstroms) X	Y	Z
1	6	0	-0.228163	-0.821098	-0.441067
2	6	0	0.953678	-0.273900	0.359833
3	6	0	-1.143999	1.292612	0.113959
4	6	0	1.221185	1.140559	-0.118173
5	6	0	0.148029	1.923067	-0.223594
6	16	0	0.210956	-0.128486	2.064475
7	7	0	-1.377807	0.071168	-0.407602
8	16	0	-2.320578	2.369982	0.756635
9	6	0	-3.451881	1.302376	1.687381
10	1	0	-3.893723	1.963048	2.434145
11	1	0	-4.229712	0.874195	1.058838
12	1	0	-2.859392	0.531414	2.182007
13	6	0	-2.681784	-0.501496	-0.574907
14	6	0	-3.561168	0.081198	-1.479629
15	6	0	-3.041143	-1.619455	0.170395
16	6	0	-4.840445	-0.447750	-1.614446
17	1	0	-3.246054	0.937769	-2.064598
18	6	0	-4.318225	-2.144851	0.020540
19	1	0	-2.331305	-2.053317	0.866061
20	6	0	-5.219500	-1.557520	-0.864058
21	1	0	-5.535283	-0.000337	-2.315129
22	1	0	-4.608799	-3.013969	0.598457
23	1	0	-6.214616	-1.971595	-0.976289
24	6	0	2.138056	-1.213241	0.202953
25	6	0	2.653068	-1.419872	-1.079190
26	6	0	2.728133	-1.857417	1.282508
27	6	0	3.746307	-2.251284	-1.277505
28	1	0	2.192111	-0.929159	-1.932111
29	6	0	3.824019	-2.694287	1.084796
30	1	0	2.333501	-1.692940	2.277321
31	6	0	4.336873	-2.892351	-0.190858
32	1	0	4.135813	-2.401751	-2.277919
33	1	0	4.279326	-3.187834	1.935826
34	1	0	5.190714	-3.543016	-0.341587
35	8	0	-0.322966	-1.881004	-0.977602
36	6	0	2.599637	1.710195	-0.233230
37	8	0	3.546387	1.327645	0.394314
38	8	0	2.630978	2.739814	-1.087050
39	6	0	3.898094	3.400884	-1.196434
40	1	0	4.204131	3.798254	-0.228158
41	1	0	4.655307	2.706233	-1.561291
42	1	0	3.748690	4.207931	-1.909277
43	1	0	0.178089	2.983127	-0.443771

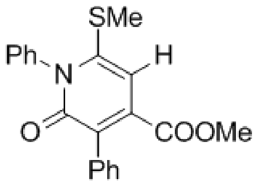

Electronic Energy (EE): -1450.86094940

Standard orientation:

Center Number	Atomic Number	Atomic Type	Coordinates (Angstroms) X	Y	Z
1	6	0	1.078041	-0.176415	0.040570
2	6	0	1.132251	1.187219	0.026408
3	6	0	-0.042467	1.987404	0.024179
4	6	0	-1.263536	1.389612	-0.035369
5	1	0	0.031140	3.064443	0.061907
6	6	0	2.279097	-1.040054	0.132980
7	6	0	3.247544	-0.801263	1.111739
8	6	0	2.469178	-2.090014	-0.768381
9	6	0	4.396388	-1.582792	1.174008
10	1	0	3.093986	-0.009185	1.838467
11	6	0	3.620421	-2.864614	-0.710142
12	1	0	1.714254	-2.292520	-1.517744
13	6	0	4.587647	-2.613541	0.259386
14	1	0	5.136766	-1.392416	1.942593
15	1	0	3.760752	-3.669144	-1.422646
16	1	0	5.482157	-3.224013	0.305981
17	16	0	-2.681268	2.435201	-0.245843
18	6	0	-3.822375	1.923067	1.079248
19	1	0	-3.265395	1.652812	1.975282
20	1	0	-4.426264	2.804875	1.295902
21	1	0	-4.467358	1.106300	0.760749
22	8	0	-0.381468	-2.048446	-0.003089
23	6	0	2.433604	1.940349	0.035664
24	8	0	2.668191	2.842067	0.797360
25	8	0	3.261477	1.526095	-0.916232
26	6	0	4.558384	2.133469	-0.921597
27	1	0	5.111979	1.633811	-1.712457
28	1	0	4.478263	3.201530	-1.127399
29	1	0	5.048987	1.982990	0.041153
30	6	0	-0.226953	-0.841724	-0.000887
31	7	0	-1.354872	0.012772	-0.049541
32	6	0	-2.633379	-0.655070	-0.105423
33	6	0	-3.365993	-0.647274	-1.283627
34	6	0	-3.103263	-1.301580	1.030571
35	6	0	-4.611234	-1.268223	-1.310570
36	1	0	-2.967618	-0.154065	-2.162182
37	6	0	-4.342057	-1.928168	0.994248
38	1	0	-2.497758	-1.309848	1.929577
39	6	0	-5.100830	-1.903878	-0.173857
40	1	0	-5.193983	-1.260819	-2.224032
41	1	0	-4.715000	-2.433918	1.876847
42	1	0	-6.068918	-2.390237	-0.200929

Route d

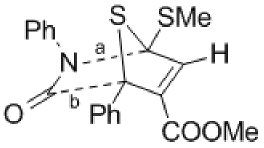

a: 2.346 Angstroms
b: 1.781 Angstroms

Frequency: 406.98i

Electronic Energy (EE): -1848.96714890

Standard orientation:

Center Number	Atomic Number	Atomic Type	Coordinates (Angstroms) X	Y	Z
1	6	0	0.137980	-0.503773	-1.277898
2	6	0	1.232132	-0.478104	0.127727
3	6	0	-0.778232	-1.003153	1.418481
4	6	0	0.636977	0.704714	0.767290
5	6	0	-0.525225	0.410720	1.409038
6	16	0	0.668192	-1.894393	1.111369
7	7	0	-1.011855	-0.983412	-0.915867
8	16	0	-2.191509	-1.786362	2.001477
9	6	0	-3.325493	-0.388618	2.237897
10	1	0	-4.301361	-0.839783	2.418443
11	1	0	-3.039244	0.209757	3.101646
12	1	0	-3.380146	0.211400	1.329168
13	6	0	-2.225067	-0.408274	-1.253329
14	6	0	-2.359907	0.967431	-1.510975
15	6	0	-3.378882	-1.208366	-1.214375
16	6	0	-3.618618	1.521715	-1.704178
17	1	0	-1.471729	1.590041	-1.556516
18	6	0	-4.631912	-0.642354	-1.410717
19	1	0	-3.263030	-2.270092	-1.032495
20	6	0	-4.761368	0.724823	-1.647203
21	1	0	-3.708855	2.583865	-1.903361
22	1	0	-5.514076	-1.272500	-1.381610
23	1	0	-5.740396	1.163899	-1.798960
24	6	0	2.687628	-0.572722	-0.210325
25	6	0	3.639261	0.072757	0.581337
26	6	0	3.112687	-1.392354	-1.255451
27	6	0	4.993805	-0.079871	0.317705
28	1	0	3.320955	0.692789	1.412611
29	6	0	4.469870	-1.546681	-1.517310
30	1	0	2.382609	-1.899848	-1.873990
31	6	0	5.412217	-0.887765	-0.735976
32	1	0	5.723153	0.430041	0.936115
33	1	0	4.787905	-2.179049	-2.337793
34	1	0	6.469426	-1.004800	-0.944554
35	8	0	0.647086	-0.062845	-2.276425
36	1	0	-1.227621	1.147549	1.770281
37	6	0	1.061722	2.100735	0.460332
38	8	0	1.901969	2.404428	-0.340145
39	8	0	0.372527	2.983916	1.193589
40	6	0	0.685259	4.360078	0.945875
41	1	0	0.046217	4.932876	1.613276
42	1	0	1.735607	4.556454	1.163300
43	1	0	0.474998	4.613769	-0.093857

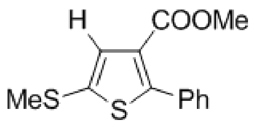

Electronic Energy (EE): -1449.34901908

Standard orientation:

Center Number	Atomic Number	Atomic Type	Coordinates (Angstroms) X	Y	Z
1	6	0	-2.326906	-0.549206	0.034632
2	6	0	-1.866484	0.732425	0.038862
3	6	0	-0.436819	0.808009	0.016356
4	6	0	0.179086	-0.418382	0.009487
5	16	0	-1.010771	-1.682355	0.029100
6	1	0	-2.489896	1.614896	0.039944
7	16	0	-3.974048	-1.160440	0.034247
8	6	0	-4.876568	0.407023	0.043947
9	1	0	-5.934393	0.144220	0.045928
10	1	0	-4.648289	0.979885	0.942751
11	1	0	-4.653668	0.987404	-0.851434
12	6	0	0.211165	2.140441	-0.048379
13	8	0	-0.376614	3.171870	0.168167
14	8	0	1.498003	2.087495	-0.396032
15	6	0	2.177308	3.343649	-0.469721
16	1	0	1.735007	3.970131	-1.245357
17	1	0	2.126575	3.863124	0.487929
18	1	0	3.208687	3.104104	-0.718211
19	6	0	1.602819	-0.806848	0.047559
20	6	0	2.116473	-1.696879	-0.898527
21	6	0	2.441210	-0.322792	1.056118
22	6	0	3.451148	-2.085349	-0.845328
23	1	0	1.473051	-2.069592	-1.688027
24	6	0	3.772061	-0.713012	1.107319
25	1	0	2.040294	0.355926	1.800251
26	6	0	4.281229	-1.594762	0.156000
27	1	0	3.840756	-2.770815	-1.588949
28	1	0	4.413116	-0.335575	1.895592
29	1	0	5.320418	-1.899821	0.199007

Routes for the Dipolar Cycloaddition of Mesoionic **6** and Methyl Phenyl Propiolate **7d**

Route a

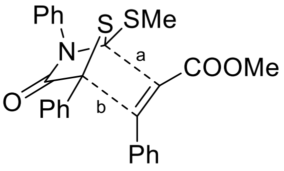

a: 2.115 Angstroms
b: 2.471 Angstroms

Frequency: 408.25i

Electronic Energy (EE): -2079.95489491

Standard orientation:

Center Number	Atomic Number	Atomic Type	Coordinates (Angstroms) X	Y	Z
1	6	0	-0.078889	-1.302907	-0.687186
2	6	0	-0.933316	-0.270418	-1.249079
3	6	0	1.359967	0.511467	-1.018393
4	6	0	-0.586820	0.803707	0.948861
5	6	0	0.524488	1.326522	0.744783
6	16	0	0.016639	0.928277	-2.048565
7	16	0	2.917635	1.297016	-1.342546
8	6	0	3.760381	0.020750	-2.331611
9	1	0	4.574995	0.535595	-2.842132
10	1	0	4.165718	-0.765443	-1.696901
11	1	0	3.075932	-0.390018	-3.073301
12	7	0	1.259615	-0.824212	-0.691839
13	8	0	-0.383141	-2.387705	-0.222658
14	6	0	-2.379480	-0.360326	-1.409273
15	6	0	-3.100829	-1.451753	-0.898793
16	6	0	-3.093703	0.696252	-1.999246
17	6	0	-4.488186	-1.474538	-0.984282
18	1	0	-2.570150	-2.268133	-0.430212
19	6	0	-4.476296	0.659421	-2.090114
20	1	0	-2.565551	1.564790	-2.379158
21	6	0	-5.182770	-0.426557	-1.577803
22	1	0	-5.027997	-2.321003	-0.575145
23	1	0	-5.003711	1.485361	-2.553472
24	1	0	-6.264577	-0.451545	-1.639812
25	6	0	1.489342	2.294502	1.261957
26	6	0	2.396713	4.428979	0.940966
27	1	0	3.412175	4.044681	0.837181
28	1	0	2.234065	5.261811	0.261259
29	1	0	2.226341	4.739645	1.972229
30	8	0	2.249240	2.098125	2.175687
31	8	0	1.444914	3.428562	0.557902
32	6	0	-1.816894	0.395450	1.545553
33	6	0	-1.872480	-0.802150	2.273861
34	6	0	-2.986907	1.146340	1.352513
35	6	0	-3.080069	-1.228111	2.811458
36	1	0	-0.969796	-1.387362	2.402519
37	6	0	-4.185905	0.711560	1.898346
38	1	0	-2.941997	2.063407	0.777165
39	6	0	-4.236448	-0.475989	2.626176
40	1	0	-3.116705	-2.153856	3.373727
41	1	0	-5.087079	1.293654	1.743646
42	1	0	-5.176973	-0.815796	3.044344
43	6	0	2.268798	-1.571377	-0.005711
44	6	0	2.561119	-2.851085	-0.471662
45	6	0	2.931911	-1.041788	1.095279
46	6	0	3.539640	-3.601975	0.165594
47	1	0	2.020810	-3.244489	-1.324021
48	6	0	3.919646	-1.799999	1.718514
49	1	0	2.688470	-0.056362	1.471502
50	6	0	4.225520	-3.075146	1.257716
51	1	0	3.768153	-4.598816	-0.192541
52	1	0	4.440724	-1.387423	2.574316
53	1	0	4.992171	-3.660750	1.751458

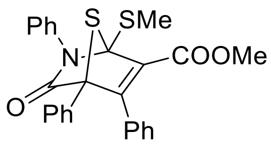

Electronic Energy (EE): -2080.03038922

Standard orientation:

Center Number	Atomic Number	Atomic Type	Coordinates (Angstroms) X	Y	Z
1	6	0	0.020095	-1.663056	0.449896
2	6	0	-1.023138	-0.964249	-0.481393
3	6	0	1.156853	-0.365869	-1.134644
4	6	0	-0.785947	0.539007	-0.157113
5	6	0	0.445001	0.880380	-0.551848
6	16	0	-0.193166	-1.140658	-2.127903
7	16	0	2.623625	-0.199775	-2.168185
8	6	0	3.802640	0.707537	-1.115075
9	1	0	4.411997	1.306542	-1.792640
10	1	0	3.298068	1.374631	-0.419086
11	1	0	4.434747	0.019810	-0.555134
12	7	0	1.271399	-1.337785	-0.021744
13	8	0	-0.228575	-2.308173	1.433879
14	6	0	-2.426191	-1.484560	-0.380065
15	6	0	-3.043104	-1.545004	0.872659
16	6	0	-3.149561	-1.854855	-1.511995
17	6	0	-4.361642	-1.966940	0.983650
18	1	0	-2.491797	-1.257181	1.758967
19	6	0	-4.471312	-2.276666	-1.399108
20	1	0	-2.694096	-1.806267	-2.495774
21	6	0	-5.080756	-2.330444	-0.151537
22	1	0	-4.828883	-2.008155	1.960565
23	1	0	-5.021656	-2.559271	-2.288923
24	1	0	-6.110499	-2.656828	-0.062663
25	6	0	1.033188	2.242740	-0.432586
26	6	0	1.984717	4.027167	-1.612462
27	1	0	2.897841	4.005753	-1.014899
28	1	0	2.214899	4.249910	-2.651410
29	1	0	1.300368	4.772298	-1.205603
30	8	0	1.193512	2.830958	0.606707
31	8	0	1.364360	2.735971	-1.623354
32	6	0	-1.809106	1.381416	0.494630
33	6	0	-1.567181	1.971956	1.736773
34	6	0	-3.048240	1.571643	-0.127861
35	6	0	-2.552099	2.742220	2.347299
36	1	0	-0.608667	1.825174	2.219016
37	6	0	-4.023243	2.350685	0.479320
38	1	0	-3.242805	1.108597	-1.088976
39	6	0	-3.778330	2.933839	1.720348
40	1	0	-2.358305	3.194363	3.313050
41	1	0	-4.977053	2.498021	-0.013640
42	1	0	-4.543708	3.535354	2.197137
43	6	0	2.417350	-1.345608	0.832813
44	6	0	2.560266	-0.371020	1.817682
45	6	0	3.403528	-2.304693	0.631322
46	6	0	3.714010	-0.346921	2.593678
47	1	0	1.778708	0.368353	1.962515
48	6	0	4.553058	-2.278768	1.414329
49	1	0	3.260822	-3.052581	-0.139651
50	6	0	4.711116	-1.297631	2.389869
51	1	0	3.833063	0.412261	3.357818
52	1	0	5.324525	-3.024680	1.262961
53	1	0	5.609055	-1.278567	2.996643

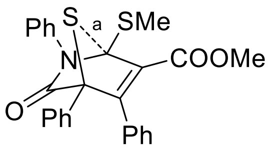

a: 2.766 Angstroms

Frequency: 240.25i

Electronic Energy (EE): -2080.00125019

Standard orientation:

Center Number	Atomic Number	Atomic Type	Coordinates (Angstroms) X	Y	Z
1	6	0	-0.724496	-1.274794	-0.435827
2	6	0	0.499096	-0.970593	0.428181
3	6	0	-1.364343	0.881223	0.309116
4	6	0	0.985990	0.437760	0.079445
5	6	0	0.012951	1.366039	0.055912
6	16	0	-0.235896	-0.861422	2.136263
7	16	0	-2.442575	1.988424	1.068544
8	6	0	-3.646970	0.906297	1.885519
9	1	0	-3.994725	1.487679	2.740254
10	1	0	-4.484835	0.660435	1.235750
11	1	0	-3.126882	0.013251	2.234635
12	7	0	-1.742226	-0.252041	-0.322309
13	8	0	-0.934687	-2.255753	-1.080667
14	6	0	1.540623	-2.058230	0.212503
15	6	0	2.068957	-2.239660	-1.069242
16	6	0	2.000896	-2.861912	1.249047
17	6	0	3.048472	-3.193833	-1.304758
18	1	0	1.716556	-1.624392	-1.891370
19	6	0	2.983801	-3.822373	1.014553
20	1	0	1.592408	-2.728288	2.242905
21	6	0	3.512503	-3.988805	-0.258541
22	1	0	3.450181	-3.317841	-2.303804
23	1	0	3.332584	-4.439902	1.834487
24	1	0	4.278597	-4.734573	-0.438283
25	6	0	0.196015	2.828422	-0.171981
26	6	0	1.591912	4.677250	0.143761
27	1	0	0.785577	5.336805	0.465760
28	1	0	2.501952	4.888007	0.699609
29	1	0	1.762349	4.803682	-0.926597
30	8	0	-0.565594	3.491230	-0.834008
31	8	0	1.270104	3.313244	0.436241
32	6	0	2.407644	0.731116	-0.189919
33	6	0	2.765710	1.407033	-1.359254
34	6	0	3.401616	0.324879	0.703730
35	6	0	4.101768	1.672056	-1.633979
36	1	0	1.995798	1.706990	-2.063662
37	6	0	4.734631	0.604597	0.432179
38	1	0	3.125487	-0.197788	1.612169
39	6	0	5.087932	1.272818	-0.737427
40	1	0	4.371431	2.186479	-2.548966
41	1	0	5.500084	0.293408	1.133217
42	1	0	6.130635	1.478422	-0.950828
43	6	0	-3.101293	-0.608349	-0.609114
44	6	0	-3.646070	-1.760606	-0.052552
45	6	0	-3.846986	0.217109	-1.442873
46	6	0	-4.971870	-2.075942	-0.321497
47	1	0	-3.039766	-2.388358	0.590768
48	6	0	-5.176817	-0.101519	-1.694163
49	1	0	-3.389331	1.096623	-1.881275
50	6	0	-5.739496	-1.245179	-1.134171
51	1	0	-5.406059	-2.970734	0.108048
52	1	0	-5.768341	0.537632	-2.338856
53	1	0	-6.774233	-1.493882	-1.339128

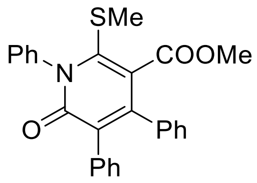

Electronic Energy (EE): -1681.87997198

Standard orientation:

Center Number	Atomic Number	Atomic Type	Coordinates (Angstroms) X	Y	Z
1	6	0	-0.572503	1.101577	0.009085
2	6	0	-0.945411	-0.215448	0.014834
3	6	0	0.058970	-1.242978	0.001648
4	6	0	1.380003	-0.915245	-0.037000
5	6	0	-1.548682	2.219996	0.095775
6	6	0	-2.471398	2.267571	1.142991
7	6	0	-1.558417	3.235656	-0.862909
8	6	0	-3.396374	3.301952	1.223427
9	1	0	-2.463195	1.489313	1.898128
10	6	0	-2.491343	4.263315	-0.788754
11	1	0	-0.833186	3.217955	-1.667772
12	6	0	-3.412364	4.299806	0.254414
13	1	0	-4.103901	3.327238	2.044164
14	1	0	-2.493835	5.041537	-1.543467
15	1	0	-4.135289	5.105356	0.314581
16	16	0	2.632371	-2.180646	-0.142322
17	6	0	2.921896	-2.439986	1.637709
18	1	0	1.999783	-2.765270	2.116637
19	1	0	3.671971	-3.228284	1.709270
20	1	0	3.312431	-1.532020	2.096246
21	8	0	1.249290	2.624705	-0.084932
22	6	0	0.840373	1.479064	-0.050780
23	6	0	-0.339580	-2.688723	0.005417
24	8	0	-0.867419	-3.047011	-1.161847
25	8	0	-0.223364	-3.428886	0.947539
26	6	0	-1.375525	-4.384939	-1.227942
27	1	0	-2.150404	-4.530582	-0.473878
28	1	0	-1.793081	-4.492077	-2.226000
29	1	0	-0.570161	-5.103263	-1.071427
30	7	0	1.760172	0.406443	-0.055788
31	6	0	3.156568	0.777483	-0.106300
32	6	0	3.818217	1.092110	1.071404
33	6	0	3.800133	0.836723	-1.333914
34	6	0	5.163164	1.444845	1.019207
35	1	0	3.279211	1.069380	2.011833
36	6	0	5.142877	1.189663	-1.379322
37	1	0	3.250846	0.598331	-2.237627
38	6	0	5.825563	1.488242	-0.203115
39	1	0	5.690645	1.690608	1.933247
40	1	0	5.656123	1.231519	-2.332623
41	1	0	6.873144	1.763637	-0.240682
42	6	0	-2.378563	-0.628152	0.012791
43	6	0	-2.879552	-1.459184	1.016179
44	6	0	-3.228125	-0.197725	-1.008307
45	6	0	-4.216009	-1.847922	1.001429
46	1	0	-2.229601	-1.788037	1.819949
47	6	0	-4.558346	-0.595500	-1.026654
48	1	0	-2.840615	0.450216	-1.786382
49	6	0	-5.056370	-1.419351	-0.020425
50	1	0	-4.598577	-2.483853	1.791322
51	1	0	-5.208333	-0.259317	-1.826076
52	1	0	-6.096392	-1.724698	-0.033186

Route c

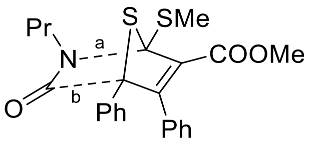

a: 2.272 Angstroms
b: 1.841 Angstroms

Frequency: 492.03i

Electronic Energy (EE): -1966.87329827

Standard orientation:

Center Number	Atomic Number	Atomic Type	Coordinates (Angstroms) X	Y	Z
1	6	0	0.023898	-1.731262	0.705412
2	6	0	-0.843038	-0.767464	-0.601402
3	6	0	1.424714	-0.306014	-1.318668
4	6	0	-0.414828	0.588196	-0.251213
5	6	0	0.864909	0.843620	-0.662146
6	16	0	0.177601	-1.306518	-1.996914
7	16	0	3.054890	-0.575919	-1.835066
8	6	0	3.987845	0.270583	-0.526248
9	1	0	4.922598	-0.279709	-0.418931
10	1	0	4.202183	1.304014	-0.788825
11	1	0	3.423145	0.204406	0.405404
12	7	0	1.262565	-1.429962	0.649382
13	8	0	-0.717306	-2.476639	1.306072
14	6	0	-2.298990	-1.099737	-0.683373
15	6	0	-3.081431	-1.087749	0.476367
16	6	0	-2.911116	-1.385042	-1.905157
17	6	0	-4.443443	-1.348805	0.406589
18	1	0	-2.622762	-0.873201	1.432399
19	6	0	-4.275194	-1.652238	-1.971557
20	1	0	-2.331452	-1.383477	-2.822050
21	6	0	-5.046052	-1.633411	-0.815506
22	1	0	-5.034954	-1.334047	1.314686
23	1	0	-4.731879	-1.869351	-2.930207
24	1	0	-6.108819	-1.840516	-0.864857
25	6	0	2.247701	-2.364024	1.161320
26	6	0	2.794214	-1.924760	2.518921
27	1	0	3.070965	-2.428359	0.436179
28	1	0	1.809931	-3.367079	1.248568
29	6	0	3.876051	-2.873217	3.025237
30	1	0	3.189252	-0.907285	2.433265
31	1	0	1.963716	-1.881590	3.229654
32	1	0	4.260898	-2.553334	3.995899
33	1	0	3.485341	-3.887861	3.139958
34	1	0	4.719238	-2.917780	2.329530
35	6	0	1.593725	2.114089	-0.396233
36	6	0	2.935745	3.773450	-1.368903
37	1	0	3.680369	3.696439	-0.574483
38	1	0	3.417460	3.936059	-2.330125
39	1	0	2.253219	4.593133	-1.142055
40	8	0	1.610428	2.707143	0.648845
41	8	0	2.210358	2.546168	-1.502703
42	6	0	-1.262909	1.469169	0.583163
43	6	0	-0.932703	1.693682	1.920351
44	6	0	-2.434121	2.015570	0.053592
45	6	0	-1.773449	2.456996	2.722439
46	1	0	-0.023384	1.263497	2.323429
47	6	0	-3.259017	2.797087	0.853373
48	1	0	-2.700110	1.820080	-0.979586
49	6	0	-2.933276	3.011595	2.189519
50	1	0	-1.519261	2.622684	3.762789
51	1	0	-4.162795	3.225638	0.436371
52	1	0	-3.583974	3.611102	2.816023

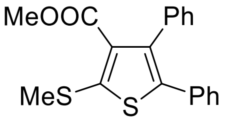

Electronic Energy (EE): -1680.37032221

Standard orientation:

Center Number	Atomic Number	Atomic Type	Coordinates (Angstroms) X	Y	Z
1	6	0	0.651020	-0.938839	-0.033220
2	6	0	0.039962	0.283636	0.033483
3	6	0	-1.397304	0.182482	0.012202
4	6	0	-1.848973	-1.117303	-0.073883
5	16	0	-0.521427	-2.222012	-0.122083
6	6	0	-2.288610	1.347808	0.210778
7	8	0	-2.000446	2.348711	0.813740
8	8	0	-3.490306	1.148696	-0.350379
9	6	0	-4.462986	2.172610	-0.124803
10	1	0	-4.636196	2.305164	0.943891
11	1	0	-4.129385	3.116275	-0.557955
12	1	0	-5.370135	1.829412	-0.616695
13	6	0	2.085039	-1.282600	-0.093094
14	6	0	2.594153	-2.335083	0.674487
15	6	0	2.954514	-0.571596	-0.928277
16	6	0	3.942808	-2.667475	0.611768
17	1	0	1.934699	-2.885958	1.336952
18	6	0	4.302428	-0.901616	-0.982451
19	1	0	2.568529	0.237587	-1.537151
20	6	0	4.801617	-1.949945	-0.213767
21	1	0	4.322460	-3.482798	1.216603
22	1	0	4.963639	-0.340912	-1.633034
23	1	0	5.853763	-2.206007	-0.259435
24	16	0	-3.512496	-1.680097	-0.113475
25	6	0	-3.287622	-3.457714	0.161748
26	1	0	-2.784712	-3.652149	1.109080
27	1	0	-4.296055	-3.869862	0.208084
28	1	0	-2.756919	-3.933203	-0.663481
29	6	0	0.792366	1.561934	0.092221
30	6	0	0.601811	2.539352	-0.886759
31	6	0	1.728489	1.783698	1.102866
32	6	0	1.342792	3.714420	-0.860917
33	1	0	-0.126637	2.373569	-1.673949
34	6	0	2.468960	2.960355	1.129112
35	1	0	1.874637	1.028695	1.867798
36	6	0	2.281013	3.926616	0.145922
37	1	0	1.189634	4.465188	-1.627768
38	1	0	3.193404	3.121827	1.919043
39	1	0	2.859185	4.843215	0.165559

Route b

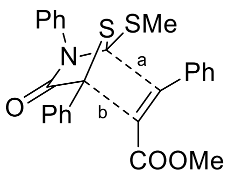

a: 2.285 Angstroms
b: 2.278 Angstroms

Frequency: 402.94i

Electronic Energy (EE): -2079.94699749

Standard orientation:

Center Number	Atomic Number	Atomic Type	Coordinates (Angstroms) X	Y	Z
1	6	0	-0.832460	-1.505793	-0.150159
2	6	0	-1.602126	-0.430135	-0.775810
3	6	0	0.762503	-0.243092	-1.326102
4	6	0	-0.670391	1.014102	0.718869
5	6	0	0.500429	1.254910	0.378855
6	16	0	-0.708715	0.257928	-2.090768
7	16	0	2.211059	-0.057384	-2.330459
8	6	0	3.617083	0.022951	-1.164452
9	1	0	3.957226	1.052954	-1.073817
10	1	0	3.347659	-0.374647	-0.190193
11	1	0	4.401907	-0.597902	-1.595941
12	7	0	0.506851	-1.365715	-0.562638
13	8	0	-1.196479	-2.360583	0.633548
14	6	0	-3.066992	-0.316125	-0.728709
15	6	0	-3.865420	-1.360135	-0.240393
16	6	0	-3.683493	0.873180	-1.142824
17	6	0	-5.247263	-1.217792	-0.198500
18	1	0	-3.397093	-2.271202	0.105463
19	6	0	-5.064777	1.006729	-1.098859
20	1	0	-3.074900	1.707230	-1.477017
21	6	0	-5.852379	-0.040267	-0.628808
22	1	0	-5.855067	-2.034986	0.172880
23	1	0	-5.523145	1.934461	-1.420722
24	1	0	-6.930632	0.064433	-0.592252
25	6	0	-1.886185	1.330381	1.455741
26	8	0	-2.446359	2.395254	1.387701
27	8	0	-2.324693	0.292403	2.165334
28	6	0	-3.624870	0.450388	2.744523
29	1	0	-4.345889	0.729244	1.973379
30	1	0	-3.876427	-0.518907	3.168126
31	1	0	-3.605255	1.214491	3.523234
32	6	0	1.727091	2.011117	0.457697
33	6	0	2.007395	2.964056	-0.529362
34	6	0	2.643901	1.801841	1.494770
35	6	0	3.192452	3.686569	-0.482862
36	1	0	1.291116	3.129655	-1.325895
37	6	0	3.829722	2.524700	1.530062
38	1	0	2.424418	1.067350	2.261433
39	6	0	4.111123	3.464007	0.540509
40	1	0	3.398362	4.425150	-1.249007
41	1	0	4.536398	2.356826	2.334633
42	1	0	5.037268	4.025792	0.571404
43	6	0	1.537030	-2.112290	0.094670
44	6	0	1.753862	-1.909024	1.453251
45	6	0	2.308345	-3.007576	-0.635139
46	6	0	2.786946	-2.593798	2.082533
47	1	0	1.111539	-1.228234	2.000400
48	6	0	3.341795	-3.686326	0.002712
49	1	0	2.100106	-3.159062	-1.687450
50	6	0	3.585030	-3.474772	1.356648
51	1	0	2.965470	-2.443948	3.140793
52	1	0	3.951613	-4.384609	-0.558215
53	1	0	4.389991	-4.006186	1.851012

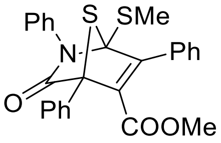

Electronic Energy (EE): -2080.02899185

Standard orientation:

Center Number	Atomic Number	Atomic Type	Coordinates (Angstroms) X	Y	Z
1	6	0	-0.641987	-1.571004	0.451343
2	6	0	-1.520938	-0.606726	-0.416262
3	6	0	0.724012	-0.466633	-1.101935
4	6	0	-0.960325	0.781607	-0.068003
5	6	0	0.307873	0.899290	-0.464262
6	16	0	-0.778877	-0.897425	-2.095009
7	16	0	2.173664	-0.566796	-2.165813
8	6	0	3.567656	-0.144972	-1.067750
9	1	0	4.310285	0.336689	-1.704931
10	1	0	3.264644	0.560556	-0.296889
11	1	0	3.989512	-1.042455	-0.618330
12	7	0	0.640679	-1.467076	-0.015531
13	8	0	-1.022176	-2.236418	1.379344
14	6	0	-2.999821	-0.816650	-0.286203
15	6	0	-3.587394	-0.729515	0.978753
16	6	0	-3.800913	-1.086781	-1.391658
17	6	0	-4.955554	-0.913974	1.128789
18	1	0	-2.970689	-0.528370	1.848057
19	6	0	-5.170982	-1.274601	-1.239703
20	1	0	-3.366705	-1.146156	-2.384030
21	6	0	-5.750806	-1.190117	0.019928
22	1	0	-5.400621	-0.847424	2.114692
23	1	0	-5.781764	-1.486350	-2.109432
24	1	0	-6.818027	-1.337134	0.139044
25	6	0	-1.823454	1.851698	0.509134
26	8	0	-2.754451	2.332034	-0.077126
27	8	0	-1.448036	2.205625	1.740050
28	6	0	-2.227659	3.248691	2.342057
29	1	0	-3.269984	2.939031	2.426420
30	1	0	-1.795984	3.406329	3.327394
31	1	0	-2.163382	4.160044	1.746639
32	6	0	1.206603	2.063163	-0.328011
33	6	0	1.719826	2.691396	-1.467999
34	6	0	1.585076	2.520900	0.937372
35	6	0	2.608742	3.751361	-1.339369
36	1	0	1.415517	2.348563	-2.449571
37	6	0	2.478382	3.580352	1.060486
38	1	0	1.178542	2.041052	1.819545
39	6	0	2.997079	4.191552	-0.076295
40	1	0	2.998587	4.235251	-2.227308
41	1	0	2.772178	3.924240	2.045415
42	1	0	3.696937	5.013516	0.020271
43	6	0	1.766164	-2.055323	0.632020
44	6	0	2.289467	-1.482485	1.787185
45	6	0	2.347649	-3.189533	0.074336
46	6	0	3.422261	-2.036616	2.373818
47	1	0	1.809802	-0.608194	2.214086
48	6	0	3.483468	-3.735884	0.660935
49	1	0	1.910100	-3.622246	-0.817379
50	6	0	4.023668	-3.156723	1.806209
51	1	0	3.836135	-1.594407	3.272413
52	1	0	3.943387	-4.615428	0.226101
53	1	0	4.908942	-3.584783	2.262035

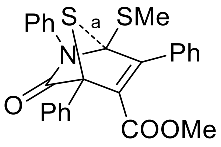

a: 2.782 Angstroms

Frequency: 212.58i

Electronic Energy (EE): -2080.00068827

Standard orientation:

Center Number	Atomic Number	Atomic Type	Coordinates (Angstroms) X	Y	Z
1	6	0	-0.338723	-1.592638	-0.498016
2	6	0	0.799866	-1.163223	0.425769
3	6	0	-1.301360	0.423330	0.318138
4	6	0	1.069204	0.305629	0.156559
5	6	0	0.013185	1.121355	0.145035
6	16	0	0.003954	-1.270459	2.098292
7	16	0	-2.681768	1.164043	1.029567
8	6	0	-1.911760	2.315698	2.201036
9	1	0	-2.655689	2.445124	2.987840
10	1	0	-1.020071	1.851968	2.623859
11	1	0	-1.691766	3.274491	1.737571
12	7	0	-1.484365	-0.689166	-0.420622
13	8	0	-0.425390	-2.589407	-1.144346
14	6	0	2.032451	-2.021046	0.184984
15	6	0	2.560890	-2.083524	-1.108157
16	6	0	2.688649	-2.682741	1.216650
17	6	0	3.729487	-2.790050	-1.362302
18	1	0	2.055070	-1.578367	-1.925359
19	6	0	3.858366	-3.394738	0.961418
20	1	0	2.283776	-2.627960	2.219198
21	6	0	4.383874	-3.447836	-0.323508
22	1	0	4.124898	-2.831948	-2.370784
23	1	0	4.361022	-3.904390	1.775573
24	1	0	5.295828	-4.000532	-0.518717
25	6	0	2.479327	0.785028	-0.024168
26	8	0	3.292875	0.803262	0.857273
27	8	0	2.706799	1.170053	-1.277650
28	6	0	4.044802	1.608955	-1.550934
29	1	0	4.752894	0.806485	-1.340037
30	1	0	4.058721	1.864572	-2.607431
31	1	0	4.286244	2.482700	-0.944296
32	6	0	0.074234	2.584258	-0.080558
33	6	0	0.984012	3.360641	0.643569
34	6	0	-0.761120	3.201582	-1.014934
35	6	0	1.061672	4.731766	0.428112
36	1	0	1.608292	2.891518	1.396484
37	6	0	-0.684022	4.573431	-1.223963
38	1	0	-1.464415	2.606469	-1.587864
39	6	0	0.227094	5.341421	-0.503493
40	1	0	1.765803	5.324985	0.999807
41	1	0	-1.332468	5.042378	-1.954840
42	1	0	0.283742	6.411209	-0.667081
43	6	0	-2.791152	-1.278683	-0.562528
44	6	0	-3.143202	-2.355950	0.242849
45	6	0	-3.672255	-0.742617	-1.493077
46	6	0	-4.416114	-2.897418	0.114229
47	1	0	-2.426095	-2.746042	0.957514
48	6	0	-4.944893	-1.290761	-1.609335
49	1	0	-3.358147	0.086909	-2.116625
50	6	0	-5.315052	-2.366078	-0.807343
51	1	0	-4.704500	-3.738125	0.733724
52	1	0	-5.641546	-0.884580	-2.332922
53	1	0	-6.305054	-2.796052	-0.905411

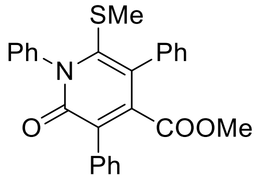

Electronic Energy (EE): -1681.87680273

Standard orientation:

Center Number	Atomic Number	Atomic Type	Coordinates (Angstroms) X	Y	Z
1	6	0	-0.777243	-1.246991	-0.056217
2	6	0	-1.098119	0.077347	-0.041234
3	6	0	-0.115015	1.117513	-0.017461
4	6	0	1.204351	0.753864	0.021466
5	6	0	-1.800751	-2.316622	-0.155824
6	6	0	-2.767082	-2.259498	-1.163674
7	6	0	-1.825213	-3.382426	0.748828
8	6	0	-3.756515	-3.233389	-1.251342
9	1	0	-2.734094	-1.459220	-1.894237
10	6	0	-2.816670	-4.351338	0.661413
11	1	0	-1.067396	-3.446684	1.519918
12	6	0	-3.786415	-4.278864	-0.336108
13	1	0	-4.495772	-3.176667	-2.042084
14	1	0	-2.831019	-5.169221	1.372673
15	1	0	-4.555268	-5.040076	-0.404141
16	16	0	2.433429	2.027807	0.176723
17	6	0	3.588669	1.669121	-1.187002
18	1	0	3.057703	1.210542	-2.020123
19	1	0	3.971707	2.638318	-1.507858
20	1	0	4.411384	1.036521	-0.859030
21	8	0	1.011321	-2.800233	0.028212
22	6	0	-2.543840	0.503716	-0.082103
23	8	0	-3.071368	1.007499	-1.036401
24	8	0	-3.148489	0.266457	1.077933
25	6	0	-4.535160	0.626022	1.132877
26	1	0	-4.870866	0.350311	2.129315
27	1	0	-4.652333	1.698426	0.970560
28	1	0	-5.095279	0.075940	0.375615
29	6	0	0.625564	-1.646036	0.003027
30	7	0	1.560092	-0.583785	0.037893
31	6	0	2.941842	-0.993015	0.141437
32	6	0	3.636393	-0.779692	1.323368
33	6	0	3.547602	-1.609993	-0.945893
34	6	0	4.974490	-1.153258	1.402071
35	1	0	3.137460	-0.314138	2.164684
36	6	0	4.880066	-1.992842	-0.856735
37	1	0	2.974479	-1.788835	-1.848182
38	6	0	5.597162	-1.755436	0.313109
39	1	0	5.525625	-0.978027	2.318344
40	1	0	5.358083	-2.475485	-1.700843
41	1	0	6.638441	-2.048846	0.378955
42	6	0	-0.568527	2.539651	-0.012746
43	6	0	-0.497814	3.317266	-1.169618
44	6	0	-1.131706	3.082433	1.141704
45	6	0	-0.974283	4.621224	-1.168191
46	1	0	-0.073857	2.890657	-2.072151
47	6	0	-1.609783	4.390794	1.142872
48	1	0	-1.185810	2.481488	2.043804
49	6	0	-1.531583	5.161415	-0.011057
50	1	0	-0.919462	5.215581	-2.073083
51	1	0	-2.038759	4.806422	2.047479
52	1	0	-1.904700	6.178990	-0.012183

<u>Route d</u>

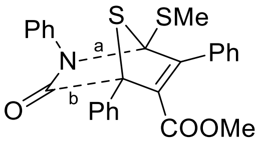

a: 2.468 Angstroms
b: 1.705 Angstroms

Frequency: 221.72i

Electronic Energy (EE): -2079.98762230

Standard orientation:

Center Number	Atomic Number	Atomic Type	Coordinates (Angstroms) X	Y	Z
1	6	0	-0.474350	-1.589550	0.625419
2	6	0	-1.345192	-0.552941	-0.410680
3	6	0	0.828821	0.044580	-1.386168
4	6	0	-0.906204	0.819120	-0.103488
5	6	0	0.315717	1.143407	-0.593637
6	16	0	-0.444864	-0.990005	-1.948650
7	16	0	2.394549	-0.246519	-2.011501
8	6	0	3.522484	0.692791	-0.940576
9	1	0	4.467852	0.154582	-1.014776
10	1	0	3.642473	1.719391	-1.278187
11	1	0	3.164707	0.649216	0.084881
12	7	0	0.763542	-1.184439	0.753203
13	8	0	-1.104109	-2.548763	1.018897
14	6	0	-2.834768	-0.788999	-0.470269
15	6	0	-3.554790	-1.125266	0.680450
16	6	0	-3.528996	-0.592898	-1.666670
17	6	0	-4.936330	-1.270026	0.623343
18	1	0	-3.034846	-1.283145	1.614155
19	6	0	-4.910165	-0.745897	-1.721104
20	1	0	-2.992084	-0.309896	-2.565087
21	6	0	-5.618500	-1.086026	-0.575039
22	1	0	-5.480342	-1.537385	1.522038
23	1	0	-5.428567	-0.593315	-2.660420
24	1	0	-6.694841	-1.207610	-0.614200
25	6	0	-1.704260	1.698680	0.799719
26	8	0	-2.089686	2.796355	0.504472
27	8	0	-1.946526	1.088454	1.957220
28	6	0	-2.837617	1.768983	2.850846
29	1	0	-3.810126	1.906615	2.376098
30	1	0	-2.927061	1.124562	3.721820
31	1	0	-2.424436	2.737430	3.134252
32	6	0	1.061209	2.377464	-0.266412
33	6	0	1.466156	3.242450	-1.283797
34	6	0	1.346874	2.686453	1.064314
35	6	0	2.157102	4.407261	-0.971433
36	1	0	1.233163	3.003573	-2.316461
37	6	0	2.043872	3.849524	1.372663
38	1	0	1.041865	1.999234	1.847822
39	6	0	2.449934	4.709272	0.356147
40	1	0	2.463163	5.081171	-1.763023
41	1	0	2.270550	4.084261	2.406236
42	1	0	2.991421	5.616434	0.598089
43	6	0	1.792113	-2.111835	0.855100
44	6	0	1.741698	-3.373978	0.238338
45	6	0	2.972092	-1.740177	1.519976
46	6	0	2.838739	-4.225761	0.289292
47	1	0	0.837886	-3.680507	-0.274887
48	6	0	4.058071	-2.601879	1.575810
49	1	0	3.006815	-0.770097	2.004768
50	6	0	4.001071	-3.849858	0.956501
51	1	0	2.781648	-5.194256	-0.195284
52	1	0	4.954054	-2.301628	2.108222
53	1	0	4.850823	-4.520891	0.997361

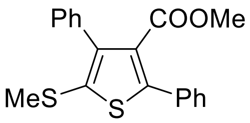

Electronic Energy (EE): -1680.36995675

Standard orientation:

Center Number	Atomic Number	Atomic Type	Coordinates (Angstroms) X	Y	Z
1	6	0	-1.011726	-1.659637	-0.168038
2	6	0	-1.028435	-0.289410	-0.063924
3	6	0	0.300712	0.244028	0.028625
4	6	0	1.288343	-0.708596	-0.006593
5	16	0	0.600151	-2.286838	-0.112530
6	16	0	-2.362898	-2.783332	-0.307022
7	6	0	-3.196905	-2.473934	1.281415
8	1	0	-4.012149	-3.195195	1.347699
9	1	0	-2.502083	-2.633283	2.105054
10	1	0	-3.603634	-1.463301	1.309113
11	6	0	0.601020	1.705031	0.082448
12	8	0	0.197780	2.460189	0.929374
13	8	0	1.383992	2.079490	-0.930398
14	6	0	1.805717	3.446789	-0.914308
15	1	0	0.943337	4.112770	-0.958040
16	1	0	2.381406	3.654201	-0.010721
17	1	0	2.428714	3.572100	-1.796440
18	6	0	2.746338	-0.519769	0.081532
19	6	0	3.617047	-1.255363	-0.727336
20	6	0	3.276850	0.406397	0.987063
21	6	0	4.990454	-1.061053	-0.638763
22	1	0	3.215722	-1.965235	-1.442588
23	6	0	4.649205	0.603192	1.066879
24	1	0	2.612791	0.957374	1.645195
25	6	0	5.510258	-0.129241	0.254333
26	1	0	5.654559	-1.634539	-1.274919
27	1	0	5.047642	1.321382	1.774327
28	1	0	6.581220	0.022806	0.320058
29	6	0	-2.273712	0.518358	-0.087079
30	6	0	-2.617752	1.326324	1.000047
31	6	0	-3.138078	0.443480	-1.182497
32	6	0	-3.809708	2.042231	0.992404
33	1	0	-1.947036	1.391520	1.847686
34	6	0	-4.328590	1.161644	-1.188311
35	1	0	-2.873264	-0.181589	-2.027566
36	6	0	-4.668183	1.960177	-0.099741
37	1	0	-4.067376	2.663981	1.842082
38	1	0	-4.990365	1.099209	-2.044415
39	1	0	-5.596833	2.519183	-0.104432

<u>Routes for the Dipolar Cycloaddition of Mesoionic **11** and Methyl Propiolate **7b** and Methyl Phenyl Propiolate **7d**</u>

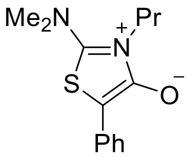

2-dimethylamino-5-phenyl-3-propylthiazol-3-ium-4-olate (**11**)

Electronic Energy (EE): -1127.12619544

Standard orientation:

Center Number	Atomic Number	Atomic Type	Coordinates (Angstroms) X	Y	Z
1	6	0	-1.449854	-0.908448	-0.078045
2	7	0	-1.348477	0.369414	-0.409350
3	6	0	0.011041	0.892277	-0.507808
4	6	0	0.915147	-0.125781	-0.179596
5	16	0	0.086303	-1.626501	0.165116
6	6	0	2.358510	-0.015800	-0.052651
7	6	0	-2.453435	1.334105	-0.415378
8	8	0	0.168306	2.076834	-0.810929
9	6	0	3.152128	-1.115224	0.321535
10	6	0	4.529844	-1.000016	0.444011
11	6	0	5.162923	0.213935	0.193368
12	6	0	4.390156	1.309635	-0.182241
13	6	0	3.010921	1.205868	-0.307186
14	1	0	-3.389600	0.812926	-0.603904
15	1	0	-2.256829	2.029062	-1.232839
16	1	0	2.692466	-2.078589	0.519945
17	1	0	5.112046	-1.867609	0.734745
18	1	0	6.238730	0.303286	0.287768
19	1	0	4.866835	2.263276	-0.381776
20	1	0	2.420281	2.063215	-0.600254
21	6	0	-2.522096	2.078486	0.915897
22	1	0	-1.558233	2.559146	1.094559
23	1	0	-2.689206	1.352116	1.717107
24	6	0	-3.639732	3.115237	0.900560
25	1	0	-3.686688	3.650784	1.850435
26	1	0	-3.479276	3.853270	0.110350
27	1	0	-4.614386	2.647557	0.733678
28	7	0	-2.632669	-1.584388	0.025354
29	6	0	-3.463761	-1.670206	-1.185081
30	1	0	-3.245684	-2.604511	-1.715846
31	1	0	-4.518910	-1.652843	-0.907906
32	1	0	-3.254321	-0.843400	-1.859831
33	6	0	-2.608378	-2.833062	0.777192
34	1	0	-2.144041	-2.677515	1.750669
35	1	0	-3.638312	-3.155395	0.932511
36	1	0	-2.074630	-3.626643	0.236685

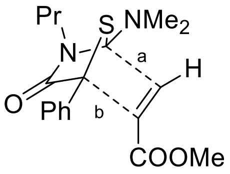

a: 2.183 Angstroms
b: 2.439 Angstroms

Frequency: 427.79i

Electronic Energy (EE): -1432.27175719

Standard orientation:

Center Number	Atomic Number	Atomic Type	Coordinates (Angstroms) X	Y	Z
1	6	0	-0.366063	0.246247	-1.133391
2	6	0	0.521180	-0.687787	-0.445641
3	6	0	-1.817054	-1.042096	0.176725
4	6	0	-0.008258	0.551353	1.587439
5	6	0	-1.159418	0.192512	1.853106
6	16	0	-0.377704	-2.024492	0.161849
7	6	0	-3.983080	-1.136435	1.358381
8	1	0	-4.866264	-1.776587	1.365520
9	1	0	-3.573299	-1.144149	2.368032
10	1	0	-4.304449	-0.117195	1.103499
11	7	0	-1.684080	-0.069525	-0.795374
12	8	0	-0.082279	1.193927	-1.853239
13	6	0	1.977667	-0.734574	-0.606791
14	6	0	2.616569	-0.016807	-1.630234
15	6	0	2.761927	-1.478383	0.287808
16	6	0	3.998643	-0.072602	-1.763393
17	1	0	2.022193	0.579929	-2.308029
18	6	0	4.142084	-1.527461	0.148001
19	1	0	2.287997	-2.000882	1.112097
20	6	0	4.766543	-0.826521	-0.880452
21	1	0	4.478777	0.479681	-2.563458
22	1	0	4.730946	-2.103676	0.852012
23	1	0	5.844556	-0.862759	-0.988137
24	6	0	-2.734981	0.884740	-1.143233
25	6	0	-2.739044	2.131820	-0.259973
26	1	0	-3.696471	0.373383	-1.096610
27	1	0	-2.550192	1.170769	-2.180410
28	6	0	-3.743382	3.150238	-0.788966
29	1	0	-2.988018	1.856897	0.768620
30	1	0	-1.732565	2.557302	-0.245527
31	1	0	-3.766436	4.042069	-0.160031
32	1	0	-3.481994	3.463524	-1.803062
33	1	0	-4.755522	2.735278	-0.816288
34	1	0	-2.007799	0.213122	2.506188
35	6	0	1.341775	1.055075	1.723383
36	8	0	2.127120	0.630788	2.535723
37	8	0	1.623610	1.999029	0.826481
38	6	0	2.993330	2.413426	0.788111
39	1	0	3.647606	1.545546	0.690836
40	1	0	3.079663	3.055316	-0.085445
41	1	0	3.249076	2.966237	1.693632
42	7	0	-3.032406	-1.707446	0.411045
43	6	0	-3.649345	-2.319691	-0.767283
44	1	0	-2.871848	-2.717981	-1.419761
45	1	0	-4.279711	-3.148574	-0.438377
46	1	0	-4.269746	-1.624851	-1.347890

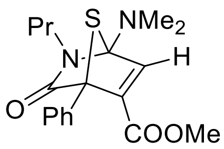

Electronic Energy (EE): -1432.36446249

Standard orientation:

Center Number	Atomic Number	Atomic Type	Coordinates (Angstroms) X	Y	Z
1	6	0	0.232059	-0.748027	1.176142
2	6	0	-0.727819	-0.539906	-0.040888
3	6	0	1.536088	-0.684939	-0.787520
4	6	0	-0.259812	0.814689	-0.613237
5	6	0	0.992174	0.719842	-1.061580
6	16	0	0.047677	-1.711733	-1.255386
7	6	0	3.820785	-0.124886	-1.565890
8	1	0	4.507932	-0.497170	-2.328535
9	1	0	3.468907	0.850018	-1.897976
10	1	0	4.388845	0.000989	-0.633824
11	7	0	1.508960	-0.875784	0.694714
12	8	0	-0.081120	-0.719003	2.342429
13	6	0	-2.201108	-0.764868	0.186920
14	6	0	-2.824946	-0.320769	1.355968
15	6	0	-2.972279	-1.398272	-0.788699
16	6	0	-4.186057	-0.525027	1.543487
17	1	0	-2.248735	0.190633	2.112758
18	6	0	-4.336265	-1.598941	-0.599914
19	1	0	-2.515146	-1.736195	-1.712547
20	6	0	-4.946832	-1.165521	0.570035
21	1	0	-4.654392	-0.175127	2.456182
22	1	0	-4.915427	-2.095716	-1.369624
23	1	0	-6.008549	-1.322516	0.722555
24	6	0	2.625283	-0.483456	1.548603
25	6	0	2.803911	1.028347	1.677769
26	1	0	3.535894	-0.948761	1.167523
27	1	0	2.427214	-0.916713	2.531782
28	6	0	3.970817	1.363638	2.600444
29	1	0	2.973085	1.468898	0.689418
30	1	0	1.875692	1.459109	2.066272
31	1	0	4.093101	2.442918	2.709665
32	1	0	3.813245	0.942178	3.596824
33	1	0	4.910141	0.958900	2.211827
34	1	0	1.564809	1.532793	-1.484336
35	6	0	-1.022481	2.081029	-0.534434
36	8	0	-1.933979	2.294294	0.220811
37	8	0	-0.545954	2.991099	-1.395272
38	6	0	-1.174150	4.275912	-1.340466
39	1	0	-1.048760	4.720397	-0.352254
40	1	0	-0.674508	4.879804	-2.094111
41	1	0	-2.237139	4.191002	-1.567862
42	7	0	2.719847	-1.073642	-1.457413
43	6	0	3.170465	-2.436059	-1.206331
44	1	0	2.321039	-3.119484	-1.205740
45	1	0	3.849182	-2.730470	-2.009178
46	1	0	3.698506	-2.549608	-0.249929

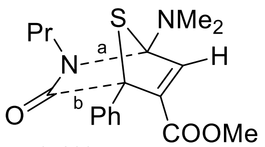

a: 2.320 Angstroms
b: 1.668 Angstroms

Frequency: 173.53i

Electronic Energy (EE): -1432.32777450

Standard orientation:

Center Number	Atomic Number	Atomic Type	Coordinates (Angstroms) X	Y	Z
1	6	0	-0.437381	0.251918	-1.327753
2	6	0	0.532670	-0.494926	-0.195122
3	6	0	-1.565125	-1.178942	0.892685
4	6	0	0.207129	0.297675	1.040322
5	6	0	-0.984072	-0.011167	1.564743
6	16	0	-0.300348	-2.105774	0.104870
7	6	0	-3.842388	-0.894852	1.678481
8	1	0	-4.435803	-1.523066	2.343265
9	1	0	-3.457489	-0.049646	2.240711
10	1	0	-4.477185	-0.526034	0.866744
11	7	0	-1.682434	-0.062016	-1.137219
12	8	0	0.132497	1.022355	-2.098397
13	6	0	1.979988	-0.608812	-0.593340
14	6	0	2.682722	0.547905	-0.950625
15	6	0	2.654833	-1.828356	-0.599667
16	6	0	4.026307	0.478272	-1.293739
17	1	0	2.166891	1.499650	-0.980151
18	6	0	3.999909	-1.898469	-0.952993
19	1	0	2.145722	-2.743269	-0.318363
20	6	0	4.691411	-0.745054	-1.297679
21	1	0	4.553966	1.384333	-1.569258
22	1	0	4.502937	-2.858462	-0.952260
23	1	0	5.739094	-0.796670	-1.571146
24	6	0	-2.672484	0.783958	-1.777819
25	6	0	-3.022519	2.003139	-0.923504
26	1	0	-3.579849	0.196904	-1.963598
27	1	0	-2.298150	1.127113	-2.751869
28	6	0	-4.080464	2.884353	-1.580265
29	1	0	-3.374117	1.658416	0.056381
30	1	0	-2.106909	2.576626	-0.746074
31	1	0	-4.328245	3.747457	-0.957900
32	1	0	-3.728093	3.260253	-2.544715
33	1	0	-5.004572	2.326101	-1.760037
34	1	0	-1.474102	0.532974	2.359320
35	6	0	1.027415	1.450180	1.527443
36	8	0	0.657789	2.593586	1.520213
37	8	0	2.203341	1.036811	1.987629
38	6	0	3.119367	2.068061	2.376095
39	1	0	2.709891	2.648787	3.203133
40	1	0	4.026408	1.553601	2.682646
41	1	0	3.322588	2.726974	1.530639
42	7	0	-2.757617	-1.691430	1.113168
43	6	0	-3.172243	-2.861038	0.343643
44	1	0	-3.196874	-2.605913	-0.720887
45	1	0	-2.478344	-3.687484	0.504433
46	1	0	-4.161307	-3.167216	0.678453

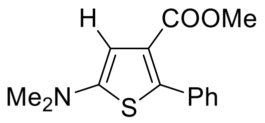

Electronic Energy (EE): -1145.80634265

Standard orientation:

Center Number	Atomic Number	Atomic Type	Coordinates (Angstroms) X	Y	Z
1	6	0	-2.408010	-0.186876	-0.087325
2	6	0	-1.797560	1.036845	-0.014185
3	6	0	-0.371343	0.944193	-0.024879
4	6	0	0.110658	-0.337948	-0.063381
5	16	0	-1.215922	-1.467053	-0.105582
6	1	0	-2.317632	1.982138	0.023205
7	6	0	-4.644782	0.640069	0.070306
8	1	0	-5.669876	0.316357	-0.107968
9	1	0	-4.559068	1.009418	1.102236
10	1	0	-4.423356	1.459658	-0.615256
11	6	0	0.439912	2.185862	-0.011652
12	8	0	0.041513	3.239558	0.421214
13	8	0	1.647824	2.027489	-0.559281
14	6	0	2.506039	3.169063	-0.515949
15	1	0	2.057357	4.007977	-1.049127
16	1	0	2.700934	3.459062	0.518039
17	1	0	3.429080	2.860742	-1.001407
18	6	0	1.481370	-0.870252	0.029251
19	6	0	1.909098	-1.889204	-0.826307
20	6	0	2.363588	-0.389564	1.003809
21	6	0	3.194648	-2.409383	-0.717232
22	1	0	1.237863	-2.261608	-1.592637
23	6	0	3.648030	-0.904899	1.106089
24	1	0	2.029425	0.383707	1.686824
25	6	0	4.068068	-1.917866	0.246457
26	1	0	3.514270	-3.196294	-1.390521
27	1	0	4.320493	-0.525000	1.866775
28	1	0	5.069362	-2.323761	0.332271
29	7	0	-3.751877	-0.476773	-0.188348
30	6	0	-4.200464	-1.766023	0.313124
31	1	0	-3.653552	-2.573830	-0.177605
32	1	0	-4.072511	-1.860021	1.400460
33	1	0	-5.255135	-1.888775	0.068227

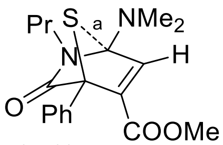

a: 2.599 Angstroms

Frequency: 144.88i

Electronic Energy (EE): -1432.35507331

Standard orientation:

Center Number	Atomic Number	Atomic Type	Coordinates (Angstroms) X	Y	Z
1	6	0	-0.467847	-0.149451	0.354641
2	6	0	1.766138	1.045465	-0.059289
3	16	0	0.282476	0.380922	1.968282
4	6	0	2.754777	3.131766	0.604708
5	1	0	2.188300	3.317022	1.524784
6	1	0	3.765626	3.520603	0.708191
7	1	0	2.273041	3.640945	-0.225796
8	6	0	2.994569	-0.936357	-1.053918
9	1	0	2.621178	-1.323728	-2.006991
10	1	0	3.715027	-0.148580	-1.282175
11	7	0	1.852683	-0.312533	-0.368983
12	6	0	0.643623	-1.004549	-0.296378
13	8	0	0.535908	-2.134938	-0.696128
14	6	0	-1.769220	-0.926109	0.410120
15	6	0	-2.339840	-1.362682	-0.789426
16	6	0	-2.434930	-1.177863	1.603477
17	6	0	-3.558607	-2.028118	-0.794562
18	1	0	-1.825691	-1.184233	-1.729380
19	6	0	-3.656749	-1.847911	1.600755
20	1	0	-1.999254	-0.834271	2.533881
21	6	0	-4.223237	-2.272447	0.405329
22	1	0	-3.985361	-2.363212	-1.733246
23	1	0	-4.165549	-2.035224	2.539674
24	1	0	-5.173196	-2.794764	0.405515
25	6	0	-0.553414	1.113844	-0.499753
26	6	0	3.645551	-2.074851	-0.274200
27	1	0	3.984734	-1.720121	0.701245
28	1	0	2.895074	-2.846202	-0.093891
29	6	0	4.817008	-2.652434	-1.062370
30	1	0	5.278679	-3.483276	-0.525868
31	1	0	4.488584	-3.027315	-2.035511
32	1	0	5.590494	-1.898269	-1.237597
33	6	0	0.603329	1.741587	-0.680830
34	1	0	0.725254	2.658017	-1.242556
35	6	0	-1.821922	1.678309	-1.054325
36	8	0	-1.943393	2.070984	-2.186602
37	8	0	-2.776219	1.722346	-0.134679
38	6	0	-4.066452	2.146626	-0.586945
39	1	0	-4.715554	2.085912	0.282788
40	1	0	-4.022967	3.170060	-0.961312
41	1	0	-4.423219	1.481534	-1.374735
42	7	0	2.836420	1.693035	0.367913
43	6	0	3.801744	0.969474	1.197974
44	1	0	3.273678	0.168035	1.722003
45	1	0	4.621178	0.559822	0.606102
46	1	0	4.209113	1.666050	1.929055

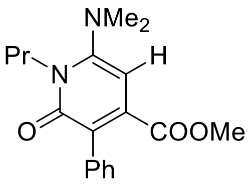

Electronic Energy (EE): -1034.22423202

Standard orientation:

Center Number	Atomic Number	Atomic Type	Coordinates (Angstroms) X	Y	Z
1	6	0	-0.610491	-0.253335	-0.198961
2	6	0	-0.419354	1.081274	0.030252
3	6	0	0.865520	1.675270	0.005672
4	6	0	1.963796	0.886874	-0.193238
5	1	0	0.966926	2.745309	0.107742
6	6	0	-1.955043	-0.869453	-0.305191
7	6	0	-2.924988	-0.282673	-1.123590
8	6	0	-2.285833	-2.022562	0.411699
9	6	0	-4.205614	-0.818441	-1.204658
10	1	0	-2.669692	0.591410	-1.715151
11	6	0	-3.566682	-2.555513	0.332724
12	1	0	-1.536336	-2.497170	1.032231
13	6	0	-4.531088	-1.955729	-0.472784
14	1	0	-4.943458	-0.354462	-1.849301
15	1	0	-3.812197	-3.445847	0.900466
16	1	0	-5.527119	-2.378594	-0.537344
17	6	0	3.823659	1.396338	-1.619619
18	1	0	3.428520	2.250512	-2.186548
19	1	0	4.911346	1.476668	-1.567466
20	1	0	3.565561	0.485271	-2.158399
21	8	0	0.501218	-2.325367	-0.514683
22	6	0	-1.564299	2.021341	0.281769
23	8	0	-1.720253	3.053359	-0.318745
24	8	0	-2.349793	1.606745	1.270643
25	6	0	-3.517625	2.399095	1.509225
26	1	0	-4.040584	1.912496	2.328628
27	1	0	-3.241021	3.417856	1.782794
28	1	0	-4.145883	2.417500	0.617284
29	6	0	0.558422	-1.111451	-0.372941
30	6	0	2.969216	-1.390240	-0.284940
31	6	0	3.160347	-1.937042	1.127445
32	1	0	3.864285	-0.861279	-0.595176
33	1	0	2.771086	-2.200541	-0.985695
34	6	0	4.282093	-2.968812	1.164733
35	1	0	3.390575	-1.101548	1.796076
36	1	0	2.224132	-2.386909	1.465679
37	1	0	4.433906	-3.346757	2.177621
38	1	0	4.048487	-3.821897	0.522179
39	1	0	5.228293	-2.539219	0.823481
40	7	0	1.813916	-0.473825	-0.342191
41	7	0	3.274192	1.379332	-0.258356
42	6	0	3.503461	2.652297	0.410017
43	1	0	4.580594	2.823585	0.450132
44	1	0	3.044270	3.500185	-0.118023
45	1	0	3.119203	2.613559	1.429214

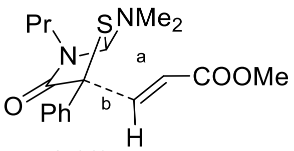

a: 3.069 Angstroms
b: 1.997 Angstroms

Frequency: 405.58i

Electronic Energy (EE): -1432.29596163

Standard orientation:

Center Number	Atomic Number	Atomic Type	Coordinates (Angstroms) X	Y	Z
1	6	0	-0.608610	1.360896	-0.145207
2	6	0	-1.348385	0.146879	0.107836
3	6	0	0.997317	0.080208	0.977851
4	6	0	-0.761507	-0.613155	-1.643504
5	6	0	0.338935	-1.198079	-1.733778
6	16	0	-0.460804	-0.806815	1.311758
7	7	0	0.765313	1.183998	0.250471
8	8	0	-0.942773	2.354974	-0.757171
9	6	0	-2.821355	0.056786	0.131114
10	6	0	-3.634814	1.145155	-0.208142
11	6	0	-3.432692	-1.172199	0.419869
12	6	0	-5.019482	1.005400	-0.229124
13	1	0	-3.181134	2.096047	-0.451510
14	6	0	-4.814100	-1.301876	0.403621
15	1	0	-2.821175	-2.041386	0.643459
16	6	0	-5.616979	-0.210930	0.079345
17	1	0	-5.633030	1.860609	-0.489583
18	1	0	-5.264712	-2.260710	0.632943
19	1	0	-6.695675	-0.312686	0.061077
20	6	0	1.754320	1.985923	-0.493670
21	6	0	1.961994	3.377255	0.093674
22	1	0	1.353178	2.071540	-1.505968
23	1	0	2.672552	1.408218	-0.568990
24	6	0	3.045515	4.125168	-0.676320
25	1	0	1.014192	3.917026	0.042873
26	1	0	2.229988	3.309066	1.152576
27	1	0	3.175683	5.136215	-0.286357
28	1	0	4.008329	3.610650	-0.605206
29	1	0	2.787586	4.205893	-1.735308
30	1	0	-1.604908	-0.278835	-2.230568
31	6	0	1.614467	-1.692904	-1.409475
32	6	0	2.880483	-3.628580	-0.970954
33	1	0	3.440071	-3.567454	-1.906418
34	1	0	3.464640	-3.153249	-0.180792
35	1	0	2.688454	-4.670655	-0.720917
36	8	0	2.661412	-1.056105	-1.415575
37	8	0	1.599444	-3.022742	-1.110102
38	7	0	2.151799	-0.335369	1.475868
39	6	0	2.243249	-1.680844	2.044111
40	1	0	3.289798	-1.980912	2.030594
41	1	0	1.883836	-1.694074	3.077927
42	1	0	1.669940	-2.386728	1.442062
43	6	0	3.370556	0.466912	1.574434
44	1	0	4.052122	0.224955	0.755925
45	1	0	3.138668	1.527163	1.576364
46	1	0	3.846530	0.226851	2.526006

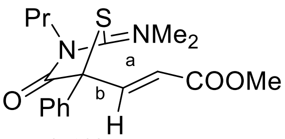

a: 3.183 Angstroms
b: 1.592 Angstroms

Electronic Energy (EE): -1432.30559621

Standard orientation:

Center Number	Atomic Number	Atomic Type	Coordinates (Angstroms) X	Y	Z
1	6	0	-0.612886	1.115245	-0.310032
2	6	0	-1.284446	-0.236244	-0.201108
3	6	0	1.027826	-0.055480	0.849544
4	6	0	-0.914382	-0.924853	-1.587475
5	6	0	0.268576	-1.346940	-1.958744
6	16	0	-0.376454	-1.053590	1.150852
7	7	0	0.742283	1.041535	0.115806
8	8	0	-1.065101	2.110016	-0.798517
9	6	0	-2.775695	-0.164947	0.026687
10	6	0	-3.561363	0.479512	-0.935522
11	6	0	-3.406822	-0.748800	1.125327
12	6	0	-4.941267	0.546794	-0.789217
13	1	0	-3.091091	0.938896	-1.795297
14	6	0	-4.789924	-0.686265	1.265415
15	1	0	-2.832894	-1.260311	1.889211
16	6	0	-5.561884	-0.035865	0.311307
17	1	0	-5.531798	1.056098	-1.541901
18	1	0	-5.259837	-1.149569	2.125053
19	1	0	-6.638931	0.013556	0.421346
20	6	0	1.641196	2.087065	-0.413694
21	6	0	1.682833	3.325270	0.474114
22	1	0	1.231693	2.345234	-1.390987
23	1	0	2.614014	1.644088	-0.598105
24	6	0	2.640544	4.363297	-0.101456
25	1	0	0.673486	3.737882	0.544530
26	1	0	1.983606	3.054392	1.491227
27	1	0	2.656450	5.265229	0.512792
28	1	0	3.661669	3.975132	-0.155834
29	1	0	2.340235	4.652408	-1.111711
30	1	0	-1.796068	-0.947203	-2.226928
31	6	0	1.594191	-1.435118	-1.534021
32	6	0	3.276596	-2.951523	-0.845650
33	1	0	3.717942	-3.065540	-1.839196
34	1	0	3.831534	-2.176806	-0.310454
35	1	0	3.345347	-3.897295	-0.307417
36	8	0	2.499292	-0.609671	-1.714054
37	8	0	1.894211	-2.646370	-0.932416
38	7	0	2.181484	-0.415062	1.369292
39	6	0	2.264371	-1.695980	2.091432
40	1	0	3.313888	-1.959347	2.193686
41	1	0	1.820980	-1.601576	3.086266
42	1	0	1.763357	-2.477983	1.521513
43	6	0	3.433026	0.349743	1.273373
44	1	0	3.896567	0.176589	0.300509
45	1	0	3.256781	1.409213	1.435387
46	1	0	4.089816	-0.002221	2.065210

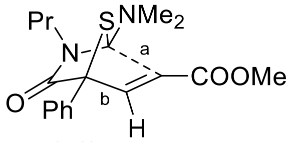

a: 2.607 Angstroms
b: 1.560 Angstroms

Frequency: 198.96i

Electronic Energy (EE): -1432.30239282

Standard orientation:

Center Number	Atomic Number	Atomic Type	Coordinates (Angstroms) X	Y	Z
1	6	0	-0.696104	1.301067	-0.300050
2	6	0	-1.337963	-0.053495	-0.097551
3	6	0	0.899124	0.170274	0.946560
4	6	0	-0.655417	-0.850455	-1.291670
5	6	0	0.555961	-1.294706	-1.182131
6	16	0	-0.590327	-0.624816	1.460310
7	7	0	0.635240	1.276415	0.191185
8	8	0	-1.123329	2.212744	-0.953598
9	6	0	-2.839909	-0.114005	-0.068473
10	6	0	-3.612229	0.973836	0.341173
11	6	0	-3.470423	-1.310115	-0.414863
12	6	0	-4.997235	0.866326	0.392835
13	1	0	-3.134549	1.907564	0.611731
14	6	0	-4.855562	-1.417440	-0.355339
15	1	0	-2.871127	-2.159095	-0.726691
16	6	0	-5.621671	-0.327624	0.044742
17	1	0	-5.589734	1.718839	0.704116
18	1	0	-5.335083	-2.351298	-0.624356
19	1	0	-6.701907	-0.407712	0.084988
20	6	0	1.616561	2.030230	-0.620268
21	6	0	1.784080	3.470212	-0.151669
22	1	0	1.233518	2.019100	-1.643533
23	1	0	2.546832	1.466718	-0.636560
24	6	0	2.853324	4.181423	-0.974379
25	1	0	0.823195	3.980624	-0.251727
26	1	0	2.044250	3.494930	0.911111
27	1	0	2.954916	5.223798	-0.667216
28	1	0	3.827911	3.698930	-0.857111
29	1	0	2.601057	4.169396	-2.037919
30	1	0	-1.280669	-0.820509	-2.186194
31	6	0	1.857056	-1.730543	-1.138694
32	6	0	3.295809	-3.576297	-0.814312
33	1	0	3.860148	-3.395192	-1.731294
34	1	0	3.829978	-3.113204	0.019613
35	1	0	3.205681	-4.648399	-0.642475
36	8	0	2.874373	-1.024024	-1.205754
37	8	0	1.967295	-3.089146	-0.921401
38	7	0	2.054830	-0.149015	1.493376
39	6	0	2.221006	-1.485421	2.069016
40	1	0	3.280085	-1.735698	2.024681
41	1	0	1.892764	-1.504779	3.112447
42	1	0	1.659648	-2.214172	1.483835
43	6	0	3.239145	0.707120	1.567818
44	1	0	3.944298	0.442302	0.777971
45	1	0	2.964434	1.755186	1.504417
46	1	0	3.699058	0.539004	2.542821

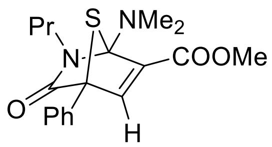

Electronic Energy (EE): -1432.36122627

Standard orientation:

Center Number	Atomic Number	Atomic Type	Coordinates (Angstroms) X	Y	Z
1	6	0	-0.786657	1.367518	0.177404
2	6	0	-1.342512	-0.088062	0.124943
3	6	0	0.922172	-0.121570	0.842518
4	6	0	-0.468998	-0.746766	-0.943344
5	6	0	0.796218	-0.816922	-0.539062
6	16	0	-0.617261	-0.783585	1.683121
7	6	0	3.401163	-0.073433	0.953192
8	1	0	4.079751	-0.902721	1.170474
9	1	0	3.317931	0.022547	-0.128363
10	1	0	3.857802	0.851709	1.323723
11	7	0	0.491858	1.301132	0.642125
12	8	0	-1.367304	2.354362	-0.210153
13	6	0	-2.825113	-0.207301	-0.057077
14	6	0	-3.397443	0.307529	-1.223246
15	6	0	-3.643811	-0.814327	0.891789
16	6	0	-4.766943	0.212871	-1.433358
17	1	0	-2.768720	0.794139	-1.960570
18	6	0	-5.016367	-0.909564	0.679797
19	1	0	-3.218495	-1.220916	1.803366
20	6	0	-5.580464	-0.396708	-0.481862
21	1	0	-5.200070	0.620263	-2.339494
22	1	0	-5.641893	-1.386135	1.425507
23	1	0	-6.649131	-0.469920	-0.646776
24	6	0	1.421833	2.380473	0.333656
25	6	0	1.872129	2.407433	-1.127009
26	1	0	2.275338	2.302389	1.008733
27	1	0	0.905916	3.313278	0.574848
28	6	0	2.920397	3.490796	-1.356756
29	1	0	2.273601	1.432006	-1.418073
30	1	0	0.995958	2.585275	-1.757451
31	1	0	3.216851	3.536861	-2.406330
32	1	0	2.536866	4.474809	-1.073500
33	1	0	3.820035	3.299223	-0.764033
34	1	0	-0.872277	-1.117605	-1.877411
35	6	0	1.837680	-1.552064	-1.302692
36	6	0	3.554017	-3.140155	-1.153845
37	1	0	4.293223	-2.454793	-1.572257
38	1	0	3.999354	-3.747021	-0.369203
39	1	0	3.159073	-3.776002	-1.946831
40	8	0	2.036528	-1.424845	-2.483459
41	8	0	2.497434	-2.409506	-0.523613
42	7	0	2.104059	-0.317435	1.587059
43	6	0	2.067301	0.135560	2.970274
44	1	0	2.988670	-0.185160	3.459172
45	1	0	1.982086	1.227780	3.062927
46	1	0	1.232011	-0.320458	3.503198

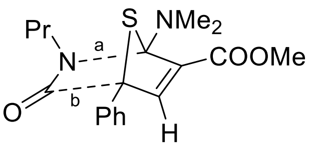

a: 2.239 Angstroms
b: 1.658 Angstroms

Frequency: 229.31i

Electronic Energy (EE): -1432.33069243

Standard orientation:

Center Number	Atomic Number	Atomic Type	Coordinates (Angstroms) X	Y	Z
1	6	0	-0.748999	1.460445	0.169222
2	6	0	-1.261902	-0.115234	0.130863
3	6	0	0.993176	-0.652111	0.913190
4	6	0	-0.404067	-0.701633	-0.934420
5	6	0	0.847890	-0.959117	-0.530617
6	16	0	-0.582701	-0.832935	1.686896
7	6	0	3.361593	-0.186352	1.005533
8	1	0	3.687436	0.707212	1.544322
9	1	0	4.129071	-0.958149	1.075402
10	1	0	3.205876	0.086488	-0.035117
11	7	0	0.461009	1.505323	0.637434
12	8	0	-1.518114	2.305422	-0.281875
13	6	0	-2.742743	-0.276695	-0.053524
14	6	0	-3.321230	0.222048	-1.224264
15	6	0	-3.560011	-0.891096	0.893102
16	6	0	-4.687066	0.099909	-1.441885
17	1	0	-2.700837	0.728633	-1.953593
18	6	0	-4.930057	-1.010229	0.675546
19	1	0	-3.140760	-1.290561	1.810477
20	6	0	-5.497369	-0.516577	-0.492297
21	1	0	-5.120689	0.496912	-2.352487
22	1	0	-5.550100	-1.492030	1.422556
23	1	0	-6.563763	-0.608515	-0.662499
24	6	0	1.189651	2.752290	0.500877
25	6	0	1.795792	2.927377	-0.892586
26	1	0	1.986789	2.785290	1.253005
27	1	0	0.516228	3.596438	0.703629
28	6	0	2.567067	4.236463	-1.023744
29	1	0	2.454437	2.078064	-1.106545
30	1	0	0.984658	2.890743	-1.626148
31	1	0	2.998089	4.350099	-2.021043
32	1	0	1.911589	5.093095	-0.844432
33	1	0	3.385405	4.288323	-0.298990
34	1	0	-0.758589	-0.830918	-1.950549
35	6	0	1.848598	-1.580854	-1.431114
36	6	0	3.741787	-2.958231	-1.555681
37	1	0	4.331746	-2.204732	-2.079775
38	1	0	4.364178	-3.512222	-0.857051
39	1	0	3.291091	-3.636345	-2.280519
40	8	0	1.838377	-1.470458	-2.629004
41	8	0	2.729567	-2.330462	-0.760190
42	7	0	2.111365	-0.645282	1.611277
43	6	0	2.042469	-0.499838	3.061341
44	1	0	1.379478	-1.252347	3.488454
45	1	0	3.040203	-0.649127	3.471244
46	1	0	1.678954	0.500908	3.319651

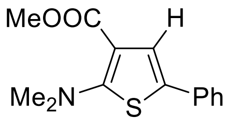

Electronic Energy (EE): -1145.80731429

Standard orientation:

Center Number	Atomic Number	Atomic Type	Coordinates (Angstroms) X	Y	Z
1	6	0	-1.133530	-0.036617	0.064562
2	6	0	-0.198933	-1.005757	0.240141
3	6	0	1.154469	-0.552255	0.147687
4	6	0	1.245232	0.817428	-0.057338
5	16	0	-0.360240	1.516734	-0.174645
6	1	0	-0.437760	-2.047438	0.410461
7	6	0	2.243720	-1.538291	0.132702
8	8	0	2.115312	-2.691875	0.471776
9	8	0	3.398233	-1.052069	-0.351740
10	6	0	4.484900	-1.977767	-0.403059
11	1	0	4.718209	-2.353189	0.594599
12	1	0	4.243365	-2.818086	-1.055197
13	1	0	5.328930	-1.420805	-0.804554
14	6	0	-2.596210	-0.159786	0.021835
15	6	0	-3.426214	0.922500	0.336736
16	6	0	-3.191207	-1.379678	-0.325205
17	6	0	-4.809440	0.789789	0.305969
18	1	0	-2.989486	1.871947	0.628292
19	6	0	-4.572953	-1.513064	-0.341411
20	1	0	-2.565789	-2.221461	-0.599556
21	6	0	-5.389499	-0.429053	-0.028056
22	1	0	-5.433713	1.640232	0.555206
23	1	0	-5.015131	-2.464905	-0.612574
24	1	0	-6.467801	-0.534212	-0.047675
25	6	0	2.181457	2.984157	-0.596102
26	1	0	1.675126	3.630352	0.135930
27	1	0	3.169488	3.399895	-0.796940
28	1	0	1.616819	2.986521	-1.529187
29	7	0	2.339898	1.627766	-0.103693
30	6	0	3.408265	1.466112	0.879142
31	1	0	3.457567	2.365591	1.503475
32	1	0	3.203918	0.613874	1.522936
33	1	0	4.372104	1.314811	0.389061

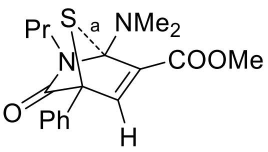

a: 2.606 Angstroms

Frequency: 164.61i

Electronic Energy (EE): -1432.35921390

Standard orientation:

Center Number	Atomic Number	Atomic Type	Coordinates (Angstroms) X	Y	Z
1	6	0	-1.156598	0.012801	0.401968
2	6	0	1.412660	-0.015781	0.434913
3	16	0	-0.548872	-0.345417	2.118147
4	6	0	2.988962	0.932878	1.983368
5	1	0	2.633635	1.898018	1.644128
6	1	0	2.594992	0.746225	2.988609
7	1	0	4.078108	0.936284	2.000287
8	6	0	1.711107	-1.938701	-1.199679
9	1	0	1.346431	-1.771040	-2.217758
10	1	0	2.739930	-1.576548	-1.163098
11	7	0	0.903824	-1.087469	-0.309160
12	6	0	-0.481687	-1.068315	-0.475155
13	8	0	-1.044146	-1.823029	-1.225072
14	6	0	-2.656573	0.023347	0.184141
15	6	0	-3.151825	0.428316	-1.058965
16	6	0	-3.555883	-0.366090	1.170809
17	6	0	-4.518027	0.454179	-1.306190
18	1	0	-2.462282	0.715570	-1.847145
19	6	0	-4.926998	-0.342992	0.924326
20	1	0	-3.176753	-0.684010	2.134227
21	6	0	-5.412269	0.068389	-0.310776
22	1	0	-4.884037	0.770735	-2.276240
23	1	0	-5.614970	-0.648154	1.704509
24	1	0	-6.479333	0.086300	-0.500711
25	6	0	-0.499589	1.312270	0.004625
26	6	0	1.625769	-3.427389	-0.876372
27	1	0	1.876358	-3.600596	0.172456
28	1	0	0.593746	-3.753260	-1.015509
29	6	0	2.559354	-4.224945	-1.781895
30	1	0	2.470555	-5.295391	-1.588270
31	1	0	2.324018	-4.057864	-2.836493
32	1	0	3.604998	-3.943470	-1.624115
33	6	0	0.835387	1.313485	0.035464
34	1	0	-1.083938	2.169462	-0.309698
35	6	0	1.689381	2.377420	-0.534108
36	8	0	2.855846	2.235565	-0.812234
37	8	0	1.012410	3.509253	-0.734996
38	6	0	1.746182	4.568047	-1.361597
39	1	0	2.099892	4.254699	-2.344366
40	1	0	1.044579	5.392841	-1.456725
41	1	0	2.596023	4.860409	-0.744256
42	7	0	2.528024	-0.149437	1.117625
43	6	0	2.924235	-1.483884	1.568788
44	1	0	2.022958	-2.018454	1.888827
45	1	0	3.437860	-2.039724	0.786259
46	1	0	3.601845	-1.369540	2.411885

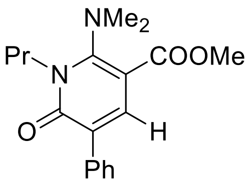

Electronic Energy (EE): -1034.22694940

Standard orientation:

Center Number	Atomic Number	Atomic Type	Coordinates (Angstroms) X	Y	Z
1	6	0	1.319448	-0.020361	0.098267
2	6	0	0.574599	1.093312	-0.138048
3	6	0	-0.842582	1.104519	-0.041799
4	6	0	-1.505781	-0.083016	0.206548
5	6	0	2.800800	0.022300	0.058606
6	6	0	3.474964	1.117871	0.609156
7	6	0	3.552705	-0.993550	-0.541720
8	6	0	4.860865	1.206865	0.548666
9	1	0	2.908779	1.897656	1.107498
10	6	0	4.938196	-0.901564	-0.603828
11	1	0	3.048833	-1.854878	-0.959805
12	6	0	5.598134	0.196813	-0.060279
13	1	0	5.363658	2.060450	0.988783
14	1	0	5.504816	-1.694570	-1.078644
15	1	0	6.679289	0.261055	-0.104517
16	6	0	-3.568928	-0.365174	1.476479
17	1	0	-3.912027	0.619866	1.813940
18	1	0	-4.430844	-1.031723	1.378110
19	1	0	-2.897310	-0.770913	2.233741
20	8	0	1.208520	-2.339561	0.597369
21	6	0	0.644128	-1.274586	0.400206
22	6	0	-1.429683	-2.543354	0.503726
23	6	0	-1.551964	-3.179479	-0.877680
24	1	0	-2.409084	-2.435785	0.957497
25	1	0	-0.810360	-3.160451	1.151674
26	6	0	-2.173591	-4.568559	-0.783624
27	1	0	-2.165280	-2.530221	-1.510805
28	1	0	-0.557351	-3.240569	-1.327266
29	1	0	-2.268501	-5.024879	-1.770847
30	1	0	-1.559392	-5.229840	-0.166654
31	1	0	-3.172191	-4.524191	-0.339475
32	1	0	1.074363	2.022874	-0.384503
33	6	0	-1.558157	2.397243	-0.035725
34	8	0	-0.895696	3.337916	-0.728237
35	8	0	-2.587832	2.631426	0.550977
36	6	0	-1.459804	4.650939	-0.682388
37	1	0	-1.482784	5.020265	0.343696
38	1	0	-0.810342	5.272659	-1.294329
39	1	0	-2.472012	4.647392	-1.087867
40	7	0	-0.772096	-1.224403	0.423185
41	7	0	-2.885711	-0.248257	0.192525
42	6	0	-3.691878	0.374673	-0.845237
43	1	0	-4.484305	-0.325008	-1.129470
44	1	0	-4.150940	1.309713	-0.512327
45	1	0	-3.081133	0.572128	-1.727242

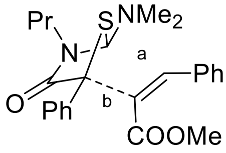

a: 3.233 Angstroms
b: 1.927 Angstroms

Frequency: 371.12i

Electronic Energy (EE): -1663.30660362

Standard orientation:

Center Number	Atomic Number	Atomic Type	Coordinates (Angstroms) X	Y	Z
1	6	0	0.569464	1.590967	0.522862
2	6	0	1.052893	0.493808	-0.312835
3	6	0	-1.417011	0.934690	-0.539179
4	6	0	0.826655	-1.052282	0.814300
5	6	0	-0.210622	-1.768570	0.759335
6	16	0	-0.209384	0.207028	-1.557751
7	7	0	-0.879504	1.648269	0.467527
8	6	0	2.430929	0.488669	-0.880435
9	6	0	2.820708	-0.619751	-1.642823
10	6	0	3.357842	1.511054	-0.657179
11	6	0	4.092779	-0.691494	-2.198240
12	1	0	2.124919	-1.441926	-1.785737
13	6	0	4.631251	1.431230	-1.208874
14	1	0	3.086801	2.353155	-0.035375
15	6	0	5.004551	0.336837	-1.984683
16	1	0	4.372575	-1.557147	-2.787905
17	1	0	5.340179	2.230967	-1.026415
18	1	0	6.000093	0.281350	-2.409581
19	8	0	1.173938	2.355313	1.234314
20	6	0	-1.457834	-2.276736	0.371111
21	6	0	-2.650111	-1.952935	1.061848
22	6	0	-1.563869	-3.180897	-0.718304
23	6	0	-3.871486	-2.478024	0.664306
24	1	0	-2.592637	-1.299246	1.925578
25	6	0	-2.791173	-3.693154	-1.100903
26	1	0	-0.663377	-3.463154	-1.251799
27	6	0	-3.963647	-3.345118	-0.423942
28	1	0	-4.765205	-2.214563	1.221723
29	1	0	-2.836514	-4.379902	-1.939707
30	1	0	-4.919002	-3.757185	-0.724849
31	7	0	-2.706426	0.776292	-0.789657
32	6	0	-3.117386	0.063672	-2.002435
33	1	0	-2.462038	0.315827	-2.836107
34	1	0	-3.109259	-1.016182	-1.827222
35	1	0	-4.124638	0.386817	-2.260260
36	6	0	-3.767414	1.044390	0.185788
37	1	0	-4.078469	2.092118	0.164740
38	1	0	-4.615613	0.416472	-0.080824
39	1	0	-3.445390	0.759593	1.183860
40	6	0	2.067205	-1.116694	1.625416
41	8	0	2.722621	-0.165032	1.963160
42	8	0	2.379633	-2.376201	1.962139
43	6	0	3.548309	-2.511421	2.771460
44	1	0	3.670057	-3.579749	2.937269
45	1	0	4.421438	-2.105492	2.258199
46	1	0	3.418297	-1.995232	3.724028
47	6	0	-1.525873	2.766902	1.174786
48	6	0	-1.851977	3.923004	0.233970
49	1	0	-0.795746	3.085545	1.917595
50	1	0	-2.406898	2.425985	1.711451
51	6	0	-2.524687	5.060979	0.994104
52	1	0	-0.922373	4.270122	-0.226368
53	1	0	-2.500474	3.577476	-0.578165
54	1	0	-2.744936	5.898986	0.330663
55	1	0	-3.466436	4.733983	1.444440
56	1	0	-1.880203	5.429610	1.796065

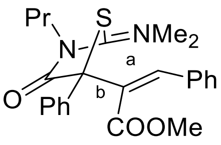

a: 3.441 Angstroms
b: 1.598 Angstroms

Electronic Energy (EE): -1663.31269066

Standard orientation:

Center Number	Atomic Number	Atomic Type	Coordinates (Angstroms) X	Y	Z
1	6	0	0.443036	1.296128	0.764243
2	6	0	1.009343	0.242985	-0.162953
3	6	0	-1.468932	0.820339	-0.494021
4	6	0	0.999211	-1.131914	0.652107
5	6	0	0.034043	-2.027231	0.720987
6	16	0	-0.225940	0.144556	-1.511383
7	7	0	-0.987068	1.381068	0.638918
8	6	0	2.343943	0.566838	-0.808821
9	6	0	3.025630	-0.470910	-1.447056
10	6	0	2.870181	1.856183	-0.850540
11	6	0	4.221584	-0.224128	-2.111407
12	1	0	2.622684	-1.478791	-1.411485
13	6	0	4.070798	2.098473	-1.509354
14	1	0	2.362907	2.672572	-0.352297
15	6	0	4.750512	1.061750	-2.140674
16	1	0	4.742001	-1.039862	-2.599997
17	1	0	4.477648	3.103013	-1.523810
18	1	0	5.687349	1.254684	-2.650367
19	8	0	1.032385	1.970106	1.553898
20	6	0	-1.262739	-2.294535	0.257200
21	6	0	-2.417736	-1.941145	1.012554
22	6	0	-1.496617	-3.071972	-0.916918
23	6	0	-3.688733	-2.328977	0.615490
24	1	0	-2.282716	-1.390494	1.938963
25	6	0	-2.772976	-3.449306	-1.292171
26	1	0	-0.643742	-3.384700	-1.509607
27	6	0	-3.896407	-3.081854	-0.541490
28	1	0	-4.538389	-2.049983	1.233007
29	1	0	-2.898464	-4.046835	-2.190247
30	1	0	-4.890111	-3.400026	-0.831323
31	7	0	-2.729015	0.761071	-0.857353
32	6	0	-3.069630	0.199020	-2.171275
33	1	0	-3.114912	-0.891297	-2.102470
34	1	0	-4.040444	0.595159	-2.462806
35	1	0	-2.341531	0.507136	-2.920870
36	6	0	-3.864230	0.975613	0.049310
37	1	0	-4.140480	2.031316	0.098240
38	1	0	-4.700105	0.402874	-0.348302
39	1	0	-3.636181	0.579957	1.034158
40	6	0	2.245566	-1.250588	1.474834
41	8	0	2.936418	-0.314641	1.804109
42	8	0	2.530278	-2.509592	1.831064
43	6	0	3.677403	-2.658394	2.663565
44	1	0	3.765875	-3.726046	2.854714
45	1	0	4.573402	-2.289058	2.161545
46	1	0	3.546767	-2.118959	3.603338
47	6	0	-1.649368	2.443749	1.421855
48	6	0	-1.858783	3.708361	0.595100
49	1	0	-0.972633	2.645866	2.250657
50	1	0	-2.578246	2.078079	1.848696
51	6	0	-2.551982	4.784882	1.422805
52	1	0	-0.884134	4.063582	0.247426
53	1	0	-2.450548	3.479549	-0.297645
54	1	0	-2.696465	5.694792	0.838126
55	1	0	-3.534062	4.448358	1.767190
56	1	0	-1.959196	5.044067	2.303558

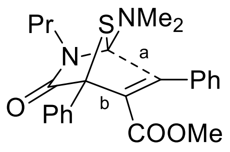

a: 2.895 Angstroms
b: 1.637 Angstroms

Frequency: 70.17i

Electronic Energy (EE): -1663.31247130

Standard orientation:

Center Number	Atomic Number	Atomic Type	Coordinates (Angstroms) X	Y	Z
1	6	0	0.590674	1.455813	0.033984
2	6	0	1.212236	0.165999	-0.431922
3	6	0	-1.078840	0.613748	-1.357468
4	6	0	0.690465	-0.945015	0.650641
5	6	0	-0.534923	-1.347419	0.701712
6	16	0	0.330171	-0.209279	-1.988148
7	6	0	-3.540933	0.737657	-1.221982
8	1	0	-4.142845	-0.174472	-1.206968
9	1	0	-3.375783	1.032834	-0.193147
10	1	0	-4.067112	1.519217	-1.774908
11	7	0	-0.756445	1.525644	-0.402646
12	8	0	1.070609	2.307030	0.734952
13	6	0	2.700698	0.127573	-0.679754
14	6	0	3.514934	1.259634	-0.653973
15	6	0	3.271316	-1.118531	-0.956459
16	6	0	4.878698	1.140970	-0.902005
17	1	0	3.092813	2.227930	-0.422954
18	6	0	4.634310	-1.232034	-1.206436
19	1	0	2.646193	-2.006721	-0.966680
20	6	0	5.443166	-0.100694	-1.177440
21	1	0	5.503092	2.026650	-0.876785
22	1	0	5.061476	-2.205237	-1.417990
23	1	0	6.506693	-0.185997	-1.367640
24	6	0	-1.515341	2.741743	-0.058259
25	6	0	-2.055055	2.740399	1.366612
26	1	0	-2.306019	2.872313	-0.796119
27	1	0	-0.813031	3.569840	-0.178197
28	6	0	-2.755451	4.060967	1.670033
29	1	0	-2.744148	1.904527	1.510097
30	1	0	-1.219912	2.585722	2.053927
31	1	0	-3.134493	4.073646	2.693035
32	1	0	-2.069895	4.904919	1.557990
33	1	0	-3.603266	4.223762	0.998062
34	6	0	1.756015	-1.354177	1.621646
35	8	0	2.009409	-2.479367	1.963557
36	8	0	2.397296	-0.275903	2.099226
37	6	0	3.558324	-0.536071	2.886855
38	1	0	4.298694	-1.078517	2.294615
39	1	0	3.947723	0.438442	3.172853
40	1	0	3.304417	-1.117940	3.773506
41	6	0	-1.879763	-1.625233	0.687445
42	6	0	-2.469884	-2.550591	-0.230838
43	6	0	-2.773610	-0.987781	1.607890
44	6	0	-3.834262	-2.777194	-0.238209
45	1	0	-1.822632	-3.088668	-0.914966
46	6	0	-4.133345	-1.231824	1.570520
47	1	0	-2.357059	-0.327112	2.359616
48	6	0	-4.696903	-2.112949	0.641146
49	1	0	-4.238965	-3.494078	-0.946621
50	1	0	-4.772345	-0.732775	2.293158
51	1	0	-5.761362	-2.310221	0.633200
52	7	0	-2.270516	0.419326	-1.882254
53	6	0	-2.399801	-0.390415	-3.096978
54	1	0	-2.389509	-1.453436	-2.849580
55	1	0	-3.350338	-0.136469	-3.564160
56	1	0	-1.601257	-0.156001	-3.801126

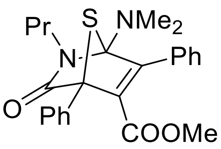

Electronic Energy (EE): -1663.38704811

Standard orientation:

Center Number	Atomic Number	Atomic Type	Coordinates (Angstroms) X	Y	Z
1	6	0	-0.791939	-1.612557	0.737018
2	6	0	-1.341232	-0.579078	-0.278725
3	6	0	0.904373	-1.134576	-0.843445
4	6	0	-0.423595	0.638185	-0.059504
5	6	0	0.835143	0.368265	-0.420182
6	16	0	-0.651435	-1.253333	-1.864877
7	6	0	3.380250	-1.439726	-0.908456
8	1	0	4.107564	-1.152110	-1.672708
9	1	0	3.387542	-0.672401	-0.140291
10	1	0	3.717010	-2.381756	-0.459990
11	7	0	0.473639	-1.941331	0.344202
12	8	0	-1.339608	-1.990823	1.748808
13	6	0	-2.818478	-0.310788	-0.366614
14	6	0	-3.771450	-1.103092	0.270984
15	6	0	-3.240916	0.762843	-1.157884
16	6	0	-5.126040	-0.810787	0.128877
17	1	0	-3.459203	-1.932375	0.890068
18	6	0	-4.591979	1.052384	-1.294564
19	1	0	-2.506358	1.370454	-1.679138
20	6	0	-5.540602	0.265023	-0.646780
21	1	0	-5.859064	-1.430610	0.632320
22	1	0	-4.902580	1.888787	-1.910011
23	1	0	-6.596229	0.487993	-0.751116
24	6	0	1.385452	-2.520928	1.321863
25	6	0	1.939595	-1.512005	2.327266
26	1	0	2.188106	-3.032485	0.790424
27	1	0	0.814291	-3.285634	1.854146
28	6	0	2.870230	-2.190654	3.326727
29	1	0	2.474822	-0.714342	1.803611
30	1	0	1.097856	-1.044416	2.848001
31	1	0	3.261660	-1.475854	4.053180
32	1	0	2.346019	-2.975711	3.878634
33	1	0	3.723306	-2.651386	2.819524
34	6	0	-0.916840	1.914935	0.517440
35	8	0	-0.643857	3.014454	0.112735
36	8	0	-1.723226	1.677593	1.554893
37	6	0	-2.384855	2.820511	2.105948
38	1	0	-2.980055	3.315679	1.337013
39	1	0	-3.028516	2.436024	2.893156
40	1	0	-1.659192	3.524002	2.515816
41	6	0	1.974308	1.306066	-0.407358
42	6	0	2.679983	1.551439	-1.589292
43	6	0	2.344361	1.967426	0.765080
44	6	0	3.743352	2.444765	-1.592241
45	1	0	2.387728	1.037292	-2.498166
46	6	0	3.413906	2.855951	0.759153
47	1	0	1.793932	1.779347	1.681268
48	6	0	4.115582	3.094934	-0.418123
49	1	0	4.280838	2.636960	-2.513664
50	1	0	3.696086	3.364577	1.673629
51	1	0	4.948256	3.788754	-0.422402
52	7	0	2.060144	-1.576101	-1.536749
53	6	0	1.926885	-2.864635	-2.207230
54	1	0	2.855787	-3.067084	-2.742601
55	1	0	1.738051	-3.689715	-1.505897
56	1	0	1.119393	-2.841842	-2.940187

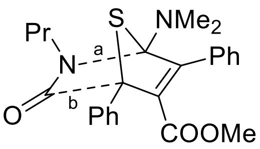

a: 2.321 Angstroms
b: 1.644 Angstroms

Frequency: 159.54i

Electronic Energy (EE): -1663.35339907

Standard orientation:

Center Number	Atomic Number	Atomic Type	Coordinates (Angstroms) X	Y	Z
1	6	0	-0.882511	-1.551838	0.772836
2	6	0	-1.331996	-0.432860	-0.344225
3	6	0	0.849118	-0.814039	-1.423930
4	6	0	-0.353786	0.688556	-0.134048
5	6	0	0.867158	0.452189	-0.644482
6	16	0	-0.785920	-1.148805	-1.959350
7	6	0	3.199037	-1.436965	-1.280765
8	1	0	3.448433	-2.475381	-1.050085
9	1	0	3.960267	-1.017430	-1.941091
10	1	0	3.168343	-0.876632	-0.352511
11	7	0	0.286303	-2.033317	0.469391
12	8	0	-1.644334	-1.734543	1.724536
13	6	0	-2.792914	-0.069076	-0.415725
14	6	0	-3.783556	-0.989055	-0.070266
15	6	0	-3.174428	1.180266	-0.913952
16	6	0	-5.127478	-0.651806	-0.197388
17	1	0	-3.500322	-1.957527	0.317635
18	6	0	-4.517152	1.518487	-1.032647
19	1	0	-2.419686	1.892958	-1.233075
20	6	0	-5.499207	0.602541	-0.668704
21	1	0	-5.886661	-1.373739	0.081065
22	1	0	-4.794485	2.493780	-1.415687
23	1	0	-6.547616	0.862879	-0.759078
24	6	0	0.949922	-2.854672	1.460602
25	6	0	1.767861	-2.023511	2.451406
26	1	0	1.614366	-3.563660	0.951653
27	1	0	0.207637	-3.442049	2.019017
28	6	0	2.479312	-2.889810	3.486071
29	1	0	2.500014	-1.427234	1.893683
30	1	0	1.090826	-1.319244	2.946277
31	1	0	3.073068	-2.286202	4.176567
32	1	0	1.759601	-3.461874	4.077891
33	1	0	3.154321	-3.603953	3.004418
34	6	0	-0.687944	1.881817	0.699242
35	8	0	-0.413697	3.015564	0.401975
36	8	0	-1.347749	1.524503	1.793234
37	6	0	-1.832624	2.593874	2.612341
38	1	0	-2.523623	3.216630	2.042090
39	1	0	-2.348251	2.114784	3.440504
40	1	0	-1.003744	3.202722	2.975187
41	6	0	2.073506	1.285168	-0.454530
42	6	0	2.816932	1.712530	-1.557651
43	6	0	2.484764	1.640381	0.831751
44	6	0	3.953809	2.489413	-1.374147
45	1	0	2.496793	1.440905	-2.558514
46	6	0	3.624721	2.414496	1.012339
47	1	0	1.915503	1.295032	1.689131
48	6	0	4.362000	2.838272	-0.089193
49	1	0	4.520744	2.825698	-2.234597
50	1	0	3.938141	2.684990	2.013845
51	1	0	5.251330	3.441227	0.052912
52	7	0	1.889250	-1.435654	-1.940063
53	6	0	1.665865	-2.567213	-2.835666
54	1	0	2.619211	-2.846128	-3.281831
55	1	0	1.257713	-3.411483	-2.269420
56	1	0	0.977729	-2.292427	-3.635283

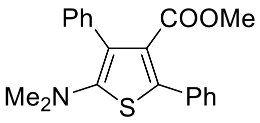

Electronic Energy (EE): -1376.82338384

Standard orientation:

Center Number	Atomic Number	Atomic Type	Coordinates (Angstroms) X	Y	Z
1	6	0	-1.064736	1.608939	0.035310
2	6	0	-1.074249	0.234165	-0.021887
3	6	0	0.255983	-0.301254	-0.086539
4	6	0	1.247292	0.644010	-0.080349
5	16	0	0.567530	2.234535	-0.041403
6	6	0	-3.009608	2.413068	-1.132057
7	1	0	-3.939409	2.947092	-0.928371
8	1	0	-2.508448	2.883985	-1.990410
9	1	0	-3.254832	1.382467	-1.385199
10	6	0	0.560760	-1.762626	-0.055281
11	8	0	0.203155	-2.563620	-0.880733
12	8	0	1.298333	-2.081408	1.010300
13	6	0	1.735035	-3.441791	1.079814
14	1	0	0.879683	-4.117646	1.107439
15	1	0	2.362164	-3.681690	0.219148
16	1	0	2.310827	-3.518739	1.998974
17	6	0	2.706099	0.451426	-0.117201
18	6	0	3.560384	1.250035	0.650272
19	6	0	3.261919	-0.541179	-0.933271
20	6	0	4.935521	1.053428	0.609563
21	1	0	3.143662	2.013960	1.297631
22	6	0	4.635881	-0.742916	-0.962084
23	1	0	2.616071	-1.140988	-1.566388
24	6	0	5.478458	0.054109	-0.192102
25	1	0	5.583311	1.678192	1.213793
26	1	0	5.050247	-1.513780	-1.601709
27	1	0	6.550741	-0.099823	-0.220500
28	6	0	-2.325451	-0.562078	0.038275
29	6	0	-2.639731	-1.479990	-0.967435
30	6	0	-3.233982	-0.364093	1.083306
31	6	0	-3.835616	-2.189509	-0.925836
32	1	0	-1.942615	-1.635676	-1.780864
33	6	0	-4.428908	-1.072859	1.122085
34	1	0	-2.998550	0.354100	1.860554
35	6	0	-4.733263	-1.988070	0.117246
36	1	0	-4.064752	-2.898640	-1.713063
37	1	0	-5.123371	-0.913324	1.939043
38	1	0	-5.665333	-2.540634	0.148065
39	7	0	-2.169127	2.457834	0.064833
40	6	0	-1.917516	3.816877	0.514648
41	1	0	-1.337197	4.402300	-0.216577
42	1	0	-2.874187	4.318843	0.666575
43	1	0	-1.382136	3.804679	1.465076

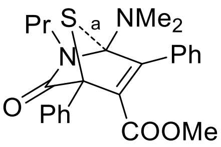

a: 2.651 Angstroms

Frequency: 116.56i

Electronic Energy (EE): -1663.37451789

Standard orientation:

Center Number	Atomic Number	Atomic Type	Coordinates (Angstroms) X	Y	Z
1	6	0	0.576135	-1.755190	-0.536497
2	6	0	1.267381	-0.780295	0.435577
3	6	0	-1.298786	-0.877604	0.580718
4	6	0	0.550554	0.557774	0.279699
5	6	0	-0.779208	0.527562	0.382406
6	16	0	0.799100	-1.459440	2.093328
7	6	0	-2.636164	-2.564840	1.636882
8	1	0	-3.281776	-3.015939	0.883411
9	1	0	-1.690222	-3.109408	1.714171
10	1	0	-3.147918	-2.603270	2.596041
11	7	0	-0.826664	-1.770299	-0.374656
12	8	0	1.125344	-2.485718	-1.316279
13	6	0	2.753261	-0.673983	0.133354
14	6	0	3.157926	-0.334950	-1.162032
15	6	0	3.721222	-0.839786	1.117848
16	6	0	4.502445	-0.159437	-1.463178
17	1	0	2.415808	-0.213181	-1.943944
18	6	0	5.070176	-0.666262	0.816673
19	1	0	3.411397	-1.099962	2.122063
20	6	0	5.465486	-0.323972	-0.470388
21	1	0	4.798527	0.098731	-2.473808
22	1	0	5.812618	-0.797783	1.595700
23	1	0	6.516012	-0.189561	-0.702051
24	6	0	-1.614453	-2.747224	-1.137807
25	6	0	-2.895353	-2.170674	-1.727987
26	1	0	-1.817982	-3.640775	-0.541879
27	1	0	-0.952899	-3.058147	-1.946587
28	6	0	-3.633079	-3.236051	-2.533563
29	1	0	-3.551408	-1.786789	-0.940556
30	1	0	-2.634193	-1.324256	-2.370568
31	1	0	-4.548433	-2.833145	-2.970607
32	1	0	-3.009680	-3.613305	-3.347993
33	1	0	-3.909929	-4.086497	-1.904111
34	6	0	1.343065	1.802210	0.022200
35	8	0	2.018841	2.347136	0.851400
36	8	0	1.217451	2.214662	-1.238951
37	6	0	1.996205	3.366888	-1.586603
38	1	0	3.055663	3.166613	-1.420785
39	1	0	1.799880	3.547132	-2.640646
40	1	0	1.687557	4.226890	-0.990451
41	6	0	-1.699698	1.665980	0.163024
42	6	0	-1.400507	2.934350	0.672435
43	6	0	-2.886924	1.487319	-0.554173
44	6	0	-2.259479	4.003116	0.443699
45	1	0	-0.509831	3.079367	1.273739
46	6	0	-3.745116	2.557261	-0.776368
47	1	0	-3.135571	0.508437	-0.947784
48	6	0	-3.432830	3.819703	-0.281304
49	1	0	-2.016218	4.979276	0.847188
50	1	0	-4.658652	2.404275	-1.339307
51	1	0	-4.103586	4.653151	-0.453550
52	7	0	-2.343146	-1.160475	1.335807
53	6	0	-2.719821	-0.247393	2.416803
54	1	0	-2.581214	0.790672	2.139001
55	1	0	-3.767133	-0.415093	2.662846
56	1	0	-2.085448	-0.476625	3.281291

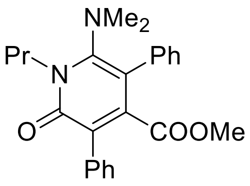

Electronic Energy (EE): -1265.23863988

Standard orientation:

Center Number	Atomic Number	Atomic Type	Coordinates (Angstroms) X	Y	Z
1	6	0	1.366263	0.469696	-0.215848
2	6	0	0.485370	-0.574890	-0.230117
3	6	0	-0.926469	-0.393406	-0.254156
4	6	0	-1.414071	0.894139	-0.332137
5	6	0	2.837101	0.279023	-0.160113
6	6	0	3.488621	-0.547848	-1.078401
7	6	0	3.592283	0.914796	0.830937
8	6	0	4.862851	-0.755610	-0.992995
9	1	0	2.922142	-1.019180	-1.873233
10	6	0	4.961632	0.704410	0.916639
11	1	0	3.097955	1.575485	1.532747
12	6	0	5.601950	-0.133917	0.006191
13	1	0	5.353532	-1.396721	-1.716430
14	1	0	5.532469	1.199725	1.693827
15	1	0	6.672116	-0.293473	0.071964
16	6	0	-3.604566	0.416825	-1.373647
17	1	0	-4.139991	-0.395626	-0.866716
18	1	0	-4.342623	1.093408	-1.816277
19	1	0	-3.002652	-0.006540	-2.176570
20	8	0	1.563204	2.831013	-0.249605
21	6	0	1.012047	-1.983443	-0.144119
22	8	0	1.104194	-2.733145	-1.080163
23	8	0	1.350888	-2.294720	1.102557
24	6	0	1.903640	-3.605441	1.282926
25	1	0	2.103030	-3.696139	2.347618
26	1	0	1.189906	-4.364084	0.960134
27	1	0	2.828707	-3.700375	0.712537
28	6	0	0.855604	1.831714	-0.245257
29	6	0	-1.012295	3.351158	-0.525743
30	6	0	-0.939656	4.224268	0.726984
31	1	0	-2.028818	3.295755	-0.909100
32	1	0	-0.371646	3.786143	-1.295183
33	6	0	-1.557378	5.592366	0.455149
34	1	0	-1.459321	3.739742	1.557150
35	1	0	0.105609	4.331395	1.018176
36	1	0	-1.482458	6.238203	1.332091
37	1	0	-1.047601	6.095640	-0.371006
38	1	0	-2.616171	5.506038	0.193053
39	7	0	-0.542038	1.968290	-0.320440
40	6	0	-1.857461	-1.534238	-0.028278
41	6	0	-1.991962	-2.573756	-0.951606
42	6	0	-2.642679	-1.555124	1.129310
43	6	0	-2.903665	-3.601323	-0.729879
44	1	0	-1.388016	-2.568369	-1.851584
45	6	0	-3.558209	-2.578485	1.348282
46	1	0	-2.529285	-0.761478	1.860749
47	6	0	-3.693926	-3.603127	0.415562
48	1	0	-3.002242	-4.397145	-1.459269
49	1	0	-4.160932	-2.579264	2.249477
50	1	0	-4.408089	-4.401056	0.583271
51	7	0	-2.766088	1.190721	-0.473521
52	6	0	-3.491207	1.808787	0.624992
53	1	0	-4.122289	2.627950	0.263136
54	1	0	-4.135284	1.069000	1.119494
55	1	0	-2.800368	2.203269	1.367316

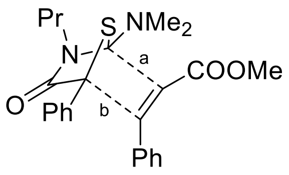

a: 2.145 Angstroms
b: 2.398 Angstroms

Frequency: 388.23i

Electronic Energy (EE): -1663.31791436

Standard orientation:

Center Number	Atomic Number	Atomic Type	Coordinates (Angstroms) X	Y	Z
1	6	0	0.072669	-1.611631	0.033523
2	6	0	-0.651714	-0.841027	-0.958923
3	6	0	1.737561	-0.328886	-0.957288
4	6	0	-0.235765	0.968480	0.559287
5	6	0	0.959464	1.219677	0.306908
6	16	0	0.443580	-0.227905	-2.156399
7	6	0	3.919728	-1.123795	-1.652600
8	1	0	3.662143	-1.517100	-2.645685
9	1	0	4.946623	-0.757075	-1.668487
10	1	0	3.851623	-1.929215	-0.927448
11	7	0	1.457495	-1.361135	-0.094780
12	8	0	-0.369117	-2.294597	0.948162
13	6	0	-2.096369	-0.856184	-1.171139
14	6	0	-2.953744	-1.513706	-0.274051
15	6	0	-2.670744	-0.136676	-2.232145
16	6	0	-4.332072	-1.443740	-0.440465
17	1	0	-2.530536	-2.064902	0.553346
18	6	0	-4.046852	-0.078325	-2.394967
19	1	0	-2.036565	0.394664	-2.934289
20	6	0	-4.888132	-0.728783	-1.495563
21	1	0	-4.975648	-1.951306	0.269399
22	1	0	-4.464122	0.482163	-3.223923
23	1	0	-5.963836	-0.679160	-1.618750
24	6	0	2.291013	-1.630501	1.085682
25	6	0	2.642576	-3.107347	1.235136
26	1	0	1.721738	-1.297281	1.959806
27	1	0	3.180235	-1.002443	1.013078
28	6	0	3.459702	-3.336401	2.502496
29	1	0	1.717145	-3.684243	1.272750
30	1	0	3.201326	-3.450499	0.359243
31	1	0	3.719800	-4.390564	2.615447
32	1	0	4.389584	-2.760591	2.487153
33	1	0	2.895475	-3.035990	3.389396
34	6	0	2.083973	2.048501	0.711711
35	6	0	3.115706	4.140713	0.491933
36	1	0	4.057551	3.712038	0.145258
37	1	0	2.912517	5.075992	-0.024549
38	1	0	3.168223	4.306996	1.568369
39	8	0	2.982911	1.680843	1.425422
40	8	0	2.021935	3.275653	0.170656
41	6	0	-1.538037	1.061468	1.142772
42	6	0	-1.848315	0.350769	2.310619
43	6	0	-2.535251	1.815321	0.504855
44	6	0	-3.130951	0.417383	2.841085
45	1	0	-1.083308	-0.251489	2.785250
46	6	0	-3.814518	1.865688	1.037628
47	1	0	-2.294229	2.349521	-0.406725
48	6	0	-4.114912	1.170937	2.207945
49	1	0	-3.363595	-0.129852	3.747273
50	1	0	-4.582458	2.442734	0.535433
51	1	0	-5.116028	1.213348	2.621185
52	7	0	3.037832	-0.014078	-1.287763
53	6	0	3.182751	1.120443	-2.190701
54	1	0	2.950547	0.845010	-3.230065
55	1	0	2.523021	1.933988	-1.887980
56	1	0	4.215782	1.468775	-2.150651

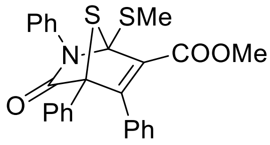

Electronic Energy (EE): -1663.38357978

Standard orientation:

Center Number	Atomic Number	Atomic Type	Coordinates (Angstroms) X	Y	Z
1	6	0	0.011707	-1.675241	0.973184
2	6	0	-0.793123	-1.071900	-0.219500
3	6	0	1.537502	-0.985659	-0.679233
4	6	0	-0.318778	0.407437	-0.230404
5	6	0	0.981409	0.454094	-0.530320
6	16	0	0.141017	-1.775571	-1.647115
7	6	0	3.890962	-0.261125	-0.967869
8	1	0	4.812088	-0.695789	-1.357091
9	1	0	3.758191	0.693722	-1.478058
10	1	0	4.033290	-0.078213	0.103971
11	7	0	1.336782	-1.659196	0.639577
12	8	0	-0.452635	-2.018783	2.034727
13	6	0	-2.271732	-1.327182	-0.197148
14	6	0	-3.014478	-0.975830	0.934125
15	6	0	-2.931335	-1.872012	-1.297353
16	6	0	-4.390112	-1.168342	0.957005
17	1	0	-2.515128	-0.547379	1.794001
18	6	0	-4.309687	-2.063901	-1.273583
19	1	0	-2.376555	-2.144197	-2.189147
20	6	0	-5.042201	-1.712110	-0.146217
21	1	0	-4.953909	-0.892506	1.840650
22	1	0	-4.805870	-2.490428	-2.137541
23	1	0	-6.115487	-1.862114	-0.125764
24	6	0	2.329050	-1.608930	1.710900
25	6	0	2.352550	-0.283317	2.470144
26	1	0	3.308824	-1.823669	1.281287
27	1	0	2.088057	-2.425739	2.395652
28	6	0	3.418170	-0.299062	3.561159
29	1	0	2.539168	0.542895	1.778466
30	1	0	1.364561	-0.116337	2.910826
31	1	0	3.426731	0.640247	4.117212
32	1	0	3.240358	-1.108908	4.274127
33	1	0	4.416053	-0.441286	3.135101
34	6	0	1.736284	1.734860	-0.690805
35	6	0	2.418678	3.356989	-2.235105
36	1	0	3.460838	3.293312	-1.918068
37	1	0	2.355707	3.497572	-3.311144
38	1	0	1.929568	4.180296	-1.713515
39	8	0	2.264503	2.352421	0.197094
40	8	0	1.735693	2.125661	-1.964777
41	6	0	-1.179655	1.557700	0.111389
42	6	0	-0.811372	2.399743	1.164742
43	6	0	-2.356977	1.821671	-0.597858
44	6	0	-1.606069	3.489996	1.503805
45	1	0	0.096951	2.190813	1.718358
46	6	0	-3.142584	2.916705	-0.261432
47	1	0	-2.650826	1.172707	-1.415224
48	6	0	-2.771130	3.750293	0.791223
49	1	0	-1.313594	4.131971	2.326554
50	1	0	-4.049012	3.119006	-0.820136
51	1	0	-3.390134	4.600339	1.054834
52	7	0	2.801804	-1.183853	-1.282655
53	6	0	3.220354	-2.578421	-1.390756
54	1	0	3.976600	-2.655182	-2.173684
55	1	0	3.640117	-2.982149	-0.460451
56	1	0	2.371968	-3.201640	-1.672730

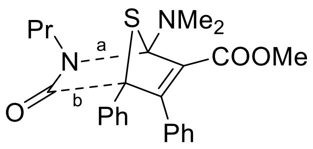

a: 2.239 Angstroms
b: 1.658 Angstroms

Frequency: 199.92i

Electronic Energy (EE): -1663.35191859

Standard orientation:

Center Number	Atomic Number	Atomic Type	Coordinates (Angstroms) X	Y	Z
1	6	0	0.009038	-1.703852	0.833541
2	6	0	-0.715237	-0.915982	-0.432509
3	6	0	1.564905	-0.449777	-1.185709
4	6	0	-0.321081	0.532290	-0.234068
5	6	0	0.948932	0.767177	-0.619366
6	16	0	0.302931	-1.471742	-1.866652
7	6	0	3.888545	0.082002	-0.768732
8	1	0	4.572656	-0.670875	-0.371556
9	1	0	4.436317	0.762606	-1.422257
10	1	0	3.471204	0.628989	0.075122
11	7	0	1.292896	-1.537099	0.752418
12	8	0	-0.713333	-2.308712	1.624683
13	6	0	-2.186210	-1.196484	-0.595289
14	6	0	-3.030248	-1.101908	0.517160
15	6	0	-2.748494	-1.493510	-1.837406
16	6	0	-4.397696	-1.303340	0.382341
17	1	0	-2.611739	-0.872226	1.487064
18	6	0	-4.119398	-1.699205	-1.970132
19	1	0	-2.125606	-1.555315	-2.722766
20	6	0	-4.948901	-1.604166	-0.860227
21	1	0	-5.034789	-1.225006	1.255591
22	1	0	-4.533019	-1.931508	-2.944623
23	1	0	-6.016324	-1.763576	-0.960669
24	6	0	2.141860	-2.137376	1.758684
25	6	0	2.366198	-1.213556	2.957276
26	1	0	3.112307	-2.379436	1.307099
27	1	0	1.700593	-3.080019	2.110400
28	6	0	3.308870	-1.827216	3.987570
29	1	0	2.765228	-0.258245	2.597805
30	1	0	1.394263	-1.001593	3.412839
31	1	0	3.460800	-1.160894	4.839658
32	1	0	2.909042	-2.770420	4.369916
33	1	0	4.289797	-2.035942	3.549761
34	6	0	1.609797	2.097282	-0.587138
35	6	0	3.033612	3.541666	-1.764133
36	1	0	3.729503	3.609448	-0.926143
37	1	0	3.573217	3.527388	-2.708042
38	1	0	2.347021	4.387687	-1.732527
39	8	0	1.525346	2.902372	0.301144
40	8	0	2.308909	2.308268	-1.711777
41	6	0	-1.229155	1.491174	0.429643
42	6	0	-0.959708	1.918492	1.730650
43	6	0	-2.385998	1.929607	-0.220066
44	6	0	-1.845403	2.772178	2.378285
45	1	0	-0.059976	1.575724	2.227507
46	6	0	-3.255156	2.802301	0.422641
47	1	0	-2.603970	1.583112	-1.224221
48	6	0	-2.990105	3.217690	1.724637
49	1	0	-1.636464	3.093652	3.391758
50	1	0	-4.145895	3.146956	-0.089095
51	1	0	-3.676188	3.888134	2.229380
52	7	0	2.832530	-0.608456	-1.508343
53	6	0	3.246865	-1.809700	-2.226495
54	1	0	2.650089	-1.937505	-3.129899
55	1	0	4.289259	-1.694435	-2.518457
56	1	0	3.135434	-2.690067	-1.585081

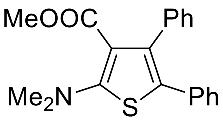

Electronic Energy (EE): -1376.82268419

Standard orientation:

Center Number	Atomic Number	Atomic Type	Coordinates (Angstroms) X	Y	Z
1	6	0	-0.779167	-0.980788	-0.059664
2	6	0	-0.050032	0.169395	-0.159265
3	6	0	1.374105	-0.063189	-0.142218
4	6	0	1.714199	-1.398274	-0.079395
5	16	0	0.262813	-2.382455	-0.003928
6	6	0	2.347463	1.052306	-0.181759
7	8	0	2.319926	1.979250	-0.950453
8	8	0	3.279930	0.932589	0.780667
9	6	0	4.314128	1.919565	0.757392
10	1	0	4.836383	1.906547	-0.201195
11	1	0	3.897748	2.913565	0.924060
12	1	0	4.994623	1.656391	1.564206
13	6	0	-2.233004	-1.175030	0.092792
14	6	0	-2.883397	-2.205885	-0.594966
15	6	0	-2.986887	-0.349606	0.936838
16	6	0	-4.252041	-2.403839	-0.448835
17	1	0	-2.315496	-2.845204	-1.262554
18	6	0	-4.355149	-0.543976	1.073387
19	1	0	-2.494633	0.441770	1.489424
20	6	0	-4.993491	-1.570855	0.382050
21	1	0	-4.739310	-3.205315	-0.991944
22	1	0	-4.924067	0.104336	1.729846
23	1	0	-6.060996	-1.721408	0.492951
24	6	0	4.074577	-1.343947	-0.795702
25	1	0	4.715061	-0.881669	-0.037296
26	1	0	4.661592	-2.081226	-1.349023
27	1	0	3.745722	-0.579877	-1.501566
28	6	0	-0.657345	1.522701	-0.191437
29	6	0	-0.294399	2.483296	0.756199
30	6	0	-1.624675	1.843821	-1.145549
31	6	0	-0.894151	3.737031	0.755650
32	1	0	0.451067	2.241091	1.507414
33	6	0	-2.225003	3.097955	-1.145717
34	1	0	-1.906790	1.103391	-1.885902
35	6	0	-1.863154	4.047042	-0.194454
36	1	0	-0.606601	4.471674	1.499002
37	1	0	-2.974212	3.334766	-1.892386
38	1	0	-2.331134	5.024664	-0.195389
39	7	0	2.924313	-2.019770	-0.226295
40	6	0	3.215125	-3.220198	0.538610
41	1	0	3.513196	-2.983338	1.567534
42	1	0	2.349162	-3.882478	0.572307
43	1	0	4.028424	-3.759857	0.051978

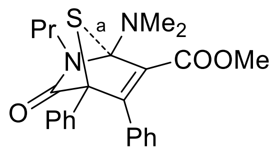

a: 2.619 Angstroms

Frequency: 114.98i

Electronic Energy (EE): -1663.37882599

Standard orientation:

Center Number	Atomic Number	Atomic Type	Coordinates (Angstroms) X	Y	Z
1	6	0	0.412148	-1.788233	0.530205
2	6	0	-0.583470	-1.107687	-0.438798
3	6	0	1.854993	-0.305615	-0.577690
4	6	0	-0.402623	0.409607	-0.252355
5	6	0	0.871931	0.809287	-0.376503
6	16	0	0.106916	-1.509123	-2.112373
7	6	0	3.836990	-1.306963	-1.503590
8	1	0	4.333980	-1.171899	-2.462231
9	1	0	4.598089	-1.380633	-0.725046
10	1	0	3.234057	-2.217596	-1.540024
11	7	0	1.727252	-1.336987	0.350763
12	8	0	0.137371	-2.643163	1.330066
13	6	0	-1.995794	-1.593296	-0.158112
14	6	0	-2.534391	-1.423390	1.121750
15	6	0	-2.787026	-2.171229	-1.144243
16	6	0	-3.837844	-1.806248	1.401994
17	1	0	-1.929522	-0.981041	1.905947
18	6	0	-4.097300	-2.558230	-0.865844
19	1	0	-2.372972	-2.312997	-2.134945
20	6	0	-4.627774	-2.374048	0.403999
21	1	0	-4.238832	-1.663725	2.399006
22	1	0	-4.699900	-3.005926	-1.648107
23	1	0	-5.647198	-2.673043	0.620568
24	6	0	2.795024	-1.824441	1.235576
25	6	0	3.595664	-0.698444	1.881054
26	1	0	3.446875	-2.525584	0.709043
27	1	0	2.275233	-2.397688	2.003671
28	6	0	4.695056	-1.269645	2.770758
29	1	0	4.030524	-0.044133	1.120335
30	1	0	2.914532	-0.075084	2.467749
31	1	0	5.403354	-1.867641	2.190058
32	1	0	5.255683	-0.471168	3.260030
33	1	0	4.277556	-1.912705	3.550104
34	6	0	1.408388	2.163882	-0.095519
35	6	0	0.993790	4.466781	-0.164190
36	1	0	1.972232	4.720446	-0.573704
37	1	0	0.227479	5.115697	-0.580707
38	1	0	1.019047	4.557704	0.922799
39	8	0	2.462240	2.353380	0.464322
40	8	0	0.619982	3.137667	-0.541998
41	6	0	-1.537950	1.295424	0.085576
42	6	0	-1.532122	2.036816	1.268210
43	6	0	-2.641752	1.364787	-0.767010
44	6	0	-2.620436	2.837170	1.597325
45	1	0	-0.681028	1.970766	1.939541
46	6	0	-3.720888	2.176575	-0.442185
47	1	0	-2.648187	0.782030	-1.681344
48	6	0	-3.715529	2.909049	0.741815
49	1	0	-2.614910	3.400202	2.523479
50	1	0	-4.571821	2.229119	-1.111081
51	1	0	-4.565447	3.530677	0.999167
52	7	0	2.955840	-0.161407	-1.281805
53	6	0	3.074239	0.892876	-2.285726
54	1	0	2.965315	0.431201	-3.271276
55	1	0	2.293975	1.639429	-2.184934
56	1	0	4.052898	1.366538	-2.200947

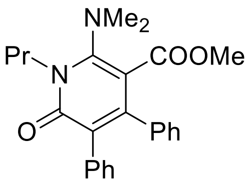

Electronic Energy (EE): -1265.24264474

Standard orientation:

Center Number	Atomic Number	Atomic Type	Coordinates (Angstroms) X	Y	Z
1	6	0	0.466885	-1.114736	0.193096
2	6	0	0.463213	0.250754	0.086045
3	6	0	-0.772410	0.971982	0.149441
4	6	0	-1.960021	0.301051	0.290934
5	6	0	1.713043	-1.927165	0.191064
6	6	0	2.726194	-1.685421	1.121109
7	6	0	1.890293	-2.943527	-0.750912
8	6	0	3.899324	-2.432001	1.100762
9	1	0	2.594524	-0.906653	1.864224
10	6	0	3.066729	-3.683020	-0.778376
11	1	0	1.100720	-3.152059	-1.463211
12	6	0	4.075109	-3.429955	0.147286
13	1	0	4.675113	-2.234732	1.831661
14	1	0	3.193358	-4.463036	-1.520469
15	1	0	4.989992	-4.011016	0.129113
16	6	0	-3.802939	1.172607	1.605193
17	1	0	-3.434999	2.123204	2.010709
18	1	0	-4.892219	1.213282	1.519221
19	1	0	-3.539803	0.376508	2.303360
20	8	0	-0.868573	-3.066239	0.365512
21	6	0	-0.787837	-1.846716	0.322250
22	6	0	-3.220478	-1.830805	0.410073
23	6	0	-3.677522	-2.183297	-1.001881
24	1	0	-3.982709	-1.250468	0.921323
25	1	0	-3.024302	-2.737264	0.980263
26	6	0	-4.942231	-3.034769	-0.968292
27	1	0	-3.857722	-1.256563	-1.555755
28	1	0	-2.872887	-2.723145	-1.507946
29	1	0	-5.273593	-3.286369	-1.977571
30	1	0	-4.768097	-3.971067	-0.431707
31	1	0	-5.760799	-2.508103	-0.469432
32	6	0	-0.778688	2.463549	0.128638
33	8	0	-0.258109	2.949827	-0.999473
34	8	0	-1.199969	3.159588	1.019130
35	6	0	-0.078741	4.368538	-1.043146
36	1	0	0.569858	4.690489	-0.226662
37	1	0	0.388577	4.576907	-2.002608
38	1	0	-1.040291	4.878311	-0.967778
39	7	0	-1.959161	-1.068458	0.391669
40	6	0	1.734714	1.019789	-0.052517
41	6	0	2.095118	1.956297	0.917517
42	6	0	2.571497	0.813146	-1.149110
43	6	0	3.283401	2.670409	0.795793
44	1	0	1.454763	2.114134	1.779800
45	6	0	3.752387	1.534398	-1.274472
46	1	0	2.295268	0.081548	-1.900309
47	6	0	4.113027	2.462575	-0.301249
48	1	0	3.561702	3.384718	1.562249
49	1	0	4.395110	1.366566	-2.130952
50	1	0	5.039186	3.017611	-0.396020
51	7	0	-3.225023	0.905308	0.290267
52	6	0	-3.511917	1.939254	-0.693829
53	1	0	-4.583256	1.915280	-0.912566
54	1	0	-3.260975	2.949343	-0.347207
55	1	0	-2.972918	1.731600	-1.619146
